# Development of pharmacological immunoregulatory anti-cancer therapeutics: current mechanistic studies and clinical opportunities

**DOI:** 10.1038/s41392-024-01826-z

**Published:** 2024-05-22

**Authors:** Nanhao Yin, Xintong Li, Xuanwei Zhang, Shaolong Xue, Yu Cao, Gabriele Niedermann, You Lu, Jianxin Xue

**Affiliations:** 1 Division of Thoracic Tumor Multimodality Treatment, Cancer Center & State Key Laboratory of Biotherapy, and The National Clinical Research Center for Geriatrics, West China Hospital, Sichuan University, No. 37, Guoxue Lane, Chengdu, 610041 Sichuan PR China; 2 Department of Gynecology and Obstetrics, West China Second University Hospital, Sichuan University, No. 20, Section 3, South Renmin Road, Chengdu, 610041 Sichuan PR China; 3 Department of Emergency Medicine, Laboratory of Emergency Medicine, West China Hospital, Sichuan University, No. 37, Guoxue Lane, Chengdu, 610041 Sichuan PR China; 4 Institute of Disaster Medicine & Institute of Emergency Medicine, Sichuan University, No. 17, Gaopeng Avenue, Chengdu, 610041 Sichuan PR China; 5 Department of Radiation Oncology, Medical Center—University of Freiburg, Faculty of Medicine, University of Freiburg, German Cancer Consortium (DKTK) Partner Site DKTK-Freiburg, Robert-Koch-Strasse 3, 79106 Freiburg, Germany; 6 Laboratory of Clinical Cell Therapy, West China Hospital, Sichuan University, No. 2222, Xinchuan Road, Chengdu, 610041 Sichuan PR China

**Keywords:** Tumour immunology, Tumour immunology

## Abstract

Immunotherapy represented by anti-PD-(L)1 and anti-CTLA-4 inhibitors has revolutionized cancer treatment, but challenges related to resistance and toxicity still remain. Due to the advancement of immuno-oncology, an increasing number of novel immunoregulatory targets and mechanisms are being revealed, with relevant therapies promising to improve clinical immunotherapy in the foreseeable future. Therefore, comprehending the larger picture is important. In this review, we analyze and summarize the current landscape of preclinical and translational mechanistic research, drug development, and clinical trials that brought about next-generation pharmacological immunoregulatory anti-cancer agents and drug candidates beyond classical immune checkpoint inhibitors. Along with further clarification of cancer immunobiology and advances in antibody engineering, agents targeting additional inhibitory immune checkpoints, including LAG-3, TIM-3, TIGIT, CD47, and B7 family members are becoming an important part of cancer immunotherapy research and discovery, as are structurally and functionally optimized novel anti-PD-(L)1 and anti-CTLA-4 agents and agonists of co-stimulatory molecules of T cells. Exemplified by bispecific T cell engagers, newly emerging bi-specific and multi-specific antibodies targeting immunoregulatory molecules can provide considerable clinical benefits. Next-generation agents also include immune epigenetic drugs and cytokine-based therapeutics. Cell therapies, cancer vaccines, and oncolytic viruses are not covered in this review. This comprehensive review might aid in further development and the fastest possible clinical adoption of effective immuno-oncology modalities for the benefit of patients.

## Introduction

**Fig. 1:**
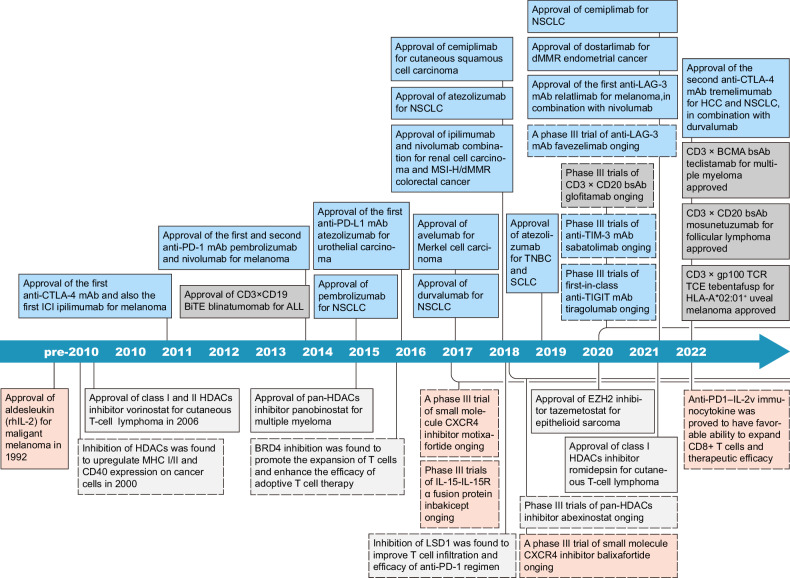
Timeline showing representative events of drug approval, clinical trials, and key biological discovery for immunoregulatory targets of anti-cancer therapeutics. Events involving immunoregulatory receptors and bispecific antibodies are in blue and dark gray boxes and those related to immuno-epigenetics and cytokines are in light gray and pink boxes, respectively. Boxes with solid lines indicate approvals, while other events are in dashed line boxes. Lines with rounded corners indicate the period of clinical trials. The approvals include the final approval, the accelerated approval by FDA, and the approval by EMA. Cancer stage descriptions such as advanced or metastatic, pathological subtypes, and details of combination therapies are omitted. ICI immune checkpoint inhibitors, mAb monoclonal antibody, BiTE bispecific T cell engager, ALL acute lymphoid leukemia, NSCLC non-small cell lung cancer, MSI-H microsatellite instability-high, dMMR deficient mismatch repair, TNBC triple-negative breast cancer, SCLC small cell lung cancer, bsAb bispecific antibody, HCC hepatocellular carcinoma, TCR T-cell receptor, TCE T cell engager, rhIL-2 recombinant human interleukin-2

Immunotherapies attempt to harness the innate and adaptive immune system to attack cancer cells.^1^ Since early systematic clinical applications of immunotherapy in oncology, such as the use of Coley’s bacterial toxin for sarcoma more than 100 years ago and Bacillus Calmette-Guérin vaccine for bladder cancer in the 1970s,^2^ there has been an exponential evolution accelerated by the epochal FDA approvals of the first immune checkpoint inhibitors (ICIs), the antibody ipilimumab against anti-cytotoxic T lymphocyte-associated antigen 4 (CTLA-4) in 2011 and the first antibodies against anti-programmed cell death protein 1 (PD-1) pembrolizumab and nivolumab in 2014^3^ (Fig. 1).

Despite the remarkable success achieved by ICIs and ICI-based treatment combinations in some tumor entities,^4–20^ many patients are unresponsive or experience weak responses^21–23^ and immune-related adverse events (irAEs),^24,25^ stressing the need for novel immunomodulatory strategies. Multiple host intrinsic and extrinsic factors associated with ICI response and toxicity have been reported, providing insights for the development of next-generation immunotherapeutics.^26^ It would be advantageous if next-generation immunotherapeutics had distinct mechanisms of action compared to classical anti-PD-(L)1 and anti-CTLA-4 antibodies and showed significant single-agent anti-tumor efficacy or enhanced the efficacy and safety of classical immunotherapeutics. Although many drug candidates and associated mechanisms already have received immense research interest, some research areas are still in the early stages of mechanistic exploration and therapeutic development, e.g., regarding aging, obesity, microbiota, and other systemic and host extrinsic factors.^26^ Certain drug candidates have already progressed significantly into pharmacological development and relevant therapeutic strategies have evolved with great clinical potential, as indicated by recent clinical trial results. Considering the large number of immunomodulatory agents under development, identifying the dominant drivers of anti-tumor immunity within the complex anti-tumor immune network remains one of the top challenges in selecting major therapeutic targets and optimizing treatment combinations.^27^ Extensive assessment of biological patient parameters to establish predictive biomarkers and the use of analytical platforms^28^ are important to handle inter- and intra-patient tumor heterogeneity. This requires a deep understanding but also a panoramic grasp of the current knowledge of mechanisms of anti-tumor immunity as well as of established and potential therapeutic targets and immunomodulatory agents.

Therefore, in this review we summarize recent advances in mechanistic exploration and drug development of therapeutics targeting relevant anti-tumor immunomodulatory molecules (Fig. 1). Our study is based mainly on articles published between 2017–2022, reports from recent annual meetings of the American Society of Clinical Oncology, American Association for Cancer Research, European Society for Medical Oncology, Society for Immunotherapy of Cancer, and a comprehensive analysis of clinical trial databases. We do not describe previously developed drugs that have been removed from the pipelines. Our statistics are up to December 2022.

First, we present a comprehensive update on the biology and drug development related to immune checkpoints and co-stimulatory molecules, highlighting additional inhibitory immune checkpoints beyond PD-1/programmed cell death ligand 1 (PD-L1) and CTLA-4. We then summarize the current state of the development of bi-specific antibodies (bsAbs) and multi-specific antibodies (msAbs) in immuno-oncology. Lastly, we discuss recent advances in exploiting epigenetics and cytokines for the development of immunomodulatory anti-tumor therapeutics. In each section, we discuss the biology and functions of the respective immune targets in cancer and the developmental status and clinical trial data of agents acting on these targets.

## Inhibitory checkpoints and protein families

**Fig. 2:**
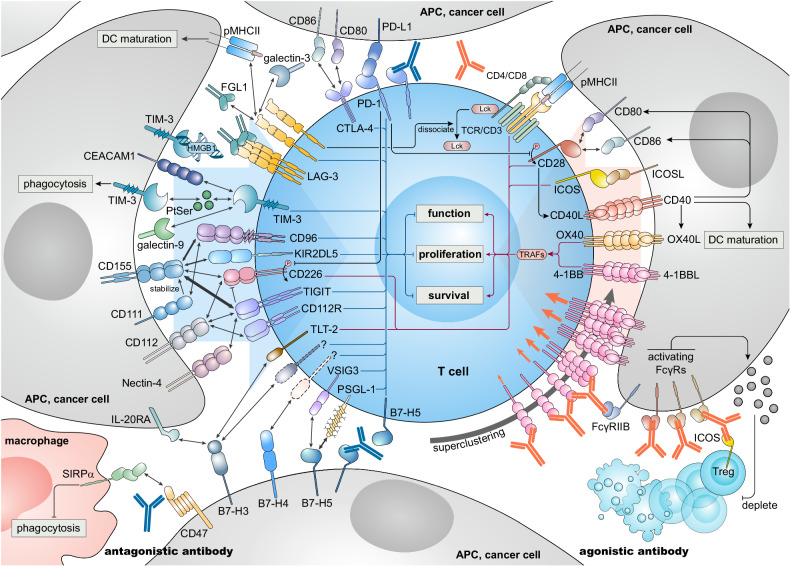
Antagonistic and agonistic antibodies act on inhibitory immune checkpoints and co-stimulatory molecules respectively to promote anti-tumoral T cell functions. By blocking the inhibitory receptor-ligand binding process, antagonistic antibodies prevent the activation of inhibitory downstream signaling in T cells, thereby sustaining the function, proliferation, and survival of T cells (left panel). Beyond the two first confirmed receptor-ligand pairs, PD-1-PD-L1 and CTLA-4-CD80/CD86, more and more immune checkpoint receptor-ligand pairs have been identified. Besides the interaction between specific cognate receptor and ligand pairs, inter-relationships exist between certain immune checkpoints such as PD-1 and TIGIT/CD226. With a certain amount of redundancy and robustness, these inhibitory circuits guarantee balanced T cell immunity under physiological conditions. However, these mechanisms are utilized by cancer cells and the inhibitory TME to limit anti-tumor immunity. By promoting trimerization and superclustering, agonistic antibodies promote and amplify downstream signaling of co-stimulatory molecules to sustain the function, proliferation, and survival of T cells (right panel). Likewise, by upregulating the expression of receptors and ligands, inter-relationships also exist between co-stimulatory molecules such as CD28 and CD40. Another strategy using agonistic antibodies is modifying the Fc segment to predispose agonists to bind activating FcγRs for Treg depletion. DC Dendritic cell, cGAS-STING cyclic GMP-AMP synthase-stimulator of interferon genes, pMHCII peptide major histocompatibility complex class II, PD-1 programmed cell death protein 1, PD-L1 programmed death-ligand 1, FGL1 fibrinogen-like protein 1, HMGB1 high mobility group box 1, CTLA-4 cytotoxic T-lymphocyte-associated protein 4, LAG-3 lymphocyte-activation gene 3, TIM-3 T-cell immunoglobulin and mucin-domain containing-3, CEACAM1 carcinoembryonic antigen-related cell adhesion molecule 1, KIR2DL5 killer cell immunoglobulin-like receptor, two Ig domains and long cytoplasmic tail 5, Eomes eomesodermin, TIGIT T-cell immunoreceptor with Ig and ITIM domains, PtSer phosphatidylserine, TLT-2 triggering receptor expressed on myeloid cells 2, PSGL-1 P-selectin glycoprotein ligand-1, SIRPα signaling-regulatory protein α, APC antigen-presenting cell, NF-κB nuclear factor kappa B, ICOS inducible T-cell costimulator, ICOSL inducible T-cell costimulator ligand, FcγR Fc gamma receptor, Treg regulatory T cell

Besides the canonical immune checkpoints PD-1 and CTLA-4, alternative negative regulatory checkpoints have been found and are focused on by cancer biologists, clinical oncologists, and industry. Biology and therapeutic potential of the immunoglobulin (Ig) superfamily (IgSF) members, including LAG-3, TIM-3, TIGIT, CD47/SIRPα, B7 family members, and others such as leukocyte Ig-like receptor family, butyrophilin family, and sialic acid-binding Ig-type lectins, are increasingly found to be important in T cell-mediated anti-tumor immunity. In addition, the more recent development of PD-(L)1 and CTLA-4 inhibitors has sought to generate new agents that can overcome shortcomings of currently used ICIs. These inhibitory molecules are involved in intricate networks illustrated in Fig. 2.

### Additional checkpoints: LAG-3, TIM-3, and TIGIT

#### LAG-3: biology, drug development, and therapeutic efficacy

Lymphocyte activation gene 3 (LAG-3, CD223) is a membrane protein sharing homology with CD4.^29^ It is expressed on T cells, regulatory T cells (Tregs), B cells, natural killer (NK) cells, and myeloid cells. Upon activation, its expression is elevated on T cells to prevent autoimmunity in concert with PD-1^30^ and is also one of the hallmarks of exhausted CD8^+^ T cells.^31,32^ By selectively recognizing stable complexes of peptide and MHC class II (pMHCII), LAG-3 inhibits the activity and expansion of CD4^+^ effector T cells and antigen-specific CD8^+^ T cells^33–37^ (Fig. 2). LAG-3 blockade rescues accumulation and functions of T cells,^38–40^ especially with PD-1 blockade.^41,42^ Another ligand of LAG-3, galectin-3 also suppresses CD8^+^ T cells and inhibits immunostimulatory plasmacytoid dendritic cells.^43^ Fibrinogen-like protein 1 (FGL1) also binds LAG-3 and mediates clustering of membrane LAG-3-FGL1 complexes,^44^ but pMHCII rather than FGL1 may be the major functional ligand for LAG-3-mediated immunosuppression.^45^ Surprisingly, LAG-3 can move to the immune synapse and dissociate the tyrosine kinase Lck from the CD4/CD8 co-receptor to attenuate T cell receptor (TCR) signaling, even without ligands^46^ (Fig. 2). In contrast, LAG-3 induces dendritic cell (DC) activation and maturation by ligating MHC class II on DCs,^47^ and soluble LAG-3 (LAG-3-Ig fusion protein) is feasible to stimulate T cells indirectly by antigen presenting cells (APCs).^48^

Expression of LAG-3 on tumor-infiltrating lymphocytes (TILs) and peripheral blood cells correlates with early recurrence and poor prognosis in patients who received anti-PD-1 ± anti-CTLA-4 regimen.^49–51^ Development of drugs targeting LAG-3 focuses on anti-LAG-3 monoclonal antibodies (mAbs), LAG-3-Ig fusion proteins, and bsAbs/msAbs targeting LAG-3 (Table 1 and Supplementary Table 1). Anti-LAG-3 agent monotherapy was not as effective as anti-PD-1 antibody in preclinical models but showed synergy with anti-PD-1 antibody.^42^ Of note, the first anti-LAG-3 antibody entered into phase III, relatlimab, received FDA approval according to higher median PFS (mPFS) of 10.1 months in combination with nivolumab, compared to 4.6 months observed with nivolumab monotherapy in the RELATIVITY-047 study in melanoma patients; moreover, the relatlimab/nivolumab combination appeared to be less toxic compared to nivolumab plus plus ipilimumab.^52^ Other combinations like miptenalimab plus ezabenlimab and favezelimab plus pembrolizumab are still under phase I exploration (Table 1 and Supplementary Table 1). LAG-3-Ig fusion protein eftilagimod alpha plus pembrolizumab caused an overall response rate (ORR) of 33% and 50% in pembrolizumab-refractory and anti-PD-1 naïve non-small cell lung cancer (NSCLC) patients, respectively,^53^ and showed a similarly considerable effect^54^ in head and neck squamous cell carcinoma (HNSCC) patients, thus was granted fast track status by FDA for NSCLC and HNSCC. The LAG-3 pathway has thus now been established as the third immune checkpoint pathway that can be inhibited to stimulate anti-tumor immune responses with clinical benefit.

**Table 1:** Therapeutics targeting inhibitory immune checkpoints and co-stimulatory molecules

Basic information					Representative trial					
Target	Agent	Manufacturer	Agent type	Highest developmental phase	Phase	Cancer type	Therapeutic combination	Study	Identifier	Status
Additional inhibitory checkpoints										
LAG-3 pathway										
LAG-3	Relatlimab (BMS-986016)	Bristol-Myers Squibb	Antagonistic mAb	FDA approved	III	Melanoma	Anti-PD-1 (Nivolumab)	RELATIVITY-047	NCT03470922	Active, not recruiting
	Miptenalimab (BI 754111)	Boehringer Ingelheim	Antagonistic mAb	II	II	Solid Tumors	anti-PD-1 (Ezabenlimab), VEGF/Ang2 inhibitor (BI 836880)		NCT03697304	Recruiting
	Favezelimab (MK4280)	Merck Sharp & Dohme	Antagonistic mAb	III	III	CRC	Anti-PD-1 (Pembrolizumab)	MK-4280A-007	NCT04938817	Not yet recruiting
	Fianlimab (REGN3767)	Regeneron	Antagonistic mAb	II	II	Breast Cancer	Anti-PD-1 (Cemiplimab)	I-SPY	NCT01042379	Recruiting
	Leramilimab (LAG525, IMP-701)	Immutep Limited, Novartis	Antagonistic mAb	II	II	Breast Cancer	Anti-PD-1 (Spartalizumab), chemotherapy		NCT03499899	Active, not recruiting
	Encelimab (TSR-033)	AnaptysBio, GlaxoSmithKline	Antagonistic mAb	I	I	Solid Tumors	Anti-PD-1 (Dostarlimab), chemotherapy	CITRINO	NCT03250832	Active, not recruiting
	Sym 022	Symphogen	Antagonistic mAb	I	I	Solid Tumors	Anti-PD-1 (Sym021), anti-TIM-3 (Sym023)		NCT03311412	Recruiting
	IBI-110	Innovent Biologics	Antagonistic mAb	II	II	SCLC	Anti-PD-1 (Sintilimab), chemotherapy		NCT05026593	Recruiting
	INCAGN02385/INCAGN 2385	Agenus	Antagonistic mAb	I/II	I/II	Melanoma	Anti-PD-1 (INCMGA00012), anti-TIM-3 (INCAGN02390)		NCT04370704	Recruiting
	SHR-1802	Jiangsu Hengrui	Antagonistic mAb	I	I	Malignancies	–		NCT04414150	Recruiting
	LBL-007	Leads Biolabs	Antagonistic mAb	I	I	Melanoma	Anti-PD-1 (Toripalimab)		NCT04640545	Recruiting
	Eftilagimod alpha (IMP 321)	Immutep/Merck & Co	LAG-3-Ig fusion protein	II	II	NSCLC, HNSCC	Anti-PD-1 (Pembrolizumab)	TACTI-002	NCT03625323	Recruiting
TIM-3 pathway										
TIM-3	Cobolimab (TSR-022)	AnaptysBio/Glaxo SmithKline	Antagonistic mAb	II	II	NSCLC	Anti-PD-1 (Dostarlimab), chemotherapy	COSTAR Lung	NCT04655976	Recruiting
	Sabatolimab (MBG-453)	Novartis	Antagonistic mAb	III	III	MDS, CMML	Chemotherapy	STIMULUS-MDS2	NCT04266301	Recruiting
	INCAGN2390	Agenus	Antagonistic mAb	I/II	I/II	Melanoma	Anti-PD-1 (Retifanlimab/INCMGA00012), anti-LAG-3 (INCAGN02385/INCAGN 2385)		NCT04370704	Recruiting
	BGB-A425	BeiGene	Antagonistic mAb	I/II	I/II	Solid Tumors	Anti-PD-1 (Tislelizumab)		NCT03744468	Recruiting
	SHR-1702	Jiangsu Hengrui	Antagonistic mAb	I	I	Solid Tumors	Anti-PD-1 (Camrelizumab)		NCT03871855	Not yet recruiting
	LY3321367	Eli Lilly and Company	Antagonistic mAb	I	I	Solid Tumors	Anti-PD-L1 (LY3300054)	PACT	NCT02791334	Active, not recruiting
	Sym023	Symphogen	Antagonistic mAb	I	I	BTC	Anti-PD-1 (Sym021), anti-LAG-3 (INCAGN02385/INCAGN 2385)		NCT04641871	Active, not recruiting
	BMS-986258	Bristol-Myers Squibb	Antagonistic mAb	I/II	I/II	Solid Tumors	Anti-PD-1 (Nivolumab), Recombinant human hyaluronidase (rHuPH20)		NCT03446040	Recruiting
CEACAM-1	CM24	Purple Biotech, Bristol-Myers Squibb	Antagonistic mAb	I/II	I/II	Solid Tumors	Anti-PD-1 (Nivolumab), chemotherapy		NCT04731467	Recruiting
galecting-9	LYT-200	PureTech	Antagonistic mAb	I/II	I/II	Solid Tumors	Anti-PD-1, chemotherapy		NCT04666688	Recruiting
TIGIT pathway										
TIGIT	Vibostolimab (MK-7684)	Merck Sharp & Dohme	Antagonistic mAb	III	III	NSCLC	Anti-PD-1 (Pembrolizumab)	MK-7684A-003	NCT04738487	Recruiting
	Tiragolumab (MTIG-7192A)	Genentech, Roche	Antagonistic mAb	III	III	NSCLC	Anti-PD-1 (Atezolizumab)	SKYSCRAPER-01	NCT04294810	Recruiting
	Ociperlimab (BGB-A1217)	BeiGene	Antagonistic mAb	III	III	NSCLC	Anti-PD-1 (Tislelizumab), chemotherapy	AdvanTIG-301	NCT04866017	Recruiting
	Etigilimab (OMP-313M32)	Mereo BioPharma, OncoMed	Antagonistic mAb	II	II	Fallopian Tube Cancer, Ovarian Cancer, Peritoneal Cancer	Anti-PD-1 (Nivolumab)	EON	NCT05026606	Recruiting
	BMS-986207	Bristol-Myers Squibb, Compugen	Antagonistic mAb	II	I/II	Solid Tumors	Anti-PD-1 (Nivolumab), anti-CD112R (COM701)		NCT04570839	Recruiting
	Domvanalimab (AB154)	Arcus Biosciences	Antagonistic mAb	III	III	NSCLC	Anti-PD-1 (Zimberelimab/AB122)	ARC-10	NCT04736173	Recruiting
	SEA-TGT (SGN-TGT)	Seattle Genetics, Seagen	Antagonistic mAb	I/II	I/II	NSCLC	Anti-PD-1 (Sasanlimab), VEGFR1/2/3 TKI (Axitinib)		NCT04585815	Recruiting
	IBI-939	Innovent Biologics	Antagonistic mAb	I	I	NSCLC	Anti-PD-1 (Sintilimab)		NCT04672369	Not yet recruiting
	JS006	Shanghai Junshi	Antagonistic mAb	I	I	Malignancies	Anti-PD-1 (Toripalimab)		NCT05061628	Recruiting
	AK127	Akeso Biopharma	Antagonistic mAb	I	I	Solid Tumors	PD-1 x CTLA-4 bsAb (AK104)		NCT05021120	Not yet recruiting
CD155	PVSRIPO (Lerapolturev)	Istari Oncology, Duke University Medical Center	Recombinant polio virus (act by biology of CD155)	II	II	Glioblastoma	Anti-PD-1 (Pembrolizumab)	LUMINOS-101	NCT04479241	Active, not recruiting
	EOS884448 (EOS-448, GSK-4428859)	iTeos Therapeutics	Antagonistic mAb	II	II	NSCLC	Anti-PD-1 (Dostarlimab)		NCT03739710	Recruiting
CD96	GSK6097608	GlaxoSmithKline	Antagonistic mAb	I	I	Solid Tumors	Anti-PD-1 (Dostarlimab)		NCT04446351	Recruiting
CD112R	COM701 (CGEN-15029)	Compugen	Antagonistic mAb	I/II	I/II	Solid Tumors	Anti-PD-1 (Nivolumab), anti-TIGIT (BMS-986207)		NCT04570839	Recruiting
Nectin-4	Enfortumab Vedotin	Astellas, Merck & Co, Seagen	ADC	III	III	Ureteral Cancer	–	EV-301	NCT03474107	Active, not recruiting
	BT8009	Bicycle Therapeutics	Bicycle toxin conjugate	I/II	I/II	Solid Tumorss	Anti-PD-1(Nivolumab)		NCT04561362	Recruiting
CD47-SIRPα pathway										
CD47	Magrolimab (Hu5F9-G4)	Gilead	Antagonistic mAb	III	III	AML	Chemotherapy	ENHANCE-3	NCT05079230	Active, not recruiting
	Lemzoparlimab (TJ011133, TJC4)	I-Mab Biopharma	Antagonistic mAb	III	III	MDS	Chemotherapy		NCT05709093	Recruiting
	Ligufalimab (AK117)	Akeso	Antagonistic mAb	II	I/II	Malignancies	Anti-PD-1/VEGF (AK112), chemotherapy		NCT05229497	Recruiting
	Letaplimab (IBI188)	Innovent Biologics (Suzhou) Co. Ltd.	Antagonistic mAb	I	I	Malignancies	–		NCT03763149	Completed
	Urabrelimab (SRF231)	Surface Oncology	Antagonistic mAb	I	I	Malignancies	–		NCT03512340	Completed
	ZL-1201	Zai Lab (Shanghai) Co., Ltd.	Antagonistic mAb	I	I	Advanced Cancer	–		NCT04257617	Completed
	AO-176	Arch Oncology	Antagonistic mAb	I/II	I/II	Ovarian Cancer	Anti-PD-1 (Pembrolizumab), chemotherapy	KEYNOTE-C49	NCT03834948	Completed
	STI-6643	Sorrento Therapeutics, Inc.	Antagonistic mAb	I	I	Solid Tumors	–		NCT04900519	Recruiting
	CC-90002	Celgene	Antagonistic mAb	I	I	Malignancies	Anti-CD20 (Rituximab)		NCT02367196	Completed
	Evorpacept (ALX148)	ALX Oncology Inc.	Anti-CD47-Fc fusion protein	II/III	II/III	GC, GEJC	Anti-HER2 (Trastuzumab), anti-EGFR (Ramucirumab), chemotherapy	ASPEN-06	NCT05002127	Recruiting
	Maplirpacept (TTI-622, PF-07901801)	Pfizer	Anti-CD47-Fc fusion protein	II	I/II	DLBCL	Anti-CD19 (Tafasitamab), Lenalidomide		NCT05626322	Recruiting
	Nibrozetone (RRx-001)	EpicentRx, Inc.	Small molecule MYC and CD47 downregulator	III	III	SCLC	Chemotherapy	REPLATINUM	NCT05566041	Active, not recruiting
SIRPα	BI 765063 (OSE-172)	Boehringer Ingelheim	Antagonistic mAb	I	I	Solid Tumors	Anti-PD-1 (BI 754091)		NCT03990233	Active, not recruiting
	Ontorpacept (TTI-621)	Pfizer	SIRPα-Fc fusion protein	II	II	DLBCL	Anti-PD-1 (Pembrolizumab)		NCT05507541	Recruiting
	DSP107	Kahr Medical	SIRPα-4-1BBL bifunctional fusion protein	I/II	I/II	Solid Tumors	Anti-PD-L1 (Atezolizumab)		NCT04440735	Recruiting
	IMM01	ImmuneOnco Biopharmaceuticals (Shanghai) Inc.	SIRPα-Fc fusion protein	I/II	I/II	AML, MDS	Azacitidine		NCT05140811	Recruiting
	IMM0306	ImmuneOnco Biopharmaceuticals (Shanghai) Inc.	SIRPα-anti-CD20 fusion protein	I/II	I/II	B-cell NHL	Lenalidomide		NCT05771883	Not yet recruiting
B7 family proteins										
B7-H3	MGC018	MacroGenics	ADC	I/II	I/II	Solid tumors	Anti-PD-1 (Retifanlimab)		NCT03729596	Recruiting
	mirzotamab clezutoclax (ABBV-155)	AbbVie	ADC	I	I	Solid tumors	Chemotherapy		NCT03595059	Recruiting
	DS-7300a	Daiichi Sankyo	ADC	I/II	I/II	Solid tumors	–		NCT04145622	Recruiting
B7-H4	Alsevalimab (FPA-150)	Amgen, Five Prime Therapeutics	Antagonistic mAb	I	I	Solid tumors	Anti-PD-1 (Pembrolizumab)	FPA150-001	NCT03514121	Completed
	NC762	NextCure	Antagonistic mAb	I/II	I/II	Solid tumors	–		NCT04875806	Recruiting
B7-H5	HMBD-002	Hummingbird Bioscience	Antagonistic mAb	I	I	Solid tumors	Anti-PD-1 (Pembrolizumab)		NCT05082610	Recruiting
	Onvatilimab (JNJ-61610588, CI-8993, VSTB112)	Curis, Janssen, ImmuNext	Antagonistic mAb	I	I	Solid tumors	–		NCT04475523	Recruiting
	CA-170	Aurigene Discovery Technologies	Small molecule inhibitor	II	II	Solid tumors and lymphomas			CTRI/2017/12/011026	Completed
Co-stimulatory molecules										
ICOS	Vopratelimab (JTX-2011)	Jounce Therapeutics	Agonistic mAb	II	II	NSCLC	Anti-PD-1 (Pimivalimab, JTX-4014)	SELECT	NCT04549025	Active, not recruiting
	Alomfilimab (KY1044, SAR-445256)	Kymab, Sanofi	Agonistic mAb	I/II	I/II	Solid Tumors	Anti-PD-1 (Atezolizumab)		NCT03829501	Recruiting
CD40	SEA-CD40	Seagen, Merck Sharp & Dohme	Agonistic mAb	II	I	Solid Tumors	Anti-PD-1 (Pembrolizumab), chemotherapy	SGNS40-001	NCT02376699	Active, not recruiting
	Mitazalimab (Vanalimab, ADC-1013, JNJ-64457107)	Alligator Bioscience, Janssen	Agonistic mAb	I/II	I/II	PDAC	Chemotherapy	OPTIMIZE-1	NCT04888312	Enrolling by invitation
	Sotigalimab (APX005M)	Apexigen, Bristol-Myers Squibb	Agonistic mAb	II	I/II	PDAC	Anti-PD-1 (Nivolumab), anti-CSF1R (Cabiralizumab)	PRINCE, PICI0002	NCT03214250	Active, not recruiting
	Giloralimab (ABBV-927)	AbbVie	Agonistic mAb	II	II	Pancreatic Cancer	Anti-PD-1 (Budigalimab), chemotherapy		NCT04807972	Recruiting
	YH003	Eucure Biopharma	Agonistic mAb	II	II	Melanoma, PDAC	Anti-PD-1 (Toripalimab), chemotherapy		NCT05031494	Recruiting
	CDX-1140	Celldex Therapeutics	Agonistic mAb	II	II	Ovarian Cancer	Anti-PD-1 (Pembrolizumab), anti-VEGF (Bevacizumab), chemotherapy		NCT05231122	Not yet recruiting
OX40	Revdofilimab (ABBV-368)	Abbvie	Agonistic mAb	I	I	Solid Tumors	Anti-PD-1 (Budigalimab), anti-CTLA-4 (Ipilimumab)		NCT04196283	Active, not recruiting
	HFB301001	HiFiBiO Therapeutics	Agonistic mAb	I	I	Solid Tumors			NCT05229601	Recruiting
	BGB-A445	BeiGene	Agonistic mAb	I	I	Solid Tumors	Anti-PD-1 (Tislelizumab)		NCT04215978	Recruiting
4-1BB/CD137	ADG106	Adagene	Agonistic mAb	I/II	I/II	NSCLC	Anti-PD-1 (Nivolumab)	ADIVO Lung	NCT05236608	Recruiting
	LVGN6051	Lyvgen Biopharma	Agonistic mAb	I/II	I/II	Soft Tissue Sarcoma	TKI (Anlotinib)		NCT05301764	Not yet recruiting
	AGEN2373	Agenus	Agonistic mAb	I	I	Solid Tumors	Anti-CTLA-4 (AGEN1181)		NCT04121676	Recruiting
	ATOR1017	Alligator Bioscience	Agonistic mAb	I	I	Solid Tumors			NCT04144842	Recruiting
Anti-PD-1/PD-L1 and anti-CTLA-4 agents										
PD-1	Pembrolizumab	Merck	Antagonistic mAb	FDA approved	I	Solid tumors	–	KEYNOTE-001	NCT01295827	Completed
	Nivolumab	Bristol-Myers Squibb	Antagonistic mAb	FDA approved	III	NSCLC	–	CheckMate 017	NCT01642004	Completed
	Dostarlimab	GlaxoSmithKline	Antagonistic mAb	FDA approved	I	Solid tumors	–	GARNET	NCT02715284	Recruiting
	Cemiplimab	Regeneron	Antagonistic mAb	FDA approved	III	NSCLC	–	EMPOWER-Lung 1	NCT03088540	Active, not recruiting
	Toripalimab (JS001)	Shanghai Junshi Biosciences	Antagonistic mAb	III	III	NSCLC	Chemotherapy		NCT04158440	Recruiting
	Sintilimab (IBI308)	Eli Lilly /Innovent Biologics	Antagonistic mAb	III	III	HCC	Anti-VEGF (Bevacizumab)	DaDaLi	NCT04682210	Not yet recruiting
	Tislelizumab (BGB-A317)	BeiGene	Antagonistic mAb	IV	IV	NMIBC	Cancer vaccine (BCG)		NCT05580354	Not yet recruiting
	Camrelizumab (SHR-1210)	Jiangsu Hengrui	Antagonistic mAb	III	III	NSCLC	TKI (Famitinib)		NCT05042375	Recruiting
	Pucotenlimab (HX008)	Taizhou Hanzhong Pharmaceuticals	Antagonistic mAb	III	III	GC	Chemotherapy		NCT04486651	Recruiting
	Serplulimab (HLX10)	Shanghai Henlius	Antagonistic mAb	III	III	NSCLC	Anti-VEGF (HLX04), chemotherapy		NCT03952403	Recruiting
	Budigalimab (ABBV-181)	AbbVie	Antagonistic mAb	II	II	Pancreatic Cancer	Anti-CD40 (ABBV-927), chemotherapy		NCT04807972	Recruiting
	Retifanlimab (INCMGA00012)	Incyte Corporation/Macrogenics	Antagonistic mAb	III	III	NSCLC	Chemotherapy	POD1UM-304	NCT04205812	Recruiting
	Ezabenlimab (BI 754091)	Boehringer Ingelheim	Antagonistic mAb	II	II	Solid Tumors	VEGF/ANG2 inhibitor (BI 836880), anti-LAG-3 (BI 754111)		NCT03697304	Active, not recruiting
	Penpulimab (AK105)	Akeso Biopharma/Chia Tai Tianqing	Antagonistic mAb	IV	IV	NSCLC	TKI (Anlotinib)	pcwaintrl	NCT05387109	Not yet recruiting
	Spartalizumab (PDR001)	Novartis	Antagonistic mAb	III	III	Melanoma	Braf inhibitor (Dabrafenib), MAP1/2 inhibitor (Trametinib)	COMBI-i	NCT02967692	Active, not recruiting
	Cetrelimab (JNJ-63723283)	Janssen	Antagonistic mAb	III	III	MIBC	Chemotherapy	SunRISe-2	NCT04658862	Recruiting
	Balstilimab (AGEN2034)	Agenus	Antagonistic mAb	II	II	CRC	Anti-CTLA-4 (Botensilimab)	NEST-1	NCT05571293	Not yet recruiting
	Zimberelimab (GLS-010)	Arcus/Guangzhou Gloria/Taiho Pharmaceutical	Antagonistic mAb	III	III	NSCLC	Anti-TIGIT (Domvanalimab), chemotherapy	ARC-10	NCT04736173	Recruiting
	Geptanolimab (Genolimzumab, APL 501)	Apollomics	Antagonistic mAb	II	II	Cervical Cancer	–		NCT03808857	Recruiting
	Prolgolimab (BCD-100)	Biocad	Antagonistic mAb	III	III	Cervical Cancer	Anti-EGFR (Bevacizumab), chemotherapy	FERMATA	NCT03912415	Recruiting
	Sasanlimab (PF-06801591)	Pfizer	Antagonistic mAb	III	III	NMIBC	Cancer vaccine (BCG)	CREST	NCT04165317	Recruiting
	Cosibelimab	Checkpoint Therapeutics/TG Therapeutics	Antagonistic mAb	III	III	NSCLC	Chemotherapy	CONTERNO	NCT04786964	Active, not recruiting
	Pimivalimab (JTX-4014)	Celgene, Jounce Therapeutics	Antagonistic mAb	II	II	NSCLC	ICOS agonist (Vopratelimab)	SELECT	NCT04549025	Active, not recruiting
	MEDI0680 (AMP514)	Amplimmune; AstraZeneca; MedImmune	Antagonistic mAb	I/II	I/II	Malignancies	Anti-PD-L1 (Durvalumab)		NCT02118337	Completed
	Nofazinlimab (CS1003)	CStone Pharmaceuticals	Antagonistic mAb	III	III	HCC	TKI (Lenvatinib)		NCT04194775	Recruiting
PD-L1	Atezolizumab	Roche, Genentech	Antagonistic mAb	FDA approved	III	NSCLC	–	OAK	NCT02008227	Completed
	Durvalumab	Celgene, MedImmune	Antagonistic mAb	FDA approved	III	NSCLC	–	PACIFIC	NCT02125461	Active, not recruiting
	Avelumab	Merck	Antagonistic mAb	FDA approved	II	MCC	–	JAVELIN Merkel 200	NCT02155647	Active, not recruiting
	Pacmilimab (CX-072)	CytomX Therapeutics	Probody	II	II	Breast Cancer	ADC (CX-2009)		NCT04596150	Active, not recruiting
	Sugemalimab (CS1001)	CStone Pharmaceuticals, Bayer	Antagonistic mAb	III	III	NSCLC	–	GEMSTONE-301	NCT03728556	Active, not recruiting
	Opucolimab (HLX-20)	Henlix Biotech	Antagonistic mAb	I	I	Solid Tumors	–		NCT03588650	Completed
	Envafolimab (KN035)	Alphamab	Antagonistic mAb	III	III	Biliary Tract Cancer	chemotherapy	KN035-BTC	NCT03478488	Recruiting
	Adebrelimab (SHR-1316)	Atridia; Jiangsu Hengrui	Antagonistic mAb	III	III	NSCLC	chemotherapy		NCT04316364	Recruiting
	INCB86550	Incyte Corporation	Small molecule inhibitor	II	II	Solid Tumors	–		NCT04629339	Active, not recruiting
	MAX-10181	Maxinovel Pharmaceuticals	Small molecule inhibitor	I	I	Solid Tumors	–		NCT05196360	Recruiting
CTLA-4	Ipilimumab	Bristol-Myers Squibb	Antagonistic mAb	FDA approved	III	NSCLC	Anti-PD-1 (Nivolumab), chemotherapy	CHECKMATE-227	NCT02477826	Active, not recruiting
	Tremelimumab	Pfizer, AstraZeneca	Antagonistic mAb	FDA approved	III	HCC	Anti-PD-L1 (Durvalumab)	HIMALAYA	NCT03298451	Recruiting
	BMS-986249	Bristol-Myers Squibb	Probody	I/II	I/II	Solid Tumors	Anti-PD-1 (Nivolumab)		NCT03369223	Recruiting
	Botensilimab (AGEN-1811)	Agenus	Antagonistic mAb	II	II	CRC	Anti-PD-1 (Balstilimab)	NEST-1	NCT05571293	Not yet recruiting
	Zalifrelimab (AGEN1884)	Agenus	Antagonistic mAb	II	II	Cervical Cancer	Anti-PD-1 (Balstilimab)		NCT05033132	Recruiting
	Quavonlimab (MK-1308)	Merck Sharp & Dohme	Antagonistic mAb	III	III	RCC	TKI (Lenvatinib), anti-PD-1 (Pembrolizumab)		NCT04736706	Recruiting
	Porustobart (HBM-4003)	Harbour BioMed	Antagonistic mAb	I	I	Solid Tumors	Anti-PD-1 (Triprilimab)		NCT04727164	Not yet recruiting
	YH-001	Eucure Biopharma	Antagonistic mAb	II	II	HCC, NSCLC	Anti-PD-1 (Toripalimab)		NCT05212922	Not yet recruiting
	ADG-116	Adagene	Antagonistic mAb	I	I	Solid Tumors	Anti-PD1 mAb, CD137 agonist (ADG106)		NCT04501276	Recruiting
	ONC-392	OncoImmune	Antagonistic mAb	II	II	Ovarian Cancer, Primary Peritoneal Carcinoma, Fallopian Tube Cancer	Anti-PD-1 (Pembrolizumab)	PRESERVE-004	NCT05446298	Not yet recruiting

The data are up to December 2022. For each agent, only one representative clinical trial is listed. All ongoing clinical trials for each agent are listed in Supplementary Tables 1–3. The therapeutic combination described in the representative trial is a simplified summary. For each drug, the cases of all kinds of combinations of the drug and other agents in one or several cohorts in a multi-cohort study, or the combination of the drug and other agents in a mono-cohort study are not separately described

*mAb* monoclonal antibody, *FDA* Food and Drug Administration, *NSCLC* non-small cell lung cancer, *HCC* hepatocellular carcinoma, *NMIBC* non-muscle-invasive bladder cancer, *BCG* bacillus Calmette-Guérin, *GC* gastric cancer, *CRC* colorectal cancer, *MCC* Merkel cell carcinoma, *RCC* renal cell carcinoma, *SCLC* small-cell lung cancer, *HNSCC* head and neck squamous cell carcinoma, *AML* acute myeloid leukemia, *MDS* myelodysplastic syndrome, *CMML* chronic myelomonocytic leukemia, *GEJC* gastroesophageal junction cancer, *DLBCL* diffuse large B cell lymphoma, *NHL* non-Hodgkin’s lymphoma, *BTC* biliary tract cancer, *ADC* antibody-drug conjugate, *PDAC* pancreatic ductal adenocarcinoma, *TKI* tyrosine kinase inhibitors

#### TIM-3: biology, drug development, and therapeutic efficacy

T cell immunoglobulin domain and mucin domain 3 (TIM-3, HAVCR2) is a membrane protein whose functions and signaling are not fully clear hitherto.^55^ TIM-3 is expressed on T cells, DCs, NK cells, and Tregs with distinct functions. TIM-3 can be expressed on activated CD4^+^ Th1 cells, mediating immune inhibition.^56–58^ On tumor-specific exhausted CD8^+^ T cells, expression of TIM-3 is upregulated.^59^ Galectin-9, carcinoembryonic antigen cell adhesion molecule 1 (CEACAM1), high mobility group box protein 1 (HMGB1), and phosphatidylserine have been identified as ligands of TIM-3 but none of them seems exclusive (Fig. 2). Galectin-9 and CEACAM1 suppress anti-tumor immunity by ligating TIM-3 to inhibit type 1 immunity.^60,61^ Expression of intracellular protein Bat3, an inhibitor of TIM-3, is reduced in TIM-3^+^CD4^+^ exhausted T cells.^62,63^ A current hypothesis is that Bat3 binds the cytoplasmic tail of TIM-3 and recruits tyrosine kinase Lck, impeding TIM-3 immunosuppression.^55^ However, co-stimulatory activity of TIM-3 is also purported, based on the finding that its transmembrane domain recruits it to the immune synapse, its cytoplasmic tail enhances TCR-signaling^64^ and its expression promotes the development of short-lived effector T cells^65^ and CD8^+^ T cell responses.^66^

On conventional DCs, TIM-3 mainly displays inhibitory functions. TIM-3 on tumor-infiltrating DCs sequesters nucleic acid-carrying protein HMGB1^67^ and thus can silence the immunogenicity of nucleic acids, resulting in reduced downstream cyclic GMP-AMP synthase (cGAS)-stimulator of interferon genes (STING) activation with reduced interferon-I, CXCR3, and CXCL9 production.^68,69^ In the CD8^+^ DC subset, TIM-3 recognizes phosphatidylserine and mediates phagocytosis of dying cell-associated antigens, which might silence tumor antigenicity^70^ (Fig. 2). Loss of TIM-3 activates NLR family pyrin domain containing 3 (NLRP3) inflammasome and subsequent interleukin (IL)-1β and IL-18 production, thus maintaining CD8^+^ effector and stem-like T cells.^71,72^ Moreover, TIM3^+^ Tregs induce stronger immunosuppression and express upregulated immunosuppressive markers.^73^

In human cancers, TIM-3 expression indicates an exhausted immune phenotype and correlates with poor outcome.^74–79^ Blockade of TIM-3 plus PD-1 showed synergy in preclinical models.^80,81^ Representative TIM-3 blocking antibodies cobolimab, sabatolimab, and LY3321367 showed good safety but limited efficacy in combination with anti-PD-1 antibody in phase I trials^82–84^ (Table 1 and Supplementary Table 1). However, in hematological malignancies, sabatolimab induced encouraging ORR of 61% and 47% in two different entities in combination with decitabine^85^ and received FDA fast track designation.^86^ A better understanding of TIM-3 biology and combination with other immunotherapeutic approaches may help overcome resistance and achieve durable responses.

#### TIGIT: biology, drug development, and therapeutic efficacy

The T cell immunoreceptor with Ig and ITIM domains (TIGIT) pathway is a complex immunoregulatory pathway due to the five different output receptors: TIGIT, CD96, CD112R (poliovirus receptor-related immunoglobulin domain-containing (PVRIG)), CD226 (DNAX accessory molecule-1 (DNAM-1)), and killer-cell Ig-like receptor 2DL5 (KIR2DL5), and four ligands: CD155 (poliovirus receptor (PVR)), CD112 (poliovirus receptor-related (PVRL)2, Nectin-2), CD111 (PVRL1, Nectin-1), and Nectin-4 (PVRL4) that have been identified so far (Fig. 2). Among the five receptors, TIGIT, CD96, CD112R, and KIR2DL5 mediate immunosuppression, while CD226 activates immunity. TIGIT interacts with CD155 and CD112 to inhibit activation and cytotoxicity of T and NK cells^87,88^ (Fig. 2). It is expressed on memory and effector CD8^+^ T cells and NK cells, and its expression is elevated in the tumor microenvironment (TME) and is associated with their exhaustion.^89,90^ TIGIT also characterizes highly suppressive regulatory B cells^91^ and Tregs.^92,93^ CD96 ligates CD155 but not CD112, resulting in inhibition of T and NK cell activity.^94–96^ Blockade of CD96 in animal models induced hyperresponsive NK and T cells with decreased tumor development and metastases.^95,97,98^ CD112R selectively binds CD112 and similarly suppresses CD8^+^ T and NK cells.^99–102^ KIR2DL5, a receptor on NK cells and T cells, specific for CD155,^103,104^ can be engaged by CD155 to inhibit cytotoxicity.^105^ Co-stimulatory CD226 competes with the four co-inhibitory receptors for binding to CD155 and CD112,^106^ and can promote graft-versus-host disease (GVHD).^107^ CD226 is also involved in lymphocyte function-associated antigen 1 (LFA-1)-mediated co-stimulatory signaling.^108,109^ CD226-CD155 interaction also plays a role in regulating NK cell-mediated cytotoxicity toward cancer cell.^110,111^

A remarkable feature of this pathway is the affinity disparity between the ligand-receptor interactions (Fig. 2). As reported,^96,112^ CD155 has the highest affinity to TIGIT and lower affinity to CD96 and CD226. CD112 binds TIGIT and CD226 less strong than CD155, and does not bind CD96.^96,112^ CD111 only interacts with and stabilizes CD155.^113^ Nectin-4 only interacts with TIGIT.^114^ These preferences bring about competitive binding dynamics, explaining the mechanism of immunosuppression mediated by this network-like pathway in cancers. Due to this, TIGIT and CD96 compete with CD226 to bind CD155/CD112 dominantly,^95,96,115^ and TIGIT can disturb the dimerization of CD226 for CD226 activation *in cis*.^89^ Other mechanisms include the upregulation of the transcription factor eomesodermin in T cells of the TME which inhibits CD226 expression, making TILs non-responsive to anti-PD-1 therapy,^109^ and PD-1-mediated direct inhibition of phosphorylation of CD226 and CD28.^116^ These effects on CD226 disrupt its stimulatory function. Taken together, TIGIT blockade abrogates the inhibitory effect by TIGIT and CD96 and is CD226-dependent, explaining anti-TIGIT and anti-PD-1 synergy.^90,116^

Expression of CD155 and CD112 is elevated in some human cancers,^100,117–124^ and TIGIT and CD96 are upregulated on T and NK cells in a series of malignancies, which is associated with poor prognosis and poor response to anti-PD-1 therapy, whereas benefit is observed with TIGIT and/or CD96 blockade.^117,118,125–131^ Anti-TIGIT mAb is the major agent type targeting this pathway, with fewer anti-CD96, anti-CD112R, and anti-CD155 mAbs (and recombinant poliovirus agent for CD155) available (Table 1 and Supplementary Table 1). Nectin-4 is overexpressed in many cancers and is mainly investigated as antibody-drug conjugate (ADC) target, i.e., as a tumor-associated antigen (TAA). Anti-TIGIT mAbs evaluated in phase III trials include vibostolimab, tiragolumab, ociperlimab, and domvanalimab (Table 1 and Supplementary Table 1). Anti-TIGIT mAbs are generally combined with anti-PD-1 mAb. Data of the phase II CITYSCAPE trial showed an ORR of 69.0% in the PD-L1 tumor proportion score (TPS) ≥ 50% group and 38.8% in the intention-to-treat group using tiragolumab and atezolizumab. The mPFS and median OS (mOS) of combination therapy also nearly doubled in the intention-to-treat group with quadrupled mPFS in the PD-L1 TPS ≥ 50% group (16.6 vs. 4.1 months).^132^ However, the phase III SKYSCRAPER-01 and SKYSCRAPER-02^133^ trials combining tiragolumab and atezolizumab did not meet their PFS endpoint compared with atezolizumab, although the OS endpoint is immature. Other phase III studies of tiragolumab are currently ongoing (Table 1 and Supplementary Table 1). A phase I study of vibostolimab showed an ORR of 26% in anti-PD-(L)1-naïve NSCLC patients with pembrolizumab.^134^ Other phase II and III studies of vibostolimab are ongoing.

### Myeloid checkpoint: CD47

CD47 (integrin associated protein (IAP), MER6, OA3) is expressed on normal tissue cells, cancer cells, and immune cells.^135,136^ It primarily exerts innate immune inhibitory effects through the signal-regulatory protein (SIRP) family proteins, especially SIRPα and SIRPγ expressed on myeloid cells^137–141^ to inhibit phagocytosis signals (Fig. 2). Compared to SIRPα, SIRPγ has much lower affinity for CD47,^142^ rendering SIRPα the main study focus. SIRPα has three Ig-like domains, a transmembrane domain, and a cytoplasmic tail carrying an ITIM and an immunoreceptor tyrosine-based switching motif (ITSM).^143–145^ It is predominantly expressed on myeloid cells, including macrophages, DCs, mast cells, and neutrophils.^140,141,146,147^ Similar to other inhibitory receptors, upon binding of CD47, activated ITIM and ITSM in SIRPα and the downstream signaling cascade mediated by SHP-1/2, Csk, and Grb-2 contribute to the weakened phagocytic effects.^144,145,148–150^ CD47 also interacts with pro-phagocytic SLAMF7 *in cis* to inhibit phagocytosis triggered by SLAMF7^151^ as well as integrins and thrombospondin-1 in the extracellular matrix to activate integrin signaling and platelet activation.^152–154^

Under physiological conditions, CD47 participates in various biological processes and reduces excessive destruction of cells and cellular components, including red blood cells (RBCs),^152–154^ platelets,^155^ and neuronal synapses.^156–158^ Under pathological conditions, phagocytosis is abnormally attenuated through the CD47-SIRPα axis and mediates retention of pathological RBCs,^159,160^ macrophage dysfunction,^161,162^ and abnormal proliferation of brain tissue.^163^ Regarding anti-tumor immunity, CD47 is expressed in various hematological and solid tumors,^164–167^ promoting tumor survival by evading the phagocytic activity of innate immune cells, laying the foundation for blocking the CD47-SIRPα axis to enhance tumor cell killing by phagocytosis. When the CD47-SIRPα axis is nonfunctional, macrophage clusters^168^ and IgA-mediated anti-tumoral neutrophils^168^ can generate potent anti-tumor responses. This axis also interferes with adaptive immunity. T cell responses are regulated by this axis indirectly via myeloid cells^136^ and directly through the CD47 and SIRPα expression on T cells.^136,169,170^ CD47 expressed on CD8^+^ T cells promotes their adhesion to cancer cells and sensitizes melanoma to ICIs when binding to SIRPα on cancer cells,^171^ and it also shields CD8^+^ T cells from necroptosis when interacting with conventional DCs, promoting the survival and functions of CD8^+^ T cells.^172^ These contrasting roles of the CD47-SIRPα axis in anti-tumor immunity need further study. Nevertheless, CD47-SIRPα axis blockade has shown anti-cancer effects and synergy with other anti-cancer^136,139–141^ therapies. However, since CD47 protects RBCs and platelets from destruction by myeloid cells, inhibiting this pathway may lead to adverse effects such as anemia^173–176^ and thrombocytopenia,^177,178^ which requires patients to receive a preceding low dose priming in the clinic.^179,180^ The Fc-FcγR interaction required for the anti-tumor activity of anti-CD47 mAbs is another contributor to these off-tumor adverse effects.^181^ Thus, the balance between effect and toxicity is crucial in CD47 drug development. Current pharmaceutical development focuses on structural modifications to reduce RBC toxicities as well as on providing additional pro-phagocytic signals to trigger the optimal anti-tumor effects of macrophages.^182^

The primary class of early CD47-SIRPα pathway-targeting drugs have been mAbs. Magrolimab, the most advanced anti-CD47 antibody, resulted in a high response rate in hematological tumors (complete response (CR): 53% in untreated acute myeloid leukemia (AML)/myelodysplastic syndrome (MDS), 10% in relapsed/refractory AML/MDS^179^). Though the phase III ENHANCE study for high-risk MDS has been terminated due to insufficient efficacy, results are still expected from other phase III trials in both hematological and solid tumors (Table 1 and Supplementary Table 1). Next generation anti-CD47 antibodies with reduced binding to RBCs due to cell type-specific glycosylation modification have been developed^183–188^ (Table 1 and Supplementary Table 1), some showing enhanced safety and efficacy in clinical trials.^174,189–191^ Lemzoparlimab, an anti-CD47 IgG4 antibody, enables a unique RBC-sparing property while retaining strong anti-tumor activity.^188^ Due to its promising early phase results,^192–194^ it is now being evaluated in MDS patients with azacitidine in a phase III clinical trial (NCT05709093). Ligufalimab similarly did not associate with hematological adverse effects and does not require a priming dose to prevent anemia.^189^ CD47-blocking fusion proteins with reduced binding to RBC and/or additional pro-phagocytic signal are developed. Although IgG1 possesses the best ability to induce phagocytosis by macrophages, IgG4 has been the most-chosen partner for fusion protein development to avoid severe RBC toxicity at the expense of some anti-tumor activity. Notably, ontorpacept exhibits only weak binding to RBCs, thus allowing the use of IgG1 to induce stronger phagocytosis.^195^ It is undergoing phase II evaluation for diffuse large B cell lymphoma and leiomyosarcoma (Table 1 and Supplementary Table 1), and preliminary results are promising.^196,197^

Combinatorial therapy has become another mainstream strategy. The current focus is on the use of azacitidine with or without venetoclax in hematological tumors.^175,198,199^ The use of chemotherapy increases the overall “eat me” signal of tumors, which synergizes with blockade of the “don’t eat me” signal and leads to enhanced phagocytic effects. Both the doublet^198^ and triplet^175^ combination resulted in promising CR rates in AML patients (doublet: over 30% in newly diagnosed patients; triplet: over 40% in newly diagnosed patients, over 10% in relapsed/refractory patients). In solid tumors, combinations with PD-(L)1 inhibitors and standard chemotherapy and radiotherapy receives extensive interest (Table 1 and Supplementary Table 1). Though efficacy results of phase I/II trials are mixed,^173,200–206^ most studies reported a feasible safety profile and preliminary signs of action, promoting further investigation. In previously treated small cell lung cancer patients, combined use of chemotherapy and nibrozetone, a first-in-class small molecule MYC and CD47 downregulator, resulted in 1/26 CR and 6/26 partial response.^203^ Ligufalimab, cadonilimab, and chemotherapy resulted in an ORR of 75% and a disease control rate (DCR) of 100% in 8 gastric or gastroesophageal junction cancer (GC/GEJC) patients.^205^

In general, though the CD47-SIRPα axis receives immense interest regarding biological exploration and shows promising results in early clinical trials, there are still gaps to be filled in our knowledge about its immunomodulatory mechanisms, and its pharmacological development is in an early stage with ongoing phase I and II clinical trials. Further validation is still required, and there will be more novel agents applying innovative drug delivery methods^207–213^ or engineered protein forms and antibody format^214^ entering clinical trials in the near future.

### B7 family proteins

The B7 family includes ten transmembrane glycoproteins identified so far: B7-1 (CD80), B7-2 (CD86), B7-H1 (PD-L1, CD274), B7-DC (PD-L2, CD273), B7-H2 (ICOSL, CD275, B7h), B7-H3 (CD276), B7-H4 (VTCN1), B7-H5 (VISTA), B7-H6 (NCR3LG1), and B7-H7 (HHLA2). PD-L1, PD-L2, CD80, and CD86 have been thoroughly investigated, and B7-H2 and B7-H6 are recognized as co-stimulatory, hence they are not discussed here. B7-H3, B7-H4, and B7-H7 are immunoreceptor ligands expressed on APCs or cancer cells, while B7-H5 simultaneously acts as a ligand or receptor (Fig. 2). In the following, we focus on B7-H3, B7-H4, and B7-H5, whose drug development has reached the stage of clinical trials.

#### B7-H3

B7-H3 (CD276, B7RP-2) is expressed on non-hematopoietic cells and APCs. It can also be induced on T cells, NK cells, and many types of cancer cells.^215–217^ B7-H3 inhibits T cell immunity, especially Th1 immunity by acting directly on T cells^218,219^ or indirectly on DCs.^220^ However, a co-stimulatory receptor, triggering receptor expressed on myeloid cells (TREM)-like transcript 2 (TLT-2, TREML2) expressed on CD8^+^ T cells constitutively and on activated CD4^+^ T cells, has been identified as a receptor of B7-H3, and their ligation promotes T cell immunity^221^ (Fig. 2). Another study claimed that B7-H3 on cancer cells reduced Tregs in the TME, enhancing anti-tumor immunity.^222^ But still, more studies consider B7-H3 as immunosuppressive in cancer. Recently, IL20RA has been identified as one of the receptors of B7-H3,^104,223^ and its expression is found predominantly on epithelial cells and carcinomas,^224^ suggesting cancer cell-cancer cell B7-H3-IL20RA interaction *in cis* or in trans. IL20RA upregulates PD-L1 expression by the JAK1-STAT3-SOX2 cascade,^225^ and B7-H3 maintains STAT3 levels to express CCL2, polarizing macrophages in the TME to the M2 phenotype.^226^

B7-H3 is expressed in a series of cancers, and higher expression is associated with worse prognosis.^227–236^ Moreover, B7-H3 is co-expressed with other immunosuppressive molecules such as PD-L1, B7-H4, and IDO1 on cancer cells.^237^ B7-H3 is also upregulated on APCs in the TME, suppressing T cell immunity.^238^ Anti-B7-H3 mAb induced CD8^+^ T and/or NK cell dependent anti-tumor immunity.^227,239,240^ However, due to the yet elusive immunobiology of B7-H3, the therapeutic approach using it as a TAA to develop CAR-T cells, ADCs, or bsAbs is more common. B7-H3 ADCs showed favorable efficacy preclinically^241,242^ and have entered clinical trials, for example MGC018, mirzotamab clezutoclax, and DS-7300a (Table 1 and Supplementary Table 1). Enoblituzumab is an Fc-enhanced anti-B7-H3 mAb inducing antibody-dependent cellular cytotoxicity (ADCC)-mediated anti-tumor activity;^243^ phase I studies and a phase II prostate cancer study (NCT02923180) demonstrated favorable safety and efficacy. However, another phase II study of enoblituzumab with anti-PD-1 mAb or PD-1×LAG-3 bsAb in HNSCC has been closed due to safety concerns (NCT04634825). B7-H3 targeting agents may be mainly developed as ADCs and msAbs in the future. The anti-tumor activity of B7-H3 mAb caused by interference with B7-H3 ligand-receptor interaction should be further clarified.

#### B7-H4

B7-H4 (V-Set Domain Containing T Cell Activation Inhibitor 1 (VTCN1), B7x, B7S1) is expressed on hematopoietic cells and especially on myeloid APCs. B7-H4 ligation of the not yet identified putative receptor on T cells mediates profound inhibitory effects on T cell immunity^244^ (Fig. 2). B7-H4 limits Th1 and Th17-mediated autoimmunity^245^ and neutrophil-dependent innate immunity.^246^ Inhibition of B7-H4 can partially restore CD28 or inducible T-cell costimulator (ICOS) deficiency-mediated inhibition of T cell proliferation and functions.^247^

Expression of B7-H4 is upregulated in several cancers and is related to worse prognosis.^232,237,248–251^ Its expression is also complementary to PD-L1 expression in lung cancer.^252,253^ B7-H4 is expressed on immunosuppressive tumor-associated macrophages (TAMs) in the TME.^254^ Its expression is stimulated by STAT3 activated by IL-6 and IL-10 produced by TAMs in an autocrine manner, and this autocrine loop is induced by Tregs recruited by CCL22 secreted from TAMs.^255,256^ B7-H4 expressed on DCs in the TME interacts with its putative receptor on CD8^+^ T cells to induce T cell dysfunction.^257^ Combinatorial blockade of B7-H4 and PD-1 synergistically enhanced anti-tumor immunity in a preclinical study.^257^ However, two studies indicated a co-stimulatory role of B7-H4 in anti-tumor immunity^258^ and renal immunopathy.^259^ As B7-H4 is generally regarded as a co-inhibitory ligand, its precise function should be clarified by identifying its receptor.

In line with inhibitory properties of B7-H4, anti-B7-H4-blocking antibodies showed encouraging preclinical anti-tumor efficacy.^257,260,261^ Anti-B7-H4 mAb has entered clinical trials including first-in-class antibody alsevalimab and NC762 (Table 1 and Supplementary Table 1). Enrollment for phase Ib monotherapy and phase Ia combinatorial therapy for alsevalimab is ongoing.

#### B7-H5

B7-H5 (V-domain Ig suppressor of T cell activation (VISTA), PD-1H) contains one PD-L1-like extracellular IgV-like domain. Human B7-H5 lacks immunoreceptor tyrosine-based inhibitory motif (ITIM) but possesses three intracellular SH3 binding motifs, suggesting roles as both receptor and ligand, and bidirectional signaling. B7-H5 is primarily expressed on hematopoietic cells including myeloid APCs and T cells, and is predominantly expressed higher on the former ones.^262–264^ As a ligand, B7-H5 on APCs ligates VSIG3, P-selectin glycoprotein ligand 1 (PSGL-1), and less confirmed VSIG8 on T cells^264–266^ thus inhibiting T cell functions^262^ (Fig. 2). B7-H5 on T cells regulates naïve-T cell quiescence, suppresses CD4^+^ T cell immunity as a receptor,^267,268^ and is nonredundant with PD-1.^269^ Absence of functional B7-H5 exacerbates autoimmunity by impairing B7-H5-mediated quiescence of self-reactive naïve T cells.^268,270–272^ B7-H5 on T cells, neutrophils, and DCs can transmit inhibitory signals as a receptor, reducing their activation and functions.^270^ B7-H5 is upregulated on APCs and Tregs in the TME but not predominantly on cancer cells.^263,273–275^ It can also be upregulated on TAMs after activation of histamine receptors, resulting in downregulation of histamine-mediated allergy or tumor inflammation.^276,277^ Moreover, in the hypoxic TME, upregulated hypoxia-inducible factor-1α elevates B7-H5 expression on myeloid-derived suppressor cells (MDSCs).^278^ Meanwhile, this acidic TME promotes B7-H5-PSGL-1 binding,^264,266^ inducing enhanced immunosuppression.

As a potential therapy, B7-H5 blockade suppressed tumor growth by enhancing the infiltration, proliferation, and effector function of T cells, and reducing B7-H5^+^ MDSCs and Tregs.^273,278^ Anti-B7-H5 mAb HMBD-002 and onvatilimab, and small molecule inhibitor CA-170 have entered clinical trials (Table 1 and Supplementary Table 1). HMBD-002 reversed B7-H5-induced immunosuppression and inhibited tumor growth.^279^ A phase I study of HMBD-002 ± pembrolizumab is ongoing (NCT05082610). CA-170 increased CD8^+^ T cell infiltration, decreased infiltration of MDSCs and Tregs, and provoked almost complete suppression of lung cancer when combined with a peptide vaccine.^280^ CA-170 monotherapy induced clinical benefit rate (CBR) and mPFS of 75% and 19.5 weeks in immunotherapy-naïve NSCLC patients in a phase I study,^281^ and CBR of 68.18% in this population in a phase II study.^282^

### Structurally or functionally optimized anti-PD-(L)1 and anti-CTLA-4 agents

Meanwhile, a relatively large number of alternative ICIs targeting PD-1, PD-L1, and CTLA-4 have been developed. A major aim of current drug development is to overcome limitations of existing ICIs. New antibodies such as toripalimab, sintilimab, and spartalizumab are specifically designed to bind epitopes of PD-1 so far not targeted, reinforcing affinity and PD-1 saturation, and have shown considerable clinical efficacy.^283,284^ Besides, the binding to Fc-gamma receptors (FcγRs) is minimized in tislelizumab or eliminated in penpulimab, impairing antibody-dependent macrophage-mediated killing of T effector cells. Novel anti-CTLA-4 antibodies such as AGEN1181 are Fc-engineered to prompt Treg depletion.^285^ Further development utilizes innovative molecule structures. The unique design of the novel anti-PD-L1 antibody envafolimab fusing a single Fab domain to an ADCC/complement-dependent cytotoxicity (CDC)-silent Fc domain can improve tumor penetration and subcutaneous injectability.^286^ Probody technique-based anti-PD-L1 pacmilimab is proteolytically conditionally activated in tumor tissue, and may thus reduce off-target toxicity. In summary, improvement strategies for new anti-PD-(L)1 and anti-CTLA-4 antibodies include (1) binding previously not yet targeted epitopes of PD-1 (e.g., toripalimab, sintilimab, and spartalizumab), (2) Fc engineering, either abating/eliminating or enhancing binding of the antibody Fc segment to Fc receptors, and (3) adapting new structures (e.g., envafolimab and pacmilimab). In the second strategy, the Fc segment can be silenced to avoid disturbance from FcγRs (e.g., tislelizumab, penpulimab, and prolgolimab). Alternatively, binding of FcγRs by anti-CTLA-4 antibodies can be enhanced, facilitating efficient Treg depletion (e.g., botensilimab and porustobart). Another approach involves enhancing binding to the neonatal Fc receptor, thereby extending half-life of the antibody (e.g., pucotenlimab).^287^ Approaches are further diversified by introducing RNA interference and small molecule inhibitors, not only aiming at blocking receptor/ligand interaction but instead kinases or other pathways regulating immune checkpoint activities, resulting in very diverse approaches of anti-PD-1/PD-L1 and anti-CTLA-4 agent development. At present, more than 30 anti-PD-1/PD-L1 and more than 10 anti-CTLA-4 agents so far without FDA approval are under clinical investigation (Table 1 and Supplementary Table 2).

## Co-stimulatory molecules of T cells

T-cell activity is not only regulated by inhibitory checkpoints but also by positive co-stimulatory molecules. To initiate anti-cancer immunity, activation signals from CD28 and other positive co-stimulatory molecules are needed for naïve-T cell priming. The use of ICIs, e.g., of PD-(L)1 blockers, does not appear promising in the case of insufficient T cell priming, as in “cold” tumors and non-responsive patients. For successful priming, T cells need additional signals from molecules including IgSF member ICOS and tumor necrosis factor (TNF) receptor (TNFR) superfamily (TNFRSF) members CD40, GITR, OX40, 4-1BB, and others for further activation, proliferation, and differentiation (Fig. 2). After the first two activation signals from the TCR/CD3 complex-MHC molecule interaction and CD28-CD80/CD86 interaction, TNFRSF member CD40 on APCs interacts with its ligand CD40L on T cells.^288,289^ This elicits further signals driving T cell activation and DC maturation and reciprocally enhances CD28 and CD80/CD86 expression, resulting in a feedforward cycle.^288^ Thereafter, additional TNFRSF co-stimulatory molecules preserve T cell function by their ligation and downstream signaling. Besides CD40, these molecules include OX40, 4-1BB, GITR, TNFR1/2, CD27, and others. B7-H2/ICOSL and B7-H6 are regarded as ligands of ICOS and NKp30, respectively. Our discussion focuses on ICOSL and ICOS as the physiology of B7-H6 is not well known yet. For TNFRSF members, our discussion focuses on CD40, OX40, and 4-1BB.

### Targeting co-stimulatory molecules with agonistic antibodies: mechanism of action and characteristics

The main strategy for utilizing these molecules in cancer immunotherapy is developing agonistic antibodies or agonists. Different from ICIs blocking receptor/ligand interactions and TAA mAbs inducing ADCC/CDC, co-stimulatory agonists are meant to stabilize bridging and immune synapses formed by co-stimulatory ligand-receptor interaction between APCs and T cells, stabilize receptor oligomerization and superclustering to mediate strong activation^290,291^ (Fig. 2). Therefore, the efficiency of agonists is affected by unique factors. First of all, agonists with very high affinity or at excessive dose can lose their agonistic function,^292^ suggesting a bell-shaped affinity-agonism and dose-response relationship and an optimal affinity and dose. Secondly, agonistic antibodies can bind both natural ligand binding sites and exclusive epitopes.^290,293,294^ For example, different domains of CD40 are associated with agonistic or antagonistic effects of anti-CD40 antibodies.^295^ Characterizing the antibody binding epitope is therefore very important for agonist development.

Moreover, the interaction between the antibody Fc domain and FcγRs can induce both agonist and ADCC/CDC effects. Except for the inhibitory FcγRIIB, other FcγRs are activating and FcγRI has the highest affinity for the Fc region. Binding FcγRIIB is proposed to promote target receptor crosslinking and to maintain immune synapses, thus providing true agonism^290,291^ (Fig. 2). Instead, binding activating FcγRs can elicit ADCC, which can be utilized to deplete Tregs, especially using the IgG1 isotype with the strongest binding to activating FcγRs^290,291^ (Fig. 2). Therefore, agonists can either activate anti-cancer immune cells or deplete immunosuppressive populations. However, issues might arise from indiscriminate ADCC triggered by activating FcγRs, depleting Tregs but also effector cells. Binding activating FcγRs also contributes to toxic side effects, e.g., in case of 4-1BB agonists.^296,297^ For these reasons, Fc engineering is crucial and has been shown to be highly useful for the development of pure agonists by removing the Fc segment,^297,298^ mutation methods abating Fc-FcγR interactions^299^ or selectively enhancing Fc-FcγRIIB binding.^300^

In particular, human IgG2 agonists can activate co-stimulatory molecules including CD40, 4-1BB, and CD28 independent of FcγRs.^290,301^ Later studies showed that agonists with rigid conformation constrained by “tight” hinge region promote clustering of co-stimulatory molecules^301–303^ and tend not to bind excess epitopes mediating antagonism as is the case for more flexible antibodies,^301,304^ thus providing sufficient agonism even without FcγRs,^295^ and this phenomenon exists on natural IgG2 isotype mAb.^301,302^

### IgSF co-stimulatory receptor: ICOS

ICOS (CD278) is the receptor of ICOSL (B7-H2, CD275, B7h). Upon initial activation of TCR and CD28 signaling, ICOS is upregulated on T cells and this can non-redundantly enhance T cell immunity^288,290,291^ while ICOS is constitutively expressed on Tregs.^291^ ICOSL is constitutively expressed on APCs.^288^ After activation, ICOS induces phosphoinositide 3-kinase (PI3K)-Akt signaling,^305^ mammalian target of rapamycin (mTOR),^306^ and nuclear factor of activated T cells (NFAT)-responsive genes^290^ in T cells.

Anti-ICOS agonistic antibodies currently under development include vopratelimab and alomfilimab (Table 1 and Supplementary Table 3). The widely reported IgG4 pure agonist feladilimab has been removed from the GlaxoSmithKline pipeline due to its unsatisfactory clinical activity in phase II studies. The IgG1 mAbs vopratelimab and alomfilimab are designed to deplete intratumoral Tregs. Although vopratelimab plus nivolumab only elicited a total ORR of 2.3%, patients with ICOS^high^ CD4^+^ effector T cells had longer PFS and OS than patients without these cells (6.2 vs. 1.9 and 20.7 vs. 9.0, months).^306^ This finding guided the patient selection for the phase II SELECT study in NSCLC, where the combination of vopratelimab at 0.03 mg/kg with pimivalimab (a PD-1 inhibitor) yielded an ORR of 40% and a 6-month PFS rate of 80%. However, the study did not reach the primary endpoint of tumor shrinkage between vopratelimab plus pimivalimab and pimivalimab monotherapy groups.^307^ Alomfilimab depleted ICOS^high^ Tregs, had monotherapy anti-tumor efficacy, and improved anti-PD-L1 efficacy in a pre-clinical study.^308^ According to a preliminary report there were 5 OR cases out of 103 patients in a phase I/II trial testing alomfilimab ± atezolizumab.^309^ In summary, ICOS drug development is still challenging.

### TNFRSF co-stimulatory receptor: CD40, OX40, and 4-1BB

Upon ligand trimer ligation, TNFRs on T cells trimerize to recruit TNFR-associated factor (TRAF)1-6 in different preferences and activate distinct downstream adapters but predominantly converge at nuclear factor-κB (NF-κB) signaling.^289,290^ According to the chronological impact on T cell activation as discussed above, we first discuss CD40, then focus on OX40 and 4-1BB that aroused most incentives of industries. Unlike OX40 and 4-1BB, the development of GITR agonists has been largely terminated due to limited responses.^310–315^ Similarly, agents targeting the CD27-CD70 pathway, such as the widely reported CD27 agonist varlilumab and CD70 agonist cusatuzumab, have also been removed from the pipelines of Celldex and Argenx, respectively, due to unfavorable developmental prospects. Likewise, the development of TNFR1/2 agonists remains immature, with almost all agents still under preclinical investigation.^316,317^ Therefore, other TNFRSF receptors, including GITR, CD27/CD70, and TNFR1/2, are not the focus of our discussion.

#### CD40

CD40 (TNFRSF5) expressed mainly by APCs plays an important role in initial activation of CD4^+^ T cells following the CD28 signal. CD40L (CD154) mainly expressed by CD4^+^ T cells ligates and activates CD40, triggering the maturation of DCs which is crucial for the efficient priming of T cells including CD4^+^ Th cells and cross-primed CD8^+^ T cells^288,318^ (Fig. 2). Activated CD40 stimulates expression of CD80 and CD86 on DCs thus stimulating the CD28 coreceptor on T cells which in turn leads to upregulation of CD40L on T cells coordinately driving T cell stimulation and DC maturation (Fig. 2).

The agents presently developed all entered phase II clinical trials (Table 1 and Supplementary Table 3), while only the development of selicrelumab has been discontinued. SEA-CD40, mitazalimab, sotigalimab, and giloralimab are IgG1 FcγR-dependent DC activators, whereas YH003 and CDX-1140 are IgG2 pure agonists. In the phase Ib/II PRINCE study of sotigalimab plus chemotherapy ± nivolumab in pancreatic adenocarcinoma, the total ORR was 58% in the phase Ib part,^319^ while in phase II part, the confirmed ORR of sotigalimab plus chemotherapy was 33%.^319^ Mitazalimab efficiently upregulated CD80/CD86 expression and IL-12 secretion by DCs, induced antigen-specific T cell proliferation and anti-tumor activity preclinically.^320,321^ Efficacy evaluation is ongoing in the phase II OPTIMIZE-1 study combining mitazalimab and chemotherapy in pancreatic ductal adenocarcinoma (PDAC) patients. More studies will be needed on combinations with other agents or regarding optimizing indication selection.

#### OX40

OX40 (CD134, TNFRSF4) is temporarily expressed by memory T cells and activated T cells following TCR/CD3 signaling and has important roles in their survival, yet it does not participate in T cell priming.^288,291^ It is also constitutively expressed by Tregs.^288,291^ Interestingly, OX40 agonism does not impair the immunosuppressive functions of Tregs but only confers them an inflammatory phenotype.^322^ Expression of OX40L (CD252) is upregulated on APCs after their activation and can be promoted by activated CD40.^288^ After binding of OX40L,^323^ trimerized OX40 recruits TRAF2-3 and TRAF5 to transmit canonical and non-canonical NF-κB and other signals^288,289^ (Fig. 2).

Several major companies have withdrawn from the development of OX40 agonists due to unfavorable clinical efficacies,^324–326^ indicating the necessity of strategy improvement for further development. OX40 agonists under development currently include revdofilimab, HFB301001, and BGB-A445 (Table 1 and Supplementary Table 3). The IgG1 agonist INCAGN1949 is proven to FcγR-dependently stimulate OX40 and deplete OX40^high^ Tregs.^327^ However, in a phase I/II study, INCAGN1949 monotherapy only elicited an ORR of 1.15%,^328^ hence it has been removed from the pipeline of Agenus. Trials of other agonists are all still ongoing. The development of many OX40 agonists has been discontinued. Due to the transient expression of OX40, the timing of OX40 agonist administration may be important.^291^ Further development of OX40 agonists may need either combining with other agents in an appropriate order or developing msAbs.

#### 4-1BB

4-1BB (CD137, TNFRSF9) is also transiently upregulated following TCR/CD3-mediated signaling mainly on activated T cells^289^ but is also detected on NK cells and APCs.^291^ Upon ligation of 4-1BBL (TNFSF9), 4-1BB recruits TRAF1-2 to activate downstream signaling similar to OX40^289^ (Fig. 2). Considering the substantial liver toxicity at doses of ≥1 mg/kg^290,293,294^ and modest ORR of 3.8%^329^ observed in trials of the first generation 4-1BB agonistic antibodies urelumab and utomilumab respectively, Bristol-Myers Squibb and Pfizer deprioritized the development of these two drugs. However, subsequent analyses have guided further design of 4-1BB agonists. As many reports indicated, utomilumab showed insufficient clinical monotherapy activity while urelumab induces strong agonism but also severe toxicity in a fraction of the patients.^291^ Structural analysis indicated that utomilumab blocks natural ligands and binds 4-1BB at proximal domains while urelumab binds the distal one,^330,331^ which is consistent with antibodies against CD40.^295^ This reflects the importance of the binding epitopes in the design of agonists. The toxicity of urelumab mostly stems from Fc-FcγR interaction, thus Fc engineering is relevant for toxicity management of 4-1BB agonists. Based on such considerations, next-generation 4-1BB agonists including ADG106,^332^ LVGN6051,^333^ AGEN2373,^334^ and ATOR1017 have been developed and are being investigated in clinical trials (Table 1 and Supplementary Table 3). In a phase I trial of ADG106, treatment appeared to be safe with a DCR of 57%.^332^ LVGN6051 monotherapy elicited a DCR of 70% and induced preliminary ORR of 25% combined with pembrolizumab in a phase I study.^335^ AGEN2373 induced a DCR of 26.3% without liver toxicity.^336^ Dose escalation for ATOR-1017 is still ongoing with the best response of SD observed.^337^

In summary, agonists targeting costimulatory receptors appear powerful candidates for future immunotherapy and a wave of new agonistic molecules has been developed many of which have entered clinical trials. However, agonist development is more difficult than the development of antagonists because more parameters have to be taken into account. Clinical trials have shown that agonist monotherapies scarcely induce favorable responses hence combination with ICIs or other agents may become particularly important. Next-generation constructs including Fc-engineered mAbs, multi-valent mAbs, and bsAbs/msAbs seem promising.

## Immunoregulatory bispecific and multi-specific antibodies

**Fig. 3:**
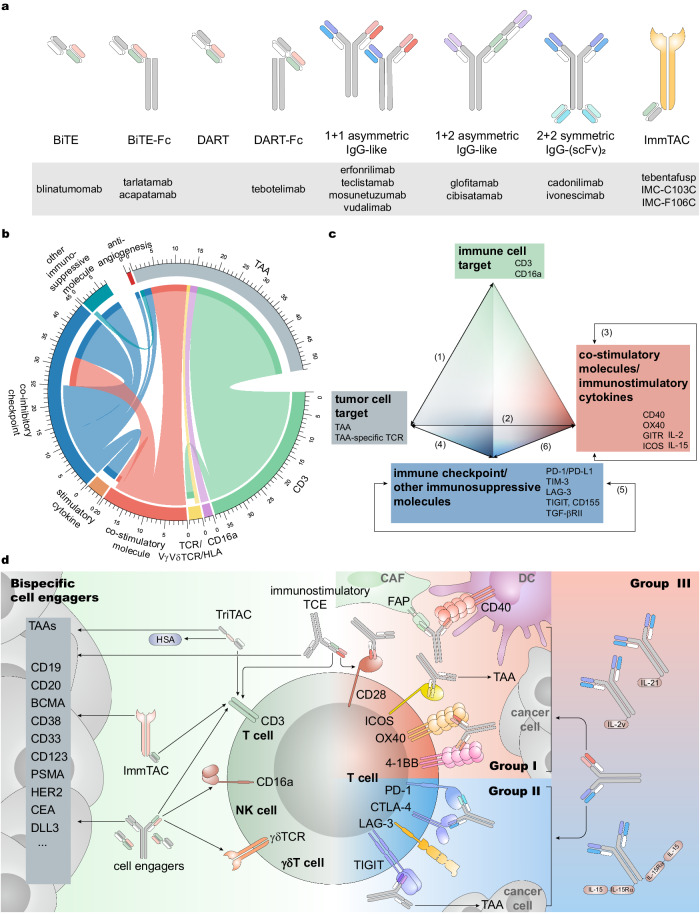
Various formats and categories of bsAbs. **a** Examples of common bsAb formats. Short bars indicate the antibody Fab segment, long bars indicate the Fc segment, and for ImmTAC, the Fab segment is linked to an antigen-specific TCR. In the same antibody icon, different color combinations of the Fab segment indicate the binding to different target proteins. BsAb examples of these formats are in the gray box. **b** According to our statistics of bsAbs that have entered clinical trials, the TCEs comprising the anti-CD3 scFv account for about half of all bsAbs under development at present. The development of other bsAb categories presents a diversified landscape. The two ends of each arc indicate two targets of bsAbs. Only bsAbs are counted for this figure, with msAbs with higher valency excluded. The data of this figure are consistent with Table 2 and Supplementary Table 4. The statistic is up to October 2022 and bsAbs with terminated development are excluded. **c** Prism of developmental strategies of bsAbs. The strategy of bsAb development is mainly to combine four types of targets: immune cell targets, tumor cell targets, co-stimulatory molecules/immunostimulatory cytokines, and immune checkpoints or other immunosuppressive molecules. By these designs, immune cells and immunomodulatory signals can be introduced into the TAA-expressing environment. The black lines on the edge of the prism indicate that the corresponding bsAb category targets the target types directed by the arrows: (1) TCEs and NKEs; group I general bsAbs: (2) co-stimulatory molecule × TAA or TME protein and (3) co-stimulatory molecule × co-stimulatory molecule; group II general bsAbs: (4) inhibitory checkpoint × TAA and (5) inhibitory checkpoint × inhibitory checkpoint; group III general bsAbs: (6) inhibitory checkpoint × co-stimulatory molecule. **d** Mechanism-of-action of bispecific cell engagers and group I-III general immunoregulatory anti-cancer bsAbs. DART dual-affinity retargeting, scFv single-chain variable fragment, TAA tumor-associated antigen, HLA human leukocyte antigen, HSA human serum albumin, TriTAC Tri-specific T cell activating construct, CAF cancer-associated fibroblast, FAP fibroblast activation protein

The concept of bsAb targeting two different molecules was proposed in the last century.^338^ At that time, shortly after gaining insights into immunoglobulin biology, Alfred Nisonoff envisioned combining two distinct antigen-binding sites within a single molecule. He connected rabbit Fab fragments with different specificities using chemical methods and demonstrated bispecificity of the resulting product.^339^ Subsequently, other researchers advanced the field of bsAbs by introducing hybridoma methods for mAbs, phage display techniques, and strategies to direct antibody effects towards various target cells.^340–342^ However, bsAbs/msAbs with promising efficacy and acceptable safety had not been developed until the last decade, when the CD3×CD19 bispecific T-cell engager (BiTE) blinatumomab was approved by the FDA.^342^ Along with the advances in antibody format design, and further comprehension of cancer immunology, anti-cancer bsAbs/msAbs targeting immunoregulatory and other cancer-related molecules are under intensive development. Here we present an update of the developmental landscape of these agents (Fig. 3a–d, Table 2 and Supplementary Table 4) compared with previous summaries^342–346^ according to data from the pipeline and clinical trials. We briefly introduce the characteristics of anti-cancer immunoregulatory bsAbs/msAbs, mainly discussing their categories according to mechanism-of-action, and clinical vista of widely reported agents.

**Table 2:** Immunoregulatory anti-cancer bispecific and multi-specific antibodies

Basic information						Representative trial					
Agent	Manufacturer	Components			Highest developmental phase	Phase	Cancer type	Therapeutic combination	Study	Identifier	Status
		Component 1	Component 2	Component 3							
T Cell Engagers											
		CD3 × TAA									
Blinatumomab (AMG-103, MEDI538)	Amgen	anti-CD3	anti-CD19		FDA approved	II	B-precursor ALL	–	BLAST	NCT01207388	Completed
Teclistamab (JNJ-64007957)	Janssen, Genmab	anti-CD3	anti-BCMA		FDA approved, EMA approved	I	MM	–	MajesTEC-1	NCT03145181	Recruiting
Mosunetuzumab (RG7828, RO7030816)	Genentech, Roche	anti-CD3	anti-CD20		FDA approved, EMA approved	I/II	FL, NHL, CLL	anti-PD-L1 (Atezolizumab)		NCT02500407	Recruiting
APVO436	Aptevo Therapeutics	anti-CD3	anti-CD123 (IL-3Rα)		I	I	AML, MDS	–		NCT03647800	Recruiting
GB261	Genor Biopharma	anti-CD3	anti-CD20		I/II	I/II	CLL, B-Cell NHL	–		NCT04923048	Recruiting
Epcoritamab (GEN3013)	AbbVie, Genmab	anti-CD3	anti-CD20		III	III	DLBCL	chemotherapy	EPCORE DLBCL-1	NCT04628494	Recruiting
Glofitamab (RG6026, RO 7082859)	Roche, Genentech, Chugai Pharmaceutical	anti-CD3	anti-CD20		III	III	DLBCL	anti-CD20 (Obinutuzumab), anti-IL-6 (Tocilizumab), chemotherapy		NCT04408638	Recruiting
Plamotamab (XmAb13676)	Xencor, Janssen, Novartis	anti-CD3	anti-CD20		II	II	DLBCL	anti-CD19 (Tafasitamab)		NCT05328102	Recruiting
Odronextamab (REGN-1979)	Regeneron	anti-CD3	anti-CD20		II	II	B-Cell NHL	–	ELM-2	NCT03888105	Recruiting
Runimotamab (RG6194, RO-7227780)	Genentech	anti-CD3	anti-HER2		I	I	Solid Tumors	anti-HER2 (Trastuzumab), anti-IL-6 (Tocilizumab)		NCT03448042	Recruiting
AMX 818	Amunix, Sanofi	anti-CD3	anti-HER2		I	I	HER2-Expressing Cancers	anti-PD-1 (Pembrolizumab)		NCT05356741	Recruiting
ISB-1342 (GBR 1342)	Glenmark Pharmaceuticals	anti-CD3	anti-CD38		I	I	MM	–		NCT03309111	Recruiting
Vixtimotamab (AMV-564)	Affimed Therapeutics	anti-CD3	anti-CD33 (Siglec-3)		I	I	AML	anti-PD-1 (Pembrolizumab)		NCT03144245	Completed
Cevostamab (BFCR-4350A, RG 6160, RO-7187797)	Genentech	anti-CD3	anti-FcRH5		I	I	MM	anti-IL-6 (Tocilizumab), anti-CD38 (Daratumumab), chemotherapy	CAMMA 1	NCT04910568	Recruiting
Elranatamab (PF-06863135)	Pfizer	anti-CD3	anti-BCMA		III	III	MM	chemotherapy	MagnetisMM-7	NCT05317416	Recruiting
Pavurutamab (AMG701)	Amgen	anti-CD3	anti-BCMA		I	I	MM	–	ProxiMMity-1	NCT04998747	Not yet recruiting
CM336	Keymed Biosciences	anti-CD3	anti-BCMA		I/II	I/II	MM	–		NCT05299424	Not yet recruiting
TNB-383B (ABBV-383)	TeneoBio, AbbVie, Amgen	anti-CD3	anti-BCMA		I	I	MM	–		NCT03933735	Recruiting
Ubamatamab (REGN4018)	Regeneron	anti-CD3	anti-MUC16		I/II	I/II	Ovarian Cancer	anti-PD-1 (Cemiplimab)		NCT03564340	Recruiting
AMG199	Amgen	anti-CD3	anti-MUC17		I	I	MUC17-positive Solid Tumors	–		NCT04117958	Recruiting
Tarlatamab (AMG757)	Amgen	anti-CD3	anti-DLL3		II	II	SCLC	–	DeLLphi-301	NCT05060016	Recruiting
BI 764532	Boehringer Ingelheim	anti-CD3	anti-DLL3		I	I	SCLC, Neuroendocrine Tumors	–		NCT04429087	Recruiting
Cibisatamab (RG7802, RO6958688)	Roche	anti-CD3	anti-CEA		I/II	I/II	NSCLC	anti-PD-1 (Atezolizumab), anti-IL-6 (Tocilizumab)	Morpheus Lung	NCT03337698	Recruiting
Acapatamab (AMG 160)	Amgen	anti-CD3	anti-PSMA		I/II	I/II	PC	anti-PD-1 (AMG404)		NCT04631601	Active, not recruiting
AMG 340 (TNB-585)	Amgen	anti-CD3	anti-PSMA		I	I	PC	–		NCT04740034	Recruiting
CCW702	Calibr, AbbVie	anti-CD3	anti-PSMA		I	I	PC	–		NCT04077021	Recruiting
AMG 509	Amgen, BeiGene, Xencor	anti-CD3	anti-STEAP1		I	I	PC	anti-PD-1 (Pembrolizumab), chemotherapy		NCT04221542	Recruiting
ERY974	Chugai Pharmaceutical	anti-CD3	anti-Glypican 3 (GPC3)		I	I	HCC	anti-PD-1 (Atezolizumab), anti-IL-6 (Tocilizumab), anti-VEGF (Bevacizumab)		NCT05022927	Recruiting
CM350	KeyMed Biosciences	anti-CD3	anti-Glypican 3 (GPC3)		I/II	I/II	Solid Tumors	–		NCT05263960	Recruiting
Talquetamab (JNJ-64407564)	Janssen, Gemnmab	anti-CD3	anti-GPRC5D		II	II	MM	–		NCT04634552	Recruiting
AMG 427	Amgen, BeiGene	anti-CD3	anti-FLT3		I	I	AML	–		NCT03541369	Recruiting
NVG-111	NovalGen	anti-CD3	anti-ROR1		I/II	I/II	CLL, Lymphoma	–		NCT04763083	Recruiting
IBI-389	Innovent Biologics	anti-CD3	anti-Claudin 18.2		I	I	Solid Tumors	anti-PD-1 (Sintilimab)		NCT05164458	Not yet recruiting
		CD3 × TAA × co-stimulatory molecule									
SAR442257	Sanofi	anti-CD3	anti-CD38	CD28 agonist	I	I	MM, NHL	–		NCT04401020	Recruiting
		CD3 × TAA × PK/PD improvement element									
HPN424	Harpoon Therapeutics	anti-CD3	anti-PSMA	human serum albumin	I/II	I/II	PC	–		NCT03577028	Recruiting
HPN536	Harpoon Therapeutics	anti-CD3	anti-mesothelin	human serum albumin	I/II	I/II	Solid Tumors With Mesothelin Expression	–		NCT03872206	Recruiting
HPN328	Harpoon Therapeutics	anti-CD3	anti-DLL3	human serum albumin	I/II	I/II	SCLC	–		NCT04471727	Recruiting
TAK-186 (MVC-101)	Takeda, Maverick Therapeutics	anti-CD3	anti-EGFR	human serum albumin	I/II	I/II	HNSCC, SCLC, CRC	–		NCT04844073	Recruiting
		CD3 × HLA-intracellular oncoprotein									
RG6007 (RO7283420)	Roche	anti-CD3	HLA-A2-WT1		I	I	AML	anti-IL-6 (Tocilizumab), TKI (Dasatinib), chemotherapy		NCT04580121	Recruiting
		CD3 x affinity-enhanced TCR (ImmTAC)									
Tebentafusp (IMCgp100)	Immunocore	anti-CD3	gp100 TCR		FDA approved						
IMC-C103C (RG6290)	Immunocore, Roche	anti-CD3	MAGE-A4 TCR		I/II	I/II	Solid Tumors	anti-PD-1 (Atezolizumab)	IMC-C103C-101	NCT03973333	Recruiting
IMC-F106C	Immunocore	anti-CD3	PRAME TCR		I/II	I/II	Solid Tumors	anti-PD-(L)1		NCT04262466	Recruiting
		Vγ9Vδ2 TCR × TAA									
LAVA-051	Lava Therapeutics	anti-Vγ9Vδ2 TCR	anti-CD1d		I/II	I/II	CLL, MM, AML	–		NCT04887259	Recruiting
NK Cell Engagers											
AFM13	Affimed Therapeutics	anti-CD16a/FcγRIIIA	anti-CD30		II	II	T Cell Lymphoma, Mycosis Fungoides	–	REDIRECT	NCT04101331	Active, not recruiting
AFM24	Affimed Therapeutics	anti-CD16a/FcγRIIIA	anti-EGFR		I/II	I/II	Solid Tumors	–		NCT04259450	Recruiting
General immunoregulatory anti-cancer bsAb/msAb											
Group I (stimulating co-stimulatory molecules)											
		Co-stimulatory molecule x TAA/Tumor microenvironment protein									
BT7480	Bicycle Therapeutics	4-1BB agonist	anti-Nectin-4		I/II	I/II	Solid Tumors	anti-PD-1 (Nivolumab)	BT7480-100	NCT05163041	Recruiting
Cinrebafusp alfa (PRS-343)	Pieris Pharmaceuticals	4-1BB agonist	anti-HER2		II	II	HER2-positive GC	HER2 TKI (Tucatinib), anti-VEGFR2 (Ramucirumab), chemotherapy		NCT05190445	Recruiting
CB307	Crescendo Biologics	4-1BB agonist	anti-PSMA	human serum albumin	I	I	Solid Tumors	–	POTENTIA	NCT04839991	Recruiting
RG6076 (RO7227166)	Roche	4-1BBL	anti-CD19		I	I	NHL	CD3 x CD20 BiTE (Glofitamab), anti-CD20 (Obinutuzumab), anti-IL-6 (Tocilizumab)		NCT04077723	Recruiting
RG7827 (RO7122290)	Roche	4-1BBL	anti-FAP		I/II	I/II	CRC	CD3 x CEA BiTE (Cibisatamab), anti-CD20 (Obinutuzumab)		NCT04826003	Recruiting
RG6189 (RO7300490)	Roche	CD40 agonist	anti-FAP		I	I	Solid Tumors	anti-PD-1 (Atezolizumab)		NCT04857138	Recruiting
MP0317	Molecular Partners	CD40 agonist	anti-FAP		I	I	Solid Tumors	–		NCT05098405	Recruiting
REGN5678	Regeneron	CD28 agonist	anti-PSMA		I/II	I/II	PC	anti-PD-1 (Cemiplimab)		NCT03972657	Recruiting
REGN5668	Regeneron	CD28 agonist	anti-MUC16		I/II	I/II	Ovarian Cancer	anti-PD-1 (Cemiplimab), CD3 x MUC16 BiTE (Ubamatamab)		NCT04590326	Recruiting
REGN7075	Regeneron	CD28 agonist	anti-EGFR		I/II	I/II	Solid Tumors	anti-PD-1 (Cemiplimab)	COMBINE-EGFR-1	NCT04626635	Recruiting
		Co-stimulatory molecule x Co-stimulatory molecule									
GEN1042	Genmab, BioNTech	4-1BB agonist	CD40 agonist		I/II	I/II	Solid Tumors	anti-PD-1 (Pembrolizumab), chemotherapy		NCT04083599	Recruiting
Group II (blocking inhibitory molecules)											
		Inhibitory checkpoint x TAA									
Fidasimtamab (IBI-315)	Hanmi Pharmaceutical, Innovent Biologics	anti-PD-1	anti-HER2		I	I	Solid Tumors	–		NCT04162327	Recruiting
SSGJ-705	Sunshine Guojian Pharmaceutical	anti-PD-1	anti-HER2		I	I	Solid Tumors	–		NCT05145179	Not yet recruiting
		Inhibitory checkpoint x Inhibitory checkpoint									
SHR-2002	Jiangsu Hengrui	anti-TIGIT	anti-CD112R		I	I	Solid Tumors	anti-PD-1 (Camrelizumab), anti-PD-L1 (SHR-1316), PD-L1xTFG-βRII bsAb (SHR-1701)		NCT05198817	Enrolling by invitation
AGEN1777	Agenus, Bristol-Myers Squibb	anti-TIGIT	Undisclosed (T/NK cell inhibitory receptor)		I	I	Solid Tumors	anti-PD-1		NCT05025085	Recruiting
Vudalimab (XmAb717)	Xencor	anti-PD-1	anti-CTLA-4		II	I	Solid Tumors	–	DUET-2	NCT03517488	Active, not recruiting
Lorigerlimab (MGD019)	MacroGenics	anti-PD-1	anti-CTLA-4		II	II	Cervical Cancer	–	TRACTION	NCT05475171	Not yet recruiting
Cadonilimab (AK104)	Akeso Biopharma	anti-PD-1	anti-CTLA-4		III	I/II	GC, GEJC	chemotherapy		NCT03852251	Recruiting
Erfonrilimab (KN046)	Alphamab	anti-PD-L1	anti-CTLA-4		III	III	NSCLC	–		NCT04474119	Active, not recruiting
RG6139 (RO7247669)	Roche	anti-PD-1	anti-LAG-3		II	II	ESCC	–		NCT04785820	Recruiting
Tebotelimab (MGD013)	MacroGenics, Zai Lab	anti-PD-1	anti-LAG-3		II/III	II/III	GC, GEJC	anti-HER2 (Margetuximab), chemotherapy	MAHOGANY	NCT04082364	Active, not recruiting
ABL501	ABL Bio	anti-PD-L1	anti-LAG-3		I	I	Solid Tumors	–		NCT05101109	Recruiting
FS118	F-star Therapeutics	anti-PD-L1	anti-LAG-3		I/II	I/II	Solid Tumors	–		NCT03440437	Recruiting
IBI-323	Innovent Biologics	anti-PD-L1	anti-LAG-3		I	I	Solid Tumors	–		NCT04916119	Recruiting
NGM707	NGM Biopharmaceuticals	anti-LILRB1	anti-LILRB2		I/II	I/II	Solid Tumors	anti-PD-1 (Pembrolizumab)		NCT04913337	Recruiting
		Inhibitory checkpoint x Other inhibitory molecule									
HX009	Waterstone Hanxbio	anti-PD-1	anti-CD47		II	II	Solid Tumors	–		NCT04886271	Active, not recruiting
Simridarlimab (IBI-322)	Innovent Biologics	anti-PD-L1	anti-CD47		II	II	NSCLC	TKI (Lenvatinib), chemotherapy		NCT05296278	Not yet recruiting
LBL-015	Nanjing Leads Biolabs	anti-PD-1	TGF-βRII		I/II	I/II	Solid Tumors	–		NCT05107011	Recruiting
Retlirafusp alfa (SHR-1701)	Jiangsu Hengrui	anti-PD-L1	TGF-βRII		III	III	NSCLC	anti-VEGF (Bevacizumab), chemotherapy		NCT05132413	Not yet recruiting
BJ-005	BJ Bioscience	anti-PD-L1	TGF-βRII		I	I	Solid Tumors, Lymphoma	–		NCT05115292	Recruiting
TST005	Transcenta Holding	anti-PD-L1	TGF-βRII		I	I	Solid Tumors	–		NCT04958434	Recruiting
		Other inhibitory molecule x TAA									
BCA101	Bicara Therapeutics	TGF-β-trap	anti-EGFR		I/II	I/II	Solid Tumors	anti-PD-1 (Pembrolizumab)		NCT04429542	Recruiting
		Inhibitory checkpoint x Anti-angiogenesis									
Ivonescimab (AK112)	Akeso Biopharma	anti-PD-1	anti-VEGF		III	III	NSCLC	–		NCT05184712	Recruiting
Group III (targeting of co-stimulatory and inhibitory molecules)											
		Inhibitory checkpoint x Co-stimulatory molecule									
Izuralimab (XmAb104)	Xencor	anti-PD-1	ICOS agonist		I	I	Solid Tumors	anti-CTLA-4 (Ipilimumab)	DUET-3	NCT03752398	Recruiting
CDX-527	Celldex Therapeutics	anti-PD-1	CD27 agonist		I	I	Solid Tumors	–		NCT04440943	Recruiting
PRS-344 (S095012)	Pieris Pharmaceuticals, Servier	anti-PD-L1	4-1BB agonist		I/II	I/II	Solid Tumors	–		NCT05159388	Recruiting
LBL-024	Nanjing Leads Biolabs	anti-PD-L1	4-1BB agonist		I/II	I/II	Solid Tumors	–		NCT05170958	Recruiting
FS222	F-star Therapeutics	anti-PD-L1	4-1BB agonist		I	I	Solid Tumors	–		NCT04740424	Recruiting
MCLA-145	Merus, Incyte Corporation	anti-PD-L1	4-1BB agonist		I	I	Solid Tumors, B-cell Lymphoma	–		NCT03922204	Recruiting
ABL503 (TJ-L14B)	ABL Bio, I-MAB Biopharma	anti-PD-L1	4-1BB agonist		I	I	Solid Tumors	–		NCT04762641	Recruiting
DSP107	KAHR Medical	SIRPα	4-1BBL		I/II	I/II	Solid Tumors	anti-PD-1 (Atezolizumab)		NCT04440735	Recruiting
		Inhibitory checkpoint x Stimulatory cytokine (immunocytokine)									
AMG 256	Amgen	anti-PD-1	IL-21 mutein		I	I	Solid Tumors	–		NCT04362748	Recruiting
RG6279 (RO7284755)	Roche	anti-PD-1	IL-2v		I	I	Solid Tumors	–		NCT04303858	Recruiting
SAR445710 (KD033)	Kadmon Holdings, Sanofi	anti-PD-L1	IL-15	IL-15RA	I	I	Solid Tumors	–		NCT04242147	Recruiting
GI-101	GI Innovation, Simcere Pharmaceutical	CD80 (CTLA-4 trap)	IL-2v		I/II	I/II	Solid Tumors	anti-PD-1 (Pembrolizumab), TKI (Lenvatinib), Radiation		NCT04977453	Recruiting

The data are up to December 2022. For each agent, only one representative clinical trial is listed. All ongoing clinical trials for each agent are listed in Supplementary Table 4. The therapeutic combination described in the representative trial is a simplified summary. For each drug, the cases of all kinds of combinations of the drug and other agents in one or several cohorts in a multi-cohort study, or the combination of the drug and other agents in a mono-cohort study are not separately described

*FDA* Food and Drug Administration, *ALL* acute lymphoblastic leukemia, *MM* multiple myeloma, *FL* follicular lymphoma, *NHL* non-Hodgkin lymphoma, *CLL* chronic lymphocytic leukemia, *MDS* myelodysplastic syndrome, *DLBCL* diffuse large B cell lymphoma, *SCLC* small-cell lung cancer, *NSCLC* non-small cell lung cancer, *PC* prostate cancer, *HCC* hepatocellular carcinoma, *AML* acute myeloid leukemia, *HNSCC* head and neck squamous cell carcinoma, *CRC* colorectal cancer, *TKI* tyrosine kinase inhibitors, *GC* gastric cancer, *BiTE* bispecific T-cell engager, *GEJC* gastroesophageal junction cancer, *ESCC* esophageal squamous-cell carcinoma

BsAbs/msAbs have both similar and distinct mechanisms of action compared with mAbs. Fc-FcγR interactions are thought to be mainly responsible for the toxicity of early bsAbs, as in the case of the bispecific trifunctional antibody catumaxomab (anti-EpCAM×anti-×anti-CD3).^342^ Thus, now T cell engagers (TCEs) are mainly constructed without Fc segment or with a functionally silenced Fc segment. Complete removal of the Fc segment as in the cases of BiTEs and DARTs has not been the main trend and the development of many BiTEs and DARTs has been discontinued due to insufficient efficacy and safety issues. With the Fc segment silenced by mutation, the leading format of TCE development at present is the 1 + 1 asymmetric IgG-like form (Fig. 3a). The affinity toward different targets of a single bsAb can be fine-tuned by adjusting the two single-chain variable fragment (scFv) arms independently, thus ameliorating safety or pharmacokinetic/pharmacodynamic (PK/PD) properties. Moreover, in terms of PK/PD characteristics, the optimal dose for bsAbs is one that results in maximum target-bsAb-target trimer formation.^347^

Some bsAbs/msAbs can elicit biological effects that cannot be induced by the corresponding mAb mixture, therefore they are called obligate bsAbs/msAbs.^342^ For immunoregulatory anti-cancer bsAbs/msAbs, this has been demonstrated by redirecting CD3^+^ T cells, or immune cells expressing checkpoint receptors or co-stimulatory molecules to TAA-expressing cells or the TME. Moreover, bsAbs/msAbs binding different immunoregulatory targets can, at the same cellular spatial location, target multiple immunoreceptors or simultaneously enhance the co-stimulatory signal and inhibit immune checkpoints, hence potentially causing stronger anti-cancer immunity compared with the mAb mixture. These bsAbs/msAbs can be divided into cell engagers involving CD3, CD16a, or TAA-specific TCRs and general immunoregulatory anti-cancer bsAbs/msAbs combining all other immunoregulatory molecules or TAAs (Fig. 3c, d and Table 2).

In summary, bsAbs/msAbs have several potential advantages, including (1) superior specificity, safety, and therapeutic efficacy compared with the corresponding mixture of mAbs, (2) the ability to bridge and recruit immune cells, and (3) dual or multiple signal regulation. Nevertheless, disadvantages of bsAbs/msAbs still exist including chain mispairing in production, risk of inducing cytokine release syndrome (CRS), and the potential for inducing anti-drug antibodies (ADAs). In bsAb/msAb production, diverse combinations of light and heavy chains could lead to the dilution of the target bsAb, posing challenges in its isolation and resulting in low yield.^348,349^ Innovative development platforms, such as CrossMab^350,351^ and orthogonal Fab interface,^352^ have emerged to mitigate the impact of this issue. CRS is a common and distinctive adverse effect in the clinical application of bsAbs,^353–355^ mainly associated with TCEs containing the anti-CD3 arm. It is a systemic inflammatory response with symptoms ranging from fever, fatigue, and headache to multiorgan failure, triggered by T cell activation, with myeloid cells and TNF-α being the main mediators of the systemic cytokine release.^356,357^ To advance the further application of TCEs, the management of their using and the handling of adverse events should be improved, for example, with stepwise dosing, properly using tocilizumab, corticosteroids, or TNF-α blockade, and supportive^353–355,358^ care. Regarding the induction of ADAs, increased engineering and artificial design may result in greater differences between bsAbs and endogenous immunoglobulins, and bsAbs could therefore potentially contain new epitopes that elevate antigenicity and subsequently increase the likelihood of ADA development. Therefore, early monitoring of immunogenicity is crucial for increasing clinical success rates in bsAb development.^359,360^

### Bispecific T cell engagers

TCEs are representative obligate bsAbs combining anti-CD3 and anti-TAA scFvs to redirect any T cell to TAA-expressing tumor cells. TCEs make up nearly half of the immunoregulatory anti-cancer bsAbs/msAbs currently in clinical trials (Fig. 3b). Of note, the formats of TCEs comprise BiTE, dual-affinity re-targeting (DART), IgG-like full-length format, and others^342,343^ (Fig. 3a). Another type of TCE utilizing a TAA-specific TCR instead of an anti-TAA scFv is called ImmTAC. The development of TCEs surged after the approval of blinatumomab, which, as explained above, is an Fc-free BiTE. Blinatumomab yielded a CR rate of 43% in a phase II trial in Ph- relapsed or refractory (r/r) B-precursor acute lymphoblastic leukemia (ALL) patients^361^; it was thus approved by FDA in 2014. After blinatumomab, the CD3×CD20 IgG-like TCE mosunetuzumab was conditionally approved in the European Union,^362^ and also received accelerated approval by FDA in 2022 because it induced a CR rate of 60% for r/r follicular lymphoma (FL) in phase I and II trials.^363,364^ Likewise, teclistamab monotherapy was conditionally approved in the European Union^365^ and approved by FDA^366^ in 2022 for r/r multiple myeloma (MM) due to an ORR of 63.0%, a CR rate of 39.4% and mPFS of 11.3 months in the phase I/II MajesTEC-1 trial.^367,368^ Because of the reported mOS of 21.7 months in HLA-A*02:01^+^ uveal melanoma patients in a phase III trial,^369^ tebentafusp became the first approved ImmTAC in 2022.

The indications of TCEs depend on the TAA expression of the cancer type. For example, TCEs targeting CD20, CD19, and CD38 are all designed for hematological malignancies and are rivals of CAR-T cell therapies in hematology. The development of TCEs against solid tumors seems more challenging. Challenges include heterogeneity in TAA expression, on-target off-tumor toxicity for normal tissue, the immunosuppressive TME, disordered vasculature, and limited tumor penetration. These challenges might be overcome by further structure design exploration, antibody avidity fine-tuning, or therapy combinations.

At present, TCEs that have been approved or entered phase III clinical trials all target hematological TAAs. TCEs advanced into phase III trials before approval include epcoritamab (CD3×CD20), glofitamab (CD3×CD20), and elranatamab (CD3×B-cell maturation antigen) (Table 2 and Supplementary Table 4). For epcoritamab, the phase I/II EPCORE NHL-1 study showed an ORR of 68% and 90% for r/r B-cell non-Hodgkin lymphoma (B-NHL) and r/r FL patients with monotherapy,^370^ supporting the ongoing phase III EPCORE DLBCL-1 study. The majority of trials of glofitamab combine it with rituximab, obinutuzumab, or tocilizumab pretreatment to mitigate cytokine release.^371^ A phase I study combining glofitamab and obinutuzumab pretreatment in r/r B-NHL patients showed an ORR of 53.8% and a CR rate of 36.8%.^372^ For elranatamab, the phase I MagnetisMM-1 study has demonstrated an ORR of 75% at high doses,^373^ supporting two ongoing phase III trials.

Beyond conventional TCEs, other components are introduced in novel formats to refine immunostimulatory properties, PK/PD attributes, and toxicity (Fig. 3d and Table 2). By introducing a CD28 immunostimulatory arm, Sanofi designed Fc-silenced CD3 × CD38 × CD28 TCE with better stimulation of anti-tumoral T cells.^374^ Based on this design, SAR442257 has been developed and is being tested in a phase I trial (NCT04401020). Another category called Tri-specific T Cell-Activating Construct (TriTAC) introduced anti-human serum albumin scFv to improve PK/PD properties for solid tumors. Preclinical results showed superior T-cell killing compared with conventional BiTEs targeting EGFR or PSMA and favorable efficacy,^375^ supporting phase I/II trials (Table 2 and Supplementary Table 4). To improve safety, XTENylated protease-activated T cell engagers (XPATs) were created by introducing scFvs with TME-specific degradable masking, thus avoiding off-tumor T cell activation. Sanofi completed the acquisition of this technology in 2022, including the HER2 XPAT AMX-818. Moreover, as functions are being continuously discovered, innate immune cell populations are also evaluated for immuno-oncology agent development. BsAbs targeting CD16A/FcγRIIIa, an activating FcγR, to redirect NK cells to TAA-expressing cells are called NK cell engagers (NKEs) or innate cell engagers (ICEs) (Table 2 and Supplementary Table 4). Unfortunately, AFM13, a representative CD16A × CD30 NKE for r/r Hodgkin lymphoma, only induced an ORR below 25% in several trials as monotherapy.^376,377^ However, an ORR of 88% was induced by combining AFM13 and pembrolizumab,^378^ suggesting combination therapy for further development.

### General immunoregulatory anti-cancer bsAbs/msAbs

Apart from CD3-engaging TCEs, many other anti-cancer bsAbs/msAbs target immunoregulatory proteins other than the CD3 complex. Based on the design, this category includes three subgroups: bsAbs/msAbs stimulating co-stimulatory molecules (group I), blocking immune checkpoints (group II), and the combination of these two tactics (group III) (Fig. 3d, Table 2 and Supplementary Table 4). These bsAbs/msAbs are currently mainly developed for the treatment of solid tumors.

Two designs are used for group I bsAbs/msAbs (Table 2). The first one is by binding co-stimulatory molecules on immune cells and TAA-expressing tumor cells or fibroblast activation protein on cancer-associated fibroblasts. The second one is to concurrently target distinct IgSF/TNFRSF co-stimulatory molecules on immune cells.

Group II bsAbs/msAbs include three subtypes (Table 2). The first one redirects PD-1/PD-L1 blockade toward TAAs or tyrosine kinase expression-enriched TME. The second one concurrently targets different immune checkpoint ligand-receptor axes. Due to thorough research on ICI combination therapies, the development of this subtype is the main trend for group II bsAbs/msAbs and is also most advanced in this category. The third one targets PD-1/PD-L1 and immunosuppressive molecules beyond IgSF checkpoints, such as CD47 and TGF-βRII. Group III includes designs mainly combining anti-PD-1/PD-L1 and co-stimulatory agonist arms, and fusion proteins combining ICI and immunostimulatory cytokines (immunocytokines) (Fig. 3d and Table 2). In a preclinical study, anti-PD1–IL-2v immunocytokine was proved to have superior ability to expand tumor-specific CD8^+^ effector-like T cells and therapeutic efficacy than the (agonistic) IL-2Rβγ-biased mutant IL-2 variant IL-2v in tandem with an anti-FAP scFv.^379^ These findings support the clinical development of RG6279, a bispecific anti-PD1–IL-2v fusion protein directing IL-2v to PD-1^+^ tumor-reactive T cells.

Encouraging preclinical results have been reported for various agents in these bsAb categories, including 4-1BB×HER2,^380^ 4-1BB×CD40,^381^ 4-1BB×PD-1/PD-L1,^382,383^ PD-1×GITRL,^384^ PD-L1×LAG-3,^385^ PD-1×CTLA-4,^386^ PD-L1×IL-15/IL-15RA^387^ bispecifics, and others. However, in general, except for several group II bsAbs, most others are still at early phases of development. BsAbs entered in phase III trials include cadonilimab, erfonrilimab, tebotelimab, retlirafusp alfa, and ivonescimab (Table 2 and Supplementary Table 4). Cadonilimab is an Fc-silenced symmetric IgG1 PD-1×CTLA-4 bsAb. Combined with chemotherapy, cadonilimab elicited an ORR of 65.9% in phase I/II trial for GC/GEJC.^388^ In the phase I/II trial for PD-L1 TPS ≥ 1% NSCLC, cadonilimab combined with anlotinib induced an ORR of 62.5%.^389^ Thus, cadonilimab combined with chemotherapy or targeted therapy elicited excellent ORRs (Table 2). Erfonrilimab is a symmetric full-length IgG1 PD-1×CTLA-4 bsAb. Combined with chemotherapy, erfonrilimab induced ORRs of 50.6%, 58.3%, and 55.6% in NSCLC,^390^ ESCC,^391^ and PDAC patients.^392^ A similar ORR of 57% was also observed combining erfonrilimab and lenvatinib in HCC patients.^393^ Tebotelimab is a PD-1×LAG-3 Fc-preserved DART molecule. Combined with anti-HER2 mAb margetuximab, tebotelimab induced a preliminary ORR of 40% in HER2^+^ malignancies. The phase II/III MAHOGANY study combining margetuximab and tebotelimab or retifanlimab ± chemotherapy in HER2^+^ GC/GEJC is ongoing. Retlirafusp alfa is an anti-PD-L1–TGF-βII fusion protein. In phase I trials in advanced solid tumors, NSCLC with EGFR mutation, and GC, retlirafusp alfa induced medium ORRs of 17.8%,^394^ 16.7%,^395^ and 19.4%,^396^ but an impressive ORR of 44.2% was observed in the PD-L1^+^ NSCLC cohort.^397^ The efficacy of retlirafusp alfa needs further confirmation since its design is similar to bintrafusp alfa, which was removed from the pipeline of Merck, owing to its inferiority compared to pembrolizumab in a phase III trial.^398^ Ivonescimab is a symmetric IgG1 PD-1×VEGF bsAb. Combined with chemotherapy, ivonescimab induced a high ORR of 40.0% and 76.9% in r/r NSCLC and treatment-naïve NSCLC.^399^ At present, excellent ORR endpoints of phase I/II trials are mostly observed in therapeutic settings combining general immunoregulatory bsAbs with other treatments. Results of currently ongoing phase III trials are eagerly awaited.

## Immuno-epigenetics

**Fig. 4:**
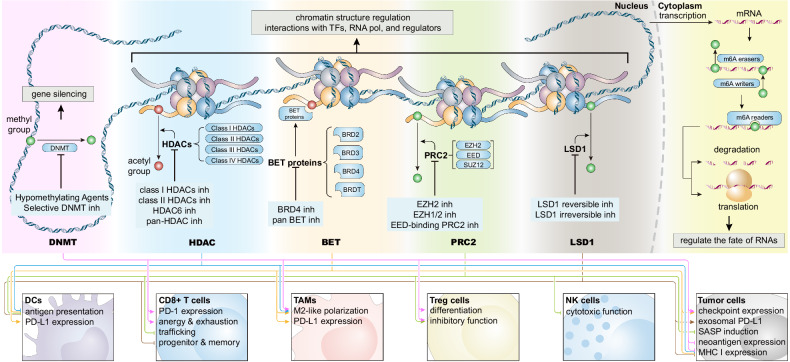
Epigenetic targets and their impact on different immune cell types and tumor cells in the TME. Lines of the same color indicate the impacts on different cell types of the same epigenetic process. And the color of lines corresponds to the background color of the specific process those lines indicated. Epigenetic regulation mainly comprises transcriptional regulation via DNA methylation, histone modification, and post-transcriptional modification. Immune-related pharmacological development has mainly focused on DNA methylation by DNMTs, histone deacetylation by HDACs, recognition of acetylated histone by BET proteins, and histone demethylation by PRC2 and LSD1. DNA methylation, which is mainly mediated by DNMTs, represses gene transcription when located in a gene promoter and regulates anti-tumor immunity with the orchestration of different cell members. The aforementioned histone modifications are capable of remodeling chromatin structures and interactions with other regulating factors (e.g., recruitment of transcription factors) and affect gene transcription of various cell types in the TME. The post-transcriptional m6A methylation represents a new layer of epigenetic regulation that mainly affects the fate of RNAs via promoting or antagonizing their degradation or translation. Classification of drugs of each epigenetic target are indicated in the blue boxes. inh inhibitor, DNMT DNA methyltransferase, HDAC histone deacetylase, BET bromodomain and extraterminal domain, BRD bromodomain, BRDT bromodomain testis-specific protein, RNA pol RNA polymerase, PRC2 polycomb repressive complex 2, EZH enhancer of zeste homolog, EED embryonic ectoderm development, SUZ suppressor of zeste, LSD1 lysine-specific demethylase 1, m6A N6-methyladenosine, SASP senescence-associated secretory phenotype

Epigenetics refers to gene expression fine-tuning without changes in DNA sequence mainly via selective transcription; it mainly includes DNA methylation, histone modification, and chromatin remodeling.^400,401^ It plays a critical regulatory role in a variety of physiological and pathological processes.^400–402^ N6-methyladenosine (m6A) RNA modification, the most common RNA methylation, is closely associated with cancer progression, drug resistance^373,374^ and cancer immunity.^403–405^ Notably, antagonizing m6A modifiers can sensitize tumors to PD-1 blockade in mice.^406–410^ However, most agents targeting m6A regulators are still in preclinical development and none has entered clinical evaluation.^411^ Thus, considering the volume and scope of this review, we mainly focus on DNA methylation by DNA methyltransferases (DNMTs), histone deacetylation by histone deacetylases (HDACs), recognition of acetylated histone by the mammalian bromodomain and extra-terminal (BET) proteins, and demethylation by histone methylase polycomb repressive complex 2 (PRC2) and lysine-specific histone demethylase 1 (LSD1) (Fig. 4).

### DNA methyltransferases (DNMTs)

Targeting DNA methylation has become important for the treatment of certain hematological malignancies with the intention to reactivate tumor suppressors and promote differentiation of the malignant cells. Regarding anti-tumor immunity, therapeutic DNA demethylation can enhance tumor immunogenicity by inducing expression of endogeneous retroviral elements and of neoantigens normally silenced by DNA methylation. Expression of the former induces double-strand RNA which, in turn, can induce interferon-based innate immune activation essential for adaptive antitumor immunity, and is one reason why DNMT inhibitors can cause immunogenic cell death of malignant cells.^412,413^ Furthermore, therapeutic DNA demethylation can alter the composition and behavior of immune cells; it can increase the expression of MHC molecules, alleviate T cell exhaustion and enhance T cell effector and memory potential, increase secretion of Th1-type cytokines, and reduce immunosuppressive myeloid and Treg cells.^412,413^ DNA methylation status and demethylating agents can also directly affect the expression of multiple immune checkpoints, including PD-1,^414,415^ PD-L1^,^^414,416,417^, LAG3,^414^ TIM-3^,^^414,418,419^, CTLA-4,^414,420^ and TIGIT,^421,422^ by recruiting of proteins involved in gene repression or by inhibiting the binding of them.

Because of these interesting antitumor immune effects, combinations of hypomethylating agents (HMAs), currently mainly DNMT inhibitors, with immunotherapeutics are being investigated. Decitabine plus camrelizumab caused high response rates and long-term benefits in patients with Hodgkin’s lymphoma who failed PD-1 inhibitors.^423,424^ The combination of decitabine and pembrolizumab induced better response in patients with relapsed AML, with transcriptional signs of immune activation.^425^ Other combinations of HMAs and ICIs also show good safety and preliminary anti-tumor effects in patients with hematological malignancies in clinical trials^426–429^ (Supplementary Table 5). Regarding solid tumors, although the preclinical and some early clinical results using the combination of PD-1 blockade and HMAs are highly promising,^430–432^ most clinical data has been disappointing. No responses were observed after guadecitabine plus atezolizumab in metastatic urothelial carcinoma which had progressed on previous immune checkpoint blockade (ICB).^433^ The combination of guadecitabine or azacytidine and pembrolizumab or durvalumab produced only modest anti-tumor effects in a variety of solid tumors.^434–436^ The addition of azacytidine or CC-486 (oral azacytidine) to pembrolizumab^437,438^ or durvalumab^439^ was not more effective than standalone ICI treatment. Lack of robust tumor DNA demethylation and of viral mimicry was found to be associated with a missing clinical response in one study.^439^

Overall, the combination of HMAs and ICIs needs further studies, especially in solid tumors. Notably, investigations of how dosing and scheduling of these drug classes affect the immunomodulatory and anti-tumor effects in the clinical setting are expected. In mouse solid tumor models, low-dose HMAs plus ICIs outperform either HMAs or ICIs alone in restricting tumor growth and prolonging survival, with significant HMA-related immune modulation.^430,431^ Epigenetic priming using HMAs with sequential ICIs has the potential to produce durable clinical benefit associated with immune responses in patients with solid tumors.^440,441^ In addition, there is considerable interest in the development of compounds targeting a selective subtype of DNMTs, which may enhance the tolerability and efficacy.^442–444^ CAR T cells pretreated with low-dose decitabine can show enhanced anti-tumor activity and persistence,^430^ and cell products primed with demethylating agents are undergoing clinical evaluation (Supplementary Table 5).

### Histone deacetylases (HDACs)

As important epigenetic writers, HDACs include four classes of proteins, of which HDAC I, II, IV are Zn^+^ dependent, whereas HDAC III is not (HDAC I: HDAC1-3, 8; HDAC II: HDAC4-7, 9, 20; HDAC IV: HDAC 11; HDAC III: SIRT1-7). HDAC inhibition can profoundly affect anti-tumor immune responses, including enhancing MHC class I antigen presentation,^445^ promoting M1-like polarization of TAMs,^446,447^ and depleting MDSCs.^448^ HDAC inhibition can maintain intra-tumoral macrophages with a pro-inflammatory tumoricidal phenotype and preserve their ability to conduct ADCC needed by ADCC-dependent therapeutic antibodies,^449^ which cannot be achieved by the depletion of TAMs (Fig. 4). To date, both selective and pan-HDAC inhibitors have been developed (Table 3 and Supplementary Table 5). Early attempts inhibiting class I^450–452^ or II^453,454^ HDACs have produced suboptimal results in solid tumors, which may be explained by the selective inhibition of immunosuppressive polymorphonuclear MDSCs and monocytic-MDSCs by the class I HDAC inhibitor entinostat and the class II HDAC inhibitor ricolinostat, respectively.^455^ Thus, novel selective HDAC inhibitors with superior immunostimulatory activity as well as inhibitors of more classes of HDACs to completely inhibit different MDSCs subsets, such as class I/II HDAC and pan-HDAC inhibitors may be more effective. For example, domatinostat, a novel class I HDAC inhibitor, has demonstrated good tolerability and preliminary effectiveness as adjuvant to checkpoint blockade.^456,457^ In the SENSITIZE trial, domatinostat treatment increased expression of antigen processing-related genes and MHC molecules along with enhanced cytotoxic T cell infiltration in some patients with advanced melanoma who had failed PD-1 blockade, with tumors either immunologically cold or hot.^458^ Domatinostat has obtained FDA approval as an investigational new drug allowing the clinical evaluation in various solid tumors to overcome resistance to ICIs (Supplementary Table 5). Clinical performances of class I/II HDAC inhibitors vary across cancer types and regimens. Vorinostat demonstrated only modest activity when used with pembrolizumab in HNSCC,^459^ NSCLC,^460^ and breast cancer.^461^ Another class I/II HDAC inhibitor, resminostat, induced a CR rate of 54.8% in basal cell carcinoma in a phase II study.^462^ However, the results in biliary tract cancer^463^ and liver cancer^464^ were disappointing. Pan-HDAC inhibitors suppressing the activity of Zn^+^ dependent HDACs (class I, II, and IV) have entered phase III trials due to their success in MM and other hematological malignancies (Table 3). However, the accelerated FDA approval of the panobinostat plus bortezomib combination for MM has been withdrawn in 2021 due to the minimal survival benefit and high TRAEs-related discontinuation rate^465,466^ as well as inadequate follow-up studies confirming the prolonged PFS in the PANORAMA1 study. Encouragingly, optimization of dosing^467^ and administration route^468^ may improve the tolerability of this regimen. And the efficacy of panobinostat in patients with solid tumors remains to be tested. Additionally, some other pan-HDAC inhibitors have shown favorable tolerability and efficacy in solid tumors in phase I and II trials,^469,470^ calling for more advanced clinical evaluations.

**Table 3:** Therapeutics targeting immuno-epigenetics and cytokines

Basic information					Representative trial					
Target	Agent	Manufacturer	Agent type	Highest developmental phase	Phase	Cancer type	Therapeutic combination	Study	Identifier	Status
Immuno-epigenetics										
DNMTs										
	azacytidine	Pfizer	small molecule inh	FDA Approved	II	Pancreatic Cancer	anti-PD-1 (Pembrolizumab)		NCT03264404	Active, not recruiting
	CC-486	Pfizer	small molecule inh	FDA Approved	II	NSCLC	anti-PD-1 (Pembrolizumab)		NCT02546986	Active, not recruiting
	decitabine	Janssen-Cilag/Otsuka Pharmaceutical	small molecule inh	FDA Approved	II	Breast Cancer	anti-PD-1 (Pembrolizumab), chemotherapy		NCT02957968	Active, not recruiting
	guadecitabine	Astex Pharmaceuticals	small molecule inh	Phase III	I	Lung Cancer	anti-PD-1 (Pembrolizumab), HDAC inh (Mocetinostat)		NCT03220477	Active, not recruiting
	ASTX727	Astex Pharmaceuticals	small molecule inh	FDA Approved	I/II	Melanoma	anti-PD-1 (Nivolumab)		NCT05089370	Recruiting
HDACs										
class I HDACs	Romidepsin (FK228, Depsipeptide, FR 901228, NSC 630176)	Celgene Corporation	small molecule inhibitor	FDA approved	III	T-Cell Lymphoma	aurora A kinase inhibitor (Alisertib), chemotherapy		NCT01482962	Completed
	Entinostat (SNDX-275, MS 275, MS 27-275, KHK2375)	Syndax Pharmaceuticals	small molecule inhibitor	III	III	Breast Cancer	hormone therapy (Exemestane)		NCT03538171	Active, not recruiting
	Mocetinostat (MGCD0103, MG-0103)	Mirati Therapeutics	small molecule inhibitor	II	II	UC	–		NCT02236195	Completed
	Domatinostat (4SC-202)	4SC AG	small molecule inhibitor	II	II	Gastrointestinal Cancer	anti-PD-L1 (Avelumab)	EMERGE	NCT03812796	Recruiting
	Nanatinostat (Tractinostat, VRx-3996, CHR-3996)	Viracta Therapeutics	small molecule inhibitor	II	II	EBV-associated Lymphoma	anti-virus (Valganciclovir)	NAVAL-1	NCT05011058	Recruiting
	OKI-179	OnKure	small molecule inhibitor	I/II	I/II	Melanoma	MEK1/2 inhibitor (Binimetinib)		NCT05340621	Recruiting
	Zabinostat (CXD101)	Celleron Therapeutics	small molecule inhibitor	I/II	I/II	CRC	anti-PD-1 (Nivolumab)	CAROSELL	NCT03993626	Active, not recruiting
Class II HDAC6	Ricolinostat (ACY-1215)	Regenacy Pharmaceuticals	small molecule inhibitor	I/II	I/II	MM	–	ACY-1215	NCT01323751	Completed
	Citarinostat (ACY-241)	Celgene Corporation	small molecule inhibitor	I	I	MM	IMiD (Pomalidomide), chemotherapy		NCT02400242	Active, not recruiting
	KA-2507	Karus Therapeutics Limited	small molecule inhibitor	II	I	Solid Tumors	–		NCT03008018	Completed
Class I and II HDACs	Vorinostat (Suberoylanilide hydroxamic acid, Zolinza, L-001079038)	Merck & Co.	small molecule inhibitor	FDA approved	III	CTCL	anti-CCR4 (KW-0761)	MAVORIC	NCT01728805	Completed
	Tucidinostat (Chidamide, HBI-8000)	HUYA Bioscience International	small molecule inhibitor	III	III	DLBCL	anti-CD20 (Rituximab), chemotherapy	DEB	NCT04231448	Recruiting
Class I, II and IV HDACs (pan-HDAC)	Panobinostat (LBH 589, Farydak, MTX110)	Novartis/Secura Bio	small molecule inhibitor	FDA approved	III	MM	proteasome inhibitor (Bortezomib), Dexamethasone	PANORAMA-1	NCT01023308	Completed
	Abexinostat (PCI 24781, CRA-24781)	Xynomic Pharmaceuticals	small molecule inhibitor	III	III	RCC	TKI (Pazopanib)	RENAVIV	NCT03592472	Recruiting
	Pracinostat (SB-939)	MEI Pharma	small molecule inhibitor	III	II	MDS	chemotherapy	MEI-005	NCT01993641	Completed
	Resminostat (4SC-201, RAS2410)	4SC AG	small molecule inhibitor	II	II	HCC	Raf inhibitor (Sorafenib)	Shelter	NCT00943449	Completed
HDAC and other molecules	Fimepinostat (CUDC-907)	Curis	PI3K/HDAC small molecule dual inhibitor	II	II	DLBCL	–		NCT02674750	Completed
BET family proteins										
BET proteins (pan-BET)	INCB057643	Incyte Corporation	small molecule inhibitor	I/II	I	Myeloid Neoplasms	stem cell transplantation, JAK1/2 Inhibitor (Ruxolitinib)		NCT04279847	Recruiting
	RO6870810 (RG 6146)	Roche	small molecule inhibitor	I	I	MM	anti-CD38 (Daratumumab)		NCT03068351	Completed
	Molibresib (I-BET-762, GSK525762)	GlaxoSmithKline	small molecule inhibitor	II	II	Hematological malignancies	–		NCT01943851	Completed
	Mivebresib (ABBV-075)	AbbVie	small molecule inhibitor	I	I	Myelofibrosis	chemotherapy, JAK1/2 inhibitor (Ruxolitinib)		NCT04480086	Active, not recruiting
	Trotabresib (CC-90010)	Celgene Corporation	small molecule inhibitor	I	I	Solid tumors	–		NCT03220347	Recruiting
	Pelabresib (CPI-0610)	MorphoSys	small molecule inhibitor	III	III	Myelofibrosis	JAK1/2 inhibitor (Ruxolitinib)	MANIFEST-2	NCT04603495	Recruiting
	BMS-986158	Bristol Myers Squibb	small molecule inhibitor	I/II	I/II	MM	EZH2 inhibitor (Tazemetostat), MEK1/2 inhibitor (Trametinib), dexamethasone		NCT05372354	Not yet recruiting
	ODM-207	OrionPharma	small molecule inhibitor	I/II	I/II	Solid Tumors	–	BETIDES	NCT03035591	Completed
BRD4	PLX2853	Daiichi Sankyo group	small molecule inhibitor	I/II	I/II	Ovarian Cancer	chemotherapy		NCT04493619	Active, not recruiting
	PLX51107	Daiichi Sankyo group	small molecule inhibitor	I	I	AML, MDS	chemotherapy		NCT04022785	Recruiting
	BI 894999	Boehringer Ingelheim	small molecule inhibitor	I	I	Solid tumors	–		NCT02516553	Completed
	AZD5153 (SRA 515)	AstraZeneca	small molecule inhibitor	I/II	I/II	AML	BCL2 inhibitor (Venetoclax)		NCT03013998	Recruiting
PRC2 pathway										
EZH2	Tazemetostat	Epizyme Inc	small molecule inhibitor	FDA approved	III	FL	IMiD (Lenalidomide), anti-CD20 (Rituximab)		NCT04224493	Recruiting
	CPI-0209	Constellation Pharmaceuticals Inc	small molecule inhibitor	I/II	I/II	Solid Tumors and lymphoma	–		NCT04104776	Recruiting
LSD1 pathway										
LSD1	Ladademstat (ORY1001, RG-6016)	Oryzon Genomics	TCP-based irreversible LSD1 inhibitor	II	II	SCLC, Neuroendocrine Cancer	chemotherapy		NCT05420636	Not yet recruiting
	INCB059872	Incyte Corporation	TCP-based irreversible LSD1 inhibitor	I/II	I/II	Solid Tumors, Hematological Neoplasms	chemotherapy, anti-PD-1 (Nivolumab)		NCT02712905	Active, not recruiting
	Pulrodemstat (CC-90011)	Celgene Corporation	reversible LSD1 inhibitor	II	II	Malignancies	anti-PD-1 (Nivolumab)		NCT04350463	Recruiting
	Seclidemstat (SP-2577)	Salarius Pharmaceuticals	reversible LSD1 inhibitor	I/II	I/II	CML, MDS	chemotherapy		NCT04734990	Recruiting
PRMT5 pathway										
PRMT5	Pemrametostat (GSK-3326595)	GlaxoSmithKline, Epizyme	small molecule inhibitor	II	II	Breast Cancer	–	OTT-19-06	NCT04676516	Not yet recruiting
	PF-06939999	Pfizer	small molecule inhibitor	I	I	Solid Tumors	chemotherapy		NCT03854227	Active, not recruiting
	PRT543	Prelude Therapeutics	small molecule inhibitor	I	I	Malignancies	–		NCT03886831	Active, not recruiting
Cytokines										
Targeting Interleukins										
IL-2	aldesleukin	Clinigen/Novartis	rhIL-2	FDA approved	III	Melanoma	chemotherapy, G-CSF (Filgrastim), IFN-α	PROCLIVITY01	NCT00006237	Completed
	THOR-707 (SAR444245)	Synthorx Inc	non-α IL-2 variant	II	II	HNSCC	anti-PD-1 (Pembrolizumab), anti-EGFR (Cetuximab)		NCT05061420	Recruiting
	SHR-1916	Jiangsu Hengrui Medicine	non-α IL-2 variant	I	I	Solid Tumors	–		NCT04842630	Recruiting
	Nemvaleukin alfa (ALKS 4230)	Alkermes plc	non-α IL-2 variant-IL-2Rα fusion protein (blocking the IL-2Rα binding)	III	III	Ovarian Cancer, Fallopian Tube Cancer, Primary Peritoneal Cancer	anti-PD-1 (Pembrolizumab)	ARTISTRY-7	NCT05092360	Recruiting
	Simlukafusp alfa (SIM, FAP-IL2v, RO6874281)	Roche	IL-2 variant-anti-FAP antibody fusion protein	II	II	Head and Neck, esophageal and cervical Cancers	anti-PD-L1 (Atezolizumab)		NCT03386721	Completed
	Eciskafusp Alfa (PD-1–IL2v)	Hoffmann-La Roche	IL-2 variant-anti-PD-1 antibody fusion protein	I	I	Solid Tumors	anti-PD-1 (Atezolizumab)		NCT04303858	Recruiting
	CUE-101 (E7-pHLA-IL2-Fc)	Cue Biopharma	IL-2 variant-HLA complex+ HPV16 E7 peptide epitope fusion protein	I	I	HPV16 + HNSCC	anti-PD-1 (Pembrolizumab)		NCT03978689	Recruiting
	GI-101 (CD80-IgG4-IL-2-Fc)	GI Innovation	IL-2 variant-anti-CD80 antibody fusion protein	I/II	I/II	Solid Tumors	anti-PD-1 (Pembrolizumab), multi-kinase inh (Lenvatinib), radiation		NCT04977453	Recruiting
	Cergutuzumab amunaleukin (CEA–IL2v)	Hoffmann-La Roche	IL-2 variant-anti-CEA antibody fusion protein	I	I	Solid tumors	anti-PD-1 (Atezolizumab)		NCT02350673	Completed
	MDNA11	Medicenna Therapeutics	IL-2 variant-rhalbumin fusion protein	I/II	I/II	Solid Tumors	checkpoint inhibitor	ABILITY	NCT05086692	Recruiting
	Darleukin (L19-IL2, Daromun, Philogen)	Philogen SpA	IL-2-anti-ED-B fibronectin antibody fusion protein	III	III	Melanoma	ADC (L19-TNF)	Pivotal	NCT02938299	Recruiting
	STK-012	Synthekine	α/β-IL-2 variant	I	I	Solid Tumors	anti-PD-1 (Pembrolizumab)		NCT05098132	Recruiting
	RG6292 (RO7296682)	Hoffmann la Roche	anti-CD25 mAb	I	I	Solid Tumors	anti-PD-L1 (Atezolizumab)		NCT04642365	Recruiting
	XTX202	Xilio Therapeutics	conditionally-activated IL-2	I/II	I/II	Solid Tumors	–		NCT05052268	Recruiting
IL-15	NIZ985 (hetIL-15)	Admune Therapeutics	IL-15-IL-15Rα fusion protein	I	I	Solid Tumors	anti-PD-1 (Spartalizumab)		NCT02452268	Completed
	XmAb24306 (RO7310729, RG6323)	Xencor Inc	IL-15-IL-15Rα fusion protein	I	I	Solid Tumors	anti-PD-L1 (Atezolizumab)		NCT04250155	Recruiting
	Inbakicept (N-803, ALT-803)	ImmunityBio	IL-15-IL-15Rα fusion protein	III	III	NSCLC	anti-PD-1 (Pembrolizumab), chemotherapy	QUILT 2.023	NCT03520686	Recruiting
	SHR-1501	Jiangsu Hengrui Medicine	IL-15-IL-15Rα fusion protein	I	I	Malignancies	anti-PD-L1 (SHR-1316)		NCT03995472	Recruiting
	BJ-001	BJ Bioscience	IL-15-IL-15Rα-integrin-binding motif fusion protein	I	I/II	Solid Tumors	anti-EGFR (Cetuximab)		NCT04616196	Recruiting
	NKTR-255	Nkarta Therapeutics	polymer conjugated IL-15	II	II	UC	anti-PD-L1 (Avelumab)	JAVELIN Bladder Medley	NCT05327530	Not yet recruiting
IL-10	Ilodecakin (Pegilodecakin, AM0010)	ARMO BioSciences	PEGylated human IL-10	III	III	Pancreatic Cancer	chemotherapy	Sequoia	NCT02923921	Completed
IL-12	NHS-IL12 (M9241)	National Cancer Institute (USA)	IL-12-anti-DNA-histone H1 complex mAb fusion protein	II	II	Bowel Cancers, CRC	cancer vaccine (CV301), PD-L1 x TGFβ bsAb (MSB0011359C), IL-15 superagonist (N-803)		NCT04491955	Recruiting
	SON 1010 (IL12FHAB)	Sonnet Biotherapeutics	IL-12-FHAB fusion protein	I	I	Solid Tumors	–		NCT05352750	Recruiting
	GEN-1 (EGEN-001)	Celsion Corporation	plasmid-encoded IL-12	II	II	Ovarian Carcinoma, Fallopian Tube Carcinoma, Primary Peritoneal Carcinoma	–		NCT01118052	Completed
	MEDI 0457 (INO-9012)	AstraZeneca/Inovio Pharmaceuticals	plasmid-encoded IL-12	II	II	HPV-associated Cancers	anti-PD-L1 (Durvalumab)		NCT03439085	Active, not recruiting
	Tavokinogene telseplasmid	OncoSec Medical	plasmid-encoded IL-12	II	II	Melanoma	anti-PD-1 (Pembrolizumab)	Keynote695	NCT03132675	Recruiting
	INXN 2001 (ad-RTS-hIL-12)	ZIOPHARM Oncology Inc	adenovirus encoding activable IL-12	II	II	Glioblastoma	anti-PD-1 (Cemiplimab)		NCT04006119	Completed
Targeting TGF-β										
TGF-β	NIS793	Novartis	antagonistic pan-TGF-β mAb	III	III	PDAC	chemotherapy	daNIS-2	NCT04935359	Recruiting
	AVID-200 (BMS-986416)	Formation Biologics, Bristol-Myers Squibb	TGF-βR ECD-Fc fusion protein (TGF-β1/3 Trap)	I	I	Solid Tumors	anti-PD-1 (Nivolumab)		NCT04943900	Recruiting
	Trabedersen	Oncotelic Therapeutics	antisense against TGF-β2	II	II	Glioblastoma	–		NCT00431561	Completed
TGF-βR	Vactosertib (TEW-7197)	MedPacto	small molecule inhibitor	II	II	CRC	anti-PD-1 (Pembrolizumab)		NCT03844750	Recruiting
	YL-13027	Shanghai Yingli Pharmaceutical	small molecule inhibitor	I	I	Solid Tumors	–		NCT03869632	Recruiting
Activation of L-TGF-β	SRK-181	Scholar Rock	selective L-TGF-β1 antagonistic mAb	I	I	Solid Tumors	anti-PD-1/PD-L1	DRAGON	NCT04291079	Recruiting
	ABBV-151	AbbVie	selective L-TGF-β1 antagonistic mAb	I	I	Solid Tumors	anti-PD-1 (Budigalimab)		NCT03821935	Recruiting
	PF-06940434	Pfizer	αvβ8 integrin mAb	I	I	Solid Tumors	anti-PD-1 (Sasanlimab)		NCT04152018	Recruiting
Targeting chemokines										
CXCR4	Balixafortide (POL6326)	Spexis	small molecule inhibitor	III	III	Breast Cancer	microtubule inhibitor (Eribulin)	FORTRESS	NCT03786094	Active, not recruiting
	Motixafortide (BL-8040)	Biokine Therapeutics, BioLineRx	small molecule inhibitor	III	III	MM	G-GSF, stem cell transplantation	GENESIS	NCT03246529	Active, not recruiting
	Mavorixafor (X4P-001)	Sanofi, X4 Pharmaceuticals	small molecule inhibitor	I/II	I/II	ccRCC	VEGFR1/2/3 inhibitor (Axitinib)		NCT02667886	Active, not recruiting
CXCR2	AZD5069	AstraZeneca	small molecule inhibitor	I/II	I/II	Solid Tumors	anti-PD-L1 (MEDI4736), anti-CTLA-4 (Tremelimumab)	SCORES	NCT02499328	Active, not recruiting
	SX-682	Syntrix Biosystems Inc	CXCR1/2 small molecule inhibitor	I/II	I/II	CRC	anti-PD-1 (Nivolumab)	STOPTRAFFIC-1	NCT04599140	Recruiting

The statistic is up to December 2022. For each agent, only one representative clinical trial is listed. All ongoing clinical trials for each agent are in Supplementary Tables 5 and 6. The therapeutic combination described in the representative trial is a simplified summary. For each drug, the cases of all kinds of combinations of the drug and other agents in one or several cohorts in a multi-cohort study, or the combination of the drug and other agents in a mono-cohort study are not separately described

*FDA* Food and Drug Administration, *RCC* renal cell carcinoma, *TKI* tyrosine kinase inhibitors, *mAb* monoclonal antibody, *MM* multiple myeloma, *IMiD* immunomodulatory drug, *NSCLC* non-small cell lung cancer, *HNSCC* head and neck squamous cell carcinoma, *CRC* colorectal cancer, *PC* prostate cancer, *CRPC* castration-resistant prostate cancer, *NHL* non-Hodgkin lymphoma, *HCC* hepatocellular carcinoma, *UC* urothelial carcinoma, *CTCL* cutaneous T-cell lymphoma, *DLBCL* diffuse large B cell lymphoma, *MDS* myelodysplastic syndrome, *AML* acute myeloid leukemia, *FL* follicular lymphoma, *SCLC* small-cell lung cancer, *CML* chronic myeloid leukemia, *ADC* antibody-drug conjugate, *PDAC* pancreatic ductal adenocarcinoma, *ccRCC* clear cell renal cell carcinoma

### The mammalian bromodomain and extra-terminal family proteins (BET family proteins)

The BET family proteins (including BRD2, BRD3, BRD4, and BRDT) are all bromodomain-containing epigenetic modifiers, which have histone acetyltransferase activity. The main mechanisms supporting the development of inhibitors of these proteins are transcriptional activation of multiple pro-tumorigenic pathways^471,472^ (Fig. 4). Their inhibition also stimulates anti-tumor immunity at several steps of the cancer-immunity cycle, suggesting their combinations with existing immunotherapies may be beneficial. For example, inhibition of BRD4, the most studied and targeted BET protein, enhances antigen presentation via increasing MHC class I expression,^473^ converts TAMs towards the M1-like phenotype,^474^ and reduces the expression of immune checkpoints (PD-L1 expression on DCs, TAMs and cancer cells^474,475^ and CD47 expression on cancer cells^476^). In addition, BRD4 is required for the activation of senescence-associated secretory phenotype genes and downstream paracrine signaling, inducing immune surveillance of the premalignant senescent cells.^477^ Thus, combination of PD-1/PD-L1 blockade and BET inhibition might be synergistic; however, adding BET inhibitors to ICIs did not improve patient responses.^478–480^ Encouragingly, NEO2734, an orally active BET and p300/CBP dual inhibitor, causes apoptosis and immunogenic cell death of tumor cells^481^ and acts synergistically with anti-PD-L1 and anti-CTLA-4 treatment, outstripping another BET inhibitor.^482^ Considering the current evidence and the potential of BET proteins in cancer and immune-related diseases,^483^ the exploration of their impacts on anti-tumor immunity and the development of drugs targeting BETs worth more effort.

### Histone methylase polycomb repressive complex 2 (PRC2)

PRC2, which is formed when zeste homolog 2 (EZH2) associates with embryonic ectoderm development (EED) protein and SUZ12, is responsible for histone methylation mainly at histone 3 lysine 27 (H3K27). It has a broad impact on cancer immunity^484^ (Fig. 4). It mediates long-term transcriptional silencing of the MHC-I antigen processing pathway^485^ and represses CXCL9 and CXCL10 production by tumors, two critical chemokines for effector T-cell trafficking.^486,487^ The orchestrated immune modulation also includes higher MDSC infiltration, less NK cell-mediated killing and more Treg-mediated immune suppression.^484,488^ EZH2 inhibition could enhance the efficacy and overcome resistance to current immunotherapies.^488^ Tazemetostat, an inhibitor of EZH2, the main catalytic unit, demonstrated clinical activity in epithelioid sarcoma in a phase II trial (ORR: 15%, duration of response: not reached).^489^ It was approved by FDA for locally advanced or metastatic epithelioid sarcoma in 2020. EZH1, a paralog of EZH2, can also form functional PRC2 complexes as a compensatory mechanism for tumor cells to escape EZH2 inhibition.^490,491^ Therefore, co-inhibition of EZH2 and EZH1^492–494^ or EED inhibition^492,495^ could more completely inhibit the activity of PRC2, especially in the presence of innate or acquired resistance mutations in EZH2 and by addressing the potential compensatory mechanism of EZH1-driven tumor growth. SETD2, an upstream regulator of EZH2, can also be targeted to combat EZH2-high tumors.^496^ Future preclinical and clinical investigations may identify novel drug targets and formats, and will provide more insight into the value of PRC2 inhibition in cancer immunotherapy.

### Lysine-specific histone demethylase 1 (LSD1)

LSD1 inhibitors are widely applied in myeloid hematological malignancies as they promote the differentiation of myeloid cells via regulation of myeloid transcription factors GFI1 and PU.1.^497,498^ Regarding anti-tumor immunity, LSD1 undermines T cell-mediated cytotoxicity via promoting terminal differentiation of T cells^499,500^ (Fig. 4). Accordingly, LSD1 inhibition expands progenitor exhausted T cells with stem-like properties, thereby enhancing the efficacy of immunotherapy.^499,500^ LSD1 inhibition also increases antigen presentation mediated by MHC I complexes on cancer cells^485,501^ and decreases exosomal PD-L1.^502^ Although tranylcypromine-based flavin adenine dinucleotide (FAD) domain-binding irreversible inhibitors exert long-lasting inhibition on LSD1 and yield encouraging clinical results both in myeloid malignancies^503^ and solid tumors,^504^ they induce significant TRAEs due to their covalent binding to FAD domains contained in critical enzymes other than LSD1 and the ensuing off-target reactivity.^505^ This could be ameliorated using reversible LSD1 inhibitors. For example, minimal inhibition of the cytochrome P450 enzymes containing a FAD domain was reported using the reversible LSD1 inhibitor TACH101.^506,507^ Another two clinical stage reversible LSD1 inhibitors, seclidemstat^508^ and CC-90011,^509^ also show immune activation and efficacy in combination with ICIs. Selectively targeting nuclear LSD1 phosphorylated at serine 111 (nLSD1p) might also be a plausible therapeutic approach to tackle the safety issue.^510^ Similar to most other anti-cancer agents targeting immuno-epigenetic modifiers, LSD1 inhibitors are combined with PD-1/PD-L1 blockade in phase I/II trials with promising preliminary results.^511^

Targeting epigenetics faces problems related to broad specificity and pleiotropic activity. Discovered immunomodulatory effects of some existing epigenetic modulators might contrast their previously known antitumor functions. For example, although HDAC activity generally seems to impair anti-tumor immunity, the intrinsic HDAC activity of Tcf1 and Lef1 is crucial for maintaining CD8^+^ T cell identity.^512,513^ Protein arginine methyltransferase 5 (PRMT5), another promising immune-epigenetic target with its inhibitors undergoing clinical evaluation and producing clinical benefits,^514^ has been reported to help improve anti-tumor immunity in melanoma^515^ (Table 3). However, it is also required for survival, function, homeostasis, and differentiation of effector T cells including CD4^+^ Th cells and invariant NK T cells,^516,517^ and increased PD-L1 expression is induced by PRMT5 inhibition in tumor cells.^518,519^ Accordingly, genetic or pharmacological targeting of PRMT5 compromises T cell-mediated antitumor immunity. Therefore, more information on how epigenetic regulators regulate immunity seems necessary in order to develop successful combination therapies, and cell-specific and/or conditionally activated agents might help to tackle these problems. There are many other promising immuno-epigenetic targets and processes, such as histone phosphorylation, various forms of RNA modifications including the aforementioned m6A modification, and noncoding RNAs.^520,521^ More joint efforts involving the industry are required to explore their therapeutic potential and promote clinical translation.

## Cytokines

Cytokines are soluble regulators of various intercellular communications. They are particularly important to the immune system and have constantly been the focus of studies in immune-related diseases, including cancer.

### Immunostimulatory interleukins: structural design and gene therapy

**Fig. 5:**
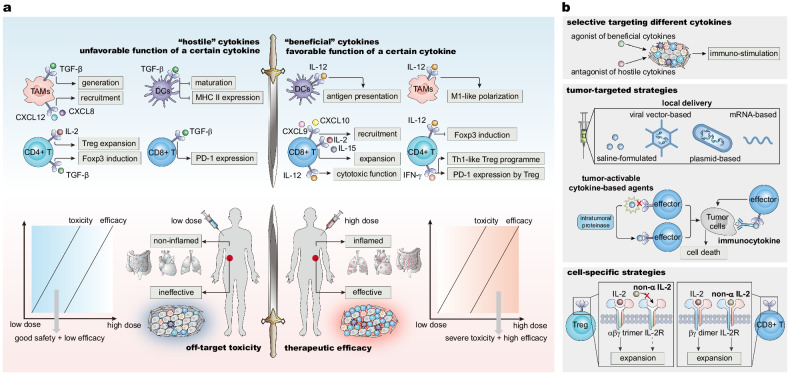
Two double-edged swords of cytokines and cytokine-based anti-tumor immunotherapy in cancer. **a** The upper panel illustrates the major functions of cytokines summarized in this review and highlights the pleiotropism of each cytokine and the cytokine family. On one hand, the broad spectrum of cytokines comprises both immunosuppressive and immunostimulatory cytokines; on the other hand, a certain cytokine may have both immunosuppressive and immunostimulatory impact toward different immune cell types concurrently. The bottom panel illustrates the importance of balancing between off-target toxicity and therapeutic efficacy when utilizing cytokine-based anti-tumor immunotherapy. **b** Current strategies of utilizing cytokines in cancer immunotherapy include: (1) stimulating immunostimulatory cytokines or antagonizing immunosuppressive cytokines, (2) confining the effects of cytokine-based agents within the TME using tumor-targeted approaches, and (3) targeting specific cell types to tackle pleiotropism. Selective tumor accumulation of cytokines could be achieved by local delivery methods and cytokine-based gene therapy, tumor-activatable agents, and immunocytokines. The bottom panel is an example of cell-specific strategies. Natural IL-2 binds to the αβγ trimer IL-2R expressed on Tregs and the βγ dimer IL-2R expressed on CD8^+^ T cells and induces their expansion concurrently; structurally modified non-α IL-2 variants prevent the binding to the α chain of IL-2R, thus avoiding expansion of immunosuppressive Tregs. TGF-β transforming growth factor-beta, IFN interferon, IL interleukin, Mφ macrophage, Th T helper cell, Foxp3 forkhead box protein P3, IL-2R interleukin-2 receptor

Deploying immunostimulatory cytokines, with an emphasis on interleukins, has been a research hotspot to activate both innate and adaptive anti-tumor immunity (Fig. 5a, b). IL-2 was the first cytokine discovered to promote T cell proliferation and expansion, and recombinant IL-2 (rIL-2) was the first immunotherapeutic that as monotherapy reproducibly induced durable, complete, and in some patients, curative regression of metastatic malignancies (melanoma and renal cancer).^189,190^ However, high-dose rIL-2 can cause severe life-threatening adverse effects such as capillary leakage, limiting its clinical application. Moreover, IL-2 is essential for immunosuppressive CD4^+^ Tregs. IL-2 can promote expansion of CD8^+^ T cells and of NK cells via binding to the intermediate-affinity dimeric IL-2Rβγ receptor without IL-2Rα (CD25),^522–524^ but expands Tregs via binding to the high-affinity trimeric IL-2R containing CD25,^525,526^ which is constitutively expressed on Tregs and transiently on recently activated nonregulatory T cells. The CD25-containing high-affinity receptor is also expressed on vascular endothelial cells and is involved in the capillary leakage mentioned above.^527^ During the last several years, development of IL-2-targeted agents had focused on non-α-binding IL-2 variants, which allow more selective activation of IL-2 signaling in CD8^+^ T and NK cells rather than CD4^+^ Tregs and vascular endothelial cells (Fig. 5b, Table 3 and Supplementary Table 6). However, though very promising in early trials,^528^ such agents showed suboptimal efficacy in recent trials. Bempegaldesleukin plus nivolumab had no added clinical efficacy versus nivolumab in two phase III trials, the PIVOT IO-001 study in melanoma^529^ and the PIVOT-09 trial in renal cell carcinoma,^530^ as well as in the phase II PIVOT-10 trial in urothelial cancer,^531^ which brought about the termination of other bempegaldesleukin trials. The reasons for this failure are unclear. However, it is likely that future developments will focus on agents that more selectively activate anti-tumor immunity, e.g., by targeting wild-type or mutant IL-2 to tumors or tumor-specific T cells, and that they will consider emerging knowledge of the effects of IL-2 and IL-2 variants on T cell exhaustion/differentiation. The current literature on the effects on T cell exhaustion appears controversial. One paper reported an unfavorable role of IL-2 inducing T cell exhaustion via activation of STAT5 and subsequently nucleus translocation of AhR.^532^ However, several recent papers reported less terminal exhaustion, and expansion of stem-like and effector-like T cells upon treatment with IL-2 or IL-2 variants.^379,533,534^ When recombinant wild-type IL-2 was combined with PD-1 blockade in the model of chronic lymphocytic choriomeningitis virus infection, even a deviation from the normal exhaustion program towards the formation of “better effectors” was observed and depended on IL-2 binding to CD25.^534,535^ Similar observations were made in tumor models using an IL-2Rβγ-biased IL-2 derivative fused to an anti-PD-1 antibody.^379^ The combination of the IL-2Rβγ-biased IL-2 variant nemvaleukin alfa with pembrolizumab, which received FDA fast track designation based on promising results from the ARTISTRY-1^536^ and ARTISTRY-2^537^ studies, might still produce clinical benefits in ongoing phase III testing. A plethora of other IL2-based agents and therapies may still have the potential to benefit cancer patients.^538^ As a natural “non-α IL-2 variant”, IL-15 mainly combines with the IL-15Rα subunit forming IL-15-IL-15Rα dimers on APCs and signals through IL-2/15Rβ (CD122)/cγ (CD132) on T cells or NK cells, with no binding to CD25^539^ (Table 3 and Supplementary Table 6). ALT-803, in which IL-15 and IL-15Rα subunits are precomplexed to mimic the in vivo form of APC-dependent dimerization, yielded a potent anti-tumor response (ORR: 29%, DCR: 76%) with nivolumab in anti-PD-1 r/r NSCLC patients,^540^ and similarly in a patient cohort with different ICI r/r solid tumors.^541^ Novel structure designs such as introducing tumor-targeted^542,543^ and/or conditionally activated (within the TME)^544,545^ moieties into cytokine-based agents can avoid systemic toxicity and achieve preferential tumor control and cell-biased binding properties (Fig. 5b). For example, fusing a non-α IL-2 mutein to an antibody against fibroblast activation protein-α (FAP) expressed on cancer-associated fibroblasts, such as simlukafusp alfa,^542^ or to an antibody against carcinoembryonic antigen often overexpressed by cancer cells, such as cergutuzumab amunaleukin,^543^ has achieved targeted expansion of CD8^+^ T cells at tumor sites in preclinical models and potentiated other T cell-stimulating immunotherapies. The first-in-human result of simlukafusp alfa seems promising,^546^ supporting further exploration of it especially in combination with ICIs. XTX202, an IL-2 mutein linked to an inactivation domain that could be cleaved by tumor proteases in the TME, induced potent tumor growth inhibition without systemic toxicity or peripheral immune activation in mouse models,^547^ and it is currently undergoing clinical evaluation (Table 3).

Apart from structurally altered derivatives, local administration of gene therapy may also help avoid toxicities associated with systemic administration and allow better control of the magnitude of the cytokine response (Fig. 5b). For example, the cytokine IL-12 has been reported to augment antigen presentation, tumor infiltration, activation, and function of CD8^+^ T cells, and the generation of M1-like macrophages and to suppress the expression PD-1 and Foxp3^548^ (Fig. 5a). The IL-12-encoding DNA plasmid tavokinogene telseplasmid resulted in robust tumor response (ORR: 36%, CRR: 18%) when electroporated into melanoma lesions in a phase II trial,^549^ and the efficacy was further augmented (ORR: 41%, CRR: 36%) by combining it with pembrolizumab.^550^ Intra-tumoral administration of saline-formulated^551^ or oncolytic nanoparticle-coated^552^ mRNAs encoding different anti-tumoral cytokines including IL-12 induced effective anti-tumor activity and potentiated the effects of ICIs in anti-PD-1-resistant tumors. The induced cytokine expression by gene therapy could be further controlled by orally available agents.^553^ Of note, these local delivery strategies can generate systemic anti-tumor immunity and immune memory, mediating regression of solid tumors at untreated sites and preventing tumor rechallenge.^549^

Many other immunostimulatory cytokines are also under clinical evaluation^554,555^ (Table 3). For instance, the modified IL-10 pegilodecakin enhanced response rates and durability of benefits, especially combined with PD-1 blockade in NSCLC,^556^ renal cell carcinoma (RCC),^557^ and melanoma^558^ patients, even in settings with unfavorable immunological features, such as no PD-L1 expression, low tumor mutational burden, presence of liver metastasis, and progression on prior checkpoint blockade.

### Transforming growth factor-β (TGF-β)

In addition to utilizing immunostimulatory cytokines and their agonists, antagonizing immunosuppressive ones can also augment anti-tumor immunity, as exemplified by agents targeting transforming growth factor-β (TGF-β) (Fig. 5a). After activation from latent TGF-β (L-TGF-β), TGF-β triggers the canonical TGF-β-Smad signaling and the non-canonical signaling crosstalking with other pathways such as the PI3K-AKT, ERK, and NF-κB pathways, which are considered pro-tumorigenic and immunosuppressive and are upregulated in advanced cancers.^559,560^ Regarding the initiation of the cancer-immunity cycle, TGF-β signaling hampers DC maturation, chemotaxis, and expression of key components of the antigen-presenting machinery.^561–566^Regarding the effector phase, the proliferation and tumor infiltration of CD8^+^ T cells are suppressed^567,568^; it also suppresses CD8^+^ T cell cytotoxicity via inhibition of TCR signaling and of T-bet and eomesodermin expression,^569^ two pivotal transcription factors controlling the CD8^+^ effector program.^570–572^ Generation of multiple immunosuppressive cell populations is promoted by TGF-β signaling, including Tregs,^573,574^ tumor-associated neutrophils,^575,576^ TAMs,^577^ and cancer-associated fibroblasts.^578,579^ Furthermore, it upregulates PD-L1 expression on TAMs^580^ and PD-1 mRNA in CD8^+^ T cells,^581^ warranting co-blockade of the PD-1/PD-L1 axis and TGF-β signaling. Pan-TGF-β mAb NIS793 is the only anti-TGF-β mAb still in phase III trials. It showed a favorable efficacy and safety profile in phase I exploration and received FDA orphan drug designation for pancreatic cancer,^582,583^ with phase II and III studies ongoing (Table 3 and Supplementary Table 6). However, many anti-TGF-β mAbs and small-molecule receptor kinase inhibitors have failed to demonstrate expected clinical benefits,^584,585^ which may be explained by the spatial-temporal versatility of TGF-β signaling. For example, although TGF-β supports tumor growth in established tumors, it suppresses tumor development at the early stages, and abrogation of TGF-β signaling can result in cancerous transformation of healthy tissue.^586–588^ Besides, debates still exist on its inhibitory effects on Tregs.^589,590^ Similar to immunostimulatory interleukins, recent drug development efforts for inhibiting TGF-β focus on enabling tissue/cell-specific engagement. This mainly includes targeting specific TGF-β isoforms (TGF-β1/2/3) and using bsAbs/msAbs to selectively inhibit TGF-β signaling in PD-1^+^CD8^+^ T cells, Tregs, or other cells within the TME. This could mitigate the adverse effects caused by the disruption of normal regulation of cardiovascular smooth muscles by TGF-β blockade, which made drug development stagnant for nearly two decades.^560^ SRK-181, a mAb targeting L-TGF-β1, has been promising as it alleviated cardiovascular adverse effects by selectively inhibiting activation of TGF-β1, and synergized with anti-PD-1 mAb.^591^ Similarly, TGF-β1/3 selective ligand trap AVID-200 elicited irAEs no greater than grade 3 with SD more than 12 weeks in 2 patients in a phase I trial (NCT03834662). Bintrafusp alfa, a bifunctional fusion protein enabling the colocalized and simultaneous blockade of TGF-β and PD-L1 and the consequent immunostimulatory effects as well as the preferential accumulation at the tumor site,^592,593^ outperforms either a TGF-β trap or a PD-L1 mAb in mouse models^594^ and shows signs of efficacy in early clinical trials in patients with various types of solid tumors.^595–600^ Unfortunately, the phase III INTR@PID Lung 037 study testing it in comparison with pembrolizumab as a first-line treatment in patients with PD-L1^+^ advanced NSCLC has been terminated due to its unlikeliness to reach the primary endpoint,^398^ similar to what is observed for the phase II INTR@PID BTC 055 and 047 trials testing its combination with chemotherapy as first-line and second-line treatment for biliary tract cancer.^601,602^ Other clinical trials testing the Bintrafusp alfa-based combinations in the INTR@PID program are ongoing^603^ and the clinical performance of other agents targeting the dual inhibition of PD-L1 and TGF-β are promising.^593^

### Chemokines

Beyond interleukins and TGF-β, based on their instrumental role in leukocyte attraction, chemotactic cytokines (chemokines) are also exploited for therapeutic use.^604–606^ There is extensive literature documenting the role of chemokines in the generation and delivery of immune cells, but chemokines are also reported to regulate the phenotype and function of immune cells as well as their arrangement in the TME.^604–606^ The drug developmental interest shows an emphasis on several specific chemokine-receptor axes, including the CXCL8/CXCL5-CXCR2,^607–609^ CXCL12-CXCR4^610^ and CCL2-CCR2^611,612^ axes, which largely participate in attracting suppressive cells to tumor sites, such as TAMs and MDSCs. Unmasking of additional immunomodulatory effects, such as promoting PD-L1 expression on macrophages^613^ and tumor cells^614^ and facilitating T cell exclusion,^615^ further supports the development of chemokine-targeting therapeutics. Most of them are undergoing phase I/II clinical evaluation combined with other anti-tumor treatments, mostly PD-1/PD-L1 blockade (Table 3 and Supplementary Table 6). In the COMBAT study, small molecule CXCR4 inhibitor motixafortide improved patient response and OS in metastatic PDAC patients in combination with pembrolizumab.^616^ Mavorixafor, another CXCR4 inhibitor, sensitized patients with advanced RCC to nivolumab.^617^ Further investigations into the mechanisms underlying the multifarious chemokine axes as well as the development of chemokine-based immunotherapies are expected.

The development of novel cytokine derivatives refined by protein engineering and modifications to enhance their pharmacokinetic/pharmacodynamic properties, such as Fc fusions, PEGylation, and ‘masked’ cytokines, is where important advances are being made, which may pave the way for future developments.^618,619^ Substantial progress can be made in enhancing the safety and efficacy of cytokine-based therapeutics with these emerging principles. In addition to the tremendous efforts devoted to the pharmacological development, research in recent years enables a more granular insight into cytokine biology with discoveries on novel immunological roles of both popular and less-studied cytokines - a wide research space to explore.

## Conclusions and perspectives

In-depth understanding of cancer immunobiology mechanisms and the progress in drug development platforms have resulted in a surge in the number of promising immunoregulatory targets, newly developed drugs and drug candidates, and related clinical trials. Identifying the most promising targets and drugs, and the most important challenges ahead are necessary for more efficient and specific future research and accelerating translation from basic research to patient benefits. Therefore, we have reviewed recent advances of mechanistic investigations and drug development for popular classes of immunomodulatory targets.

In the last few years, the development of immunoregulatory anti-cancer therapies has expanded from anti-PD-(L)1 and anti-CTLA-4 agents into several major areas as we discussed above. These next-generation immunotherapies, which target untapped pathways and/or utilize novel drug classes, are promising to benefit patients who are unresponsive to classical immunotherapies.

Since it has turned out that quite a large number of inhibitory and co-stimulatory immunoreceptors exist, as well as a number of agents targeting them are being developed, research focuses more and more on checkpoints other than PD-(L)1 and CTLA-4 to tackle resistance against classical ICIs. With the first approval of relatlimab, the LAG-3 checkpoint has gained considerable interest. Likewise, the phase III clinical trials of anti-TIGIT antibodies are attracting intensive attention, although final confirmation of their efficacy is still pending. The puzzles of mechanism-of-action of the various immune checkpoints are gradually being pieced together, and a detailed mechanistic clarification is needed to facilitate related clinical drug development. Biology and roles in anti-tumor immunity of some other inhibitory checkpoints, such as B7-H7/H long terminal repeat-associating 2 (HHLA2),^620–624^ leukocyte immunoglobulin-like receptors B family members,^625–627^ neuropilins and semaphorins,^628–630^ sialic-acid-binding immunoglobulin-like lectins (Siglecs),^631–633^ and butyrophilin family members,^634–638^ including their ligand-receptor interactions, have not yet been completely elucidated. Further studies are needed to evaluate the potential of these checkpoints in anti-cancer immunity. Meanwhile, clinical trials need to be conducted to validate their therapeutic potential as targets. The prospects of targeting co-stimulatory molecules remain uncertain, with terminated development of multiple agonistic antibodies due to lack of efficacy or too much toxicity. Encouragingly, recent advances in the further clarification of the mechanism-of-action of agonistic antibodies^288,301–304,639^ bring new research vitality to this field.

In addition to the expanding repertoire of targetable inhibitory and co-stimulatory molecules, the recent surge of bsAb/msAb development provides opportunities to enhance the safety and efficacy of agents targeting either conventional or novel molecules, based on the unique pharmacological properties of these novel drugs that go beyond the sum of their parts. The clinical development of several bsAbs for the treatment of hematological malignancies has progressed rapidly from phase III observations to their FDA approval due to induction of considerable CR rates. BsAbs/msAbs combining other immunoregulatory targets are in clinical trials, and many of them exhibit promising improvements in anti-tumoral responses. Notably, other novel drug types beyond bsAb/msAb and some new drug delivery platforms also facilitate the development of different kinds of immuno-modulatory therapeutics. Promising examples include engineered cytokine variants,^536,540^ nucleic acid-based delivery of cytokines,^551,553^ nanoparticles,^207,209,640^ cellular vesicles,^213^ and exosomes^641^ encapsulating antagonists/agonists of different immuno-modulatory pathways, and engineered bacteria.^642^ Thus, in addition to the biological discoveries of novel therapeutic targets and pathways, harnessing the full potential of these novel drug types and drug delivery platforms is also important for improving the efficacy and safety of cancer treatment.

Epigenetic therapy has been developed as anti-tumor therapy to tackle the epigenome dysregulation-driven cancer onset and progression.^400,401,643^ With the recent revelation of their immunoregulatory potential, there have also been lots of efforts to develop agents for epigenetic immunomodulation, particularly histone modifiers. In comparison to highly cell-specific ICIs, epigenetic therapies are context-dependent and pleiotropic, which enables them to orchestrate multiple components within the TME and target multiple steps in the tumor immune cycle at the same time, making them important combinatorial and adjuvant therapies to classical ICIs.^27,644^ Hopefully, optimal combination and sequencing of these agents with ICI-centered immunotherapy will overcome treatment resistance and improve treatment efficacy. Clinical trials exploring and comparing the sequential or simultaneous combination of these types of agents will likely be a major trend in the future. Besides, given the abundance of epigenetic drugs that have already been approved as anticancer therapeutics, it is likely that some of those can be repurposed for immunomodulation and combination with classical cancer immunotherapeutics. Novel immuno-epigenetic targets are also emerging, highlighted by RNA modifications via methylation, acetylation, uridylation and other modifications at different sites that have been well summarized.^645–647^ Although their biology and immunological effects have just been reported in animal studies, targeting these novel immuno-epigenetic processes might improve patient benefits which will require future studies exploring their therapeutic potential. However, being fundamental to every living cell, epigenetic processes may exert differential impacts on different immune cell types, and epigenetics-targeting agents encounter problems of insufficient specificity. To tackle this problem, both mechanistic explorations elucidating their immunological effects and efforts from the industry to improve their pharmacological properties are highly expected.

As one of the earliest immunotherapies, cytokine-based anti-cancer therapeutics have always received strong interest from the biotech and pharmaceutical industry. The large family of cytokines and the complex cytokine network play a crucial role in TME heterogeneity and the differentiation and functions of immunocytes, and this likely affects patient prognosis and responses to classical immunotherapies. Therefore, cytokine-based therapy offers substantial potential to overcome ICI resistance and considerable room for developing personalized, adaptable therapies tailored to various tumor immune subtypes of each patient. Similar to epigenetic therapy, cytokine-based therapies, based on their ability to regulate different components and steps of the anti-tumor immune response, also potentially synergize with ICIs and such combinations are being extensively tested in clinical trials. However, their varied roles in anti-tumor immunity across cell types, tissues, and concentrations, and between physiological and pathological conditions^555,648,649^ leads to an arduous efficacy-toxicity balance (Fig. 5a). Tissue-/cell-specific therapeutics and/or conditionally activated agents might help to overcome these problems. Both cytokine biology research and protein engineering and novel delivery platforms for cytokines have greatly advanced in recent years. They will hopefully help to design better drug structures and to expand the realm of targetable cytokines, continuously promoting the development of cytokine-based therapeutics.

It is worth noting that from a clinical perspective, differences exist in clinical practices for treatment of different cancer types. In fact, due to the varying immune backgrounds and intrinsic differences between cancer types, immunoregulatory anti-cancer therapies targeting different targets indeed have different optimal indications. Melanoma is well known for its robust immune responsiveness, which made it predestined for initial evaluation of therapeutic potential of LAG-3, TIM-3, CD40, and other immunoregulatory targets. Pembrolizumab induces CR in melanoma patients, and over 90% maintain CR for 5 years,^650^ highlighting potent efficacy of ICB. Relatlimab and tebentafusp were also approved for melanoma as their first indication. Activating the immune system against melanoma through cytokine-based therapies such as aldesleukin, darleukin, tavokinogene telseplasmid, has also proven to be effective.^529,651,652^

For other solid tumors, additional checkpoints like LAG-3 and TIGIT are likely to play significant roles. In the case of NSCLC, the relatively favorable immune environment in most NSCLC cases^653^ suggests that targeting these additional checkpoints could potentially be advantageous. LAG-3 agents have demonstrated efficacy in solid tumor entities such as NSCLC and HNSCC,^52–54^ and TIGIT agents are currently in several clinical trials in combination with PD-1/PD-L1 agents in NSCLC (Table 1). Exploration of B7 is ongoing across various solid tumors.^654^ The ADC enfortumab vedotin targeting nectin-4 has shown promising results particularly in combination with pembrolizumab in bladder cancer (NCT04223856). The situation is different for SCLC. While atezolizumab combined with carboplatin/etoposide is approved as first-line treatment for extensive-stage SCLC, many SCLC subtypes still respond weakly.^655,656^ These non-immunogenic tumor subtypes may rely on TAAs, such as DLL3 to be targeted, for example, with CD3×DLL3 TCE tarlatamab and HPN328. Neuroendocrine features of SCLC can also be managed with LSD1 inhibitors such as ladademstat to suppress neuroendocrine transcription factors.^656–658^

In gastrointestinal tumors, excellent efficacy for GC/GEJC is primarily observed with regimens based on anti-PD-1 agents and bsAbs containing anti-PD-1 scFv, such as cadonilimab^388^ and tebotelimab.^659^ For PDAC, CAFs are the main component of its TME, forming a strong physical barrier with the ECM that hampers T cell infiltration.^660^ CD40 agonists like sotigalimab can enhance T cell infiltration and show efficacy in combination with chemotherapy and nivolumab.^319^ Inhibiting TGF-β with NIS793 in combination with anti-PD-1 agents may help remodel the CAF-rich TME of PDAC.^578,579^ HCC is immune-privileged, with abundant MDSCs and an abnormal vascular system.^661^ Non-inflammatory HCC subtypes predominate,^661^ requiring anti-PD-1-based immunotherapy combined with anti-angiogenic therapy or dual immunotherapy to enhance immune response. Currently, atezolizumab plus bevacizumab is the first-line treatment for advanced HCC, with nivolumab plus ipilimumab and durvalumab plus tremelimumab also demonstrating efficacy.^662,663^ New bsAbs such as erfonrilimab and cadonilimab plus lenvatinib have achieved very high ORR.^205,393^ Additionally, direct targeting of HCC TAAs using bsAbs, such as CD3×GPC3 TCE ERY974, can be an effective approach.^664^

On the other hand, targeting TIM-3 and CD47 appears to be effective in hematological malignancies such as AML and MDS.^85,179,193,194^ Given that some types of cancer cells are themselves transformed immune cells, immuno-epigenetic agents can elicit effects via either immune or non-immune mediated mechanisms. Moreover, highly effective TCEs have shown remarkably high response rates in clinical trials, and reshaped the treatment for certain hematological malignancies with emerging new chemotherapy- free regimens.^665–668^

The TME of pediatric and nervous system tumors lacks TILs and shows poor expression of PD-(L)1, while TAMs, Tregs, and other immunosuppressive populations play crucial roles.^669,670^ Therefore, reshaping the suppressive TME and enhancing T cell infiltration are important. However, immunotherapy for nervous system tumors and non-hematological pediatric tumors is still in its early stages,^671,672^ with no immunotherapy yet proven to improve prognosis for gliomas.^672^ Adenovirus-encoded IL-12 INXN 2001 (Table 3 and Supplementary Table 6) may help ameliorate the suppressive microenvironment. CD155 serves as both the ligand for the inhibitory receptor TIGIT and the poliovirus receptor. The polio-rhinovirus chimera lerapolturev (Table 1 and Supplementary Table 1) offers some hope for treatment of gliomas.^673^ Redirecting anti-tumor immunity relying on TAAs is also an important strategy for immunotherapy of pediatric and nervous system tumors, with the mAbs or TCEs targeting B7-H3 and GD2 showing promise.^674,675^

For gynecologic tumors, each of the main cancer types presents distinct characteristics. Immunotherapy for endometrial cancer is mainly limited to the MSI-H/dMMR subgroup, represented by PD-1 agents such as pembrolizumab and dostarlimab,^676^ with the potential of other immunoregulatory therapies yet to be explored. Ovarian cancer exhibits a highly immunosuppressive TME and relatively low immunogenicity,^677^ resulting in poor response to immunotherapy. Combination therapies blocking multiple checkpoints, bsAb like ubamatamab or non-α IL-2 variant nemvaleukin alfa modulating the TME, can possibly enhance immune responses.^677–679^ Cervical cancer shows better responses to immunotherapy,^680^ with promising outcomes observed with pembrolizumab plus chemotherapy (recently approved by FDA), anti-PD-1 plus anti-CTLA-4 agents, and bsAbs.^680–682^

Beyond the drug therapies discussed in detail, other therapies, such as cancer gene therapy and cancer vaccines, are also promising anti-cancer treatments with immunoregulatory effects. While they may not be totally classified as conventional drug therapies, their rapid development and effectiveness are noteworthy. Cancer gene therapy can alter genes in vivo or ex vivo. Ex vivo gene therapy, represented by CAR T cell therapy, has achieved great clinical successes.^683–685^ Additional genetic modifications hold promise to further improve cell therapy, as manifested by the good safety and feasibility of CRISPR-edited TCR T cells and CAR T cells in patients with solid tumors.^686,687^ In-vivo gene therapy introduces the target gene directly into patients using a vector. Stimulating intra-tumoral cytokine gene expression (elaborated in the section on cytokines) and co-stimulatory molecules as well as inhibiting immunosuppressive molecules/cell types with anti-sense oligonucleotides (ASOs) and small interfering RNAs (siRNAs) attract high research interest. The combined local delivery of OX40L, CD80, and CD86 mRNAs cause significant local and systemic immune activation and facilitate tumor regression at both local and abscopal sites.^688^

Cancer vaccines amplify the signal of tumor-specific antigens (TSAs) or TAAs via encapsulated antigen-encoding DNA and RNA, peptides, or antigen-loaded APCs.^689–691^ They actively stimulate patients’ own anti-tumor immune response at the very beginning of the cancer immunity cycle. With promising potential,^692–696^ current research focuses on identifying antigens with the best quality and optimizing the delivery platform.^689–691^ High-throughput sequencing and bioinformatics tools in recent years have greatly facilitated the screening of highly immunogenic neoantigens.^692,697^ Improved vectors^698,699^ and immune adjuvants^700–702^ have been reported to enhance the efficacy of vaccine delivery and the ensuing immune activation and tumor-killing effects in preclinical studies. Combining cancer vaccines with ICIs and TME-reprogramming may help tackle the problem of immunosuppressive TME observed in classical immunotherapies, and is now being extensively tested in clinical trials with some producing promising results and advancing into phase III evaluations (NCT05141721, NCT06077760), but the optimal combination, dosage, and sequence of combination therapy still require further exploration. In the broader landscape of immunoregulatory cancer therapeutics, it is essential to recognize the contributions of these diverse approaches.

In summary, we have reviewed highly promising avenues for the development of immunoregulatory anti-cancer therapeutics by analyzing a large volume of recent published research, also including conference reports, and clinical trials. We summarized recent advances in the understanding of the mechanisms of action of classes of immunotherapy drug targets and the progress of the corresponding drug development. Despite considerable success so far, further research is necessary to boost drug development to improve treatment responses and prolong cancer patient survival. Moreover, next-generation drug development in these immunotherapy fields will continue to rely on clarification of immunological target biology and progress in drug developmental platforms, whereas the final evaluation of drug efficacy depends on rigorous high-quality clinical trials. This needs effective cooperation of academia, pharmaceutical and biotech industry, and the clinical medical community. More and more promising pharmacological immunoregulatory anti-cancer therapeutics are likely to be developed in innovative forms to the benefit of patients. This will further expand and enrich the landscape of immunoregulatory anti-cancer therapies.

### Supplementary information

Supplementary Table 1–6
