# Supplementary material for: Development of pharmacological immunoregulatory anti-cancer therapeutics: current mechanistic studies and clinical opportunities
Source: Signal Transduct Target Ther. 2024 May 22;9:126. doi: 10.1038/s41392-024-01826-z (PMC11109181; doi:10.1038/s41392-024-01826-z)
Supplement: Supplementary file 1 — Supplementary Table 1–6 [file 41392_2024_1826_MOESM1_ESM.docx]

Supplementary Materials for

Development of pharmacological immunoregulatory anti-cancer therapeutics:

current mechanistic studies and clinical opportunities

Nanhao Yin^1†^, Xintong Li^1†^, Xuanwei Zhang^1†^, Shaolong Xue^2^, Yu Cao^3,4^, Gabriele Niedermann^5*^, You Lu^1,6*^ and Jianxin Xue^1,6*^

Correspondence to: [radjianxin@163.com](mailto:radjianxin@163.com); [gabriele.niedermann@uniklinik-freiburg.de](mailto:gabriele.niedermann@uniklinik-freiburg.de); [radyoulu@163.com](mailto:radyoulu@163.com).

**This file includes:**

Supplementary Table 1. Therapeutics targeting additional inhibitory molecules, CD47, and B7 family proteins

Supplementary Table 2. Therapeutics targeting PD-1/PD-L1 and CTLA-4

Supplementary Table 3. Therapeutics targeting co-stimulatory molecules

Supplementary Table 4. Bi- and multi-specific antibodies

Supplementary Table 5. Therapeutics targeting immuo-epigenetics

Supplementary Table 6. Therapeutics targeting cytokines

**Supplementary Table 1. Therapeutics targeting additional inhibitory molecules, CD47, and B7 family proteins**

| Drug name | Manufacturer | Type of agent | Phase | Disease Area (Selected Indications) | Therapeutic combination | Identifier | |  | |  |
| --- | --- | --- | --- | --- | --- | --- | --- | --- | --- | --- |
|  |  |  |  |  |  | Trial number | Trial Name | Status | |  |
| ***Targeting LAG-3*** | | | | | | | | | |  |
| Relatlimab (BMS-986016) | Bristol-Myers Squibb | antagonistic mAb | III | Melanoma | anti-PD-1 (Nivolumab) | NCT03470922 | RELATIVITY-047 | Active, not recruiting | |  |
| Miptenalimab (BI 754111) | Boehringer Ingelheim | antagonistic mAb | II | Solid Tumors | anti-PD-1 (Ezabenlimab), VEGF/Ang2 inhibitor (BI 836880) | NCT03697304 |  | Recruiting | |  |
|  |  |  | I | Solid Tumors | anti-PD-1 (Ezabenlimab), MDM2 inhibitor | NCT03964233 |  | Recruiting | |  |
|  |  |  | I | NSCLC, HNSCC | anti-PD-1 (Ezabenlimab) | NCT03780725 |  | Terminated | |  |
|  |  |  | I | Solid Tumors | anti-PD-1 (Ezabenlimab) | NCT03433898 |  | Active, not recruiting | |  |
|  |  |  | I | Solid Tumors | anti-PD-1 (Ezabenlimab) | NCT03156114 |  | Active, not recruiting | |  |
| Favezelimab (MK4280) | Merck Sharp & Dohme | antagonistic mAb | I/II | SCLC | anti-PD-1 (Pembrolizumab) | NCT05064059 | MK-3475-B98/KEYNOTE-B98 | Recruiting | |  |
|  |  |  | III | CRC | anti-PD-1 (Pembrolizumab) | NCT04938817 | MK-4280A-007 | Not yet recruiting | |  |
|  |  |  | I/II | ccRCC | anti-PD-1 (Pembrolizumab) | NCT04626518 | MK-3475-03B | Recruiting | |  |
|  |  |  | I/II | ccRCC | anti-PD-1 (Pembrolizumab) | NCT04626479 | MK-3475-03A | Recruiting | |  |
|  |  |  | I/II | Hodgkin lymphoma, DLBCL, indolent non-Hodgkin lymphoma | anti-PD-1 (Pembrolizumab) | NCT03598608 | MK-4280-003 | Recruiting | |  |
|  |  |  | II | NSCLC | anti-PD-1 (Pembrolizumab) | NCT03516981 | MK-3475-495/KEYNOTE-495 | Recruiting | |  |
|  |  |  | I | Solid Tumors | anti-PD-1 (Pembrolizumab), chemotherapy | NCT02720068 | MK-4280-001 | Recruiting | |  |
| Fianlimab (REGN3767) | Regeneron | antagonistic mAb | I | Solid Tumors and lymphoma | anti-PD-1 (Cemiplimab) | NCT03005782 |  | Recruiting | |  |
|  |  |  | II | Breast Cancer | anti-PD-1 (Cemiplimab) | NCT01042379 | I-SPY | Recruiting | |  |
| Leramilimab (LAG525, IMP-701) | Immutep Limited, Novartis | antagonistic mAb | I | Breast Cancer | anti-PD-1 (Spartalizumab), A2aR antagonist (NIR178), MET inhibitor (Capmatinib), anti-CSF-1 (lacnotuzumab), anti-IL-1β (canakinumab) | NCT03742349 |  | Recruiting | |  |
|  |  |  | II | Breast Cancer | anti-PD-1 (Spartalizumab), chemotherapy | NCT03499899 |  | Active, not recruiting | |  |
|  |  |  | II | Melanoma | anti-PD-1 (Spartalizumab) | NCT03484923 | PLATforM | Recruiting | |  |
|  |  |  | II | Maliganancies | anti-PD-1 (Spartalizumab) | NCT03365791 |  | Completed | |  |
|  |  |  | I/II | Solid Tumors | anti-PD-1 (Spartalizumab) | NCT02460224 |  | Completed | |  |
| Encelimab (TSR-033) | AnaptysBio, GlaxoSmithKline | antagonistic mAb | I | Solid Tumors | anti-PD-1 (Dostarlimab), anti-TIM-3 (Cobolimab) | NCT02817633 |  | Recruiting | |  |
|  |  |  | I | Solid Tumors | anti-PD-1 (Dostarlimab), chemotherapy | NCT03250832 | CITRINO | Active, not recruiting | |  |
| Sym 022 | Symphogen | antagonistic mAb | I | Solid Tumors | anti-PD-1 (Sym021), anti-TIM-3 (Sym023) | NCT03311412 |  | Recruiting | |  |
|  |  |  | I | Solid Tumors |  | NCT03489369 |  | Completed | |  |
| IBI-110 | Innovent Biologics | antagonistic mAb | I | DLBCL | anti-PD-1 (Sintilimab) | NCT05039658 |  | Not yet recruiting | |  |
|  |  |  | I | Maliganancies | anti-PD-1 (Sintilimab) | NCT04085185 |  | Recruiting | |  |
|  |  |  | II | SCLC | anti-PD-1 (Sintilimab), chemotherapy | NCT05026593 |  | Recruiting | |  |
| INCAGN02385/INCAGN 2385 | Agenus | antagonistic mAb | I/II | Melanoma | anti-PD-1 (INCMGA00012), anti-TIM-3 (INCAGN02390) | NCT04370704 |  | Recruiting | |  |
|  |  |  | I | Solid Tumors and DLBCL | | NCT03538028 |  | Completed | |  |
| SHR-1802 | Jiangsu Hengrui | antagonistic mAb | I | Malignancies |  | NCT04414150 |  | Recruiting | |  |
| LBL-007 | Leads Biolabs | antagonistic mAb | I | Melanoma | anti-PD-1 (Toripalimab) | NCT04640545 |  | Recruiting | |  |
| Eftilagimod alpha (IMP 321) | Immutep/Merck & Co | LAG-3-Ig fusion protein | II | HNSCC | anti-PD-1 (Pembrolizumab) | NCT04811027 |  | Not yet recruiting | |  |
|  |  |  | I | Breast Cancer | chemotherapy | NCT04252768 | AIPAC-002 | Not yet recruiting | |  |
|  |  |  | II | NSCLC, HNSCC | anti-PD-1 (Pembrolizumab) | NCT03625323 | TACTI-002 | Recruiting | |  |
|  |  |  | I | Solid Tumors | anti-PD-L1 (Avelumab) | NCT03252938 |  | Recruiting | |  |
|  |  |  | I | melanoma | anti-PD-1 (Pembrolizumab) | NCT02676869 | TACTI-mel | Completed | |  |
|  |  |  | II | Breast Cancer | chemotherapy | NCT02614833 | AIPAC | Completed | |  |
| ***Targeting TIM-3*** | | | | | | | | | |  |
| Cobolimab (TSR-022) | AnaptysBio/Glaxo SmithKline | antagonistic mAb | I | Solid Tumors | ICOS agonist (Feladilimab), anti-PD-1 (Dostarlimab) | NCT02723955 | INDUCE-1 | Active, not recruiting |  |  |
|  |  |  | I | Solid Tumors | anti-PD-1 (Dostarlimab, Nivolumab), anti-LAG-3 (Encelimab), Chemotherapy | NCT02817633 | AMBER | Recruiting |  |  |
|  |  |  | II | NSCLC | anti-PD-1 (Dostarlimab), Chemotherapy | NCT04655976 | COSTAR Lung | Recruiting |  |  |
|  |  |  | II | Melanoma | anti-PD-1 (Dostarlimab) | NCT04139902 |  | Recruiting |  |  |
|  |  |  | I/II | NSCLC | ICOS agonist (Feladilimab), anti-PD-1 (Dostarlimab) | NCT03739710 |  | Recruiting |  |  |
|  |  |  | II | Liver Cancer | anti-PD-1 (Dostarlimab) | NCT03680508 |  | Recruiting |  |  |
|  |  |  | I | Malignancies | anti-PD-1 (Dostarlimab), Chemotherapy | NCT03307785 |  | Active, not recruiting |  |  |
| Sabatolimab (MBG-453) | Novartis | antagonistic mAb | I/II | Malignancies | anti-PD-1 (Spartalizumab) | NCT02608268 |  | Active, not recruiting |  |  |
|  |  |  | II | MDS |  | NCT04823624 |  | Not yet recruiting |  |  |
|  |  |  | II | MDS | Chemotherapy | NCT04878432 | STIMULUS MDS-US | Not yet recruiting |  |  |
|  |  |  | II | AML | Chemotherapy, Bcl2 inhibitor (Venetoclax) | NCT04150029 | STIMULUS-AML1 | Recruiting |  |  |
|  |  |  | III | MDS, CMML | Chemotherapy | NCT04266301 | STIMULUS-MDS2 | Recruiting |  |  |
|  |  |  | II | MDS | Chemotherapy | NCT03946670 | STIMULUS-MDS1 | Active, not recruiting |  |  |
|  |  |  | I | AML, High-risk MDS | p53-MDM2 inhibitor (Siremadlin) | NCT03940352 |  | Recruiting |  |  |
|  |  |  | I/II | AML | Chemotherapy | NCT04623216 |  | Recruiting |  |  |
|  |  |  | I | Hematological Malignancies | anti-PD-1 (Spartalizumab), Bcl2 inhibitor (Venetoclax) | NCT03066648 |  | Active, not recruiting |  |  |
|  |  |  | I | Glioblastoma Multiforme | anti-PD-1 (Spartalizumab) | NCT03961971 |  | Recruiting |  |  |
|  |  |  | II | MDS | Chemotherapy, Bcl2 inhibitor (Venetoclax) | NCT04812548 | STIMULUS-MDS3 | Recruiting |  |  |
|  |  |  | I | MDS | Anti-IL-1β (canakinumab), Anti-TGF-β (NIS793) | NCT04810611 |  | Recruiting |  |  |
|  |  |  | I/II | MF | JAK1/2 inhibitor (Ruxolitinib) | NCT04097821 | ADORE | Recruiting |  |  |
| INCAGN2390 | Agenus | antagonistic mAb | I/II | Melanoma | anti-PD-1 (Retifanlimab/INCMGA00012), anti-LAG-3 (INCAGN02385/INCAGN 2385) | NCT04370704 |  | Recruiting |  |  |
|  |  |  | I | Neoplasms |  | NCT03652077 |  | Active, not recruiting |  |  |
| BGB-A425 | BeiGene | antagonistic mAb | I/II | Solid Tumors | anti-PD-1 (Tislelizumab) | NCT03744468 |  | Recruiting |  |  |
| SHR-1702 | Jiangsu Hengrui | antagonistic mAb | I | AML, MDS |  | NCT04443751 |  | Recruiting |  |  |
|  |  |  | I | Solid Tumors | anti-PD-1 (Camrelizumab) | NCT03871855 |  | Not yet recruiting |  |  |
| LY3321367 | Eli Lilly and Company | antagonistic mAb | I | Solid Tumors | anti-PD-L1 (LY3300054) | NCT03099109 |  | Active, not recruiting |  |  |
|  |  |  | I | Solid Tumors | anti-PD-L1 (LY3300054) | NCT02791334 | PACT | Active, not recruiting |  |  |
| Sym023 | Symphogen | antagonistic mAb | I | Solid Tumors |  | NCT03489343 |  | Completed |  |  |
|  |  |  | I | BTC | anti-PD-1 (Sym021), anti-LAG-3 (INCAGN02385/INCAGN 2385) | NCT04641871 |  | Active, not recruiting |  |  |
|  |  |  | I | Solid Tumors and lymphoma | anti-PD-1 (Sym021), anti-LAG-3 (Sym022) | NCT03311412 |  | Recruiting |  |  |
| BMS-986258 | Bristol-Myers Squibb | antagonistic mAb | I/II | Solid Tumors | anti-PD-1 (Nivolumab), Recombinant human hyaluronidase (rHuPH20) | NCT03446040 |  | Recruiting |  |  |
| ***Targeting CEACAM-1*** | | | | | | | | |  |  |
| CM24 | Purple Biotech/Bristol-Myers Squibb | antagonistic mAb | I/II | Solid Tumors | anti-PD-1 (Nivolumab), chemotherapy | NCT04731467 |  | Recruiting |  |  |
| ***Targeting galecting-9*** | | | | | | | | |  |  |
| LYT-200 | PureTech | antagonistic mAb | I/II | Solid Tumors | anti-PD-1, chemotherapy | NCT04666688 |  | Recruiting |  |  |
| ***Targeting TIGIT*** | | | | | | | | |  |  |
| Vibostolimab (MK-7684) | Merck Sharp & Dohme | antagonistic mAb | I | Solid Tumors | anti-PD-1 (Pembrolizumab), Chemotherapy | NCT02964013 | MK-7684-001 | Recruiting | | |
|  |  |  | I/II | CRPC | anti-PD-1 (Pembrolizumab) | NCT02861573 | KEYNOTE-365/MK-3475-365 | Recruiting | | |
|  |  |  | II | Solid Tumors | anti-PD-1 (Pembrolizumab), multi-kinase inhibitor (Lenvatinib), Chemotherapy | NCT05007106 | MK-7684A-005 | Recruiting | | |
|  |  |  | II | Hematological Malignancies | anti-PD-1 (Pembrolizumab) | NCT05005442 | MK-7684A-004 | Recruiting | | |
|  |  |  | III | NSCLC | anti-PD-1 (Pembrolizumab) | NCT04738487 | MK-7684A-003 | Recruiting | | |
|  |  |  | II | NSCLC | anti-PD-1 (Pembrolizumab), Chemotherapy | NCT04725188 | MK-7684A-002 | Recruiting | | |
|  |  |  | I/II | Melanoma | anti-PD-1 (Pembrolizumab) | NCT04305054 | MK-3475-02B/KEYMAKER-U02 | Recruiting | | |
|  |  |  | I/II | Melanoma | anti-PD-1 (Pembrolizumab), anti-CTLA-4 (Quavonlimab) | NCT04305041 | MK-3475-02A/KEYMAKER-U02 | Recruiting | | |
|  |  |  | I/II | Melanoma | anti-PD-1 (Pembrolizumab) | NCT04303169 | MK-3475-02C/KEYMAKER-U02 | Recruiting | | |
|  |  |  | II | NSCLC | anti-PD-1 (Pembrolizumab), Chemotherapy | NCT04165070 | MK-3475-01A/KEYMAKER-U01A | Recruiting | | |
| Tiragolumab (MTIG-7192A) | Genentech, Roche | antagonistic mAb | III | NSCLC | anti-PD-1 (Atezolizumab) | NCT04294810 | SKYSCRAPER-01 | Recruiting | | |
|  |  |  | III | SCLC | anti-PD-1 (Atezolizumab), Chemotherapy | NCT04256421 | SKYSCRAPER-02 | Active, not recruiting | | |
|  |  |  | III | NSCLC | anti-PD-1 (Atezolizumab) | NCT04513925 | SKYSCRAPER-03 | Recruiting | | |
|  |  |  | III | SCLC | anti-PD-1 (Atezolizumab), Chemotherapy | NCT04665856 | SKYSCRAPER-02C | Active, not recruiting | | |
|  |  |  | III | ESCC | anti-PD-1 (Atezolizumab) | NCT04543617 | SKYSCRAPER-07 | Recruiting | | |
|  |  |  | III | EC | anti-PD-1 (Atezolizumab), Chemotherapy | NCT04540211 | SKYSCRAPER-08 | Recruiting | | |
|  |  |  | II | Cervical Cancer | anti-PD-1 (Atezolizumab) | NCT04300647 | SKYSCRAPER-04 | Active, not recruiting | | |
|  |  |  | II | NSCLC | anti-PD-1 (Atezolizumab), Chemotherapy | NCT04619797 | SKYSCRAPER-06 | Recruiting | | |
|  |  |  | II | HNSCC | anti-PD-1 (Atezolizumab) | NCT04665843 | SKYSCRAPER-09 | Recruiting | | |
|  |  |  | I/II | Melanoma | anti-PD-1 (Atezolizumab), PD-1 x LAG-3 bsAb (RO7247669) | NCT05116202 | MORPHEUS-Melanoma | Recruiting | | |
|  |  |  | I/II | Liver Cancer | anti-PD-1 (Atezolizumab), anti-VEGF (Bevacizumab) | NCT04524871 | MORPHEUS-Liver | Recruiting | | |
|  |  |  | I/II | UC | anti-PD-1 (Atezolizumab), Chemotherapy | NCT03869190 | MORPHEUS-UC | Recruiting | | |
|  |  |  | I/II | GC, EC, GEJC | anti-PD-1 (Atezolizumab), Chemotherapy | NCT03281369 | MORPHEUS-Gastric and Esophageal Cancer | Recruiting | | |
|  |  |  | I/II | Pancreatic Cancer | anti-PD-1 (Atezolizumab), Chemotherapy | NCT03193190 | MORPHEUS-Pancreatic Cancer | Active, not recruiting | | |
|  |  |  | II | NSCLC | anti-PD-1 (Atezolizumab), Radiation | NCT05034055 | SKYROCKET | Not yet recruiting | | |
|  |  |  | II | Solid Tumors | anti-PD-1 (Atezolizumab) | NCT04632992 | MyTACTIC | Recruiting | | |
|  |  |  | I | CRC | anti-PD-1 (Atezolizumab), anti-VEGF (Bevacizumab) | NCT04929223 | INTRINSIC | Recruiting | | |
|  |  |  | II | NSCLC | anti-PD-1 (Atezolizumab) | NCT03563716 | CITYSCAPE | Active, not recruiting | | |
|  |  |  | II | Melanoma | anti-PD-1 (Atezolizumab) | NCT05060003 |  | Not yet recruiting | | |
|  |  |  | II | Rectal Cancer | anti-PD-1 (Atezolizumab), Chemotherapy, Radiation | NCT05009069 |  | Not yet recruiting | | |
|  |  |  | II | NSCLC | anti-PD-1 (Atezolizumab), anti-VEGF (Bevacizumab) | NCT04958811 |  | Recruiting | | |
|  |  |  | II | GC, GEJC | anti-PD-1 (Atezolizumab), Chemotherapy | NCT04933227 |  | Recruiting | | |
|  |  |  | II | NSCLC | anti-PD-1 (Atezolizumab), Chemotherapy | NCT04832854 |  | Recruiting | | |
|  |  |  | II | SCLC | anti-PD-1 (Atezolizumab) | NCT04308785 |  | Recruiting | | |
|  |  |  | II | HNSCC | anti-PD-1 (Atezolizumab) | NCT03708224 |  | Recruiting | | |
|  |  |  | II | Melanoma | anti-PD-1 (Atezolizumab) | NCT03554083 |  | Recruiting | | |
|  |  |  | I | Solid Tumors | anti-PD-1 (Atezolizumab), Chemotherapy | NCT02794571 |  | Recruiting | | |
|  |  |  | I | TNBC | anti-PD-1 (Atezolizumab), Chemotherapy | NCT04584112 |  | Active, not recruiting | | |
|  |  |  | I | MM, NHL, B-Cell Lymphoma | anti-PD-1 (Atezolizumab), anti-CD20 (Rituximab), anti-CD38 (Daratumumab) | NCT04045028 |  | Recruiting | | |
| Ociperlimab (BGB-A1217) | BeiGene | antagonistic mAb | III | NSCLC | anti-PD-1 (Tislelizumab), Chemotherapy | NCT04866017 | AdvanTIG-301 | Recruiting | | |
|  |  |  | III | NSCLC | anti-PD-1 (Tislelizumab) | NCT04746924 | AdvanTIG-302 | Recruiting | | |
|  |  |  | II | BTC | anti-PD-1 (Tislelizumab), Chemotherapy | NCT05023109 |  | Not yet recruiting | | |
|  |  |  | II | ICC | anti-PD-1 (Tislelizumab), Chemotherapy | NCT05019677 |  | Not yet recruiting | | |
|  |  |  | II | SCLC | anti-PD-1 (Tislelizumab), Chemotherapy, Radiation | NCT04952597 |  | Recruiting | | |
|  |  |  | II | HCC | anti-PD-1 (Tislelizumab), anti-VEGF (BAT1706) | NCT04948697 |  | Recruiting | | |
|  |  |  | II | ESCC | anti-PD-1 (Tislelizumab) | NCT04732494 | AdvanTIG-203 | Recruiting | | |
|  |  |  | II | Cervical Cancer | anti-PD-1 (Tislelizumab) | NCT04693234 | AdvanTIG-202 | Active, not recruiting | | |
|  |  |  | II | NSCLC | anti-PD-1 (Tislelizumab) | NCT05014815 |  | Recruiting | | |
|  |  |  | I | Solid Tumors | anti-PD-1 (Tislelizumab), Chemotherapy | NCT04047862 | AdvanTIG-105 | Recruiting | | |
| Etigilimab (OMP-313M32) | Mereo BioPharma, OncoMed | antagonistic mAb | II | Fallopian Tube Cancer, Ovarian Cancer, Peritoneal Cancer | anti-PD-1 (Nivolumab) | NCT05026606 | EON | Recruiting | | |
|  |  |  | I/II | Solid Tumors | anti-PD-1 (Nivolumab) | NCT04761198 | ACTIVATE | Recruiting | | |
| BMS-986207 | Bristol-Myers Squibb, Compugen | antagonistic mAb | I/II | Solid Tumors | anti-PD-1 (Nivolumab), anti-CD112R (COM701) | NCT04570839 |  | Recruiting | | |
|  |  |  | I/II | Solid Tumors | anti-PD-1 (Nivolumab), anti-CTLA-4 (Ipilimumab) | NCT02913313 |  | Recruiting | | |
|  |  |  | II | NSCLC | anti-PD-1 (Nivolumab), anti-CTLA-4 (Ipilimumab) | NCT05005273 |  | Not yet recruiting | | |
|  |  |  | I/II | MM | Pomalidomide, Dexamethasone | NCT04150965 |  | Recruiting | | |
| Domvanalimab (AB154) | Arcus Biosciences | antagonistic mAb | II | Melanoma | anti-PD-1 (Zimberelimab/AB122) | NCT05130177 |  | Not yet recruiting | | |
|  |  |  | II | NSCLC | anti-PD-1 (Zimberelimab/AB122), A2aR/A2bR antagonist (Etrumadenant/AB928) | NCT04791839 |  | Recruiting | | |
|  |  |  | III | NSCLC | anti-PD-1 (Zimberelimab/AB122) | NCT04736173 | ARC-10 | Recruiting | | |
|  |  |  | I | Glioblastoma | anti-PD-1 (Zimberelimab/AB122) | NCT04656535 |  | Recruiting | | |
|  |  |  | II | NSCLC | anti-PD-1 (Zimberelimab/AB122), A2aR/A2bR antagonist (Etrumadenant/AB928) | NCT04262856 | ARC-7 | Recruiting | | |
|  |  |  | I | Solid Tumors | anti-PD-1 (Zimberelimab/AB122) | NCT03628677 |  | Recruiting | | |
| SEA-TGT (SGN-TGT) | Seattle Genetics, Seagen | antagonistic mAb | I | Solid Tumors and Hematological Maglinancies | anti-PD-1 (Sasanlimab) | NCT04254107 | SGNTGT-001 | Recruiting | | |
|  |  |  | I/II | NSCLC | anti-PD-1 (Sasanlimab), VEGFR1/2/3 inhibitor (Axitinib) | NCT04585815 |  | Recruiting | | |
| IBI-939 | Innovent Biologics | antagonistic mAb | I | NSCLC | anti-PD-1 (Sintilimab) | NCT04672369 |  | Not yet recruiting | | |
|  |  |  | I | Lung Cancer | anti-PD-1 (Sintilimab) | NCT04672356 |  | Recruiting | | |
|  |  |  | I | Malignancies | anti-PD-1 (Sintilimab) | NCT04353830 |  | Recruiting | | |
| JS006 | Shanghai Junshi | antagonistic mAb | I | Malignancies | anti-PD-1 (Toripalimab) | NCT05061628 |  | Recruiting | | |
| AK127 | Akeso Biopharma | antagonistic mAb | I | Solid Tumors | PD-1 x CTLA-4 bsAb (AK104) | NCT05021120 |  | Not yet recruiting | | |
| ***Targeting CD155*** | | | | | | | | | | |
| PVSRIPO (Lerapolturev) | Istari Oncology, Duke University Medical Center | recombinant polio virus (act by biology of CD155) | I/II | Solid Tumors | anti-PD-1/PD-L1 | NCT04690699 | LUMINOS-103 | Recruiting | | |
|  |  |  | II | Melanoma | anti-PD-1 | NCT04577807 | LUMINOS-102 | Recruiting | | |
|  |  |  | II | Glioblastoma | anti-PD-1 (Pembrolizumab) | NCT04479241 | LUMINOS-101 | Active, not recruiting | | |
|  |  |  | I | Melanoma |  | NCT03712358 |  | Active, not recruiting | | |
|  |  |  | I | Breast Cancer |  | NCT03564782 |  | Recruiting | | |
|  |  |  | I | Malignant Glioma |  | NCT03043391 |  | Active, not recruiting | | |
|  |  |  | II | Malignant Glioma |  | NCT02986178 |  | Active, not recruiting | | |
|  |  |  | I | Glioblastoma |  | NCT01491893 |  | Active, not recruiting | | |
| EOS884448 (EOS-448, GSK-4428859) | iTeos Therapeutics | antagonistic mAb | I/II | Solid Tumors | anti-PD-1 (Pembrolizumab), VEGFR1/2/3 inhibitor (Axitinib) | NCT05060432 | TIG-006 | Recruiting | | |
|  |  |  | I | Advanced Cancer |  | NCT04335253 |  | Active, not recruiting | | |
|  |  |  | II | NSCLC | anti-PD-1 (Dostarlimab) | NCT03739710 |  | Recruiting | | |
| ***Targeting CD96*** | | | | | | | | | | |
| GSK6097608 | GlaxoSmithKline | antagonistic mAb | I | Solid Tumors | anti-PD-1 (Dostarlimab) | NCT04446351 |  | Recruiting | | |
| ***Targeting CD112R*** | | | | | | | | | | |
| COM701 (CGEN-15029) | Compugen | antagonistic mAb | I | Solid Tumors | anti-PD-1(Nivolumab) | NCT03667716 |  | Recruiting | | |
|  |  |  | I/II | Solid Tumors | anti-PD-1(Nivolumab), anti-TIGIT (BMS-986207) | NCT04570839 |  | Recruiting | | |
|  |  |  | I | Solid Tumors | anti-TIGIT (COM-902) | NCT04354246 |  | Recruiting | | |
| ***Targeting Nectin-4*** | | | | | | | | | | |
| Enfortumab Vedotin | Astellas, Merck & Co, Seagen | ADC | III | MIBC | Anti- PD-L1 (Durvalumab), Anti-CTLA-4 (Tremelimumab) | NCT04960709 | VOLGA | Recruiting | | |
|  |  |  | III | Ureteral Cancer |  | NCT03474107 | EV-301 | Active, not recruiting | | |
|  |  |  | III | UC | anti-PD-1 (Pembrolizumab), chemotherapy | NCT04223856 | EV-302 | Recruiting | | |
|  |  |  | III | MIBC |  | NCT03924895 | MK-3475-905/KEYNOTE-905/EV-303 | Recruiting | | |
|  |  |  | III | MIBC | anti-PD-1 (Pembrolizumab) | NCT04700124 | MK-3475-B15/KEYNOTE-B15 /EV-304 | Recruiting | | |
| BT8009 | Bicycle Therapeutics | Bicycle toxin conjugate | I/II | Solid Tumors | anti-PD-1(Nivolumab) | NCT04561362 |  | Recruiting | | |
| ***Targeting CD47*** | | | | | | | | | | |
| Magrolimab (Hu5F9-G4) | Gilead | antagonistic mAb | I/II | UC | Atezolizumab | NCT03869190 | MORPHEUS-UC | Recruiting | | |
|  |  |  | III | AML | Azacitidine | NCT04778397 | ENHANCE-2 | Active, not recruiting | | |
|  |  |  | I/II | T-Cell Lymphoma | Mogamulizumab | NCT04541017 |  | Recruiting | | |
|  |  |  | I | Breast Cancer, CRPC | Olaparib | NCT05807126 | ELEVATE TNBC | Recruiting | | |
|  |  |  | II/III | TNBC | Nab-Paclitaxel, Paclitaxel, Sacituzumab Govitecan | NCT04958785 |  | Recruiting | | |
|  |  |  | II | HL | Pembrolizumab | NCT04788043 |  | Recruiting | | |
|  |  |  | I | NHL | Acalabrutinib, Rituximab | NCT03527147 | PRISM | Completed | | |
|  |  |  | I | AML, MDS | - | NCT02678338 | CAMELLIA | Completed | | |
|  |  |  | III | AML | Venetoclax, Azacitidine | NCT05079230 | ENHANCE-3 | Active, not recruiting | | |
|  |  |  | II | Myeloid Malignancies | Azacitidine, Venetoclax, Mitoxantrone, Etoposide, Cytarabine, CC-486 | NCT04778410 |  | Active, not recruiting | | |
|  |  |  | II | MM | Daratumumab, Pomalidomide, Dexamethasone, Bortezomib, Carfilzomib | NCT04892446 |  | Active, not recruiting | | |
|  |  |  | I | UC | Cisplatin, Gemcitabine | NCT05738161 |  | Recruiting | | |
|  |  |  | I/II | Solid Tumors | Cetuximab | NCT02953782 |  | Completed | | |
|  |  |  | I | Solid Tumors | - | NCT02216409 |  | Completed | | |
|  |  |  | II | HNSCC | Pembrolizumab, cetuximab, Docetaxel | NCT06046482 |  | Recruiting | | |
|  |  |  | I/II | B-cell NHL | Rituximab, Gemcitabine, Oxaliplatin | NCT02953509 |  | Active, not recruiting | | |
|  |  |  | I | Ovarian Cancer | Avelumab | NCT03558139 |  | Completed | | |
|  |  |  | I/II | AML | Azacitidine, Venetoclax | NCT04435691 |  | Active, not recruiting | | |
|  |  |  | II | CRC | Bevacizumab, Irinotecan, Fluorouracil, Leucovorin | NCT05330429 | ELEVATE CRC | Recruiting | | |
|  |  |  | I | B-cell Malignancies | Obinutuzumab, Venetoclax, Magrolimab | NCT04599634 |  | Recruiting | | |
|  |  |  | II | AML, MDS | intensive chemotherapy | NCT05829434 |  | Not yet recruiting | | |
|  |  |  | II | HNSCC | Pembrolizumab, chemotherapy, Zimberelimab | NCT04854499 |  | Recruiting | | |
|  |  |  | II | Solid Tumors | Docetaxel | NCT04827576 | ELEVATELung&UC | Active, not recruiting | | |
|  |  |  | I/II | AML, MDS | Sabatolimab, Azacitidine | NCT05367401 |  | Not yet recruiting | | |
|  |  |  | I | Brain Tumors | - | NCT05169944 | PNOC025 | Recruiting | | |
| Lemzoparlimab (TJ011133, TJC4) | I-Mab Biopharma | antagonistic mAb | I | Solid Tumors and lymphoma | Pembrolizumab, Rituximab | NCT03934814 |  | Completed | | |
|  |  |  | I | AML, MDS | Azacitidine, Venetoclax | NCT04912063 |  | Completed | | |
|  |  |  | I/II | AML, MDS | - | NCT04202003 |  | Active, not recruiting | | |
|  |  |  | III | MDS | Azacitidine (AZA) | NCT05709093 |  | Recruiting | | |
| Ligufalimab (AK117) | Akeso | antagonistic mAb | I/II | Malignancies | anti-PD-1/VEGF (AK112),Carboplatin,Cisplatin,5-Fluorouracil | NCT05229497 |  | Recruiting | | |
|  |  |  | II | MDS | Azacitidine | NCT06196203 |  | Not yet recruiting | | |
|  |  |  | I/II | AML | Azacitidine | NCT04980885 |  | Recruiting | | |
|  |  |  | II | CRC | anti-PD-1/VEGF (AK112), Oxaliplatin, Capecitabine, Irinotecan, Leucovorin,5-fluorouracil,Bevacizumab | NCT05382442 |  | Recruiting | | |
|  |  |  | I/II | Malignancies | anti-PD-1/CTLA-4 (AK104),Capecitabine,Oxaliplatin,Cisplatin,Paclitaxel,Irinotecan,Docetaxel,-FU | NCT05235542 |  | Recruiting | | |
|  |  |  | II | GC, GEJC | Cadonilimab, Oxaliplatin, Tegafur-gimeracil-oteracil potassium, Docetaxel, 5-Fluorouracil | NCT05960955 |  | Recruiting | | |
|  |  |  | I/II | Malignancies | anti-PD-1/VEGF (AK112), Chemotherapy | NCT05214482 |  | Recruiting | | |
|  |  |  | I/II | MDS | Azacitidine | NCT04900350 |  | Recruiting | | |
|  |  |  | II | TNBC | anti-PD-1/VEGF (AK112), Nab-paclitaxel, paclitaxel | NCT05227664 |  | Recruiting | | |
|  |  |  | I | Solid Tumors and lymphoma | - | NCT04728334 |  | Completed | | |
|  |  |  | I | Solid Tumors and lymphoma | anti-PD-1/CTLA-4 (AK104) | NCT04349969 |  | Completed | | |
| Letaplimab (IBI188) | Innovent Biologics (Suzhou) Co. Ltd. | antagonistic mAb | I | Malignancies | - | NCT03763149 |  | Completed | | |
|  |  |  | I | Malignancies | - | NCT03717103 |  | Completed | | |
| Urabrelimab (SRF231) | Surface Oncology | antagonistic mAb | I | Malignancies | - | NCT03512340 |  | Completed | | |
| ZL-1201 | Zai Lab (Shanghai) Co., Ltd. | antagonistic mAb | I | Advanced Cancer | - | NCT04257617 |  | Completed | | |
| AO-176 | Arch Oncology | antagonistic mAb | I/II | Ovarian Cancer | Paclitaxel, Pembrolizumab | NCT03834948 | KEYNOTE-C49 | Completed | | |
|  |  |  | I/II | MM | dexamethasone, bortezomib | NCT04445701 |  | Completed | | |
| STI-6643 | Sorrento Therapeutics, Inc. | antagonistic mAb | I | Solid Tumors | - | NCT04900519 |  | Recruiting | | |
| CC-90002 | Celgene | antagonistic mAb | I | Malignancies | Rituximab | NCT02367196 |  | Completed | | |
| Evorpacept (ALX148) | ALX Oncology Inc. | anti-CD47-Fc fusion protein | I/II | B-Cell NHL | Lenalidomide, Rituximab | NCT05025800 |  | Recruiting | | |
|  |  |  | II/III | GC, GEJC | Trastuzumab, Ramucirumab, Paclitaxel | NCT05002127 | ASPEN-06 | Recruiting | | |
|  |  |  | I/II | MDS | azacitidine | NCT04417517 | ASPEN-02 | Active, not recruiting | | |
|  |  |  | I | Breast Cancer | Fam-Trastuzumab Deruxtecan-Nxki, Zanidatamab, Tucatinib | NCT05868226 | PRE-I-SPY-PI | Recruiting | | |
|  |  |  | I | UC | Enfortumab Vedotin | NCT05524545 | ASPEN-07 | Recruiting | | |
|  |  |  | II | CRC | Cetuximab, Pembrolizumab | NCT05167409 |  | Active, not recruiting | | |
|  |  |  | I/II | HER2+ Cancers | Zanidatamab | NCT05027139 |  | Recruiting | | |
|  |  |  | II | HPVOPC | - | NCT05787639 |  | Not yet recruiting | | |
|  |  |  | II | HNSCC | Pembrolizumab, Cisplatin/Carboplatin; 5FU | NCT04675333 | ASPEN-04/KEYNOTE-B88 | Recruiting | | |
|  |  |  | I/II | Plasma Cell Myeloma | Isatuximab，Dexamethasone | NCT04643002 |  | Recruiting | | |
|  |  |  | II | HNSCC | Pembrolizumab | NCT04675294 | ASPEN-03/KEYNOTE-B87 | Recruiting | | |
|  |  |  | I | Solid Tumors and NHL | Pembrolizumab, Trastuzumab, Rituximab, Ramucirumab + Paclitaxel,5-FU + Cisplatin | NCT03013218 | ASPEN-01/ | Active, not recruiting | | |
|  |  |  | II | Ovarian Cancer | Pembrolizumab, Doxorubicin | NCT05467670 |  | Recruiting | | |
|  |  |  | I/II | AML | venetoclax, azacitidine | NCT04755244 | ASPEN-05 | Active, not recruiting | | |
| Maplirpacept (TTI-622, PF-07901801) | Pfizer | anti-CD47-Fc fusion protein | I/II | DLBCL | Tafasitamab, Lenalidomide | NCT05626322 |  | Recruiting | | |
|  |  |  | I/II | Ovarian Cancer, Fallopian Tube Cancer, Primary Peritoneal Carcinoma | Pegylated Liposomal Doxorubicin (PLD) | NCT05261490 |  | Active, not recruiting | | |
|  |  |  | I | Lymphoma and MM | - | NCT05567887 |  | Recruiting | | |
|  |  |  | I | MM | Daratumumab Hyaluronidase-fihj | NCT05139225 |  | Recruiting | | |
|  |  |  | I | MM | BCMA-CD3 bispecific antibody (Elranatamab) | NCT05675449 | MAGNETISMM-20 | Recruiting | | |
|  |  |  | I | AML, lymphoma, MM | Azacitidine, Venetoclax, Carfilzomib, Dexamethasone, Ruxience or Rituxan, Isatuximab | NCT03530683 |  | Active, not recruiting | | |
|  |  |  | II | DLBCL | Pembrolizumab | NCT05507541 |  | Recruiting | | |
|  |  |  | I | NHL and MM | - | NCT05896774 |  | Recruiting | | |
| Nibrozetone (RRx-001) | EpicentRx, Inc. | small molecule MYC and CD47 downregulator | I | Glioblastoma, oligodendroglioma | radiotherapy, temozolomide | NCT02871843 | G-FORCE-1 | Completed | | |
|  |  |  | I | Brain Tumors | Temozolomide, Irinotecan | NCT04525014 | PIRATE | Active, not recruiting | | |
|  |  |  | I | Brain Metastases | WBRT | NCT02215512 | BRAINSTORM | Completed | | |
|  |  |  | II | Oral Cancers | Cisplatin, Radiation Therapy | NCT03515538 | PREVLAR | Completed | | |
|  |  |  | I | Solid Tumors and lymphoma | Nivolumab | NCT02518958 | PRIMETIME | Completed | | |
|  |  |  | I | Solid Tumors and lymphoma | - | NCT01359982 | DINAMIC | Completed | | |
|  |  |  | II | Small Cell Carcinoma, NSCLC, Neuroendocrine Tumors, Ovarian Epithelial Cancer | Cisplatin,Etoposide,Carboplatin,Paclitaxel,Nab-Paclitaxel,Pemetrexed | NCT02489903 | QUADRUPLE THREAT | Completed | | |
|  |  |  | II | CRC | Irinotecan | NCT02096354 | ROCKET | Completed | | |
|  |  |  | II | HNSCC | Intensity Modulated Radiation Therapy (IMRT),Cisplatin | NCT05966194 | KEVLARx | Not yet recruiting | | |
|  |  |  | III | SCLC | Cisplatin/carboplatin plus etoposide | NCT05566041 | REPLATINUM | Active, not recruiting | | |
| ***Targeting SIRP-α*** | | | | | | | | | | |
| BI 765063 (OSE-172) | Boehringer Ingelheim | antagonistic mAb | I | HNSCC | Ezabenlimab,anti-EGFR/Ang2 (BI 836880),Cetuximab,Investigator's Choice Chemotherapy | NCT05249426 |  | Recruiting | | |
|  |  |  | I | Solid Tumors | anti-PD-1 (BI 754091) | NCT03990233 |  | Active, not recruiting | | |
|  |  |  | I | Solid Tumors | anti-PD-1 (BI 754091) | NCT04653142 |  | Completed | | |
|  |  |  | I | HNSCC, NSCLC | Ezabenlimab | NCT05068102 |  | Recruiting | | |
| Anzurstobart (CC-95251) | Celgene, Bristol-Myers Squibb | antagonistic mAb | I | Neoplasms | anti-CD20 (Rituximab), anti-EGFR (Cetuximab) | NCT03783403 | CC-95251-ST-001 | Active, not recruiting | | |
|  |  |  | I | AML, MDS | Azacitidine,Venetoclax | NCT05168202 | CA059-001 | Recruiting | | |
| Ontorpacept (TTI-621) | Pfizer | SIRPα-Fc fusion protein | I/II | Leiomyosarcoma | Doxorubicin | NCT04996004 |  | Active, not recruiting | | |
|  |  |  | II | DLBCL | Pembrolizumab | NCT05507541 |  | Recruiting | | |
| DSP107 | Kahr Medical | SIRPα-4-1BBL bifunctional fusion protein | I/II | Solid Tumors | atezolizumab | NCT04440735 |  | Recruiting | | |
|  |  |  | I | Hematological Malignancies | Azacitidine,Venetoclax | NCT04937166 |  | Recruiting | | |
| IMM01 | ImmuneOnco Biopharmaceuticals (Shanghai) Inc. | SIRPα-Fc fusion protein | I/II | AML, MDS | azacitidine | NCT05140811 |  | Recruiting | | |
|  |  |  | I/II | Solid Tumors and lymphoma | Tislelizumab | NCT05833984 |  | Recruiting | | |
| IMM0306 | ImmuneOnco Biopharmaceuticals (Shanghai) Inc. | SIRPα-anti-CD20 fusion protein | I/II | CD20+ B-cell NHL | - | NCT05805943 |  | Recruiting | | |
|  |  |  | I/II | B-cell NHL | Lenalidomide | NCT05771883 |  | Not yet recruiting | | |
| ***Targeting B7-H3*** | | | | | | | | | | |
| MGC018 | MacroGenics | ADC | I/II | Solid tumors | anti-PD-1 (Retifanlimab) | NCT03729596 |  | Recruiting | | |
| mirzotamab clezutoclax (ABBV-155) | AbbVie | ADC | I | Solid tumors | Chemotherapy | NCT03595059 |  | Recruiting | | |
| DS-7300a | Daiichi Sankyo | ADC | I/II | Solid tumors |  | NCT04145622 |  | Recruiting | | |
| ***Targeting B7-H4*** | | | | | | | | | | |
| Alsevalimab (FPA-150) | Amgen, Five Prime Therapeutics | antagonistic mAb | I | Solid tumors | anti-PD-1 (Pembrolizumab) | NCT03514121 | FPA150-001 | Completed | | |
| NC762 | NextCure | antagonistic mAb | I/II | Solid tumors |  | NCT04875806 |  | Recruiting | | |
| ***Targeting B7-H5*** | | | | | | | | | | |
| HMBD-002 | Hummingbird Bioscience | antagonistic mAb | I | Solid tumors | anti-PD-1 (Pembrolizumab) | NCT05082610 |  | Recruiting | | |
| Onvatilimab (JNJ-61610588, CI-8993, VSTB112) | Curis, Janssen, ImmuNext | antagonistic mAb | I | Solid tumors |  | NCT04475523 |  | Recruiting | | |
| CA-170 | Aurigene Discovery Technologies | small molecule inhibitor | II | Solid tumors and Lymphomas |  | CTRI/2017/12/011026 |  | Completed | | |
|  |  |  | I | Solid tumors and Lymphomas | NCT02812875 |  |  | Completed | | |

Annotation:

1. Cells filled with blue color for drug names indicate that the corresponding drug has been approved by the FDA.
2. For drugs approved by FDA, owing to its proven efficacy and the abundance of the clinical trials, only one representative trial is listed.

NSCLC non-small cell lung cancer, HNSCC head and neck squamous cell carcinoma, SCLC small-cell lung cancer, CRC colorectal cancer, ccRCC clear cell renal cell carcinoma, DLBCL diffuse large B cell lymphoma, MDS myelodysplastic syndrome, AML acute myeloid leukemia, CMML chronic myelomonocytic leukemia, MF myelofibrosis, BTC biliary tract cancer, CRPC castration-resistant prostate cancer, ESCC esophageal squamous-cell carcinoma, EC esophageal cancer, UC urothelial carcinoma, GC gastric cancer, GEJC gastroesophageal junction cancer, TNBC triple-negative breast cancer, MM multiple myeloma, ICC intrahepatic cholangiocarcinoma, HCC hepatocellular carcinoma, NHL non-Hodgkin lymphoma, HL Hodgkin lymphoma, HPVOPC human papillomavirus-positive oropharyngeal cancer, MIBC muscle-invasive bladder cancer.

**Supplementary Table 2. Therapeutics targeting PD-1/PD-L1 and CTLA-4**

| Drug name | Manufacturer | Type of agent | Phase | Disease Area (Selected Indications) | Therapeutic combination | Identifier | | Status |
| --- | --- | --- | --- | --- | --- | --- | --- | --- |
|  |  |  |  |  |  | Trial number | Trial Name |  |
| ***Targeting PD-1*** | | | | | | | | |
| Pembrolizumab | Merck | antagonistic mAb | I | Solid tumors | - | NCT01295827 | KEYNOTE-001 | Completed |
| Nivolumab | Bristol-Myers Squibb | antagonistic mAb | III | NSCLC | - | NCT01642004 | CheckMate 017 | Completed |
| Dostarlimab | GlaxoSmithKline | antagonistic mAb | I | Solid tumors | - | NCT02715284 | GARNET | Recruiting |
| Cemiplimab | Regeneron | antagonistic mAb | III | NSCLC | - | NCT03088540 | EMPOWER-Lung 1 | Active, not recruiting |
| Toripalimab (JS001) | Shanghai Junshi Biosciences | antagonistic mAb | III | NPC | - | NCT04376866 |  | Recruiting |
|  |  |  | III | RCC | VEGFR inhibitor (Axitinib) | NCT04394975 |  | Recruiting |
|  |  |  | III | HCC | Radiation | NCT04709380 |  | Recruiting |
|  |  |  | III | NSCLC | - | NCT04772287 |  | Not yet recruiting |
|  |  |  | III | NSCLC | chemotherapy | NCT04158440 |  | Recruiting |
|  |  |  | III | HCC | anti-VEGF (Bevacizumab) | NCT04723004 |  | Active, not recruiting |
|  |  |  | III | ESCC | - | NCT03829969 | JUPITER06 | Active, not recruiting |
|  |  |  | II/III | HCC | - | NCT03859128 | JUPITER 04 | Active, not recruiting |
|  |  |  | III | ICC | multi-kinase inhibitor (Lenvatinib), chemotherapy | NCT05342194 |  | Not yet recruiting |
|  |  |  | III | HCC | multi-kinase inhibitor (Lenvatinib) | NCT04523493 |  | Recruiting |
|  |  |  | III | NPC | Salvage surgery | NCT04778956 |  | Recruiting |
|  |  |  | III | Neuroendocrine Carcinoma | multi-kinase inhibitor (Surufatinib) | NCT05015621 |  | Recruiting |
|  |  |  | III | HCC | multi-kinase inhibitor (Lenvatinib), TACE | NCT05056337 |  | Enrolling by invitation |
|  |  |  | III | NPC | chemotherapy | NCT03581786 |  | Active, not recruiting |
|  |  |  | III | UC | chemotherapy | NCT04568304 |  | Not yet recruiting |
|  |  |  | III | NPC | chemotherapy, Radiation | NCT04453813 |  | Recruiting |
|  |  |  | III | NK/T Cell Lymphoma | chemotherapy, Radiation | NCT04365036 |  | Recruiting |
|  |  |  | II/III | ICC | chemotherapy | NCT04669496 |  | Recruiting |
|  |  |  | III | ESCC | chemotherapy | NCT04848753 |  | Recruiting |
|  |  |  | III | TNBC | chemotherapy | NCT04085276 | TORCHLIGHT | Recruiting |
|  |  |  | II/III | Neuroendocrine Carcinoma of the Bladder | chemotherapy | NCT03992911 |  | Recruiting |
|  |  |  | III | NPC | chemotherapy, Radiation | NCT04907370 | TIRA | Recruiting |
|  |  |  | III | Lynch Syndrome | - | NCT04711434 |  | Recruiting |
|  |  |  | II/III | Oral Squamous Cell Carcinoma | chemotherapy | NCT05125055 |  | Recruiting |
|  |  |  | III | UC | ADC (RC48-ADC) | NCT05302284 |  | Recruiting |
|  |  |  | IV | BC | anti-HER2 (Inetetamab), chemotherapy | NCT05291910 |  | Not yet recruiting |
|  |  |  | III | Gastric or Gastroesophageal Junction Adenocarcinoma | chemotherapy | NCT05180734 |  | Recruiting |
|  |  |  | III | EC | chemotherapy | NCT04280822 |  | Recruiting |
|  |  |  | III | NPC | chemotherapy, Radiation | NCT04557020 |  | Not yet recruiting |
|  |  |  | III | NPC | chemotherapy, Radiation | NCT05340491 |  | Recruiting |
|  |  |  | III | GC | chemotherapy, Radiation | NCT04997837 |  | Recruiting |
|  |  |  | III | NPC | chemotherapy, Radiation | NCT03907826 |  | Recruiting |
|  |  |  | II/III | NPC | chemotherapy | NCT04890522 |  | Not yet recruiting |
| Sintilimab (IBI308) | Eli Lilly /Innovent Biologics | antagonistic mAb | III | ESCC | - | NCT05495152 |  | Active, not recruiting |
|  |  |  | III | CRC | - | NCT04194359 |  | Recruiting |
|  |  |  | III | ESCC | chemotherapy, Radiation | NCT05244798 |  | Not yet recruiting |
|  |  |  | III | GC, GEJC | anti-VEGFR2 (Ramucirumab) | NCT04675983 | ORIENT-106 | Active, not recruiting |
|  |  |  | III | HCC | anti-VEGF (Bevacizumab) | NCT04682210 | DaDaLi | Not yet recruiting |
|  |  |  | III | ESCC | chemotherapy | NCT03748134 | ORIENT-15 | Recruiting |
|  |  |  | II/III | HCC | radiation (SBRT) | NCT04167293 | ISBRT01 | Recruiting |
|  |  |  | III | NPC | chemotherapy, radiation (IMRT) | NCT03700476 | CONTINUUM | Active, not recruiting |
|  |  |  | II/III | HCC | multi-kinase inhibitor (Lenvatinib), TACE/HAIC | NCT05250843 |  | Not yet recruiting |
|  |  |  | III | GC | chemotherapy | NCT03745170 | ORIENT-16 | Active, not recruiting |
|  |  |  | III | HCC | anti-CTLA-4 (IBI310) | NCT04720716 |  | Recruiting |
|  |  |  | II/III | RCC | VEGFR1/2/3 inhibitor (Fruquintinib) | NCT05522231 |  | Not yet recruiting |
|  |  |  | II/III | HCC | anti-VEGF (IBI305) | NCT03794440 | ORIENT-32 | Active, not recruiting |
|  |  |  | III | HL | chemotherapy | NCT04044222 | ORIENT-21 | Recruiting |
|  |  |  | II/III | GC | chemotherapy, radiation | NCT05002686 |  | Recruiting |
|  |  |  | II/III | Rectal Cancer | chemotherapy | NCT04304209 |  | Recruiting |
|  |  |  | III | NSCLC | chemotherapy | NCT03607539 | ORIENT-11 | Active, not recruiting |
|  |  |  | III | NSCLC | chemotherapy | NCT03629925 | ORIENT-12 | Active, not recruiting |
|  |  |  | III | NSCLC | chemotherapy | NCT05157776 |  | Recruiting |
|  |  |  | III | NSCLC | anti-VEGF (IBI305), chemotherapy | NCT03802240 | ORIENT-31 | Recruiting |
|  |  |  | III | CRC | chemotherapy | NCT05236972 |  | Recruiting |
|  |  |  | III | NSCLC | chemotherapy | NCT05116462 |  | Not yet recruiting |
|  |  |  | III | Anal Canal Cancer | chemotherapy, radiation | NCT05374252 |  | Recruiting |
|  |  |  | II/III | Rectal Cancer | chemotherapy, radiation | NCT05484024 |  | Not yet recruiting |
|  |  |  | II/III | Transitional Cell Tumor, Hepatoblastoma | multi-kinase inhibitor (Lenvatinib) | NCT05322187 | sPLENTY-pc | Not yet recruiting |
|  |  |  | III | ESCC | chemotherapy, radiation | NCT05357846 |  | Not yet recruiting |
|  |  |  | III | GC, GEJC | chemotherapy, radiation | NCT04997837 |  | Recruiting |
|  |  |  | III | HER2-positive Breast Cancer | anti-HER2 (Trastuzumab) | NCT05429684 |  | Recruiting |
|  |  |  | III | Acral Melanoma | anti-VEGF (IBI310) | NCT04277663 |  | Active, not recruiting |
|  |  |  | III | NSCLC | - | NCT03150875 |  | Active, not recruiting |
| Tislelizumab | BeiGene | antagonistic mAb | III | NPC | - | NCT05211232 |  | Not yet recruiting |
|  |  |  | III | NSCLC | chemotherapy | NCT03594747 |  | Active, not recruiting |
|  |  |  | III | DLBCL | stem cell transfer | NCT04799314 |  | Not yet recruiting |
|  |  |  | IV | NMIBC | Cancer vaccine (BCG) | NCT05580354 |  | Not yet recruiting |
|  |  |  | III | HL | - | NCT04486391 |  | Recruiting |
|  |  |  | III | DLBCL | stem cell transfer | NCT04789434 |  | Recruiting |
|  |  |  | III | HCC | multi-kinase inhibitor (Sitravatinib) | NCT05564338 |  | Not yet recruiting |
|  |  |  | III | NSCLC | chemotherapy | NCT03663205 | RATIONALE-304 | Active, not recruiting |
|  |  |  | III | NSCLC | anti-TIGIT (Ociperlimab) | NCT04746924 |  | Recruiting |
|  |  |  | III | NPC | chemotherapy | NCT03924986 | RATIONALE-309 | Active, not recruiting |
|  |  |  | III | NSCLC | multi-kinase inhibitor (Sitravatinib) | NCT04921358 |  | Recruiting |
|  |  |  | III | NSCLC | anti-TIGIT (Ociperlimab), anti-PD-L1 (Durvalumab), Chemotherapy | NCT04866017 |  | Recruiting |
|  |  |  | III | HNSCC | chemotherapy | NCT05582265 | REDUCTION-I | Not yet recruiting |
|  |  |  | III | UC | chemotherapy | NCT03967977 |  | Recruiting |
|  |  |  | III | SCLC | chemotherapy | NCT04005716 |  | Active, not recruiting |
|  |  |  | III | NSCLC | chemotherapy | NCT04379635 |  | Active, not recruiting |
|  |  |  | III | ESCC | chemotherapy | NCT03783442 |  | Active, not recruiting |
|  |  |  | III | GC, GEJC | chemotherapy | NCT03777657 | RATIONALE-305 | Active, not recruiting |
|  |  |  | III | ESCC | chemotherapy | NCT03430843 | RATIONALE-302 | Active, not recruiting |
|  |  |  | III | ESCC | chemotherapy, radiation | NCT03957590 | RATIONALE-311 | Active, not recruiting |
|  |  |  | III | HCC | - | NCT03412773 | RATIONALE-301 | Active, not recruiting |
|  |  |  | III | Solid tumors | PARP1/2 inhibitor (Pamiparib), chemotherapy | NCT04164199 |  | Enrolling by invitation |
|  |  |  | III | GC, GEJC | anti-HER2 (Zanidatamab), chemotherapy | NCT05152147 | HERIZON-GEA-01 | Recruiting |
|  |  |  | III | NSCLC | chemotherapy | NCT05346952 |  | Recruiting |
|  |  |  | III | B-cell Malignancies | BTK inhibitor (Zanubrutinib) | NCT04170283 |  | Enrolling by invitation |
|  |  |  | III | NPC | chemotherapy | NCT05342792 |  | Recruiting |
|  |  |  | III | NSCLC | - | NCT03358875 | RATIONALE-303 | Active, not recruiting |
| Camrelizumab (SHR-1210) | Jiangsu Hengrui | antagonistic mAb | III | HCC | VEGFR2 inhibitor (Apatinib) | NCT04639180 |  | Recruiting |
|  |  |  | III | Rectal Cancer | chemotherapy, radiation | NCT04928807 |  | Recruiting |
|  |  |  | III | NPC | chemotherapy, radiation (IMRT) | NCT05097209 |  | Not yet recruiting |
|  |  |  | III | HCC | HAIC, VEGFR2 inhibitor (Apatinib) | NCT05313282 | TRIPLET-III | Recruiting |
|  |  |  | III | NPC | Radiation (SBRT) | NCT04944914 |  | Recruiting |
|  |  |  | III | HL | chemotherapy | NCT04342936 |  | Recruiting |
|  |  |  | III | Cervical Cancer | multi-kinase inhibitor (Famitinib) | NCT04906993 |  | Enrolling by invitation |
|  |  |  | III | NSCLC | multi-kinase inhibitor (Famitinib) | NCT05042375 |  | Recruiting |
|  |  |  | III | HCC | VEGFR2 inhibitor (Apatinib) | NCT05198609 |  | Recruiting |
|  |  |  | III | GC, GEJC | VEGFR2 inhibitor (Apatinib) | NCT04342910 |  | Unknown status |
|  |  |  | III | TNBC | chemotherapy | NCT05134194 |  | Not yet recruiting |
|  |  |  | III | HCC | TACE, VEGFR2 inhibitor (Apatinib) | NCT05320692 |  | Recruiting |
|  |  |  | III | SCLC | VEGFR2 inhibitor (Apatinib), chemotherapy | NCT04490421 |  | Unknown status |
|  |  |  | III | NSCLC | chemotherapy, radiation (SRT/WBRT) | NCT04768075 |  | Not yet recruiting |
|  |  |  | III | NPC | chemotherapy | NCT03707509 | CAPTAIN-1st | Active, not recruiting |
|  |  |  | III | TNBC | chemotherapy | NCT04613674 |  | Recruiting |
|  |  |  | III | NSCLC | VEGFR2 inhibitor (Apatinib) | NCT04203485 |  | Unknown status |
|  |  |  | III | NSCLC | multi-kinase inhibitor (Famitinib) | NCT05106335 |  | Recruiting |
|  |  |  | III | Pancreatic Cancer | chemotherapy | NCT04674956 |  | Not yet recruiting |
|  |  |  | III | EC | chemotherapy, radiation | NCT04426955 |  | Active, not recruiting |
|  |  |  | III | NPC | chemotherapy, radiation | NCT04453826 |  | Recruiting |
|  |  |  | III | NPC | - | NCT03427827 | PACIFIC-NPC | Recruiting |
|  |  |  | III | EC | VEGFR2 inhibitor (Apatinib) | NCT05049681 |  | Not yet recruiting |
|  |  |  | III | NSCLC | multi-kinase inhibitor (Famitinib), chemotherapy | NCT04619433 |  | Recruiting |
|  |  |  | III | HCC | chemotherapy | NCT03605706 |  | Recruiting |
|  |  |  | III | EC | chemotherapy, radiation | NCT04404491 |  | Not yet recruiting |
|  |  |  | III | EC | chemotherapy | NCT03691090 |  | Unknown status |
|  |  |  | III | HCC | VEGFR2 inhibitor (Apatinib) | NCT03764293 |  | Active, not recruiting |
|  |  |  | III | GC, GEC | VEGFR2 inhibitor (Apatinib), chemotherapy | NCT03813784 |  | Active, not recruiting |
|  |  |  | III | NSCLC | - | NCT03668496 | CameL-Sq | Active, not recruiting |
|  |  |  | III | NSCLC | chemotherapy | NCT03134872 | CameL | Completed |
|  |  |  | III | EC | chemotherapy | NCT03099382 |  | Completed |
|  |  |  | III | TNBC | VEGFR2 inhibitor (Apatinib), chemotherapy | NCT04335006 |  | Recruiting |
| Pucotenlimab (HX008) | Taizhou Hanzhong Pharmaceuticals | antagonistic mAb | I | Solid Tumors | - | NCT04825392 |  | Recruiting |
|  |  |  | II | Melanoma | - | NCT04749485 |  | Active, not recruiting |
|  |  |  | II | Anaplastic Thyroid Cancer | - | NCT04574817 |  | Not yet recruiting |
|  |  |  | II | NMIBC | - | NCT04738630 |  | Recruiting |
|  |  |  | I/II | TNBC | chemotherapy | NCT04750382 |  | Active, not recruiting |
|  |  |  | II | Solid Tumors | - | NCT03704246 |  | Active, not recruiting |
|  |  |  | I/II | Melanoma | oncolytic viral therapy (OH2) | NCT04616443 |  | Recruiting |
|  |  |  | II | HCC | anti-VEGF (Bevacizumab), multi-kinase inhibitor (Lenvatinib) | NCT04741165 |  | Recruiting |
|  |  |  | III | GC | chemotherapy | NCT04486651 |  | Recruiting |
|  |  |  | I | Melanoma | anti-PD-L1 (LP002) | NCT04756934 |  | Recruiting |
|  |  |  | I/II | Solid Tumors | ADC (MRG002) | NCT05338957 |  | Recruiting |
|  |  |  | II/III | NSCLC | chemotherapy | NCT04750083 |  | Recruiting |
|  |  |  | II | Breast Cancer | PARP1/2 inhibitor (Niraparib) | NCT04508803 | CHANGEABLE | Recruiting |
|  |  |  | I/II | Solid Tumors | oncolytic viral therapy (OH2) | NCT03866525 |  | Recruiting |
|  |  |  | I | Melanoma | oncolytic viral therapy (OH2), radiation | NCT05068453 |  | Not yet recruiting |
|  |  |  | I | Melanoma | oncolytic viral therapy (OH2), multi-kinase inhibitor (Axitinib) | NCT05070221 |  | Not yet recruiting |
| Serplulimab (HLX10) | Shanghai Henlius | antagonistic mAb | II | HER2-positive GC | anti-HER2 (Trastuzumab), Chemotherapy | NCT05311189 |  | Not yet recruiting |
|  |  |  | II | Cervical Cancer | chemotherapy | NCT04150575 |  | Active, not recruiting |
|  |  |  | I | Solid Tumors | anti-DARP (HLX60) | NCT05483530 |  | Not yet recruiting |
|  |  |  | I | Solid Tumors | EGFR x 4-1BB bsAb (HLX35) | NCT05442996 |  | Not yet recruiting |
|  |  |  | III | SCLC | chemotherapy | NCT05468489 |  | Recruiting |
|  |  |  | I | Solid Tumors | anti-VEGF (HLX04) | NCT03757936 |  | Unknown status |
|  |  |  | II | SCLC | Radiation (hypofractionated radiotherapy, Prophylactic Cranial Irradiation) | NCT05443646 | ASTRUM-LC01 | Not yet recruiting |
|  |  |  | I | Solid Tumor | - | NCT03468751 |  | Unknown status |
|  |  |  | II | HNSCC | anti-EGFR (HLX07) | NCT04297995 |  | Active, not recruiting |
|  |  |  | II | MSI-H Solid Malignant Tumor | - | NCT03941574 |  | Recruiting |
|  |  |  | II | NSCLC | anti-EGFR (HLX07), chemotherapy | NCT04976647 |  | Recruiting |
|  |  |  | III | GC | - | NCT04139135 |  | Recruiting |
|  |  |  | III | SCLC | chemotherapy, Radiation (Thoracic radiotherapy, Prophylactic Cranial Irradiation) | NCT05353257 |  | Recruiting |
|  |  |  | II/III | CRC | anti-VEGF (HLX04), chemotherapy | NCT04547166 |  | Active, not recruiting |
|  |  |  | II | CRC | anti-LAG-3 (HLX26) | NCT05584137 |  | Not yet recruiting |
|  |  |  | III | TNBC | chemotherapy | NCT04301739 |  | Not yet recruiting |
|  |  |  | III | ESCC | - | NCT03958890 |  | Active, not recruiting |
|  |  |  | I | Solid Tumors | anti-LAG-3 (HLX26) | NCT05400265 |  | Recruiting |
|  |  |  | III | NSCLC | chemotherapy | NCT04033354 |  | Active, not recruiting |
|  |  |  | III | NSCLC | anti-VEGF (HLX04), chemotherapy | NCT03952403 |  | Recruiting |
|  |  |  | III | SCLC | chemotherapy | NCT04063163 |  | Active, not recruiting |
|  |  |  | II | HCC | anti-VEGF (HLX04) | NCT03973112 |  | Active, not recruiting |
|  |  |  | II | GC, GEJC | anti-EGFR (HLX07), chemotherapy | NCT05246982 |  | Recruiting |
|  |  |  | II | HCC | HAIC, anti-VEGF (HLX04) | NCT04947826 |  | Not yet recruiting |
|  |  |  | II | CRC | anti-EGFR (HLX07), chemotherapy | NCT05239650 |  | Not yet recruiting |
|  |  |  | II | SCLC | anti-EGFR (HLX07), chemotherapy | NCT05354700 |  | Not yet recruiting |
|  |  |  | II | NPC | anti-EGFR (HLX07), chemotherapy | NCT05513573 |  | Not yet recruiting |
|  |  |  | II | EC | anti-EGFR (HLX07), chemotherapy | NCT05221658 |  | Recruiting |
|  |  |  | II | HCC | anti-EGFR (HLX07), anti-VEGF (HLX04) | NCT05290220 |  | Not yet recruiting |
|  |  |  | I/II | Malignant Ascites | GM-CSF (Molgramostim), Radiation (Hypofractionated radiotherapy, SBRT) | NCT05501340 |  | Not yet recruiting |
|  |  |  | II | Cervical Cancer | anti-VEGF (Bevacizumab), chemotherapy | NCT05444374 |  | Not yet recruiting |
|  |  |  | II | GC, GEJC | multi-kinase inhibitor (Lenvatinib), chemotherapy | NCT05585580 |  | Not yet recruiting |
|  |  |  | III | Cervical Cancer | chemotherapy, Radiation (CCRT, Brachytherapy) | NCT05173272 |  | Not yet recruiting |
| Budigalimab (ABBV-181) | AbbVie | antagonistic mAb | I | Solid Tumors | anti-CD40 (ABBV-927) | NCT02988960 |  | Active, not recruiting |
|  |  |  | I | Solid Tumors | anti-GARP (ABBV-151) | NCT03821935 |  | Recruiting |
|  |  |  | I | Solid Tumors | anti-CD40 (ABBV-927), anti-OX40 (ABBV-368), chemotherapy | NCT03893955 |  | Recruiting |
|  |  |  | I | SCLC | ADC (ABBV-011) | NCT03639194 |  | Recruiting |
|  |  |  | I | Solid Tumors | Bcl2 inhibitor (Venetoclax), ADC (Rovalpituzumab) | NCT03000257 |  | Completed |
|  |  |  | I | Solid Tumors | anti-OX40 (ABBV-368) | NCT03071757 |  | Completed |
|  |  |  | I | Solid Tumors | anti-OX40 (ABBV-368), TLR9 agonist (Tilsotolimod), chemotherapy | NCT04196283 |  | Active, not recruiting |
|  |  |  | I | Solid Tumors | DLL4 x VEGF bsAb (ABT-165), chemotherapy | NCT01946074 |  | Completed |
|  |  |  | II | Pancreatic Cancer | anti-CD40 (ABBV-927), chemotherapy | NCT04807972 |  | Recruiting |
|  |  |  | I | Solid Tumors | ABBV-514 (undefined mechanism) | NCT05005403 |  | Recruiting |
|  |  |  | I | Solid Tumors | anti-CD39 (TTX-030), chemotherapy | NCT04306900 |  | Active, not recruiting |
| Retifanlimab (INCMGA00012) | Incyte Corporation/Macrogenics | antagonistic mAb | II | HNSCC | anti-LAG-3 (INCAGN02385), anti-TIM-3 (INCAGN02390) | NCT05287113 |  | Recruiting |
|  |  |  | I | HNSCC, GI cancer | anti-CD73 (INCA00186), anti-LAG-3 (INCB106385) | NCT04989387 |  | Recruiting |
|  |  |  | II | HNSCC | anti-GITR (Ragifilimab) | NCT05359692 |  | Recruiting |
|  |  |  | II | MCC | chemotherapy | NCT05594290 | MERCURY | Not yet recruiting |
|  |  |  | II | IDH-mutant Glioma | chemotherapy | NCT05345002 |  | Not yet recruiting |
|  |  |  | II | UC | anti-LAG-3 (INCAGN02385), anti-TIM-3 (INCAGN02390), IDO1 inhibitor (epacadostat) | NCT04586244 | Optimus | Recruiting |
|  |  |  | II | Pancreatic Cancer | - | NCT04116073 |  | Recruiting |
|  |  |  | II | Liposarcoma | CDK4/6 inhibitor (Palbociclib) | NCT04438824 |  | Recruiting |
|  |  |  | II | EC | - | NCT04494009 |  | Not yet recruiting |
|  |  |  | I | Solid Tumors | arginase inhibitor (INCB001158) | NCT03910530 | POD1UM-104 | Completed |
|  |  |  | II | Anal Canal Squamous Cell Carcinoma | - | NCT03597295 | POD1UM-202 | Completed |
|  |  |  | III | NSCLC | chemotherapy | NCT04205812 | POD1UM-304 | Recruiting |
|  |  |  | I | Solid Tumors | IDO1 inhibitor (Epacadostat), PI3Kδ inhibitor (INCB050465) | NCT03589651 |  | Active, not recruiting |
|  |  |  | II | MCC | - | NCT03599713 |  | Active, not recruiting |
|  |  |  | II | Penile Cancer | - | NCT04231981 | ORPHEUS | Active, not recruiting |
|  |  |  | II | NSCLC | - | NCT03679767 | POD1UM-203 | Completed |
|  |  |  | I | Solid Tumors | - | NCT03059823 |  | Active, not recruiting |
|  |  |  | I/II | Melanoma | anti-LAG-3 (INCAGN02385), anti-TIM-3 (INCAGN02390) | NCT04370704 |  | Recruiting |
|  |  |  | II | Endometrial Cancer | IDO1 inhibitor (epacadostat), FGFR1/2/3/4 inhibitor (pemigatinib), anti-LAG-3 (INCAGN02385), anti-TIM-3 (INCAGN02390) | NCT04463771 | POD1UM-204 | Recruiting |
|  |  |  | II | TNBC | oncolytic viral therapy (Pelareorep) | NCT04445844 | IRENE | Recruiting |
|  |  |  | I | Solid Tumors | AXL/MERTK inhibitor (INCB081776) | NCT03522142 |  | Recruiting |
|  |  |  | III | Anal Canal Squamous Cell Carcinoma | chemotherapy | NCT04472429 | POD1UM-303/InterAACT 2 | Recruiting |
|  |  |  | II | Glioblastoma | anti-GITR (INCAGN01876), radiation (SRS) | NCT04225039 |  | Active, not recruiting |
|  |  |  | II | Sarcoma | chemotherapy | NCT04968106 | TORNADO | Recruiting |
|  |  |  | I | Solid Tumors | A2a/A2b inhibitor (INCB106385) | NCT04580485 |  | Recruiting |
|  |  |  | II/III | GC, GEJC | anti-HER2 (margetuximab, Trastuzumab), LAG-3 x PD-1 bsAb (Tebotelimab), Chemotherapy | NCT04082364 | MAHOGANY | Active, not recruiting |
|  |  |  | II | Pancreatic Cancer | GSK-3β inhibitor (9-ING-41), chemotherapy | NCT05239182 | RiLEY | Recruiting |
|  |  |  | I | Glioblastoma Multiforme | chemotherapy, radiation | NCT05083754 |  | Recruiting |
|  |  |  | I/II | Soft Tissue Sarcoma | chemotherapy | NCT04577014 |  | Recruiting |
|  |  |  | I | Solid Tumors | anti-B7-H3 (Enoblituzumab) | NCT02475213 |  | Completed |
|  |  |  | II | Glioma, Glioblastoma | IDO1 inhibitor (Epacadostat), anti-VEGF (Bevacizumab), Radiation | NCT03532295 |  | Recruiting |
|  |  |  | I/II | CRC | gpA33 x CD3 DART (MGD007) | NCT03531632 |  | Completed |
|  |  |  | I/II | Solid Tumors | ADC (MGC018) | NCT03729596 |  | Active, not recruiting |
|  |  |  | I | Pancreatic Cancer, CRC | Cancer Vaccine (neoantigen-targeted), immunostimulant (Poly-ICLC) | NCT04799431 |  | Not yet recruiting |
|  |  |  | I/II | Breast Cancer | Cancer Vaccine (SV-BR-1-GM), chemotherapy, interferon | NCT03328026 |  | Recruiting |
|  |  |  | I | HNSCC | Cancer Vaccine (DPV-001), anti-GITR (INCAGN01876) | NCT04470024 |  | Recruiting |
|  |  |  | I | Solid Tumors | B7-H3 x CD3 DART (obrindatamab) | NCT03406949 |  | Completed |
|  |  |  | I/II | Solid Tumors | FGFR1/2/3/4 inhibitor (Pemigatinib) | NCT02393248 | FIGHT-101 | Completed |
|  |  |  | I | PDAC | MEK inhibitor (Trametinib), JAK inhibitor (Ruxolitinib) | NCT05440942 |  | Not yet recruiting |
|  |  |  | II | GC, EC | chemotherapy | NCT05177133 | AuspiCiOus | Recruiting |
|  |  |  | II | Malignancies | FGFR1/2/3/4 inhibitor (Pemigatinib) | NCT04949191 |  | Active, not recruiting |
|  |  |  | I/II | DLBCL | anti-CD19 (Tafasitamab), anti-CD20 (Rituximab), in Combination With Chemotherapy (Cyclophosphamide, Doxorubicin, Vincristine, and Prednisone) for the Treatment of Diffuse Large B-cell Lymphoma | NCT05455697 |  | Not yet recruiting |
| Ezabenlimab (BI 754091) | Boehringer Ingelheim | antagonistic mAb | I | Solid Tumors | oncolytic viral therapy (BI 1831169) | NCT05155332 |  | Recruiting |
|  |  |  | I | Solid Tumors | SIRPα inhibitor (BI 770371) | NCT05327946 |  | Recruiting |
|  |  |  | I | CRC | SIRPα inhibitor (BI 765063) | NCT05446129 |  | Recruiting |
|  |  |  | I | HNSCC, NSCLC, Melanoma | SIRPα inhibitor (BI 765063, BI 770371) | NCT05068102 |  | Recruiting |
|  |  |  | I | Solid Tumors | B7-H6 x CD3 bsAb (BI 765049) | NCT04752215 |  | Recruiting |
|  |  |  | I | Solid Tumors | SIRPα inhibitor (BI 765063) | NCT04653142 |  | Completed |
|  |  |  | I | Solid Tumors | - | NCT02952248 |  | Active, not recruiting |
|  |  |  | II | ESCC | EGFR/HER2 inhibitor (afatinib) | NCT04839471 | BEAR | Enrolling by invitation |
|  |  |  | I | Neoplasms | VEGF/ANG2 inhibitor (BI 836880) | NCT03972150 |  | Completed |
|  |  |  | I | Solid Tumors | SMAC mimetic (BI 891065) | NCT04138823 |  | Active, not recruiting |
|  |  |  | I | Solid Tumors | STING agonist (BI 1387446) | NCT04147234 |  | Recruiting |
|  |  |  | Early I | Neoplasms | anti-LAG-3 (BI 754111) | NCT03433898 |  | Active, not recruiting |
|  |  |  | I | Neoplasms, NSCLC | anti-LAG-3 (BI 754111) | NCT03156114 |  | Active, not recruiting |
|  |  |  | I | Solid Tumors | SIRPα inhibitor (BI 765063) | NCT03990233 |  | Recruiting |
|  |  |  | I/II | CRC | Cancer vaccine (ATP128, VSV-GP128) | NCT04046445 | KISIMA-01 | Recruiting |
|  |  |  | II | Solid Tumors | VEGF/ANG2 inhibitor (BI 836880), anti-LAG-3 (BI 754111) | NCT03697304 |  | Active, not recruiting |
|  |  |  | I | Solid Tumors | MDM2-p53 Antagonist (BI 907828), anti-LAG-3 (BI 754111) | NCT03964233 |  | Recruiting |
|  |  |  | I | HNSCC | SIRPα inhibitor (BI 765063), VEGF/ANG2 inhibitor (BI 836880), anti-EGFR (cetuximab), chemotherapy | NCT05249426 |  | Recruiting |
|  |  |  | I | NSCLC | VEGF/ANG2 inhibitor (BI 836880) | NCT03468426 |  | Active, not recruiting |
|  |  |  | II | Anus Squamous Cell Carcinoma | chemotherapy | NCT04719988 | INTERACT-ION | Recruiting |
|  |  |  | I | Solid Tumors | BI 1703880 (undefined mechanism) | NCT05471856 |  | Not yet recruiting |
|  |  |  | I | Solid Tumors | CD137/FAP agonist (BI 765179) | NCT04958239 |  | Recruiting |
|  |  |  | I | HER2-positive Glioblastoma | Cell therapy (NK-92/5.28.z) | NCT03383978 | CAR2BRAIN | Recruiting |
| Penpulimab (AK105) | Akeso Biopharma/Chia Tai Tianqing | antagonistic mAb | II | GC | chemotherapy | NCT05519202 |  | Not yet recruiting |
|  |  |  | II | NSCLC | multi-kinase inhibitor (Anlotinib) | NCT05460481 |  | Recruiting |
|  |  |  | II | SCLC | multi-kinase inhibitor (Anlotinib) | NCT05001971 |  | Recruiting |
|  |  |  | II/III | HCC | TACE, multi-kinase inhibitor (Anlotinib) | NCT05344924 |  | Not yet recruiting |
|  |  |  | II | DLBCL | IMiD (lenalidomide), anti-CD20 (Rituximab), chemotherapy | NCT05186558 |  | Not yet recruiting |
|  |  |  | IV | NSCLC | multi-kinase inhibitor (Anlotinib) | NCT05387109 | pcwaintrl | Not yet recruiting |
|  |  |  | II | ESCC | multi-kinase inhibitor (Anlotinib), chemotherapy | NCT05265962 |  | Not yet recruiting |
|  |  |  | II | Primary Central Nervous System Lymphoma | - | NCT05347641 |  | Not yet recruiting |
|  |  |  | II | Gynecological Cancer | multi-kinase inhibitor (Anlotinib) | NCT05028504 | ALTER-GO-020 | Recruiting |
|  |  |  | II | HNSCC | anti-EGFR (cetuximab) | NCT05260671 |  | Active, not recruiting |
|  |  |  | IV | Bladder Cancer | ADC (Disitamab) | NCT05488353 |  | Not yet recruiting |
|  |  |  | II | GC, GEJC | multi-kinase inhibitor (Anlotinib), chemotherapy | NCT05494060 | EXPLORING | Not yet recruiting |
|  |  |  | II | CRC | multi-kinase inhibitor (Anlotinib) | NCT04970914 | ALTER-C003 | Recruiting |
|  |  |  | II | NPC | Drug: TQB2618 Injection, Pempulimab Injection, Cisplatin Injection, Gemcitabine Hydrochloride Injection\|Drug: Penpulimab injection, Cisplatin Injection, Gemcitabine Hydrochloride Injection\|Drug: TQB2618 injection; Penpulimab injection | NCT05563480 |  | Not yet recruiting |
|  |  |  | III | NPC | - | NCT04974398 |  | Recruiting |
|  |  |  | III | HL | chemotherapy | NCT05244642 |  | Recruiting |
|  |  |  | II | Rectal Cancer | chemotherapy, Radiation (Short-course radiotherapy) | NCT05576480 | N-PRC | Not yet recruiting |
|  |  |  | II | CRC | multi-kinase inhibitor (Anlotinib), chemotherapy | NCT05229003 |  | Recruiting |
|  |  |  | II | NSCLC | multi-kinase inhibitor (Anlotinib), Radiation (SBRT) | NCT05485350 |  | Not yet recruiting |
|  |  |  | II | Pancreatic Cancer | multi-kinase inhibitor (Anlotinib), chemotherapy | NCT05493995 | PAAG | Recruiting |
|  |  |  | II | Thyroid Cancer | multi-kinase inhibitor (Anlotinib), radiation (Sodium Iodide I 131) | NCT04952493 |  | Recruiting |
|  |  |  | II | NSCLC | multi-kinase inhibitor (Anlotinib), chemotherapy | NCT04846634 | ALTER-L043 | Not yet recruiting |
|  |  |  | II | NPC | multi-kinase inhibitor (Anlotinib), chemotherapy | NCT04736810 |  | Recruiting |
|  |  |  | I/II | Lymphoma | anti-TIM-3 (TQB2618) | NCT05400876 |  | Recruiting |
|  |  |  | Not Applicable | GC | multi-kinase inhibitor (Anlotinib), chemotherapy | NCT05385900 | ICTCPTLAGC | Not yet recruiting |
|  |  |  | II | Cervical Cancer | multi-kinase inhibitor (Anlotinib) | NCT05137171 |  | Not yet recruiting |
|  |  |  | II | Breast Cancer | multi-kinase inhibitor (Anlotinib), chemotherapy | NCT05244993 |  | Not yet recruiting |
|  |  |  | II | HER2-positive GC | ADC (RC48), chemotherapy | NCT05313906 |  | Not yet recruiting |
|  |  |  | II | MGMT-Unmethylated Glioblastoma | multi-kinase inhibitor (Anlotinib), radiation | NCT05033587 |  | Recruiting |
|  |  |  | II | NSCLC | PD1 x VEGF bsAb (AK112), chemotherapy | NCT04736823 |  | Recruiting |
|  |  |  | I | Solid Tumors | protease inhibitor (TQB3602) | NCT05333276 |  | Not yet recruiting |
|  |  |  | I/II | HL | - | NCT03722147 |  | Unknown status |
|  |  |  | I/II | Solid Tumors | - | NCT04172506 |  | Completed |
|  |  |  | II | NPC | - | NCT03866967 |  | Active, not recruiting |
|  |  |  | I/II | Pancreatic Cancer | multi-kinase inhibitor (Anlotinib) | NCT04803851 |  | Not yet recruiting |
|  |  |  | II | Head and Neck Cancer, Chest Cancer | multi-kinase inhibitor (Anlotinib) | NCT04203719 |  | Unknown status |
|  |  |  | II | MSI-H or dMMR Solid Tumors | multi-kinase inhibitor (Anlotinib) | NCT04291248 |  | Not yet recruiting |
|  |  |  | II | GI Tumors, Urinary System Tumors, Neuroendocrine Tumors | multi-kinase inhibitor (Anlotinib) | NCT04207463 |  | Unknown status |
|  |  |  | I | Solid Tumors | - | NCT03352531 |  | Active, not recruiting |
|  |  |  | II | HCC | multi-kinase inhibitor (Anlotinib) | NCT04172571 | AK105-203 | Active, not recruiting |
|  |  |  | III | HCC | multi-kinase inhibitor (Anlotinib) | NCT04344158 |  | Not yet recruiting |
|  |  |  | III | NSCLC | chemotherapy | NCT03866993 |  | Unknown status |
|  |  |  | II | CRC | multi-kinase inhibitor (Anlotinib), chemotherapy | NCT05068206 |  | Recruiting |
|  |  |  | III | NSCLC | multi-kinase inhibitor (Anlotinib), chemotherapy | NCT03866980 |  | Active, not recruiting |
|  |  |  | III | GC, GEJC | multi-kinase inhibitor (Anlotinib) | NCT04385550 |  | Not yet recruiting |
| Spartalizumab (PDR001) | Novartis | antagonistic mAb | I | PDAC | anti-IL-1β (Canakinumab), chemotherapy | NCT04581343 | PanCAN-SR1 | Active, not recruiting |
|  |  |  | I | HNSCC | multi-kinase inhibitor (Ribociclib) | NCT04213404 | RISE-HN | Active, not recruiting |
|  |  |  | Early I | RCC | anti-IL-1β (Canakinumab) | NCT04028245 | SPARC-1 | Recruiting |
|  |  |  | II | Solid Tumors | - | NCT04802876 | ACROPOLI | Active, not recruiting |
|  |  |  | I/II | PDAC | anti-IL-6 (Siltuximab) | NCT04191421 |  | Recruiting |
|  |  |  | II | NSCLC | c-Met inhibitor (Capmatinib) | NCT04323436 |  | Active, not recruiting |
|  |  |  | I | Solid Tumors | SHP2 inhibitor (TNO155) | NCT04000529 |  | Recruiting |
|  |  |  | I | Solid Tumors, Lymphoma | IL-15 agonist (NIZ985), anti-PD-1 (Tislelizumab) | NCT04261439 |  | Recruiting |
|  |  |  | II | TNBC | anti-LAG-3 (LAG525), chemotherapy | NCT03499899 |  | Completed |
|  |  |  | II | NSCLC | c-Met inhibitor (Capmatinib) | NCT03647488 |  | Completed |
|  |  |  | II | PDAC | TGF-β inhibitor (NIS793), chemotherapy | NCT04390763 | daNIS-1 | Recruiting |
|  |  |  | II | GC | chemotherapy | NCT04736485 | GASPAR | Active, not recruiting |
|  |  |  | II | Oesophageal Adenocarcinoma | c-Met inhibitor (Capmatinib) | NCT05135845 | METIMGAST | Recruiting |
|  |  |  | I/II | Solid Tumors | multi-kinase inhibitor (Pazopanib) | NCT05210413 | SPARTO | Not yet recruiting |
|  |  |  | II | Melanoma | anti-LAG-3 (LAG525), anti-IL-1β (Canakinumab), c-Met inhibitor (Capmatinib), CDK4/6 inhibitor (Ribociclib) | NCT03484923 | PLATforM | Active, not recruiting |
|  |  |  | I | Glioblastoma Multiforme | anti-Tim-3 (MBG453), radiation (SRS) | NCT03961971 |  | Active, not recruiting |
|  |  |  | II | Cutaneous Melanoma | Braf inhibitor (Dabrafenib), MAP1/2 inhibitor (Trametinib) | NCT04310397 |  | Active, not recruiting |
|  |  |  | II | Anal Squamous Cell Carcinoma | chemotherapy, radiation | NCT04894370 | SPARTANA | Recruiting |
|  |  |  | I | Solid Tumors | - | NCT04058756 |  | Recruiting |
|  |  |  | II | NPC | - | NCT02605967 |  | Completed |
|  |  |  | I | CRC | multi-kinase inhibitor (regorafenib) | NCT03081494 |  | Completed |
|  |  |  | III | Melanoma | Braf inhibitor (Dabrafenib), MAP1/2 inhibitor (Trametinib) | NCT02967692 | COMBI-i | Active, not recruiting |
|  |  |  | I | RCC | Hif2α inhibitor (DFF332), A2aR inhibitor (NIR178) | NCT04895748 |  | Recruiting |
|  |  |  | I | TNBC | anti-LAG-3 (LAG525), anti-M-CSF (MCS110), anti-IL-1β (canakinumab), A2aR inhibitor (NIR178), c-Met inhibitor (capmatinib) | NCT03742349 |  | Active, not recruiting |
|  |  |  | Early I | Solid Tumors | KAZ954 (undefined mechanism), A2aR inhibitor (NIR178), anti-CD73 (NZV930) | NCT04237649 |  | Recruiting |
|  |  |  | I | Solid Tumors | IKZF2 inhibitor (DKY709) | NCT03891953 |  | Recruiting |
|  |  |  | II | Neuroendocrine Carcinoma | - | NCT02955069 |  | Completed |
|  |  |  | III | Pancreatic Cancer | anti-IL-1β (Canakinumab) | NCT04229004 |  | Recruiting |
|  |  |  | II | MDS, CML | anti-Tim-3 (Sabatolimab), chemotherapy | NCT05201066 |  | Not yet recruiting |
|  |  |  | I | Colorectal Cancer, Triple Negative Breast Cancer, NSCLC - Adenocarcinoma | anti-IL-1β (ACZ885), anti-IL-17A/F (CJM112), MAP1/2 inhibitor (TMT212), EGFR inhibitor (EGF816) | NCT02900664 |  | Completed |
|  |  |  | I | BRAF V600 Colorectal Cancer | Braf inhibitor (Dabrafenib), MAPK1/3 inhibitor (LTT462) | NCT04294160 |  | Recruiting |
|  |  |  | II | ESCC | - | NCT03785496 |  | Active, not recruiting |
|  |  |  | II | GC | anti-M-CSF (MCS110) | NCT03694977 |  | Unknown status |
|  |  |  | II | NSCLC | - | NCT03693326 |  | Unknown status |
|  |  |  | I | Malignancies | A2aR inhibitor (NIR178), anti-CD73 (NZV930) | NCT03549000 |  | Active, not recruiting |
|  |  |  | II | Malignancies | anti-Lag-3 (LAG525) | NCT03365791 |  | Completed |
|  |  |  | II | Solid Tumors, NHL | A2aR inhibitor (NIR178) | NCT03207867 |  | Active, not recruiting |
|  |  |  | I | MM | anti-IL-17A/F (CJM112) | NCT03111992 |  | Completed |
|  |  |  | I | Solid Tumors | TGF-β inhibitor (NIS793) | NCT02947165 |  | Completed |
|  |  |  | I | Solid Tumors | anti-PD-L1 (FAZ053) | NCT02936102 |  | Active, not recruiting |
|  |  |  | I/II | Solid Tumors | M-CSF inhibitor (BLZ945) | NCT02829723 |  | Active, not recruiting |
|  |  |  | I/II | Solid Tumors | anti-M-CSF (MCS110) | NCT02807844 |  | Completed |
|  |  |  | I/II | HCC | c-Met inhibitor (INC280) | NCT02795429 |  | Completed |
|  |  |  | I | Solid Tumors, Lymphoma | anti-GITR (GWN323) | NCT02740270 |  | Completed |
|  |  |  | I | Malignancies | - | NCT02678260 |  | Completed |
|  |  |  | I | Solid Tumors | pan-raf inhibitor (LXH254) | NCT02607813 |  | Completed |
|  |  |  | I/II | Solid Tumors | anti-LAG-3 (LAG525) | NCT02460224 |  | Completed |
|  |  |  | I | Solid Tumors | IL-15 agonist (NIZ985) | NCT02452268 |  | Completed |
|  |  |  | I/II | Solid Tumors | - | NCT02404441 |  | Completed |
|  |  |  | I | NSCLC | A2aR inhibitor (PBF-509) | NCT02403193 | AdenONCO | Completed |
|  |  |  | I/II | Solid Tumors | FGFR4 inhibitor (FGF401) | NCT02325739 |  | Completed |
|  |  |  | I/II | GI Stromal Tumors | multi-kinase inhibitor (Imatinib) | NCT03609424 |  | Recruiting |
|  |  |  | I | HCC | multi-kinase inhibitor (Sorafenib) | NCT02988440 |  | Completed |
|  |  |  | I | Solid Tumors | PORCUPINE inhibitor (LGK974) | NCT01351103 |  | Recruiting |
|  |  |  | II | Thyroid Cancer | Braf inhibitor (Dabrafenib), MAP1/2 inhibitor (Trametinib) | NCT04544111 |  | Recruiting |
|  |  |  | II | CRC | Braf inhibitor (Dabrafenib), MAP1/2 inhibitor (Trametinib) | NCT03668431 |  | Recruiting |
|  |  |  | I | AML, High-risk MDS | anti-TIM-3 (MBG453), chemotherapy | NCT03066648 |  | Active, not recruiting |
|  |  |  | I | Solid Tumors | IAP inhibitor (LCL161), mTOR inhibitor (Everolimus), HDAC inhibitor (Panobinostat) | NCT02890069 |  | Completed |
| Cetrelimab (JNJ-63723283) | Janssen | antagonistic mAb | II | MIBC | chemotherapy | NCT04919512 | SunRISe-4 | Recruiting |
|  |  |  | II | Small Cell Neuroendocrine PC | Hormone therapy (Apalutamide) | NCT04926181 |  | Recruiting |
|  |  |  | II | NMIBC | chemotherapy | NCT04640623 | SunRISe-1 | Recruiting |
|  |  |  | II | PC | PARP1/2 inhibitor (Niraparib), chemotherapy | NCT04592237 |  | Recruiting |
|  |  |  | III | MIBC | chemotherapy | NCT04658862 | SunRISe-2 | Recruiting |
|  |  |  | I | CRPC | hormone therapy (Apalutamide) | NCT03551782 |  | Completed |
|  |  |  | I/II | UC | FGFR inhibitor (Erdafitinib), chemotherapy | NCT03473743 | NORSE | Active, not recruiting |
|  |  |  | I/II | CRPC | PARP1/2 inhibitor (Niraparib) | NCT03431350 | QUEST | Active, not recruiting |
|  |  |  | I | Solid Tumors | FGFR inhibitor (Erdafitinib) | NCT03547037 |  | Completed |
|  |  |  | I/II | Neoplasms | - | NCT02908906 |  | Recruiting |
|  |  |  | II/III | MM | anti-CD38 (Daratumumab) | NCT03357952 |  | Completed |
| Balstilimab (AGEN2034) | Agenus | antagonistic mAb | II | Cervical Cancer | anti-CTLA-4 (Zalifrelimab) | NCT05033132 |  | Recruiting |
|  |  |  | II | Oropharyngeal Cancer | - | NCT05363709 |  | Not yet recruiting |
|  |  |  | II | CRC | anti-CTLA-4 (Botensilimab) | NCT05571293 | NEST-1 | Not yet recruiting |
|  |  |  | I/II | CRC | EP4 inhibitor (CR6086) | NCT05205330 |  | Recruiting |
|  |  |  | I | DIPG, DMG | Cancer vaccine (rHSC-DIPGVax), anti-CTLA-4 (Zalifrelimab) | NCT04943848 |  | Recruiting |
|  |  |  | I | Solid Tumors | anti-CTLA-4 (Botensilimab) | NCT03860272 |  | Recruiting |
|  |  |  | II | Melanoma | Oncolytic Viral Therapy (ONCOS-102) | NCT05561491 |  | Not yet recruiting |
|  |  |  | I | Solid Tumors | anti-ILT2 (AGEN1571), anti-CTLA-4 (Botensilimab) | NCT05377528 |  | Recruiting |
|  |  |  | II | Soft Tissue Sarcoma | anti-CTLA-4 (AGEN1884), chemotherapy | NCT04028063 |  | Active, not recruiting |
|  |  |  | I/II | Cervical Cancer | anti-CTLA-4 (AGEN1884) | NCT03495882 |  | Active, not recruiting |
|  |  |  | II | Cervical Cancer | anti-CTLA-4 (AGEN1884) | NCT03894215 |  | Recruiting |
|  |  |  | II | MIBC | anti-CTLA-4 (AGEN1884), chemotherapy | NCT04430036 |  | Active, not recruiting |
|  |  |  | I/II | Advanced Cancer\|Cervical Cancer | - | NCT03104699 |  | Active, not recruiting |
| Zimberelimab (GLS-010) | Arcus/Guangzhou Gloria/Taiho Pharmaceutical | antagonistic mAb | II | Cervical Cancer | chemotherapy, radiation | NCT05437692 |  | Recruiting |
|  |  |  | II | Melanoma | anti-TIGIT (Domvanalimab) | NCT05130177 |  | Recruiting |
|  |  |  | II | CRPC | anti-CD39 (SRF617), A2aR/A2bR inhibitor (etrumadenant) | NCT05177770 |  | Recruiting |
|  |  |  | II | NSCLC | anti-TIGIT (Domvanalimab), A2aR/A2bR inhibitor (etrumadenant) | NCT04791839 |  | Recruiting |
|  |  |  | II | GI Cancer | anti-TIGIT (Domvanalimab), chemotherapy | NCT05329766 |  | Recruiting |
|  |  |  | III | Advanced Upper Gastrointestinal Tract Adenocarcinoma | anti-TIGIT (Domvanalimab), chemotherapy | NCT05568095 |  | Not yet recruiting |
|  |  |  | II | UC | A2aR/A2bR inhibitor (etrumadenant), chemotherapy | NCT05335941 |  | Not yet recruiting |
|  |  |  | III | NSCLC | anti-TIGIT (Domvanalimab), chemotherapy | NCT05502237 | STAR-121 | Recruiting |
|  |  |  | I | Solid Tumors | anti-CCR8 (GS-1811) | NCT05007782 |  | Recruiting |
|  |  |  | I | Solid Tumors | - | NCT04087018 |  | Active, not recruiting |
|  |  |  | III | NSCLC | anti-TIGIT (Domvanalimab), chemotherapy | NCT04736173 | ARC-10 | Recruiting |
|  |  |  | II | NSCLC | anti-TIGIT (Domvanalimab), A2aR/A2bR inhibitor (etrumadenant) | NCT04262856 | ARC-7 | Recruiting |
|  |  |  | I | Solid Tumors | anti-TIGIT (Domvanalimab) | NCT03628677 |  | Active, not recruiting |
|  |  |  | I | Solid Tumors | A2aR/A2bR inhibitor (etrumadenant) | NCT03629756 |  | Completed |
|  |  |  | I | Pancreatic Cancer | anti-CD73 (AB680), chemotherapy | NCT04104672 | ARC-8 | Recruiting |
|  |  |  | I | HNSCC | A2aR/A2bR inhibitor (etrumadenant), chemotherapy, radiation | NCT04892875 | PANTHEoN | Not yet recruiting |
|  |  |  | I | Solid Tumors, Lymphoma | anti-TIGIT (AB308) | NCT04772989 | ARC-12 | Recruiting |
|  |  |  | I/II | Pancreatic Cancer | anti-TIGIT (DOMVANALIMAB), anti-CD40 (APX005M) | NCT05419479 |  | Not yet recruiting |
|  |  |  | I | NSCLC | A2aR/A2bR inhibitor (etrumadenant), chemotherapy | NCT03846310 | ARC-4 | Active, not recruiting |
|  |  |  | II | Rectal Cancer | A2aR/A2bR inhibitor (etrumadenant), chemotherapy, radiation | NCT05024097 | PANTHER | Recruiting |
|  |  |  | I/II | CRC | A2aR/A2bR inhibitor (etrumadenant), anti-VEGF (bevacizumab), chemotherapy | NCT04660812 | ARC-9 | Recruiting |
|  |  |  | I/II | CRPC | A2aR/A2bR inhibitor (Etrumadenant), (Quemliclustat), hormone therapy (Enzalutamide), chemotherapy | NCT04381832 | ARC-6 | Recruiting |
|  |  |  | II | HNSCC | anti-CD47 (Magrolimab), chemotherapy | NCT04854499 |  | Recruiting |
|  |  |  | II | UC | ADC (Sacituzumab Govitecan), chemotherapy | NCT03547973 | TROPHY U-01 | Recruiting |
|  |  |  | III | HL | - | NCT05518318 |  | Not yet recruiting |
|  |  |  | II | Cervical Cancer | - | NCT03972722 | CC | Recruiting |
|  |  |  | II | HL | - | NCT03655483 |  | Unknown status |
|  |  |  | I | Solid Tumors | - | NCT03713905 |  | Active, not recruiting |
|  |  |  | I | GC | multi-kinase inhibitor (Lenvatinib), chemotherapy | NCT05221775 |  | Recruiting |
|  |  |  | Not Applicable | Biliary Carcinoma | ADC (Disitamab Vedotin) | NCT05540483 | RIGHT | Recruiting |
| Geptanolimab (Genolimzumab, APL 501) | Apollomics | antagonistic mAb | I | CRC | VEGFR1/2/3 inhibitor (Fruquintinib) | NCT03977090 |  | Recruiting |
|  |  |  | I | NSCLC | VEGFR1/2/3 inhibitor (Fruquintinib) | NCT03976856 |  | Recruiting |
|  |  |  | II | Alveolar Soft Part Sarcoma | - | NCT03623581 |  | Recruiting |
|  |  |  | I | Solid Tumors | - | NCT03374007 | GB226 | Recruiting |
|  |  |  | II | Cervical Cancer | - | NCT03808857 |  | Recruiting |
|  |  |  | II | B-cell NHL | - | NCT03639181 |  | Recruiting |
|  |  |  | II | PTCL | - | NCT03502629 | Gxplore-002 | Recruiting |
|  |  |  | I | Solid Tumors | - | NCT03053466 |  | Completed |
| Prolgolimab (BCD-100) | Biocad | antagonistic mAb | II | Melanoma | - | NCT03269565 | MIRACULUM | Unknown status |
|  |  |  | I | Melanoma, Lung Cancer, Renal Cell Carcinoma | - | NCT03050047 |  | Unknown status |
|  |  |  | II | Cervical Cancer | anti-EGFR (Bevacizumab), chemotherapy | NCT03912402 |  | Unknown status |
|  |  |  | II/III | NSCLC | - | NCT03288870 | DOMINUS | Unknown status |
|  |  |  | III | NSCLC | chemotherapy | NCT03912389 | DOMAJOR | Recruiting |
|  |  |  | III | Cervical Cancer | anti-EGFR (Bevacizumab), chemotherapy | NCT03912415 | FERMATA | Recruiting |
|  |  |  | II | Melanoma | CTLA-4 x PD-1 (BCD-217) | NCT03913923 | OBERTON | Unknown status |
| Sasanlimab (PF-06801591) | Pfizer | antagonistic mAb | II | MIBC | radiation (SBRT) | NCT05241340 | RAD-VACCINE | Recruiting |
|  |  |  | I | Solid Tumors | HPK1 inhibitor (PF-07265028) | NCT05233436 |  | Recruiting |
|  |  |  | I/II | NSCLC | Braf inhibitor (Encorafenib), MAP1/2 inhibitor (Binimetinib), multi-kinase inhibitor (Axitinib), anti-TIGIT (SEA-TGT) | NCT04585815 | Landscape 1011 | Recruiting |
|  |  |  | III | NMIBC | Cancer vaccine (BCG) | NCT04165317 | CREST | Recruiting |
|  |  |  | I | Solid Tumors | PF-07263689 (undefined mechanism) | NCT05061537 |  | Active, not recruiting |
|  |  |  | I | Solid Tumors | anti-TIGIT (SEA-TGT) | NCT04254107 |  | Recruiting |
|  |  |  | I/II | Solid Tumors | - | NCT04181788 |  | Active, not recruiting |
|  |  |  | I | Solid Tumors | PF-07265807 (undefined mechanism), multi-kinase inhibitor (Axitinib) | NCT04458259 |  | Recruiting |
|  |  |  | I | Solid Tumors | - | NCT02573259 |  | Completed |
|  |  |  | I | Solid Tumors | PF-06940434 (undefined mechanism) | NCT04152018 |  | Recruiting |
|  |  |  | I | GI Tumors | PF-07062119 (undefined mechanism), Anti-VEGF (Bevacizumab) | NCT04171141 |  | Recruiting |
| Cosibelimab | Checkpoint Therapeutics/TG Therapeutics | antagonistic mAb | III | NSCLC | chemotherapy | NCT04786964 | CONTERNO | Active, not recruiting |
|  |  |  | I | Solid Tumors, Lymphoma | - | NCT03212404 |  | Recruiting |
| Pimivalimab (JTX-4014) | Celgene, Jounce Therapeutics | antagonistic mAb | I | Solid Tumors | - | NCT03790488 |  | Active, not recruiting |
|  |  |  | II | NSCLC | ICOS agonist (Vopratelimab) | NCT04549025 | SELECT | Active, not recruiting |
|  |  |  | I/II | Cancer | anti-LILRB2 (JTX-8064) | NCT04669899 |  | Recruiting |
| MEDI0680 (AMP514) | Amplimmune; AstraZeneca; MedImmune | antagonistic mAb | I | Malignancies | - | NCT02013804 |  | Completed |
|  |  |  | I/II | Malignancies | anti-PD-L1 (Durvalumab) | NCT02118337 |  | Completed |
|  |  |  | I/II | Aggressive B-cell Lymphoma | anti-CD19 (Inebilizumab) | NCT02271945 |  | Completed |
| Nofazinlimab (CS1003) | CStone Pharmaceuticals | antagonistic mAb | I | Solid Tumors, Lymphoma | - | NCT03809767 |  | Active, not recruiting |
|  |  |  | III | HCC | multi-kinase inhibitor (Lenvatinib) | NCT04194775 |  | Recruiting |
|  |  |  | I | Solid Tumors | multi-kinase inhibitor (Regorafenib) | NCT03475251 |  | Completed |
|  |  |  | I | Solid Tumors | anti-CDTLA-4 (CS1002) | NCT03523819 |  | Active, not recruiting |
| ***Targeting PD-L1*** | | | | | | | | |
| Atezolizumab | Roche, Genentech | antagonistic mAb | III | NSCLC | - | NCT02008227 | OAK | Completed |
| Durvalumab | Celgene, MedImmune | antagonistic mAb | III | NSCLC | - | NCT02125461 | PACIFIC | Active, not recruiting |
| Avelumab | Merck | antagonistic mAb | II | MCC | - | NCT02155647 | JAVELIN Merkel 200 | Active, not recruiting |
| Pacmilimab (CX-072) | CytomX Therapeutics | probody | I/II | Solid Tumors, Lymphoma | anti-CTLA-4 (ipilimumab), Braf inhibitor (vemurafenib) | NCT03013491 | PROCLAIM-CX-072 | Completed |
|  |  |  | II | Breast Cancer | ADC (CX-2009) | NCT04596150 |  | Active, not recruiting |
| Sugemalimab (CS1001) | CStone Pharmaceuticals, Bayer | antagonistic mAb | Expanded acess study | Extranodal NK/T-cell Lymphoma | - | NCT05131438 |  | Available |
|  |  |  | I | SCLC | - | NCT04421352 |  | Active, not recruiting |
|  |  |  | II | Extranodal NK/T-Cell Lymphoma | - | NCT03595657 | GEMSTONE-201 | Active, not recruiting |
|  |  |  | III | NSCLC | - | NCT03728556 | GEMSTONE-301 | Active, not recruiting |
|  |  |  | II | HL | - | NCT03505996 |  | Completed |
|  |  |  | I | Solid Tumors | - | NCT03312842 |  | Completed |
|  |  |  | III | NSCLC | - | NCT03789604 | GEMSTONE-302 | Active, not recruiting |
|  |  |  | I | Solid Tumors | - | NCT03744403 |  | Completed |
|  |  |  | III | EC | chemotherapy | NCT04187352 |  | Active, not recruiting |
|  |  |  | I | Solid Tumors | multi-kinase inhibitor (Donafenib) | NCT04472858 |  | Recruiting |
|  |  |  | I/II | Solid Tumors | multi-kinase inhibitor (Regorafenib) | NCT04200404 |  | Completed |
|  |  |  | III | GC, GEJC | chemotherapy | NCT03802591 |  | Active, not recruiting |
|  |  |  | I/II | HCC | FGFR4 inhibitor (Fisogatinib) | NCT04194801 |  | Unknown status |
| Opucolimab (HLX-20) | Henlix Biotech | antagonistic mAb | I | Solid Tumors | - | NCT03588650 |  | Completed |
| Envafolimab (KN035) | Alphamab | antagonistic mAb | II | NPC | chemotherapy, radiation | NCT05397769 |  | Recruiting |
|  |  |  | II | Endometrial Cancer | multi-kinase inhibitor (Lenvatinib) | NCT05112991 |  | Recruiting |
|  |  |  | II | NSCLC | EGFR inhibitor (Almonertinib) | NCT05534113 |  | Not yet recruiting |
|  |  |  | II | Colon Cancer | - | NCT05335460 |  | Not yet recruiting |
|  |  |  | II | NSCLC | - | NCT05414630 |  | Not yet recruiting |
|  |  |  | II | GC, GEJC | - | NCT05387681 |  | Not yet recruiting |
|  |  |  | II | NSCLC | Chemotherapy, Endostatin | NCT05243355 |  | Recruiting |
|  |  |  | II | NSCLC | Endostatin | NCT05203276 |  | Not yet recruiting |
|  |  |  | II | NSCLC | Chemotherapy, Endostatin | NCT05529355 |  | Not yet recruiting |
|  |  |  | II | NSCLC | HDAC inhibitor (Chidamide) | NCT05068427 |  | Recruiting |
|  |  |  | II | NSCLC | chemotherapy | NCT05465733 |  | Not yet recruiting |
|  |  |  | II | HCC | multi-kinase inhibitor (Lenvatinib), TACE | NCT05582109 |  | Not yet recruiting |
|  |  |  | II | CRC | endostatin | NCT05551247 |  | Not yet recruiting |
|  |  |  | II | Biliary Tract Cancer | chemotherapy | NCT04910386 |  | Not yet recruiting |
|  |  |  | II | NSCLC | - | NCT05055167 |  | Active, not recruiting |
|  |  |  | I/II | Soft Tissue Sarcoma | anti-CTLA-4 (YH001), chemotherapy | NCT05448820 |  | Not yet recruiting |
|  |  |  | II | Pancreatic cancer | Chemotherapy, Endostatin | NCT05298020 |  | Not yet recruiting |
|  |  |  | II | Solid Tumors | - | NCT04891198 |  | Recruiting |
|  |  |  | II | GC | chemotherapy | NCT05237349 |  | Recruiting |
|  |  |  | II | Ovarian cancer | multi-kinase inhibitor (Lenvatinib), chemotherapy | NCT05422183 |  | Not yet recruiting |
|  |  |  | II | Rectal Adenocarcinoma | chemotherapy, radiation | NCT05216653 | PRECAM | Recruiting |
|  |  |  | II | ESCC | chemotherapy | NCT05552651 |  | Not yet recruiting |
|  |  |  | I/II | NSCLC, HCC, RCC | multi-kinase inhibitor (Lenvatinib) | NCT05024214 |  | Recruiting |
|  |  |  | II | HCC | multi-kinase inhibitor (Lenvatinib), TACE | NCT05213221 | CISLD-12 | Recruiting |
|  |  |  | II | Undifferentiated Pleomorphic Sarcoma, Myxofibrosarcoma | anti-CTLA-4 (Ipilimumab) | NCT04480502 | ENVASARC | Recruiting |
|  |  |  | II | NSCLC | - | NCT05360979 |  | Enrolling by invitation |
|  |  |  | II | CRC | - | NCT05371197 |  | Recruiting |
|  |  |  | II | Biliary Tract Cancer | RC48-ADC | NCT05417230 |  | Not yet recruiting |
|  |  |  | II | Lung cancer | endostatin | NCT05385185 |  | Recruiting |
|  |  |  | I | Solid Tumors | - | NCT03101488 |  | Completed |
|  |  |  | I | Solid Tumors | - | NCT03248843 |  | Completed |
|  |  |  | II | Multiple Primary Cancers | - | NCT04182789 | CPOG035-01 | Unknown status |
|  |  |  | III | Biliary Tract Cancer | chemotherapy | NCT03478488 | KN035-BTC | Recruiting |
|  |  |  | II | HER2-positive Breast Cancer | anti-HER2 (Trastuzumab) | NCT04034823 |  | Unknown status |
|  |  |  | I | Solid Tumors | - | NCT02827968 |  | Completed |
|  |  |  | II | Solid Tumors | - | NCT03667170 |  | Recruiting |
|  |  |  | II | Solid Tumors | anti-VEGF (BD0801), chemotherapy | NCT05148195 |  | Recruiting |
| Adebrelimab (SHR-1316) | Atridia; Jiangsu Hengrui | antagonistic mAb | III | SCLC | chemotherapy, radiation | NCT05496166 |  | Not yet recruiting |
|  |  |  | I/II | HCC | anti-VEGF inhibitor (Bevacizumab) | NCT05444088 |  | Not yet recruiting |
|  |  |  | I/II | NSCLC | chemotherapy | NCT05416775 |  | Recruiting |
|  |  |  | I | Solid Tumors | - | NCT04979390 |  | Not yet recruiting |
|  |  |  | II | SCLC | - | NCT04647357 |  | Not yet recruiting |
|  |  |  | II | OSCC | - | NCT04215471 | NATION1907II | Recruiting |
|  |  |  | I/II | NSCLC | ADC (SHR-A1811) | NCT05482568 |  | Not yet recruiting |
|  |  |  | I | Solid Tumors | - | NCT03133247 |  | Completed |
|  |  |  | I | Malignancies | - | NCT03474289 |  | Unknown status |
|  |  |  | I | Malignancies | CD112R x TIGIT bsAb (SHR-2002) | NCT05082545 |  | Recruiting |
|  |  |  | I | Malignancies | IL-15 agonist (SHR-1501) | NCT03995472 |  | Active, not recruiting |
|  |  |  | III | NSCLC | chemotherapy | NCT04316364 |  | Recruiting |
|  |  |  | II | ESCC | chemotherapy | NCT03732508 |  | Unknown status |
|  |  |  | III | SCLC | chemotherapy | NCT03711305 | CAPSTONE-1 | Active, not recruiting |
|  |  |  | III | SCLC | chemotherapy, radiation | NCT04691063 |  | Enrolling by invitation |
|  |  |  | I/II | SCLC | chemotherapy | NCT04400188 |  | Unknown status |
|  |  |  | II | SCLC | chemotherapy, radiation | NCT04562337 |  | Not yet recruiting |
|  |  |  | I | Malignancies | HRS2300 (undefined mechanism) | NCT05048134 |  | Recruiting |
|  |  |  | I | SCLC | chemotherapy | NCT04041011 |  | Completed |
|  |  |  | II | HER2 Positive Breast Cancer | ADC (SHR-A1811) | NCT05353361 |  | Not yet recruiting |
|  |  |  | I | Advanced Malignant Tumors | CD112R x TIGIT bsAb (SHR-2002) | NCT05198817 |  | Enrolling by invitation |
|  |  |  | Not Applicable | Breast Cancer | radiation (SBRT) | NCT05132790 |  | Recruiting |
|  |  |  | II | TNBC | anti-VEGF (Bevacizumab), chemotherapy | NCT04303988 |  | Unknown status |
|  |  |  | II | Breast Cancer | CDK 4/6 inhibitor (SHR6390), chemotherapy | NCT05205200 | ENIGMA | Not yet recruiting |
|  |  |  | II | ICC | anti-CTLA-4 (IBI310) | NCT04634058 |  | Not yet recruiting |
|  |  |  | I/II | Breast cancer | CDK 4/6 inhibitor (SHR-6390), chemotherapy | NCT05582499 | FASCINATE-N | Not yet recruiting |
| INCB86550 | Incyte Corporation | small molecule inhibitor | II | Solid Tumors | - | NCT04629339 |  | Active, not recruiting |
|  |  |  | I | Healthy Volunteers | - | NCT05101369 |  | Completed |
|  |  |  | I | Solid Tumors | - | NCT03762447 |  | Active, not recruiting |
| MAX-10181 | Maxinovel Pharmaceuticals | small molecule inhibitor | I | Solid Tumors | - | NCT05196360 |  | Recruiting |
|  |  |  | I | Solid Tumors | - | NCT04122339 |  | Recruiting |
| ***Targeting CTLA-4*** | | | | | | | | |
| Ipilimumab | Bristol-Myers Squibb | antagonistic mAb | III | NSCLC | anti-PD-1 (Nivolumab), chemotherapy | NCT02477826 | CHECKMATE-227 | Active, not recruiting |
| Tremelimumab | Pfizer, AstraZeneca | antagonistic mAb | III | HCC | anti-PD-L1 (Durvalumab) | NCT03298451 | HIMALAYA | Recruiting |
| BMS-986249 | Bristol-Myers Squibb | probody | I/II | Solid Tumors | Anti-PD-1 (nivolumab) | NCT03369223 |  | Recruiting |
| Botensilimab (AGEN-1811) | Agenus | antagonistic mAb | II | Melanoma | - | NCT05529316 |  | Not yet recruiting |
|  |  |  | II | CRC | anti-PD-1 (Balstilimab) | NCT05571293 | NEST-1 | Not yet recruiting |
|  |  |  | I | Solid Tumors | anti-ILT2 (AGEN1571), anti-PD-1 (Balstilimab) | NCT05377528 |  | Recruiting |
|  |  |  | I | Solid Tumors | anti-PD-1 (Balstilimab) | NCT03860272 |  | Recruiting |
|  |  |  | I | Solid Tumors | anti-CD137 (AGEN2373) | NCT04121676 |  | Recruiting |
| Zalifrelimab (AGEN1884) | Agenus | antagonistic mAb | II | Cervical Cancer | anti-PD-1 (Balstilimab) | NCT05033132 |  | Recruiting |
|  |  |  | Expanded Access Study | Cancer | anti-PD-1 (Balstilimab) | NCT05572970 |  | Available |
|  |  |  | I/II | PDAC | chemotherapy, Hedgehog protein inhibitor (NLM-001) | NCT04827953 | NUMANTIA | Recruiting |
|  |  |  | I | DIPG, DMG | cancer vaccine (rHSC-DIPGVax), anti-PD-1 (Balstilimab) | NCT04943848 |  | Recruiting |
|  |  |  | I | NMIBC | TLR-7/8 agonist (UGN-201) | NCT05375903 |  | Recruiting |
|  |  |  | II | Soft Tissue Sarcoma | chemotherapy, anti-PD-1 (AGEN2034) | NCT04028063 |  | Active, not recruiting |
|  |  |  | I/II | Solid Tumors | - | NCT02694822 |  | Completed |
|  |  |  | I/II | Cervical Cancer | anti-PD-1 (AGEN2034) | NCT03495882 |  | Active, not recruiting |
|  |  |  | II | MIBC | chemotherapy, anti-PD-1 (AGEN2034) | NCT04430036 |  | Active, not recruiting |
|  |  |  | II | Cervical Cancer | anti-PD-1 (AGEN2034) | NCT03894215 | RaPiDS | Recruiting |
| Quavonlimab (MK-1308) | Merck Sharp & Dohme | antagonistic mAb | II | HCC | multi-kinase inhibitor (Lenvatinib), anti-PD-1 (Pembrolizumab) | NCT04740307 |  | Active, not recruiting |
|  |  |  | I/II | Solid Tumors | anti-PD-1 (Pembrolizumab) | NCT03179436 |  | Active, not recruiting |
|  |  |  | II | CRC | anti-PD-1 (Pembrolizumab) | NCT04895722 |  | Recruiting |
|  |  |  | III | RCC | multi-kinase inhibitor (Lenvatinib), anti-PD-1 (Pembrolizumab) | NCT04736706 |  | Recruiting |
|  |  |  | II | NSCLC | anti-PD-1 (Pembrolizumab) | NCT03516981 |  | Active, not recruiting |
|  |  |  | I/II | Melanoma | multi-kinase inhibitor (Lenvatinib), anti-PD-1 (Pembrolizumab) | NCT04700072 |  | Recruiting |
|  |  |  | I/II | Melanoma | multi-kinase inhibitor (Lenvatinib), anti-PD-1 (Pembrolizumab) | NCT04305041 |  | Recruiting |
|  |  |  | I/II | SCLC | multi-kinase inhibitor (Lenvatinib), anti-PD-1 (Pembrolizumab) | NCT04938817 |  | Recruiting |
|  |  |  | I/II | Melanoma | multi-kinase inhibitor (Lenvatinib), anti-PD-1 (Pembrolizumab) | NCT04305054 |  | Recruiting |
|  |  |  | I/II | RCC | multi-kinase inhibitor (Lenvatinib), anti-PD-1 (Pembrolizumab) | NCT04626479 |  | Recruiting |
|  |  |  | I/II | RCC | multi-kinase inhibitor (Lenvatinib), anti-PD-1 (Pembrolizumab) | NCT04626518 |  | Recruiting |
| Porustobart (HBM-4003) | Harbour BioMed | antagonistic mAb | I | Solid Tumors | anti-PD-1 (Triprilimab) | NCT04727164 |  | Not yet recruiting |
|  |  |  | I | Solid Tumors | anti-PD-1 (Pembrolizumab) | NCT04866485 |  | Not yet recruiting |
|  |  |  | I | Solid Tumors | anti-PD-1 (Triprilimab) | NCT05149027 |  | Not yet recruiting |
|  |  |  | I | Solid Tumors | anti-PD-1 (Toripalimab) | NCT05167071 |  | Not yet recruiting |
|  |  |  | I | Solid Tumors | - | NCT04135261 |  | Active, not recruiting |
| YH-001 | Eucure Biopharma | antagonistic mAb | II | HCC, NSCLC | anti-PD-1 (Toripalimab) | NCT05212922 |  | Not yet recruiting |
|  |  |  | I | Solid Tumors | - | NCT04699929 |  | Recruiting |
|  |  |  | I | Solid Tumors | anti-PD-1 (Toripalimab) | NCT04357756 |  | Recruiting |
|  |  |  | I/II | Soft Tissue Sarcoma | anti-PD-L1 (Envafolimab), chemotherapy | NCT05448820 |  | Not yet recruiting |
|  |  |  | I | Solid Tumors | anti-OX40 (YH002) | NCT05169697 |  | Recruiting |
|  |  |  | I | Solid Tumors | anti-CD40 (YH003), anti-PD-1 (Pembrolizumab) | NCT05176509 |  | Recruiting |
| ADG-116 | Adagene | antagonistic mAb | I | Solid Tumors | - | NCT05277402 |  | Recruiting |
|  |  |  | I | Solid Tumors | anti-PD1 mAb, anti-CD137 (ADG106) | NCT04501276 |  | Recruiting |
| ONC-392 | OncoImmune | antagonistic mAb | II | Ovarian Cancer, Primary Peritoneal Carcinoma, Fallopian Tube Cancer | anti-PD-1 (Pembrolizumab) | NCT05446298 | PRESERVE-004 | Not yet recruiting |
|  |  |  | I/II | Solid Tumors | anti-PD-1 (Pembrolizumab) | NCT04140526 | PRESERVE-001 | Recruiting |

Annotation:

1. Cells filled with blue color for drug names indicate that the corresponding drug has been approved by the FDA.
2. For drugs approved by FDA, owing to its proven efficacy and the abundance of the clinical trials, only one representative trial is listed.

NSCLC non-small cell lung cancer, NPC nasopharyngeal cancer, RCC renal cell carcinoma, HCC hepatocellular carcinoma, ESCC esophageal squamous-cell carcinoma, ICC intrahepatic cholangiocarcinoma, UC urothelial carcinoma, TNBC triple-negative breast cancer, BC biliary tract cancer, EC esophageal cancer, GC gastric cancer, CRC colorectal cancer, GEJC gastroesophageal junction cancer, HL Hodgkin lymphoma, DLBCL diffuse large B cell lymphoma, NMIBC non–muscle-invasive bladder cancer, HNSCC head and neck squamous cell carcinoma, SCLC small-cell lung cancer, GEC gastroesophageal cancer, MSI-H microsatellite instability-high, GI gastrointestinal, MCC Merkel cell carcinoma, PDAC pancreatic ductal adenocarcinoma, dMMR deficient mismatch repair, CML chronic myeloid leukemia, MDS myelodysplastic syndrome, NHL non-Hodgkin lymphoma, MM multiple myeloma, AML acute myeloid leukemia, MIBC muscle-invasive bladder cancer, PC prostate cancer, CRPC castration-resistant prostate cancer, DMG diffuse midline glioma, DIPG diffuse intrinsic pontine glioma, PTCL peripheral T cell lymphoma, OSCC oral squamous cell carcinoma.

**Supplementary Table 3. Therapeutics targeting co-stimulatory molecules**

| Drug name | Manufacturer | Type of agent | Phase | Disease Area (Selected Indications) | Therapeutic combination | Identifier | | Status |
| --- | --- | --- | --- | --- | --- | --- | --- | --- |
|  |  |  |  |  |  | Trial number | Trial Name |  |
| ***Targeting ICOS*** | | | | | | | | |
| Vopratelimab (JTX-2011) | Jounce Therapeutics | agonistic mAb | II | NSCLC | anti-PD-1 (Pimivalimab, JTX-4014) | NCT04549025 | SELECT | Active, not recruiting |
|  |  |  | I/II | Solid Tumors | anti-PD-1 (Nivolumab), anti-CTLA-4 (Ipilimumab) | NCT04319224 |  | Recruiting |
|  |  |  | II | NSCLC, UC | anti-CTLA-4 (Ipilimumab) | NCT03989362 | EMERGE | Active, not recruiting |
|  |  |  | I/II | Solid Tumors | anti-PD-1 (Pembrolizumab, Nivolumab), anti-CTLA-4 (Ipilimumab) | NCT02904226 | ICONIC | Completed |
| Alomfilimab (KY1044, SAR-445256) | Kymab, Sanofi | agonistic mAb | I/II | Solid Tumors | anti-PD-1 (Atezolizumab) | NCT03829501 |  | Recruiting |
| ***Targeting CD40*** | | | | | | | | |
| SEA-CD40 | Seagen, Merck Sharp & Dohme | agonistic mAb | II | Melanoma, NSCLC | anti-PD-1 (Pembrolizumab), Chemotherapy | NCT04993677 |  | Recruiting |
|  |  |  | I | Solid Tumors | anti-PD-1 (Pembrolizumab), Chemotherapy | NCT02376699 | SGNS40-001 | Active, not recruiting |
| Mitazalimab (Vanalimab, ADC-1013, JNJ-64457107) | Alligator Bioscience, Janssen | agonistic mAb | I/II | PDAC | Chemotherapy | NCT04888312 | OPTIMIZE-1 | Enrolling by invitation |
|  |  |  | I | Solid Tumors |  | NCT02829099 |  | Completed |
|  |  |  | I | Solid Tumors |  | NCT02379741 |  | Completed |
| Sotigalimab (APX005M) | Apexigen, Bristol-Myers Squibb | agonistic mAb | II | Ovarian Cancer | Chemotherapy, Radiation | NCT05201001 |  | Not yet recruiting |
|  |  |  | I | Melanoma, RCC | anti-PD-1 (Nivolumab), anti-CTLA-4 (Ipilimumab) | NCT04495257 |  | Recruiting |
|  |  |  | II | Melanoma |  | NCT04337931 |  | Active, not recruiting |
|  |  |  | II | Rectal Adenocarcinoma | Chemotherapy, Radiation | NCT04130854 | INNATE | Recruiting |
|  |  |  | II | Soft Tissue Sarcoma | Chemotherapy | NCT03719430 |  | Recruiting |
|  |  |  | I | Solid Tumors | anti-PD-1 (Nivolumab), anti-CSF1R (Cabiralizumab) | NCT03502330 |  | Recruiting |
|  |  |  | I | Pediatric CNS Tumors |  | NCT03389802 | PBTC-051 | Recruiting |
|  |  |  | I/II | PDAC | anti-PD-1 (Nivolumab), anti-CSF1R (Cabiralizumab) | NCT03214250 | PRINCE, PICI0002 | Active, not recruiting |
|  |  |  | II | EC | Chemotherapy, Radiation, Surgery | NCT03165994 |  | Recruiting |
|  |  |  | I/II | Solid Tumors | anti-PD-1 (Nivolumab) | NCT03123783 |  | Completed |
|  |  |  | I/II | Melanoma | anti-PD-1 (Pembrolizumab) | NCT02706353 |  | Recruiting |
|  |  |  | I | CRC, PDAC | anti-PD-1 (Pembrolizumab), TLR-7 agonist (Imiquimod), Cancer Vaccine | NCT02600949 |  | Recruiting |
|  |  |  | I | Solid Tumors |  | NCT02482168 |  | Completed |
| Giloralimab (ABBV-927) | AbbVie | agonistic mAb | II | Pancreatic Cancer | anti-PD-1 (Budigalimab), Chemotherapy | NCT04807972 |  | Recruiting |
|  |  |  | I | Solid Tumors | anti-PD-1 (Budigalimab), anti-OX40 agonist (Revdofilimab), Chemotherapy | NCT03893955 |  | Recruiting |
|  |  |  | I | Solid Tumors | anti-PD-1 (Budigalimab) | NCT02988960 |  | Active, not recruiting |
| YH003 | Eucure Biopharma | agonistic mAb | I | Solid Tumors | anti-PD-1 (Pembrolizumab), anti-CTLA-4 (YH001) | NCT05176509 |  | Recruiting |
|  |  |  | II | Melanoma, PDAC | anti-PD-1 (Toripalimab), Chemotherapy | NCT05031494 |  | Recruiting |
|  |  |  | I | Solid Tumors |  | NCT05017623 |  | Recruiting |
|  |  |  | I/II | Solid Tumors | anti-PD-1 (Toripalimab), Chemotherapy | NCT04481009 |  | Active, not recruiting |
| CDX-1140 | Celldex Therapeutics | agonistic mAb | I | Solid Tumors | anti-PD-1 (Pembrolizumab), Cell Therapy | NCT05349890 |  | Not yet recruiting |
|  |  |  | II | Ovarian Cancer | anti-PD-1 (Pembrolizumab), anti-VEGF (Bevacizumab), Chemotherapy | NCT05231122 |  | Not yet recruiting |
|  |  |  | I | TNBC | Human recombinant Flt3L (CDX-301), Chemotherapy | NCT05029999 |  | Recruiting |
|  |  |  | I | Breast Cancer | Human recombinant Flt3L (CDX-301), Chemotherapy, Radiation, Immunostimulant (Poly-ICLC) | NCT04616248 |  | Withdrawn |
|  |  |  | II | Pancreatic Cancer | Human recombinant Flt3L (CDX-301) | NCT04536077 |  | Recruiting |
|  |  |  | I | Solid Tumors | anti-PD-1 (Pembrolizumab) | NCT04520711 | Hotspot TCR-T | Recruiting |
|  |  |  | I/II | NSCLC | Human recombinant Flt3L (CDX-301), Radiation | NCT04491084 |  | Recruiting |
|  |  |  | I/II | Melanoma | Cancer Vaccine (6MHP and NeoAg-mBRAF), Immunostimulant (Poly-ICLC) | NCT04364230 | Mel66 | Recruiting |
|  |  |  | I | Solid Tumors | anti-PD-1 (Pembrolizumab), rhFlt3L (CDX-301), Chemotherapy | NCT03329950 |  | Active, not recruiting |
| ***Targeting OX40*** | | | | | | | | |
| Revdofilimab (ABBV-368) | Abbvie | agonistic mAb | I | Solid Tumors | anti-PD-1 (Budigalimab), anti-CTLA-4 (Ipilimumab) | NCT04196283 |  | Active, not recruiting |
|  |  |  | I | Solid Tumors | anti-PD-1 (Budigalimab), CD40 agonist (Giloralimab/ABBV-927), Chemotherapy | NCT03893955 |  | Recruiting |
|  |  |  | I | Head and Neck Cancer |  | NCT03818542 |  | Terminated |
|  |  |  | I | Solid Tumors | anti-PD-1 (Budigalimab) | NCT03071757 |  | Completed |
| HFB301001 | HiFiBiO Therapeutics | agonistic mAb | I | Solid Tumors |  | NCT05229601 |  | Recruiting |
| BGB-A445 | BeiGene | agonistic mAb | I | Solid Tumors | anti-PD-1 (Tislelizumab) | NCT04215978 |  | Recruiting |
| ***Targeting 4-1BB/CD137*** | | | | | | | | |
| ADG106 | Adagene | agonistic mAb | I/II | Breast Cancer | Chemotherapy | NCT05275777 |  | Not yet recruiting |
|  |  |  | I/II | NSCLC | anti-PD-1 (Nivolumab) | NCT05236608 | ADIVO Lung | Recruiting |
|  |  |  | I/II | Solid Tumors | anti-PD-1 | NCT04775680 |  | Recruiting |
|  |  |  | I | Solid Tumors | anti-PD-1 (Toripalimab), anti-CTLA-4 (ADG126) | NCT04645069 |  | Recruiting |
|  |  |  | I | Solid Tumors | anti-CTLA-4 (ADG116) | NCT04501276 |  | Recruiting |
|  |  |  | I | Solid Tumors |  | NCT03802955 |  | Active, not recruiting |
|  |  |  | I | Solid Tumors |  | NCT03707093 |  | Active, not recruiting |
| LVGN6051 | Lyvgen Biopharma | agonistic mAb | I/II | Soft Tissue Sarcoma | multi-kinase inh (Anlotinib) | NCT05301764 |  | Not yet recruiting |
|  |  |  | I | Solid Tumors | anti-PD-1 (LVGN3616), anti-CTLA-4 (ADG126) | NCT05075993 |  | Recruiting |
|  |  |  | I | Solid Tumors | anti-PD-1 (Pembrolizumab) | NCT04694781 |  | Recruiting |
|  |  |  | I | Solid Tumors | anti-PD-1 (Pembrolizumab) | NCT04130542 | MK-3475-A31/KEYNOTE-A31 | Recruiting |
| AGEN2373 | Agenus | agonistic mAb | I | Solid Tumors | anti-CTLA-4 (AGEN1181) | NCT04121676 |  | Recruiting |
| ATOR1017 | Alligator Bioscience | agonistic mAb | I | Solid Tumors |  | NCT04144842 |  | Recruiting |

NSCLC non-small cell lung cancer, UC urothelial carcinoma, PDAC pancreatic ductal adenocarcinoma, RCC renal cell carcinoma, EC esophageal cancer, CRC colorectal cancer, TNBC triple-negative breast cancer.

**Supplementary Table 4. Bi- and multi-specific antibodies**

| Drug name | Manufacturer | Components | | | Phase | Disease Area (Selected Indications) | Therapeutic combination | Identifier | | Status |
| --- | --- | --- | --- | --- | --- | --- | --- | --- | --- | --- |
|  |  | Component 1 | Component 2 | Component 3 |  |  |  | Trial Number | Trial Name |  |
| ***Cell Engagers*** | | | | | | | | | | |
| ***T Cell Engagers*** | | | | | | | | | | |
|  |  | ***CD3 × TAA*** | | | | | | | | |
| Blinatumomab (AMG-103, MEDI538) | Amgen | anti-CD3 | anti-CD19 |  | II | B-precursor ALL | - | NCT01207388 | BLAST | Completed |
| Teclistamab (JNJ-64007957) | Janssen, Genmab | anti-CD3 | anti-BCMA |  | I | MM | - | NCT03145181 | MajesTEC-1 | Recruiting |
| Mosunetuzumab (RG7828, RO7030816) | Genentech, Roche | anti-CD3 | anti-CD20 |  | I/II | FL, NHL, CLL | anti-PD-L1 (Atezolizumab) | NCT02500407 |  | Recruiting |
| APVO436 | Aptevo Therapeutics | anti-CD3 | anti-CD123 (IL-3Rα) |  | I | AML | - | NCT04973618 |  | Withdrawn |
|  |  |  |  |  | I | AML, MDS | - | NCT03647800 |  | Recruiting |
| GB261 | Genor Biopharma | anti-CD3 | anti-CD20 |  | I/II | CLL, B-Cell NHL | - | NCT04923048 |  | Recruiting |
| Epcoritamab (GEN3013) | AbbVie, Genmab | anti-CD3 | anti-CD20 |  | II | NHL | BTK inh (Ibrutinib), anti-CD20 (Rituximab), anti-CD79b (Polatuzumab), Chemotherapy | NCT05283720 |  | Recruiting |
|  |  |  |  |  | I | NHL | - | NCT05206357 |  | Recruiting |
|  |  |  |  |  | I | B-Cell NHL | anti-CD20 (Rituximab), Chemotherapy | NCT05201248 |  | Recruiting |
|  |  |  |  |  | I/II | DLBCL, FL | anti-CD20 (Rituximab), Chemotherapy | NCT04663347 | EPCORE NHL-2 | Recruiting |
|  |  |  |  |  | III | DLBCL | Chemotherapy | NCT04628494 | EPCORE DLBCL-1 | Recruiting |
|  |  |  |  |  | I/II | CLL | - | NCT04623541 | EPCORE CLL-1 | Recruiting |
|  |  |  |  |  | I/II | B-Cell NHL, FL | anti-CD20 (Rituximab), Chemotherapy | NCT04542824 |  | Recruiting |
|  |  |  |  |  | I/II | B-Cell NHL | CD37 x CD37 bsAb (GEN3009) | NCT04358458 |  | Recruiting |
|  |  |  |  |  | I/II | B-Cell NHL | - | NCT03625037 | EPCORE NHL-1 | Recruiting |
| Glofitamab (RG6026, RO 7082859) | Roche, Genentech, Chugai Pharmaceutical | anti-CD3 | anti-CD20 |  | I | DLBCL | anti-CD20 (Rituximab, Obinutuzumab), Chemotherapy | NCT05364424 |  | Not yet recruiting |
|  |  |  |  |  | II | DLBCL | BTK inh (Poseltinib), anti-IL-6 (Tocilizumab) | NCT05335018 |  | Recruiting |
|  |  |  |  |  | I | NHL | CD19 x CD28 bsAb (RO7443904), anti-CD20 (Obinutuzumab), anti-IL-6 (Tocilizumab) | NCT05219513 |  | Recruiting |
|  |  |  |  |  | I | NHL | Cereblon E3 ligase modulator (CC-220, CC-99282), anti-CD20 (Obinutuzumab), anti-IL-6 (Tocilizumab) | NCT05169515 |  | Not yet recruiting |
|  |  |  |  |  | II | Lymphoma | anti-CD20 (Rituximab), anti-IL-6 (Tocilizumab), Chemotherapy | NCT04980222 |  | Recruiting |
|  |  |  |  |  | I/II | DLBCL | anti-CD20 (Rituximab), anti-79b ADC (Polatuzumab vedotin), Chemotherapy | NCT04914741 | COALITION | Recruiting |
|  |  |  |  |  | II | Large B-cell Lymphoma | anti-CD20 (Obinutuzumab), Cell Therapy (CD19 CAR-T) | NCT04889716 |  | Recruiting |
|  |  |  |  |  | II | B-Cell NHL | anti-CD20 (Obinutuzumab) | NCT04703686 |  | Recruiting |
|  |  |  |  |  | I | DLBCL | anti-CD20 (Obinutuzumab), anti-IL-6 (Tocilizumab) | NCT04657302 |  | Active, not recruiting |
|  |  |  |  |  | III | DLBCL | anti-CD20 (Obinutuzumab), anti-IL-6 (Tocilizumab), Chemotherapy | NCT04408638 |  | Recruiting |
|  |  |  |  |  | I | B-cell Lymphoma | anti-CD20 (Obinutuzumab), anti-IL-6 (Tocilizumab), Chemotherapy | NCT04313608 |  | Completed |
|  |  |  |  |  | I | NHL | CD19 x 4-1BBL fusion protein (RO7227166), anti-CD20 (Obinutuzumab), anti-IL-6 (Tocilizumab) | NCT04077723 |  | Recruiting |
|  |  |  |  |  | I/II | NHL | anti-PD-1 (Atezolizumab), anti-CD20 (Obinutuzumab), anti-79b ADC (Polatuzumab vedotin) | NCT03533283 | NP39488 | Recruiting |
|  |  |  |  |  | I | B-Cell Lymphoma, NHL | anti-CD20 (Rituximab, Obinutuzumab), anti-IL-6 (Tocilizumab), Chemotherapy | NCT03467373 |  | Recruiting |
|  |  |  |  |  | I/II | NHL | anti-CD20 (Obinutuzumab), anti-IL-6 (Tocilizumab) | NCT03075696 | NP30179 | Recruiting |
| Plamotamab (XmAb13676) | Xencor, Janssen, Novartis | anti-CD3 | anti-CD20 |  | II | DLBCL | anti-CD19 (Tafasitamab) | NCT05328102 |  | Recruiting |
|  |  |  |  |  | I | B-Cell NHL, CLL | - | NCT02924402 |  | Recruiting |
| Odronextamab (REGN-1979) | Regeneron | anti-CD3 | anti-CD20 |  | II | B-Cell NHL | - | NCT03888105 | ELM-2 | Recruiting |
|  |  |  |  |  | I | Lymphoma | anti-PD-1 (Cemiplimab) | NCT02651662 |  | Active, not recruiting |
|  |  |  |  |  | I | NHL, CLL | - | NCT02290951 | ELM-1 | Recruiting |
| Runimotamab (RG6194, RO-7227780) | Genentech | anti-CD3 | anti-HER2 |  | I | Solid Tumors | anti-HER2 (Trastuzumab), anti-IL-6 (Tocilizumab) | NCT03448042 |  | Recruiting |
| AMX 818 | Amunix, Sanofi | anti-CD3 | anti-HER2 |  | I | HER2-Expressing Cancers | anti-PD-1 (Pembrolizumab) | NCT05356741 |  | Recruiting |
| ISB-1342 (GBR 1342) | Glenmark Pharmaceuticals | anti-CD3 | anti-CD38 |  | I | MM | - | NCT03309111 |  | Recruiting |
| Vixtimotamab (AMV-564) | Affimed Therapeutics | anti-CD3 | anti-CD33 (Siglec-3) |  | I | Solid Tumors | - | NCT04128423 |  | Active, not recruiting |
|  |  |  |  |  | I | MDS | - | NCT03516591 |  | Completed |
|  |  |  |  |  | I | AML | anti-PD-1 (Pembrolizumab) | NCT03144245 |  | Completed |
| Cevostamab (BFCR-4350A, RG 6160, RO-7187797) | Genentech | anti-CD3 | anti-FcRH5 |  | I | MM | anti-IL-6 (Tocilizumab) | NCT03275103 |  | Recruiting |
|  |  |  |  |  | I | MM | anti-IL-6 (Tocilizumab), anti-CD38 (Daratumumab), Chemotherapy | NCT04910568 | CAMMA 1 | Recruiting |
| Elranatamab (PF-06863135) | Pfizer | anti-CD3 | anti-BCMA |  | III | MM | Chemotherapy | NCT05317416 | MagnetisMM-7 | Recruiting |
|  |  |  |  |  | II | MM | - | NCT05228470 | MagnetisMM-8 | Recruiting |
|  |  |  |  |  | II | MM | Chemotherapy | NCT05090566 | MagnetisMM-4 | Recruiting |
|  |  |  |  |  | III | MM | anti-CD38 (Daratumumab) | NCT05020236 | MagnetisMM-5 | Recruiting |
|  |  |  |  |  | II | MM | - | NCT05014412 | MagnetisMM-9 | Recruiting |
|  |  |  |  |  | I | MM | - | NCT04798586 | MagnetisMM-2 | Active, not recruiting |
|  |  |  |  |  | II | MM | - | NCT04649359 | MagnetisMM-3 | Active, not recruiting |
|  |  |  |  |  | I | MM | Chemotherapy | NCT03269136 | MagnetisMM-1 | Active, not recruiting |
| Pavurutamab (AMG701) | Amgen | anti-CD3 | anti-BCMA |  | I | MM | - | NCT04998747 | ProxiMMity-1 | Not yet recruiting |
|  |  |  |  |  | I | MM | Chemotherapy | NCT03287908 |  | Recruiting |
| CM336 | Keymed Biosciences | anti-CD3 | anti-BCMA |  | I/II | MM | - | NCT05299424 |  | Not yet recruiting |
| TNB-383B (ABBV-383) | TeneoBio, AbbVie, Amgen | anti-CD3 | anti-BCMA |  | I | MM | - | NCT03933735 |  | Recruiting |
| Ubamatamab (REGN4018) | Regeneron | anti-CD3 | anti-MUC16 |  | I/II | Ovarian Cancer | MUC16 x CD28 bsAb (REGN5668) | NCT04590326 |  | Recruiting |
|  |  |  |  |  | I/II | Ovarian Cancer | anti-PD-1 (Cemiplimab) | NCT03564340 |  | Recruiting |
| AMG199 | Amgen | anti-CD3 | anti-MUC17 |  | I | MUC17-positive Solid Tumors | - | NCT04117958 |  | Recruiting |
| Tarlatamab (AMG757) | Amgen | anti-CD3 | anti-DLL3 |  | I | SCLC | anti-PD-1 (Atezolizumab), Chemotherapy | NCT05361395 |  | Not yet recruiting |
|  |  |  |  |  | II | SCLC | - | NCT05060016 | DeLLphi-301 | Recruiting |
|  |  |  |  |  | I | SCLC | anti-PD-1 (AMG404) | NCT04885998 |  | Recruiting |
|  |  |  |  |  | I | Neuroendocrine PC | - | NCT04702737 |  | Recruiting |
|  |  |  |  |  | I | SCLC | anti-PD-1 (Pembrolizumab) | NCT03319940 |  | Recruiting |
| BI 764532 | Boehringer Ingelheim | anti-CD3 | anti-DLL3 |  | I | SCLC, Neuroendocrine Tumors | - | NCT04429087 |  | Recruiting |
| Cibisatamab (RG7802, RO6958688) | Roche | anti-CD3 | anti-CEA |  | I/II | CRC | 4-1BB x FAP bsAb (RO7122290), anti-CD20 (Obinutuzumab) | NCT04826003 |  | Recruiting |
|  |  |  |  |  | I | CRC | anti-PD-1 (Atezolizumab), anti-CD20 (Obinutuzumab), anti-IL-6 (Tocilizumab) | NCT03866239 |  | Active, not recruiting |
|  |  |  |  |  | I/II | NSCLC | anti-PD-1 (Atezolizumab), anti-IL-6 (Tocilizumab) | NCT03337698 | Morpheus Lung | Recruiting |
|  |  |  |  |  | I | Solid Tumors | anti-PD-1 (Atezolizumab) | NCT02650713 |  | Completed |
|  |  |  |  |  | I | Solid Tumors | anti-CD20 (Obinutuzumab), anti-IL-6 (Tocilizumab) | NCT02324257 |  | Completed |
| Acapatamab (AMG 160) | Amgen | anti-CD3 | anti-PSMA |  | I | NSCLC | - | NCT04822298 |  | Active, not recruiting |
|  |  |  |  |  | I/II | PC | anti-PD-1 (AMG404) | NCT04631601 |  | Active, not recruiting |
|  |  |  |  |  | I | PC | anti-PD-1 (Pembrolizumab) | NCT03792841 |  | Active, not recruiting |
| AMG 340 (TNB-585) | Amgen | anti-CD3 | anti-PSMA |  | I | PC | - | NCT04740034 |  | Recruiting |
| CCW702 | Calibr, AbbVie | anti-CD3 | anti-PSMA |  | I | PC | - | NCT04077021 |  | Recruiting |
| AMG 509 | Amgen, BeiGene, Xencor | anti-CD3 | anti-STEAP1 |  | I | PC | anti-PD-1 (Pembrolizumab), Chemotherapy | NCT04221542 |  | Recruiting |
| ERY974 | Chugai Pharmaceutical | anti-CD3 | anti-Glypican 3 (GPC3) |  | I | HCC | anti-PD-1 (Atezolizumab), anti-IL-6 (Tocilizumab), anti-VEGF (Bevacizumab) | NCT05022927 |  | Recruiting |
|  |  |  |  |  | I | Solid Tumors | - | NCT02748837 |  | Completed |
| CM350 | KeyMed Biosciences | anti-CD3 | anti-Glypican 3 (GPC3) |  | I/II | Solid Tumors | - | NCT05263960 |  | Recruiting |
| Talquetamab (JNJ-64407564) | Janssen, Gemnmab | anti-CD3 | anti-GPRC5D |  | I | MM | CD3 x PSMA BiTE (Teclistamab), anti-PD-1 | NCT05338775 |  | Recruiting |
|  |  |  |  |  | I | MM | anti-CD38 (Daratumumab), Chemotherapy | NCT05050097 |  | Recruiting |
|  |  |  |  |  | I | MM | - | NCT04773522 |  | Recruiting |
|  |  |  |  |  | II | MM | - | NCT04634552 |  | Recruiting |
|  |  |  |  |  | I | MM | CD3 x PSMA BiTE (Teclistamab), anti-CD38 (Daratumumab) | NCT04586426 |  | Recruiting |
|  |  |  |  |  | I | MM | CD3 x PSMA BiTE (Teclistamab), anti-CD38 (Daratumumab), Chemotherapy | NCT04108195 |  | Recruiting |
|  |  |  |  |  | I | MM | - | NCT03399799 |  | Recruiting |
| AMG 427 | Amgen, BeiGene | anti-CD3 | anti-FLT3 |  | I | AML | - | NCT03541369 |  | Recruiting |
| NVG-111 | NovalGen | anti-CD3 | anti-ROR1 |  | I/II | CLL, Lymphoma | - | NCT04763083 |  | Recruiting |
| IBI-389 | Innovent Biologics | anti-CD3 | anti-Claudin 18.2 |  | I | Solid Tumors | anti-PD-1 (Sintilimab) | NCT05164458 |  | Not yet recruiting |
|  |  | ***CD3 × TAA × co-stimulatory molecule*** | | | | | | | | |
| SAR442257 | Sanofi | anti-CD3 | anti-CD38 | CD28 agonist | I | MM, NHL | - | NCT04401020 |  | Recruiting |
|  |  | ***CD3 × TAA × PK/PD improvement element*** | | | | | | | | |
| HPN424 | Harpoon Therapeutics | anti-CD3 | anti-PSMA | human serum albumin | I/II | PC | - | NCT03577028 |  | Recruiting |
| HPN536 | Harpoon Therapeutics | anti-CD3 | anti-mesothelin | human serum albumin | I/II | Solid Tumors With Mesothelin Expression | - | NCT03872206 |  | Recruiting |
| HPN328 | Harpoon Therapeutics | anti-CD3 | anti-DLL3 | human serum albumin | I/II | SCLC | - | NCT04471727 |  | Recruiting |
| TAK-186 (MVC-101) | Takeda, Maverick Therapeutics | anti-CD3 | anti-EGFR | human serum albumin | I/II | HNSCC, SCLC, CRC | - | NCT04844073 |  | Recruiting |
|  |  | ***CD3 × HLA-intracellular oncoprotein*** | | | | | | | | |
| RG6007 (RO7283420) | Roche | anti-CD3 | HLA-A2-WT1 |  | I | AML | anti-IL-6 (Tocilizumab), multi-kinase inh (Dasatinib), Chemotherapy | NCT04580121 |  | Recruiting |
|  |  | ***CD3 x affinity-enhanced TCR (ImmTAC)*** | | | | | | | | |
| Tebentafusp (IMCgp100) | Immunocore | anti-CD3 | gp100 TCR |  | FDA approved |  |  |  |  |  |
| IMC-C103C (RG6290) | Immunocore, Roche | anti-CD3 | MAGE-A4 TCR |  | I/II | Solid Tumors | anti-PD-1 (Atezolizumab) | NCT03973333 | IMC-C103C-101 | Recruiting |
| IMC-F106C | Immunocore | anti-CD3 | PRAME TCR |  | I/II | Solid Tumors | anti-PD-(L)1 | NCT04262466 |  | Recruiting |
|  |  | ***Vγ9Vδ2 TCR × TAA*** | | | | | | | | |
| LAVA-051 | Lava Therapeutics | anti-Vγ9Vδ2 TCR | anti-CD1d |  | I/II | CLL, MM, AML | - | NCT04887259 |  | Recruiting |
| ***Innate Cell Engager/NK Cell Engager*** | | | | | | | | | | |
| AFM13 | Affimed Therapeutics | anti-CD16a/FcγRIIIA | anti-CD30 |  | II | T Cell Lymphoma, Mycosis Fungoides | - | NCT04101331 | REDIRECT | Active, not recruiting |
|  |  |  |  |  | I/II | HL, NHL | Cell therapy, Chemotherapy | NCT04074746 |  | Recruiting |
|  |  |  |  |  | I/II | T Cell Lymphoma | - | NCT03192202 |  | Completed |
|  |  |  |  |  | I | HL | anti-PD-1 (Pembrolizumab) | NCT02665650 |  | Completed |
|  |  |  |  |  | II | HL | - | NCT02321592 | GHSG-AFM13 | Completed |
|  |  |  |  |  | I | HL | - | NCT01221571 |  | Completed |
| AFM24 | Affimed Therapeutics | anti-CD16a/FcγRIIIA | anti-EGFR |  | I/II | Solid Tumors | anti-PD-1 (Atezolizumab) | NCT05109442 |  | Recruiting |
|  |  |  |  |  | I/II | Solid Tumors | Cell therapy (SNK01) | NCT05099549 |  | Recruiting |
|  |  |  |  |  | I/II | Solid Tumors | - | NCT04259450 |  | Recruiting |
| ***General immunoregulatory bispecific antibodies*** | | | | | | | | | | |
| ***Group I (stimulating co-stimulatory molecules)*** | | | | | | | | | | |
|  |  | ***Co-stimulatory molecule x TAA/Tumor microenvironment protein*** | | | | | | | | |
| BT7480 | Bicycle Therapeutics | 4-1BB agonist | anti-Nectin-4 |  | I/II | Solid Tumors | anti-PD-1(Nivolumab) | NCT05163041 | BT7480-100 | Recruiting |
| Cinrebafusp alfa (PRS-343) | Pieris Pharmaceuticals | 4-1BB agonist | anti-HER2 |  | II | HER2-positive GC | HER2 inh (Tucatinib), anti-VEGFR2 (Ramucirumab), Chemotherapy | NCT05190445 |  | Recruiting |
|  |  |  |  |  | I | HER2-positive Solid Tumors | anti-PD-1 (Atezolizumab) | NCT03650348 |  | Active, not recruiting |
|  |  |  |  |  | I | HER2-positive Solid Tumors | - | NCT03330561 |  | Completed |
| CB307 | Crescendo Biologics | 4-1BB agonist | anti-PSMA | human serum albumin | I | Solid Tumors | - | NCT04839991 | POTENTIA | Recruiting |
| RG6076 (RO7227166 ) | Roche | 4-1BBL | anti-CD19 |  | I | NHL | CD3 x CD20 BiTE (Glofitamab), anti-CD20 (Obinutuzumab), anti-IL-6 (Tocilizumab) | NCT04077723 |  | Recruiting |
| RG7827 (RO7122290) | Roche | 4-1BBL | anti-FAP |  | I/II | CRC | CD3 x CEA BiTE (Cibisatamab), anti-CD20 (Obinutuzumab) | NCT04826003 |  | Recruiting |
|  |  |  |  |  | I/II | UC | anti-PD-1 (Atezolizumab) | NCT03869190 | MORPHEUS-UC | Recruiting |
| RG6189（RO7300490） | Roche | CD40 agonist | anti-FAP |  | I | Solid Tumors | anti-PD-1 (Atezolizumab) | NCT04857138 |  | Recruiting |
| MP0317 | Molecular Partners | CD40 agonist | anti-FAP |  | I | Solid Tumors | - | NCT05098405 |  | Recruiting |
| REGN5678 | Regeneron | CD28 agonist | anti-PSMA |  | I/II | PC | anti-PD-1 (Cemiplimab) | NCT03972657 |  | Recruiting |
| REGN5668 | Regeneron | CD28 agonist | anti-MUC16 |  | I/II | Ovarian Cancer | anti-PD-1 (Cemiplimab), CD3 x MUC16 BiTE (Ubamatamab) | NCT04590326 |  | Recruiting |
| REGN7075 | Regeneron | CD28 agonist | anti-EGFR |  | I/II | Solid Tumors | anti-PD-1 (Cemiplimab) | NCT04626635 | COMBINE-EGFR-1 | Recruiting |
|  |  | ***Co-stimulatory molecule x Co-stimulatory molecule*** | | | | | | | | |
| GEN1042 | Genmab, BioNTech | 4-1BB agonist | CD40 agonist |  | I/II | Solid Tumors | anti-PD-1 (Pembrolizumab), chemotherapy | NCT04083599 |  | Recruiting |
| ***Group II (blocking inhibitory molecules)*** | | | | | | | | | | |
|  |  | ***Inhibitory checkpoint x TAA*** | | | | | | | | |
| Fidasimtamab (IBI-315) | Hanmi Pharmaceutical, Innovent Biologics | anti-PD-1 | anti-HER2 |  | I | Solid Tumors | - | NCT04162327 |  | Recruiting |
| SSGJ-705 | Sunshine Guojian Pharmaceutical | anti-PD-1 | anti-HER2 |  | I | Solid Tumors | - | NCT05145179 |  | Not yet recruiting |
|  |  | ***Inhibitory checkpoint x Inhibitory checkpoint*** | | | | | | | | |
| SHR-2002 | Jiangsu Hengrui | anti-TIGIT | anti-CD112R |  | I | Solid Tumors | anti-PD-1 (Camrelizumab), anti-PD-L1 (SHR-1316), PD-L1xTFG-βRII bsAb (SHR-1701) | NCT05198817 |  | Enrolling by invitation |
|  |  |  |  |  | I | Solid Tumors | anti-PD-L1 (SHR-1316) | NCT05082545 |  | Recruiting |
| AGEN1777 | Agenus, Bristol-Myers Squibb | anti-TIGIT | Undisclosed (T/NK cell inhibitory receptor) |  | I | Solid Tumors | anti-PD-1 | NCT05025085 |  | Recruiting |
| Vudalimab (XmAb717) | Xencor | anti-PD-1 | anti-CTLA-4 |  | II | Anaplastic Thyroid Carcinoma | - | NCT05453799 |  | Not yet recruiting |
|  |  |  |  |  | II | Rare Cancers | - | NCT05337735 |  | Not yet recruiting |
|  |  |  |  |  | II | BTC | - | NCT05297903 |  | Recruiting |
|  |  |  |  |  | II | Gynecologic and Genitourinary Malignancies | - | NCT05032040 |  | Recruiting |
|  |  |  |  |  | II | PC | PARP inh (Olaparib), Chemotherapy | NCT05005728 |  | Recruiting |
|  |  |  |  |  | I | Solid Tumors | - | NCT03517488 | DUET-2 | Active, not recruiting |
| Lorigerlimab (MGD019) | MacroGenics | anti-PD-1 | anti-CTLA-4 |  | II | Cervical Cancer | - | NCT05475171 | TRACTION | Not yet recruiting |
|  |  |  |  |  | I | Solid Tumors | B7-H3 ADC (MGC018) | NCT05293496 |  | Recruiting |
|  |  |  |  |  | I | Solid Tumors | - | NCT03761017 |  | Active, not recruiting |
| Cadonilimab (AK104) | Akeso Biopharma | anti-PD-1 | anti-CTLA-4 |  | II | Ovarian Cancer | Chemotherapy | NCT05430906 |  | Not yet recruiting |
|  |  |  |  |  | I/II | CRC | - | NCT05426005 |  | Not yet recruiting |
|  |  |  |  |  | II | NSCLC | - | NCT05377658 |  | Not yet recruiting |
|  |  |  |  |  | II | HCC | multi-kinase inh (Lenvatinib), Chemotherapy | NCT05319431 |  | Not yet recruiting |
|  |  |  |  |  | II | ccCRC | multi-kinase inh (Axitinib) | NCT05256472 |  | Not yet recruiting |
|  |  |  |  |  | I/II | Solid Tumors | anti-CD47 (AK117), Chemotherapy | NCT05235542 |  | Not yet recruiting |
|  |  |  |  |  | III | Cervical Cancer | Radiation, Chemotherapy | NCT05235516 | AK104-305 | Not yet recruiting |
|  |  |  |  |  | II | Cervical Cancer | - | NCT05227651 |  | Not yet recruiting |
|  |  |  |  |  | II | NSCLC | Chemotherapy | NCT05215067 |  | Recruiting |
|  |  |  |  |  | I/II | Solid Tumors | anti-VEGFR2 (AK109) | NCT05142423 |  | Recruiting |
|  |  |  |  |  | II | Cervical Cancer | - | NCT05063916 |  | Recruiting |
|  |  |  |  |  | I | Solid Tumors | anti-TIGIT (AK127) | NCT05021120 |  | Not yet recruiting |
|  |  |  |  |  | III | GC, GEJC | Chemotherapy | NCT05008783 |  | Recruiting |
|  |  |  |  |  | I/II | GC, GEJC | anti-VEGFR2 (AK109), Chemotherapy | NCT04982276 |  | Recruiting |
|  |  |  |  |  | III | Cervical Cancer | anti-VEGF (Bevacizumab), Chemotherapy | NCT04982237 |  | Recruiting |
|  |  |  |  |  | II | Cervical Cancer | anti-VEGF (Bevacizumab), Chemotherapy | NCT04868708 |  | Active, not recruiting |
|  |  |  |  |  | II | HCC | multi-kinase inh (Lenvatinib) | NCT04728321 |  | Recruiting |
|  |  |  |  |  | I/II | NSCLC | Chemotherapy | NCT04647344 |  | Not yet recruiting |
|  |  |  |  |  | I/II | NSCLC | multi-kinase inh (Anlotinib) | NCT04646330 |  | Active, not recruiting |
|  |  |  |  |  | I | Solid Tumors | anti-CD73 (AK119) | NCT04572152 |  | Recruiting |
|  |  |  |  |  | II | GC, CRC | - | NCT04556253 |  | Not yet recruiting |
|  |  |  |  |  | II | Solid Tumors | - | NCT04547101 |  | Recruiting |
|  |  |  |  |  | II | NSCLC | multi-kinase inh (Anlotinib) | NCT04544644 |  | Not yet recruiting |
|  |  |  |  |  | I/II | HCC | multi-kinase inh (Lenvatinib) | NCT04444167 |  | Recruiting |
|  |  |  |  |  | I/II | T-cell Lymphoma | - | NCT04444141 |  | Recruiting |
|  |  |  |  |  | II | Cervical Cancer | - | NCT04380805 |  | Active, not recruiting |
|  |  |  |  |  | II | Nasopharyngeal Cancer | - | NCT04220307 |  | Not yet recruiting |
|  |  |  |  |  | I/II | Solid Tumors | - | NCT04172454 |  | Not yet recruiting |
|  |  |  |  |  | I/II | GC, GEJC | Chemotherapy | NCT03852251 |  | Recruiting |
|  |  |  |  |  | I | Solid Tumors | - | NCT03261011 |  | Recruiting |
| Erfonrilimab (KN046) | Alphamab | anti-PD-L1 | anti-CTLA-4 |  | I/II | Solid Tumors | multi-kinase inh (MAX-40279) | NCT05425602 |  | Not yet recruiting |
|  |  |  |  |  | II | NSCLC | multi-kinase inh (Axitinib) | NCT05420220 |  | Not yet recruiting |
|  |  |  |  |  | III | PDAC | - | NCT05149326 |  | Recruiting |
|  |  |  |  |  | II/III | NSCLC | multi-kinase inh (Lenvatinib), Chemotherapy | NCT05001724 |  | Recruiting |
|  |  |  |  |  | I/II | Solid Tumors | anti-ALK-1 (GT90001) | NCT04984668 |  | Recruiting |
|  |  |  |  |  | II | Thymic Carcinoma | - | NCT04925947 |  | Recruiting |
|  |  |  |  |  | I/II | PDAC | chemotherapy | NCT04324307 |  | Recruiting |
|  |  |  |  |  | I/II | Gastrointestinal Tumors | multi-kinase inh (Donafenib) | NCT04612712 |  | Recruiting |
|  |  |  |  |  | I/II | HCC | multi-kinase inh (Ningetinib) | NCT04601610 |  | Active, not recruiting |
|  |  |  |  |  | II | HCC | multi-kinase inh (Lenvatinib) | NCT04542837 |  | Recruiting |
|  |  |  |  |  | II | HER2-positive Solid Tumors | HER2 x HER2 bsAb (KN026) | NCT04521179 |  | Active, not recruiting |
|  |  |  |  |  | III | NSCLC | - | NCT04474119 |  | Active, not recruiting |
|  |  |  |  |  | II | Thymic Carcinoma | - | NCT04469725 |  | Recruiting |
|  |  |  |  |  | II | Breast Cancer | Chemotherapy | NCT04165993 |  | Active, not recruiting |
|  |  |  |  |  | II | NSCLC | Chemotherapy | NCT04054531 |  | Recruiting |
|  |  |  |  |  | I | HER2 Positive Solid Tumor | HER2 x HER2 bsAb (KN026) | NCT04040699 |  | Recruiting |
|  |  |  |  |  | II | HER2-positive Breast Cancer | anti-HER2 (Trastuzumab), Chemotherapy | NCT04034823 |  | Not yet recruiting |
|  |  |  |  |  | II | ESCC | Radiotherapy | NCT03927495 |  | Recruiting |
|  |  |  |  |  | II | ESCC | - | NCT03925870 |  | Recruiting |
|  |  |  |  |  | I/II | TNBC | Chemotherapy | NCT03872791 |  | Active, not recruiting |
|  |  |  |  |  | II | NSCLC | - | NCT03838848 |  | Recruiting |
|  |  |  |  |  | I | Solid Tumors, Lymphoma | - | NCT03733951 |  | Recruiting |
|  |  |  |  |  | I | Solid Tumors | - | NCT03529526 |  | Recruiting |
| RG6139 (RO7247669) | Roche | anti-PD-1 | anti-LAG-3 |  | I/II | Melanoma | - | NCT05419388 |  | Recruiting |
|  |  |  |  |  | I/II | Melanoma | anti-TIGIT (Tiragolumab) | NCT05116202 |  | Recruiting |
|  |  |  |  |  | II | ESCC | - | NCT04785820 |  | Recruiting |
|  |  |  |  |  | I/II | HCC | anti-VEGF (Bevacizumab) | NCT04524871 | Morpheus-Liver | Recruiting |
|  |  |  |  |  | I | Solid Tumors | - | NCT04140500 |  | Recruiting |
| Tebotelimab (MGD013) | MacroGenics, Zai Lab | anti-PD-1 | anti-LAG-3 |  | II | HNSCC | anti-PD-1 (Retifanlimab), anti-B7-H3 (Enoblituzumab) | NCT04634825 |  | Terminated |
|  |  |  |  |  | II/III | GC, GEJC | anti-HER2 (Margetuximab), Chemotherapy | NCT04082364 | MAHOGANY | Active, not recruiting |
|  |  |  |  |  | I | Solid Tumors, Hematologic Neoplasms | anti-HER2 (Margetuximab) | NCT03219268 |  | Active, not recruiting |
| ABL501 | ABL Bio | anti-PD-L1 | anti-LAG-3 |  | I | Solid Tumors | - | NCT05101109 |  | Recruiting |
| FS118 | F-star Therapeutics | anti-PD-L1 | anti-LAG-3 |  | I/II | Solid Tumors | - | NCT03440437 |  | Recruiting |
| IBI-323 | Innovent Biologics | anti-PD-L1 | anti-LAG-3 |  | I | Solid Tumors | - | NCT04916119 |  | Recruiting |
| NGM707 | NGM Biopharmaceuticals | anti-LILRB1 | anti-LILRB2 |  | I/II | Solid Tumors | anti-PD-1 (Pembrolizumab) | NCT04913337 |  | Recruiting |
|  |  | ***inhibitory checkpoint x other inhibitory molecule*** | | | | | | | | |
| HX009 | Waterstone Hanxbio | anti-PD-1 | anti-CD47 |  | I/II | Lymphoma | - | NCT05189093 |  | Recruiting |
|  |  |  |  |  | II | Solid Tumors | - | NCT04886271 |  | Active, not recruiting |
|  |  |  |  |  | I | Solid Tumors | - | NCT04097769 |  | Active, not recruiting |
| Simridarlimab (IBI-322) | Innovent Biologics | anti-PD-L1 | anti-CD47 |  | II | SCLC | multi-kinase inh (Lenvatinib) | NCT05296603 |  | Not yet recruiting |
|  |  |  |  |  | II | NSCLC | multi-kinase inh (Lenvatinib), Chemotherapy | NCT05296278 |  | Not yet recruiting |
|  |  |  |  |  | I | AML, MDS | Chemotherapy | NCT05148442 |  | Not yet recruiting |
|  |  |  |  |  | I | Solid Tumors | - | NCT04912466 |  | Not yet recruiting |
|  |  |  |  |  | I | Hematologic Neoplasms | - | NCT04795128 |  | Recruiting |
|  |  |  |  |  | I | Solid Tumors, Lymphoma | - | NCT04338659 |  | Recruiting |
|  |  |  |  |  | I | Solid Tumors | - | NCT04328831 |  | Recruiting |
| LBL-015 | Nanjing Leads Biolabs | anti-PD-1 | TGF-βRII |  | I/II | Solid Tumors | - | NCT05107011 |  | Recruiting |
| Retlirafusp alfa (SHR-1701) | Jiangsu Hengrui | anti-PD-L1 | TGF-βRII |  | II | Rectal Cancer | Chemotherapy | NCT05300269 |  | Recruiting |
|  |  |  |  |  | I | Solid Tumors | TIGIT x CD112R bsAb (SHR-2002), anti-PD-1 (Camrelizumab), anti-PD-L1 (SHR-1316) | NCT05198817 |  | Enrolling by invitation |
|  |  |  |  |  | III | Cervical Cancer | anti-VEGF (Bevacizumab), Chemotherapy | NCT05179239 |  | Recruiting |
|  |  |  |  |  | II | NSCLC | - | NCT05177497 |  | Not yet recruiting |
|  |  |  |  |  | II/III | GC, GEJC | - | NCT05149807 |  | Enrolling by invitation |
|  |  |  |  |  | III | NSCLC | anti-VEGF (Bevacizumab), Chemotherapy | NCT05132413 |  | Not yet recruiting |
|  |  |  |  |  | II | Melanoma | Chemotherapy | NCT05106023 |  | Not yet recruiting |
|  |  |  |  |  | I | Solid Tumors | Target undisclosed (HRS2300) | NCT05048134 |  | Recruiting |
|  |  |  |  |  | I/II | Nasopharyngeal Carcinoma | multi-kinase inh (Famitinib) | NCT05020925 |  | Not yet recruiting |
|  |  |  |  |  | II | NSCLC | anti-VEGF (Bevacizumab) | NCT04974957 |  | Not yet recruiting |
|  |  |  |  |  | III | GC, GEJC | Chemotherapy | NCT04950322 |  | Recruiting |
|  |  |  |  |  | II | NSCLC | PARP inh (Fluzoparib) | NCT04937972 |  | Recruiting |
|  |  |  |  |  | II | SCLC | multi-kinase inh (Famitinib) | NCT04884009 |  | Not yet recruiting |
|  |  |  |  |  | II/III | CRC | anti-VEGF (Bevacizumab), Chemotherapy | NCT04856787 |  | Recruiting |
|  |  |  |  |  | I/II | Solid Tumors | anti-VEGF (Bevacizumab) | NCT04856774 |  | Recruiting |
|  |  |  |  |  | II | NSCLC | multi-kinase inh (Famitinib) | NCT04699968 |  | Not yet recruiting |
|  |  |  |  |  | I/II | Solid Tumors | multi-kinase inh (Famitinib) | NCT04679038 |  | Recruiting |
|  |  |  |  |  | II | HNSCC | - | NCT04650633 |  | Recruiting |
|  |  |  |  |  | I/II | Pancreatic Cancer | Chemotherapy | NCT04624217 |  | Active, not recruiting |
|  |  |  |  |  | II | NSCLC | Chemotherapy | NCT04580498 |  | Not yet recruiting |
|  |  |  |  |  | II | NSCLC | Radiotherapy | NCT04560244 |  | Not yet recruiting |
|  |  |  |  |  | I/II | Solid Tumors, Lymphomas | EZH2 inh (SHR-2554) | NCT04407741 |  | Recruiting |
|  |  |  |  |  | II | Breast Cancer | anti-PD-1 (Camrelizumab), anti-VEGF (Bevacizumab), multi-kinase inh (Famitinib), CDK4/6 inh (Dalpiciclib), Chemotherapy | NCT04355858 | MULAN | Recruiting |
|  |  |  |  |  | I | Solid Tumors | - | NCT04324814 |  | Active, not recruiting |
|  |  |  |  |  | I | Nasopharyngeal Carcinoma | Chemotherapy | NCT04282070 |  | Active, not recruiting |
|  |  |  |  |  | I | Solid Tumors | - | NCT03774979 |  | Recruiting |
|  |  |  |  |  | I | Solid Tumors | - | NCT03710265 |  | Recruiting |
| BJ-005 | BJ Bioscience | anti-PD-L1 | TGF-βRII |  | I | Solid Tumors, Lymphoma | - | NCT05115292 |  | Recruiting |
| TST005 | Transcenta Holding | anti-PD-L1 | TGF-βRII |  | I | Solid Tumors | - | NCT04958434 |  | Recruiting |
|  |  | ***Other inhibitory molecule x TAA*** | | | | | | | | |
| BCA101 | Bicara Therapeutics | TGF-β-trap | anti-EGFR |  | I/II | Solid Tumors | anti-PD-1 (Pembrolizumab) | NCT04429542 |  | Recruiting |
|  |  | ***Inhibitory checkpoint x Anti-angiogenesis*** | | | | | | | | |
| Ivonescimab (AK112) | Akeso Biopharma | anti-PD-1 | anti-VEGF |  | II | HCC | - | NCT05432492 |  | Not yet recruiting |
|  |  |  |  |  | II | CRC | - | NCT05382442 |  | Not yet recruiting |
|  |  |  |  |  | II | NSCLC | - | NCT05247684 |  | Not yet recruiting |
|  |  |  |  |  | I/II | Solid Tumors | - | NCT05229497 |  | Recruiting |
|  |  |  |  |  | II | TNBC | - | NCT05227664 |  | Recruiting |
|  |  |  |  |  | I/II | Solid Tumors | - | NCT05214482 |  | Recruiting |
|  |  |  |  |  | III | NSCLC | - | NCT05184712 |  | Recruiting |
|  |  |  |  |  | I | SCLC | Chemotherapy | NCT05116007 |  | Recruiting |
|  |  |  |  |  | I/II | Ovarian Cancer | PARP inh (Olaparib) | NCT04999605 |  | Recruiting |
|  |  |  |  |  | I/II | NSCLC | - | NCT04900363 |  | Recruiting |
|  |  |  |  |  | II | Gynecological Cancer | - | NCT04870177 |  | Recruiting |
|  |  |  |  |  | II | NSCLC | anti-PD-1 (Penpulimab), Chemotherapy | NCT04736823 |  | Recruiting |
|  |  |  |  |  | I/II | Solid Tumors | - | NCT04597541 |  | Not yet recruiting |
|  |  |  |  |  | I | Solid Tumors | - | NCT04047290 |  | Recruiting |
| ***Group III (targeting of co-stimulatory and inhibitory molecules)*** | | | | | | | | | | |
|  |  | ***Inhibitory checkpoint x Co-stimulatory molecule*** | | | | | | | | |
| Izuralimab (XmAb104) | Xencor | anti-PD-1 | ICOS agonist |  | I | Solid Tumors | anti-CTLA-4 (Ipilimumab) | NCT03752398 | DUET-3 | Recruiting |
| CDX-527 | Celldex Therapeutics | anti-PD-1 | CD27 agonist |  | I | Solid Tumors | - | NCT04440943 |  | Recruiting |
| PRS-344 (S095012) | Pieris Pharmaceuticals, Servier | anti-PD-L1 | 4-1BB agonist |  | I/II | Solid Tumors | - | NCT05159388 |  | Recruiting |
| LBL-024 | Nanjing Leads Biolabs | anti-PD-L1 | 4-1BB agonist |  | I/II | Solid Tumors | - | NCT05170958 |  | Recruiting |
| FS222 | F-star Therapeutics | anti-PD-L1 | 4-1BB agonist |  | I | Solid Tumors | - | NCT04740424 |  | Recruiting |
| MCLA-145 | Merus, Incyte Corporation | anti-PD-L1 | 4-1BB agonist |  | I | Solid Tumors, B-cell Lymphoma | - | NCT03922204 |  | Recruiting |
| ABL503 (TJ-L14B) | ABL Bio, I-MAB Biopharma | anti-PD-L1 | 4-1BB agonist |  | I | Solid Tumors | - | NCT04762641 |  | Recruiting |
| DSP107 | KAHR Medical | SIRPα | 4-1BBL |  | I/II | Solid Tumors | anti-PD-1 (Atezolizumab) | NCT04440735 |  | Recruiting |
|  |  |  |  |  | I | AML, MDS, CML | Chemotherapy | NCT04937166 |  | Recruiting |
|  |  | ***Inhibitory checkpoint x Stimulatory cytokine (immunocytokine)*** | | | | | | | | |
| AMG 256 | Amgen | anti-PD-1 | IL-21 mutein |  | I | Solid Tumors | - | NCT04362748 |  | Recruiting |
| RG6279 (RO7284755) | Roche | anti-PD-1 | IL-2v |  | I | Solid Tumors | - | NCT04303858 |  | Recruiting |
| SAR445710 (KD033) | Kadmon Holdings, Sanofi | anti-PD-L1 | IL-15 | IL-15RA | I | Solid Tumors | - | NCT04242147 |  | Recruiting |
| GI-101 | GI Innovation, Simcere Pharmaceutical | CD80 (CTLA-4 trap) | IL-2v |  | I/II | Solid Tumors | anti-PD-1 (Pembrolizumab), multi-kinase inh (Lenvatinib), Radiation | NCT04977453 |  | Recruiting |

Annotation:

1. Cells filled with blue color for drug names indicate that the corresponding drug has been approved by the FDA.
2. For drugs approved by FDA, owing to its proven efficacy and the abundance of the clinical trials, only one representative trial is listed.

ALL acute lymphoblastic leukemia, MM multiple myeloma, FL follicular lymphoma, AML acute myeloid leukemia, MDS myelodysplastic syndrome, CLL chronic lymphocytic leukemia, NHL non-Hodgkin lymphoma, DLBCL diffuse large B cell lymphoma, SCLC small-cell lung cancer, PC prostate cancer, CRC colorectal cancer, NSCLC non-small cell lung cancer, HCC hepatocellular carcinoma, HNSCC head and neck squamous cell carcinoma, HL Hodgkin lymphoma, GC gastric cancer, UC urothelial carcinoma, BTC biliary tract cancer, ccCRC clear cell colorectal cancer, GEJC gastroesophageal junction cancer, PDAC pancreatic ductal adenocarcinoma, ESCC esophageal squamous-cell carcinoma, TNBC triple-negative breast cancer, CML chronic myeloid leukemia.

**Supplementary Table 5. Therapeutics targeting immuno-epigenetics**

| Drug name | Manufacturer | Type of agent | Phase | Disease Area (Selected Indications) | Therapeutic combination | Identifier | | Status |
| --- | --- | --- | --- | --- | --- | --- | --- | --- |
|  |  |  |  |  |  | Trial number | Trial Name |  |
| ***Targeting DNMTs (hypomethylating agents)*** | | | | | | | | |
| ***Targeting DNMTs (hypomethylating agents)*** | | | | | | | | |
| guadecitabine (SGI-110) | Astex Pharmaceuticals | small-molecule DNA methylation inh (prodrug of decitabine) | I/II | Kidney Cancer | anti-PD-L1 (Durvalumab) | NCT03308396 |  | Active, not recruiting |
|  |  |  | II | Fallopian Tube Carcinoma, Ovarian Carcinoma, Primary Peritoneal Carcinoma | anti-PD-1 (Pembrolizumab) | NCT02901899 |  | Unknown status |
|  |  |  | II | Melanoma, NSCLC | anti-CTLA-4 (Ipilimumab), anti-PD-1 (nivolumab) | NCT04250246 | NIBIT-ML1 | Not yet recruiting |
|  |  |  | I/II | CMML, Myelodysplastic Syndrome | anti-PD-1 (Atezolizumab) | NCT02935361 |  | Active, not recruiting |
|  |  |  | I | Lung Cancer | anti-PD-1 (Pembrolizumab), HDAC inh (Mocetinostat) | NCT03220477 |  | Active, not recruiting |
|  |  |  | I | Liver cancer, Pancreatic cancer, Bile Duct Cancer, Gallbladder Cancer | anti-PD-L1 (Durvalumab) | NCT03257761 |  | Active, not recruiting |
|  |  |  | I | Melanoma | anti-CTLA-4 (Ipilimumab) | NCT02608437 | NIBIT-M4 | Unknown status |
|  |  |  | I | CRPC, NSCLC | anti-PD-1 (Pembrolizumab) | NCT02998567 | HyPeR | Active, not recruiting |
|  |  |  | II | UC | anti-PD-1 (Atezolizumab) | NCT03179943 |  | Active, not recruiting |
|  |  |  | I | SCLC | anti-PD-L1 (Durvalumab), anti-CTLA-4 (Tremelimumab) | NCT03085849 |  | Completed |
|  |  |  | I/II | Fallopian Tube Carcinoma, Ovarian Carcinoma, Primary Peritoneal Carcinoma | anti-PD-1 (Atezolizumab), Cancer vaccine (CDX-1401) | NCT03206047 |  | Active, not recruiting |
|  |  |  | I | CRC | cancer vaccine (GVAX), Cyclophosphamide | NCT01966289 |  | Completed |
|  |  |  | I | AML | anti-PD-1 (Atezolizumab) | NCT02892318 |  | Completed |
| ASTX727 (E7727) | Astex Pharmaceuticals | small-molecule DNA methylation inh (oral decitabine) | I/II | Head and Neck Cancer | anti-PD-L1 (Durvalumab) | NCT03019003 |  | Active, not recruiting |
|  |  |  | I | TNBC | anti-PD-1 (Pembrolizumab), chemotherapy | NCT05673200 |  | Recruiting |
|  |  |  | I | B-cell lymphoma | anti-PD-1 (Nivolumab) | NCT05272384 |  | Recruiting |
|  |  |  | II | MDS | anti-CD47 (Magrolimab) | NCT05835011 |  | Not yet recruiting |
|  |  |  | II | MDS | anti-TIM3 (Sabatolimab) | NCT04878432 |  | Recruiting |
|  |  |  | II | MDS, CMML | anti-PD-1 (Spartalizumab), anti-TIM3 (Sabatolimab) | NCT05201066 |  | Recruiting |
|  |  |  | I/II | Melanoma | anti-PD-1 (Nivolumab) | NCT05089370 |  | Recruiting |
|  |  |  | I/II | MDS | Cytokine (BMS-986253) | NCT05148234 |  | Recruiting |
| Decitabine | Janssen-Cilag/Otsuka Pharmaceutical | small-molecule DNA methylation inh | I/II | AML | anti-PD-1 (Pembrolizumab) | NCT02996474 |  | Completed |
|  |  |  | Early I | Solid tumors, Lymphoma | anti-PD-1 (Pembrolizumab), radiation | NCT03445858 |  | Active, not recruiting |
|  |  |  | II | ESCC | anti-PD-1 (Penpulimab) | NCT05265962 |  | Not yet recruiting |
|  |  |  | II/III | HL | anti-PD-1 (Camrelizumab) | NCT04510610 |  | Recruiting |
|  |  |  | II | HL | anti-PD-1 (Camrelizumab) | NCT03250962 |  | Recruiting |
|  |  |  | I | MDS, AML | anti-CTLA-4 (Ipilimumab) | NCT02890329 |  | Active, not recruiting |
|  |  |  | II | HL | anti-PD-1 (Tislelizumab) | NCT05137886 |  | Recruiting |
|  |  |  | I/II | GI cancers | anti-PD-L1 (TQB2450), multi-kinase inh (Anlotinib) | NCT04611711 |  | Unknown status |
|  |  |  | II | AML | anti-PD-1 (Camrelizumab) | NCT04353479 |  | Unknown status |
|  |  |  | I | PTCL, CTCL | anti-PD-1 (Pembrolizumab), chemotherapy | NCT03240211 | EMBOLDEN | Recruiting |
|  |  |  | I | B-cell lymphoma | Cell therapy (CD19 PD-1/CD28 CAR-T) | NCT04850560 |  | Recruiting |
|  |  |  | II | HL | HDAC inh (Chidamide), anti-PD-1 (Camrelizumab) | NCT04514081 |  | Recruiting |
|  |  |  | I | MDS, AML | anti-PD-1 (Spartalizumab), anti-TIM3 (Sabatolimab) | NCT03066648 |  | Active, not recruiting |
|  |  |  | I/II | NHL | HDAC inh (Chidamide), anti-PD-1 (Camrelizumab) | NCT04337606 |  | Recruiting |
|  |  |  | I/II | Solid Tumors, B-cell Lymphoma | Cell therapy (cytokine-induced killer cell) | NCT01799083 | CIK | Unknown status |
|  |  |  | II | HL | HDAC inh (Chidamide), anti-PD-1 (Camrelizumab) | NCT04233294 |  | Recruiting |
|  |  |  | I | AML | Cell therapy (natural killer cell therapy), cytokine (aldesleukin) | NCT02316964 |  | Completed |
|  |  |  | I | AML | anti-PD-1 (Nivolumab), BCL-2 inh (Venetoclax) | NCT04277442 |  | Active, not recruiting |
|  |  |  | I/II | B-cell NHL | Cell therapy (Decitabine-primed Tandem CAR19/20 engineered T cells), chemotherapy | NCT04697940 |  | Recruiting |
|  |  |  | I/II | PMBCL | anti-PD-1 (Camrelizumab), chemotherapy | NCT03346642 |  | Unknown status |
|  |  |  | II | NK/T-cell Lymphoma | anti-PD-1 (Sintilimab) | NCT04279379 |  | Unknown status |
|  |  |  | I/II | B-cell NHL | HDAC inh (Chidamide), cell therapy (Decitabine-primed Tandem CAR19/20 engineered T cells) | NCT04553393 |  | Unknown status |
|  |  |  | I | MDS, AML | anti-PD-1 (Pembrolizumab), BCL-2 inh (Venetoclax) | NCT03969446 |  | Recruiting |
|  |  |  | I/II | Malignancies | Anti-PD-1 antibody | NCT02961101 |  | Unknown status |
|  |  |  | II | Breast Cancer | anti-PD-1 (Pembrolizumab), chemotherapy | NCT02957968 |  | Active, not recruiting |
|  |  |  | II | ESCC | anti-PD-1 (Tislelizumab) | NCT05638984 |  | Not yet recruiting |
|  |  |  | I/II | NHL, Solid Tumors | HDAC inh (Chidamide), Immune checkpoint inhibitors | NCT05320640 |  | Recruiting |
|  |  |  | I | Fallopian Tube Carcinoma, Ovarian Carcinoma, Primary Peritoneal Carcinoma | Cytokine (Aldesleukin), chemotherapy, cell therapy (Genetically Engineered NY-ESO-1-specific T Lymphocytes) | NCT03017131 |  | Active, not recruiting |
|  |  |  | I | MDS, AML | Cancer vaccine (CDX-1401), anti-PD-1 (Nivolumab), immunostimulant (Poly ICLC) | NCT03358719 |  | Completed |
|  |  |  | I | MDS, AML | Cancer vaccine (CDX-1401), immunostimulant (Poly ICLC) | NCT01834248 |  | Completed |
|  |  |  | II | NSCLC | anti-PD-1 (Nivolumab), chemotherapy | NCT02664181 | PRECISE | Active, not recruiting |
|  |  |  | II | MDS | HDAC inh (Vorinostat), cytokine (Interleukin-2), cell therapy (Natural killer cells) | NCT01593670 |  | Completed |
|  |  |  | I | Neuroblastoma, Sarcoma | cancer vaccine (Autologous dendritic cell vaccine with adjuvant) | NCT01241162 |  | Completed |
|  |  |  | I/II | Malignancies | cell therapy (NK cell infusion) | NCT05143125 |  | Recruiting |
|  |  |  | I | AML | cancer vaccine (DC/AML fusion cells) | NCT03679650 |  | Recruiting |
|  |  |  | I | Fallopian Tube Cancer, Ovarian Epithelial Cancer, Primary Peritoneal Cancer | cancer vaccine (NY-ESO-1 peptide vaccine), chemotherapy | NCT01673217 |  | Completed |
|  |  |  | III | Malignancies | cancer vaccine (DC-CIK) | NCT04292769 |  | Unknown status |
|  |  |  | I/II | Malignancies | chemotherapy, cell therapy (TGFbDNRII-transduced Autologous Tumor Infiltrating Lymphocytes) | NCT02650986 |  | Active, not recruiting |
|  |  |  | II | MDS | anti-TIM3 (Sabatolimab) | NCT04878432 |  | Recruiting |
|  |  |  | I/II | Malignancies | cell therapy (DC-CTL) | NCT04672473 |  | Recruiting |
|  |  |  | I/II | AML | anti-CD47 (IBI188) | NCT04485052 |  | Suspended |
|  |  |  | I | MDS, AML | anti-TIM3 (TQB2618) | NCT05426798 |  | Recruiting |
|  |  |  | I | Myeloid Malignancies | anti-CD47/PD-L1 bsAb (IBI322) | NCT05148442 |  | Not yet recruiting |
|  |  |  | II | MDS, CMML | anti-PD-1 (Spartalizumab), anti-TIM3 (Sabatolimab), HDAC inh (Venetoclax) | NCT05201066 |  | Recruiting |
|  |  |  | II | MDS | anti-CD47 (Magrolimab) | NCT03946670 | STIMULUS-MDS1 | Active, not recruiting |
|  |  |  | I/II | T-cell lymphoma | anti-PD-L1 (Durvalumab), chemotherapy | NCT03161223 | DURABILITY | Recruiting |
|  |  |  | II | AML | anti-PD-1 (Tislelizumab) | NCT04541277 |  | Unknown status |
|  |  |  | I/II | MDS | anti-TLR2 (OPN-305) | NCT02363491 |  | Completed |
|  |  |  | Early I | HNSCC | anti-PD-1 (Nivolumab) | NCT05317000 |  | Recruiting |
|  |  |  | II | NSCLC | HDAC inh (Entinostat), anti-PD-1 (Nivolumab) | NCT01928576 | NA_00084192 | Completed |
|  |  |  | I | MDS | cancer vaccine (DC vaccine) | NCT04999943 | eDC-MDS | Recruiting |
|  |  |  | II | UC | 75 approved agents | NCT02788201 |  | Completed |
|  |  |  | I/II | AML | cell therapy (cytokine-induced memory-like NK Cell Infusion) | NCT03068819 |  | Recruiting |
|  |  |  | II | MDS | anti-CTLA-4 (Ipilimumab), andti-PD-1 (Nivolumab) | NCT02530463 |  | Active, not recruiting |
|  |  |  | I | AML | HDAC inh (Venetoclax), cell therapy (NK Cells) | NCT05834244 |  | Not yet recruiting |
|  |  |  | II | HL | anti-PD-1 (Pembrolizumab) | NCT05355051 |  | Recruiting |
| CC-486 | Pfizer | small-molecule DNA methylation inh (oral azacytidine) | II | NSCLC | anti-PD-1 (Pembrolizumab) | NCT02546986 |  | Active, not recruiting |
|  |  |  | I | HL | anti-PD-1 (Nivolumab) | NCT05162976 |  | Recruiting |
|  |  |  | II | MDS | anti-PD-L1 (Durvalumab) | NCT02281084 |  | Active, not recruiting |
|  |  |  | II | Melanoma | anti-PD-1 (Pembrolizumab) | NCT02816021 |  | Active, not recruiting |
|  |  |  | I | CRC | HDAC inh (Romidepsin), anti-PD-1 (Pembrolizumab) | NCT02512172 |  | Completed |
|  |  |  | II | Myeloid Malignancies | anti-CD47 (Magrolimab) | NCT04778410 |  | Active, not recruiting |
| azacytidine (5-aza, azacitidine) | Pfizer | small-molecule DNA methylation inh | II | PTCL | Anti-PD-1 monoclonal antibody, IMiD (Lenalidomide) | NCT05182957 |  | Recruiting |
|  |  |  | I/II | MDS, AML | anti-CD47 (IMM01) | NCT05140811 |  | Recruiting |
|  |  |  | Early I | HNSCC | anti-PD-1 (Nivolumab) | NCT05317000 |  | Recruiting |
|  |  |  | I/II | MDS, AML | anti-PD-L1/CTLA-4 bsAb (MAX-40279-01) | NCT05061147 |  | Recruiting |
|  |  |  | Not Applicable | AML | anti-PD-1 (Camrelizumab) | NCT05772273 |  | Recruiting |
|  |  |  | I/II | B-cell NHL | anti-PD-1 (penpulimab), chemotherapy | NCT04897477 |  | Unknown status |
|  |  |  | I | MDS | anti-CD47 (IBI188) | NCT04485065 |  | Suspended |
|  |  |  | II | MDS | anti-TIM3 (sabatolimab), BCL-2 inh (venetoclax) | NCT04812548 | STIMULUS-MDS3 | Completed |
|  |  |  | II | HL | anti-PD-1 (Pembrolizumab) | NCT05355051 |  | Recruiting |
|  |  |  | I | MDS, AML | cancer vaccine (NPMW-peptide vaccine) | NCT02750995 | AZACTA | Completed |
|  |  |  | II | AML | anti-PD-1 (Pembrolizumab) | NCT03769532 | PEMAZA | Recruiting |
|  |  |  | III | MDS, CMML | anti-TIM3 (sabatolimab) | NCT04266301 | STIMULUS-MDS2 | Active, not recruiting |
|  |  |  | I | MDS | anti-PD-1 (Atezolizumab) | NCT02508870 |  | Completed |
|  |  |  | II | AML | anti-TIM3 (sabatolimab), BCL-2 inh (venetoclax) | NCT04150029 | STIMULUS-AML1 | Active, not recruiting |
|  |  |  | II | MDS | anti-PD-1 (Pembrolizumab) | NCT03094637 |  | Active, not recruiting |
|  |  |  | I | MDS | anti-CD47 (IBI188) | NCT04511975 |  | Suspended |
|  |  |  | II | Pancreatic Cancer | anti-PD-1 (Pembrolizumab) | NCT03264404 |  | Active, not recruiting |
|  |  |  | II | Melanoma | anti-PD-1 (Pembrolizumab) | NCT02816021 |  | Active, not recruiting |
|  |  |  | II | CRC | anti-PD-1 (Pembrolizumab) | NCT02260440 |  | Completed |
|  |  |  | II | CRC, ovarian cancer, breast cancer | anti-PD-L1 (Durvalumab) | NCT02811497 | METADUR | Completed |
|  |  |  | II | MDS, AML | anti-PD-L1 (Durvalumab) | NCT02775903 |  | Completed |
|  |  |  | I/II | AML | anti-CD47 (Magrolimab) | NCT04435691 |  | Recruiting |
|  |  |  | I/II | AML | anti-PD-1 (Nivolumab) | NCT03825367 |  | Active, not recruiting |
|  |  |  | II | AML | anti-CTLA-4 (Ipilimumab), anti-PD-1 (Nivolumab) | NCT02397720 |  | Recruiting |
|  |  |  | III | MDS | anti-CD47 (Magrolimab) | NCT04313881 | ENHANCE | Active, not recruiting |
|  |  |  | II | AML | anti-PD-1 (Nivolumab), anti-LAG-3 (Relatlimab) | NCT04913922 | AARON | Recruiting |
|  |  |  | I | Hematological Malignancies | anti-CD47 (Magrolimab) | NCT03248479 |  | Active, not recruiting |
|  |  |  | I | Melanoma, RCC | rIFNα-2b | NCT00217542 |  | Completed |
|  |  |  | I | MDS, AML | anti-CD47 (Lemzoparlimab) | NCT04912063 |  | Completed |
|  |  |  | I/II | T-Cell lymphoma | anti-PD-L1 (Durvalumab), chemotherapy | NCT03161223 |  | Recruiting |
|  |  |  | I | AML, CMML | anti-LILRB4 (IO-202), BCL-2 inh (Venetoclax) | NCT04372433 |  | Recruiting |
|  |  |  | I/II | Osteosarcoma | anti-PD-1 (Nivolumab) | NCT03628209 |  | Recruiting |
|  |  |  | Not Applicable | AML | anti-CD47 mAb | NCT05266274 |  | Recruiting |
|  |  |  | I | AML | BCL-2 inh (Venetoclax), Cell therapy (NK Cells) | NCT05834244 |  | Not yet recruiting |
|  |  |  | II | NK/T Cell Lymphoma | Dexamethasone, Pegaspargase, anti-PD-1 (Tislelizumab) | NCT04899414 |  | Not yet recruiting |
|  |  |  | I | MDS, AML | anti-CD47 (SL-172154) | NCT05275439 |  | Recruiting |
|  |  |  | III | AML | anti-CD47 (Magrolimab) | NCT05079230 | ENHANCE-3 | Recruiting |
|  |  |  | II/III | MDS, AML | anti-PD-1 (Nivolumab) | NCT03092674 |  | Active, not recruiting |
|  |  |  | II | MDS | anti-CTLA-4 (Ipilimumab), anti-PD-1 (Nivolumab) | NCT02530463 |  | Active, not recruiting |
|  |  |  | II | PTCL | anti-PD-1 (Sintilimab), HDAC inh (Chidamide) | NCT04052659 |  | Not yet recruiting |
|  |  |  | III | AML | anti-CD47 (Magrolimab) | NCT04778397 | ENHANCE-2 | Recruiting |
|  |  |  | I | MDS | anti-PD-L1 (MEDI4736), anti-CTLA-4 (tremelimumab) | NCT02117219 |  | Completed |
|  |  |  | I | Melanoma | rIFNα-2b | NCT00398450 |  | Completed |
|  |  |  | I/II | AML | Anti-OX40 (PF-04518600), anti-PD-L1 (Avelumab)\|Drug: Azacitidine\|Drug: Gemtuzumab Ozogamicin\|Drug: Glasdegib\|Drug: Glasdegib Maleate\|Drug: Venetoclax | NCT03390296 |  | Completed |
|  |  |  | I/II | AML | anti-CD47 (AK117) | NCT04980885 |  | Recruiting |
|  |  |  | II | ENKTCL | anti-PD-1 (Sintilimab), HDAC inh (Chidamide), chemotherapy | NCT05008666 |  | Not yet recruiting |
|  |  |  | I/II | MDS | anti-CD47 (AK117) | NCT04900350 |  | Recruiting |
|  |  |  | I | MDS, AML, CMML | anti-CD47 (DSP107) | NCT04937166 |  | Recruiting |
|  |  |  | II | MDS, AML | anti-PD-1 (Pembrolizumab), BCL-2 inh (Venetoclax) | NCT04284787 |  | Active, not recruiting |
|  |  |  | II | AML | anti-PD-1 (pembrolizumab) | NCT02845297 |  | Completed |
|  |  |  | I | MDS, AML | anti-TIM3 (MBG453) | NCT03066648 |  | Active, not recruiting |
|  |  |  | I/II | MDS, AML | anti-TIM3 (Sabatolimab), anti-CD47 (Magrolimab) | NCT05367401 |  | Not yet recruiting |
|  |  |  | I/II | AML | anti-TIM3 (Sabatolimab) | NCT04623216 |  | Recruiting |
|  |  |  | I/II | MDS | Cell therapy (Allogeneic NK cell Transfusion) | NCT04599426 |  | Not yet recruiting |
|  |  |  | I/II | MDS, AML | anti-CD47 (TJ011133) | NCT04202003 |  | Active, not recruiting |
|  |  |  | II | NSCLC | HDAC inh (Entinostat), anti-PD-1 (Nivolumab) | NCT01928576 | NA_00084192 | Completed |
|  |  |  | II | MDS | anti-CD47 (MBG453) | NCT04878432 |  | Recruiting |
|  |  |  | I | HL, NHL | Cell therapy (TAA-Specific CTLs) | NCT01333046 | TACTAL | Active, not recruiting |
|  |  |  | I/II | Pancreatic Cancer | anti-PD-L1 (Durvalumab), IMiD (Lenalidomide), HDAC inh (Romidepsin), chemotherapy | NCT04257448 | SEPION | Active, not recruiting |
|  |  |  | I/II | AML | anti-CD47 (IBI188) | NCT04485052 |  | Suspended |
|  |  |  | I/II | Solid Tumors | IDH inh (FT-2102), anti-PD-1 (Nivolumab), chemotherapy | NCT03684811 |  | Completed |
|  |  |  | I | MDS, AML | anti-TIM3 (TQB2618) | NCT05426798 |  | Recruiting |
|  |  |  | Not Applicable | ENKTCL | anti-PD-1 (tislelizumab), IMiD (lenalidomide) | NCT05058755 |  | Recruiting |
|  |  |  | I | Myeloid Malignancies | anti-CD47/PD-L1 bsAb (IBI322) | NCT05148442 |  | Not yet recruiting |
|  |  |  | I/II | Melanoma, Head and Neck Cancer | Cell therapy (autologous MC2 TCR T cells) | NCT04729543 | MC2TCR | Recruiting |
|  |  |  | II | MDS | anti-CD47 (MBG453) | NCT03946670 | STIMULUS-MDS1 | Active, not recruiting |
|  |  |  | II | MDS, CMML | anti-CD47 (Sabatolimab), BCL-2 inh (Venetoclax) | NCT05201066 |  | Recruiting |
|  |  |  | Not Applicable | B-cell NHL | cell therapy (CD19/CD22 CAR-T), anti-CD33 (Obinutuzumab), anti-CD20 (Obinutuzumab), IMiD (Lenalidomide), chemotherapy | NCT05797948 |  | Enrolling by invitation |
|  |  |  | II | AML | anti-PD-1 (Tislelizumab) | NCT04541277 |  | Unknown status |
|  |  |  | I | MDS | cancer vaccine (DC vaccine) | NCT04999943 | eDC-MDS | Recruiting |
|  |  |  | II | Myeloid Malignancies | anti-CD47 (Magrolimab), BCL-2 inh (Venetoclax), chemotherapy | NCT04778410 |  | Active, not recruiting |
|  |  |  | I/II | AML | Cell therapy (Allogeneic NK cell Transfusion) | NCT04599452 |  | Not yet recruiting |
|  |  |  | II | UC | 75 approved agents | NCT02788201 |  | Completed |
| ***Targeting HDACs*** | | | | | | | | |
| ***Targeting class I HDACs*** | | | | | | | | |
| Romidepsin (FK228, Depsipeptide, FR 901228, NSC 630176) | Celgene Corporation | class I HDAC small molecule inhibitor | III | T-Cell Lymphoma | Aurora A Kinase inh (Alisertib), chemotherapy | NCT01482962 |  | Completed |
| Entinostat (SNDX-275, MS 275, MS 27-275, KHK2375) | Syndax Pharmaceuticals | class I HDAC small molecule inhibitor | I | Solid Tumors | - | NCT02897778 |  | Completed |
|  |  |  | I | Healthy Volunteers | - | NCT02922946 |  | Completed |
|  |  |  | I | Volunteers with/without Renal Impairment | - | NCT03192111 |  | Completed |
|  |  |  | II | Neuroendocrine Tumors | - | NCT03211988 |  | Recruiting |
|  |  |  | I/II | Solid Tumors | PD-L1 x TGFβ bsAb (Bintrafusp Alfa), IL-12 agonist (NHS-IL12) | NCT04708470 |  | Recruiting |
|  |  |  | I/II | Epithelial Ovarian Cancer, Peritoneal Cancer, Fallopian Tube Cancer | anti-PD-L1 (avelumab) | NCT02915523 |  | Completed |
|  |  |  | I | Breast Cancer | hormone therapy (Exemestane) | NCT02833155 |  | Completed |
|  |  |  | I/II | Solid Tumors | anti-PD-1 (Nivolumab) | NCT03838042 | INFORM2 NivEnt | Recruiting |
|  |  |  | II | RCC | anti-PD-1 (Nivolumab), anti-CTLA-4 (Ipilimumab) | NCT03552380 |  | Active, not recruiting |
|  |  |  | II | lymphoma | anti-PD-1 (Pembrolizumab) | NCT03179930 |  | Recruiting |
|  |  |  | I | Breast Cancer | chemotherapy | NCT03473639 | Breast49 | Recruiting |
|  |  |  | I | MDS | anti-PD-1 (Pembrolizumab) | NCT02936752 |  | Active, not recruiting |
|  |  |  | I | Healthy Volunteers | - | NCT02922933 |  | Completed |
|  |  |  | II | Melanoma | anti-PD-1 (Pembrolizumab) | NCT03765229 |  | Recruiting |
|  |  |  | I | Solid Tumors | - | NCT02780804 |  | Completed |
|  |  |  | II | RCC | IL-2 | NCT03501381 |  | Active, not recruiting |
|  |  |  | I/II | Solid Tumors | BET inh (ZEN-3694) | NCT05053971 |  | Not yet recruiting |
|  |  |  | I/II | Breast Cancer | anti-PD-L1 (atezolizumab) | NCT02708680 | ENCORE602 | Completed |
|  |  |  | I | Solid Tumors | anti-PD-1 (Pembrolizumab) | NCT02909452 |  | Completed |
|  |  |  | I | NSCLC, Breast Cancer | hormone therapy (Erlotinib, Exemestane) | NCT01594398 | ENCORE110 | Completed |
|  |  |  | I | Breast Cancer | hormone therapy (Exemestane) | NCT02820961 |  | Completed |
|  |  |  | I | SCLC | anti-PD-L1 (Atezolizumab), chemotherapy | NCT04631029 |  | Active, not recruiting |
|  |  |  | I | Breast Cancer | anti-CTLA-4 (Ipilimumab), anti-PD-1 (Nivolumab) | NCT02453620 |  | Active, not recruiting |
|  |  |  | III | Breast Cancer | hormone therapy (Exemestane) | NCT03538171 |  | Active, not recruiting |
|  |  |  | I | Breast Cancer | HER2/EGFR inh (Lapatinib), anti-HER2 (Trastuzumab) | NCT01434303 |  | Completed |
|  |  |  | II | AML | chemotherapy | NCT01305499 |  | Suspended |
|  |  |  | Early I | Endometrial Endometrioid Adenocarcinoma | - | NCT03018249 |  | Completed |
|  |  |  | I/II | Solid Tumors | anti-PD-1 (pembrolizumab) | NCT02437136 |  | Active, not recruiting |
|  |  |  | I/II | RCC | anti-PD-L1 (Atezolizumab), anti-VEGF (Bevacizumab) | NCT03024437 |  | Suspended |
|  |  |  | I/II | RCC | IL-2 agonist (Aldesleukin) | NCT01038778 |  | Active, not recruiting |
|  |  |  | III | Breast Cancer | hormone therapy (Exemestane, Goserelin) | NCT02115282 |  | Active, not recruiting |
|  |  |  | II | Breast Cancer | chemotherpay | NCT01349959 |  | Active, not recruiting |
|  |  |  | I | ALL, Acute Leukemias of Ambiguous Lineage | chemotherapy | NCT01132573 |  | Completed |
|  |  |  | II | Melanoma | anti-PD-1 (Pembrolizumab) | NCT02697630 | PEMDAC | Active, not recruiting |
|  |  |  | I/II | NSCLC | chemotherapy | NCT00387465 |  | Completed |
|  |  |  | II | CRC | chemotherapy | NCT01105377 |  | Completed |
|  |  |  | II | MDS,CMML, AML | chemotherapy | NCT00313586 |  | Completed |
|  |  |  | II | Cholangiocarcinoma, Pancreatic Cancer | anti-PD-1 (Nivolumab) | NCT03250273 |  | Completed |
|  |  |  | I | Solid Tumors | chemotherapy | NCT00098891 |  | Completed |
|  |  |  | II | Bladder Cancer | anti-PD-1 (Pembrolizumab) | NCT03978624 |  | Recruiting |
|  |  |  | I | MDS,CMML, AML | chemotherapy | NCT00101179 |  | Completed |
|  |  |  | I/II | CRC | chemotherapy, multi-kinase inh(regorafenib) | NCT03215264 |  | Completed |
|  |  |  | II | Breast Cancer | hormone therapy (Exemestane) | NCT00676663 | ENCORE301 | Completed |
|  |  |  | I | Solid Tumors | - | NCT00020579 |  | Completed |
|  |  |  | I | Hematological Malignancies | - | NCT00015925 |  | Completed |
|  |  |  | II | MDS, AML, ALL | GM-CSF (sargramostim) | NCT00462605 |  | Completed |
|  |  |  | II | Melanoma | - | NCT00185302 |  | Completed |
|  |  |  | II | NSCLC | chemotherapy, anti-PD-1 (Nivolumab) | NCT01928576 | NA_00084192 | Recruiting |
|  |  |  | II | Breast Cancer | hormone therapy (Exemestane) | NCT03291886 |  | Completed |
|  |  |  | II | Breast Cancer | Aromatase Inhibion | NCT00828854 |  | Completed |
|  |  |  | I/II | NSCLC | EGFR inh (Erlotinib) | NCT00602030 |  | Completed |
|  |  |  | II | NSCLC | EGFR inh (Erlotinib) | NCT00750698 |  | Completed |
|  |  |  | I | Breast Cancer | hormone therapy (Exemestane) | NCT02623751 |  | Completed |
|  |  |  | I/II | Breast Cancer | anti-PD-L1 (Atezolizumab) | NCT03280563 | MORPHEUS HR+BC | Recruiting |
|  |  |  | I | Healthy Volunteers | - | NCT03187015 |  | Completed |
| Mocetinostat (MGCD0103, MG-0103) | Mirati Therapeutics | class I HDAC small molecule inhibitor | II | UC | - | NCT02236195 |  | Completed |
|  |  |  | I/II | HL | anti-CD30 (Brentuximab) | NCT02429375 |  | Active, not recruiting |
|  |  |  | I/II | DLBCL, FL | - | NCT02282358 |  | Active, not recruiting |
|  |  |  | I | Rhabdomyosarcoma | chemotherapy | NCT04299113 |  | Recruiting |
|  |  |  | I/II | MDS | chemotherapy | NCT02018926 |  | Completed |
|  |  |  | I | Lung Cancer | anti-PD-1 (Pembrolizumab), chemotherapy | NCT03220477 |  | Active, not recruiting |
|  |  |  | II | Leiomyosarcoma | chemotherapy | NCT02303262 |  | Completed |
|  |  |  | II | NSCLC | anti-PD-1 (Nivolumab) | NCT02954991 |  | Completed |
|  |  |  | II | CLL | - | NCT00431873 |  | Completed |
|  |  |  | I | Leukemia, MDS | - | NCT00324194 |  | Completed |
|  |  |  | I/II | MDS, AML | - | NCT00324220 |  | Completed |
|  |  |  | II | lymphoma | - | NCT00359086 |  | Completed |
|  |  |  | I | Solid Tumors, NHL | - | NCT00323934 |  | Completed |
|  |  |  | I | Leukemia, MDS | - | NCT00324129 |  | Completed |
|  |  |  | I/II | Malignancies | chemotherapy | NCT00372437 |  | Completed |
| Domatinostat (4SC-202) | 4SC AG | class I HDAC small molecule inhibitor | I/II | Melanoma | anti-PD-1 (Pembrolizumab) | NCT03278665 | SENSITIZE | Completed |
|  |  |  | II | MCC | anti-PD-L1 (avelumab) | NCT04393753 | MERKLIN2 | Active, not recruiting |
|  |  |  | II | Gastrointestinal Cancer | anti-PD-L1 (Avelumab) | NCT03812796 | EMERGE | Recruiting |
|  |  |  | I | Hematologic Malignancies | - | NCT01344707 | TOPAS | Completed |
|  |  |  | I | UC | anti-PD-1 (Nivolumab), anti-CTLA-4 (Ipilimumab) | NCT04871594 | TURANDOT | Active, not recruiting |
|  |  |  | I/II | Melanoma | anti-PD-1 (Nivolumab), anti-CTLA-4 (Ipilimumab) | NCT04133948 | DONIMI | Active, not recruiting |
| Nanatinostat (Tractinostat, VRx-3996, CHR-3996) | Viracta Therapeutics | class I HDAC small molecule inhibitor | I/II | EBV-associated Lymphoma/Lymphoproliferative Disorders | anti-virus (Valganciclovir) | NCT03397706 |  | Recruiting |
|  |  |  | I/II | EBV-associated Nasopharyngeal Carcinoma | anti-PD-1 (Pembrolizumab), anti-virus (Valganciclovir) | NCT05166577 |  | Recruiting |
|  |  |  | II | EBV-associated Lymphoma | anti-virus (valganciclovir) | NCT05011058 | NAVAL-1 | Recruiting |
|  |  |  | I | Solid Tumors | - | NCT00697879 |  | Completed |
| OKI-179 | OnKure | class I HDAC small molecule inhibitor | I/II | Melanoma | MEK1/2 inh (binimetinib) | NCT05340621 |  | Recruiting |
|  |  |  | I | Solid Tumors | - | NCT03931681 |  | Completed |
| Zabinostat (CXD101) | Celleron Therapeutics | class I HDAC small molecule inhibitor | I | Solid Tumors, Lymphoma, myeloma | - | NCT01977638 |  | Active, not recruiting |
|  |  |  | I/II | CRC | anti-PD-1 (Nivolumab) | NCT03993626 | CAROSELL | Active, not recruiting |
| ***Targeting class II HDAC6*** | | | | | | | | |
| Ricolinostat (ACY-1215) | Regenacy Pharmaceuticals | HDAC6 small molecule inhibitor | I | Breast Cancer | chemotherapy | NCT02632071 |  | Completed |
|  |  |  | I | CLL | BTK inh (Ibrutinib), PI3K inh (Idelalisib) | NCT02787369 |  | Active, not recruiting |
|  |  |  | I | MM | IMiD (pomalidomide), chemotherapy | NCT02189343 |  | Completed |
|  |  |  | I/II | Lymphoma | - | NCT02091063 |  | Completed |
|  |  |  | I/II | MM | IMiD (pomalidomide), chemotherapy | NCT01997840 |  | Active, not recruiting |
|  |  |  | I | Healthy Volunteers | - | NCT02088398 |  | Completed |
|  |  |  | I | MM | IMiD (lenalidomide), chemotherapy | NCT01583283 |  | Completed |
|  |  |  | I/II | MM | - | NCT01323751 | ACY-1215 | Completed |
| Citarinostat (ACY-241) | Celgene Corporation | HDAC6 small molecule inhibitor | I | MM | IMiD (Pomalidomide), chemotherapy | NCT02400242 |  | Active, not recruiting |
|  |  |  | I | Melanoma | anti-PD-1 (nivolumab), anti-CTLA-4 (ipilimumab) | NCT02935790 |  | Completed |
|  |  |  | I | Solid Tumors | - | NCT02551185 |  | Completed |
|  |  |  | I | NSCLC | anti-PD-1 (Nivolumab) | NCT02635061 |  | Active, not recruiting |
|  |  |  | I | MM | TLR3/MDA5 agonist (Hiltonol), IMiD (Lenalidomide), cancer vaccine (PVX-410) | NCT02886065 |  | Recruiting |
| KA-2507 | Karus Therapeutics Limited | HDAC6 small molecule inhibitor | I | Solid Tumors | - | NCT03008018 |  | Completed |
| ***Targeting class I and II HDACs*** | | | | | | | | |
| Vorinostat (Suberoylanilide hydroxamic acid, Zolinza, L-001079038) | Merck & Co. | class I/II HDAC small molecule inhibitor | III | CTCL | anti-CCR4 (KW-0761) | NCT01728805 | MAVORIC | Completed |
| Tucidinostat (Chidamide, HBI-8000) | HUYA Bioscience International | class I/II HDAC small molecule inhibitor | II | NSCLC | anti-PD-1 (pembrolizumab) | NCT05141357 | HBI-8000 | Recruiting |
|  |  |  | II | EC, AEG, GC | anti-PD-1 (toripalimab) | NCT05163483 |  | Not yet recruiting |
|  |  |  | I | CRC | COX2 inh (Celecoxib) | NCT05281276 |  | Recruiting |
|  |  |  | I/II | Acute T Cell Lymphoblast Leukemia/Lymphoma | JAK1/2 inh (Ruxolitinib) | NCT05075681 |  | Recruiting |
|  |  |  | II | AML | chemotherapy | NCT05330364 |  | Recruiting |
|  |  |  | I/II | AML | stem cell transplantation, chemotherapy | NCT05270200 |  | Recruiting |
|  |  |  | II | DLBCL | - | NCT04661943 |  | Recruiting |
|  |  |  | Not Applicable | TNBC | chemotherapy | NCT04582955 |  | Recruiting |
|  |  |  | I/II | Breast Cancer | chemotherapy | NCT05335473 |  | Not yet recruiting |
|  |  |  | II | DLBCL | Anti-PD-1 Antibody, anti-CD20 (Rituximab) | NCT05115409 | PCR | Not yet recruiting |
|  |  |  | III | PTCL | chemotherapy | NCT05075460 |  | Not yet recruiting |
|  |  |  | I/II | PTCL | PI3Kδ inh (Parsaclisib) | NCT05083208 |  | Not yet recruiting |
|  |  |  | II | ENKTCL | anti-PD-1 (Sintilimab) | NCT04994210 |  | Recruiting |
|  |  |  | II | Bladder Cancer | anti-PD-1 (tislelizumab) | NCT04562311 |  | Recruiting |
|  |  |  | II | Angioimmunoblastic T-cell Lymphoma | anti-PD-1 (Sintilimab) | NCT04831710 |  | Not yet recruiting |
|  |  |  | II | Breast Cancer | hormone therapy (Exemestane, Ovarian function suppression) | NCT04465097 | NeoTEE | Recruiting |
|  |  |  | II | ESCC | anti-PD-1 (Camrelizumab) | NCT04984018 |  | Not yet recruiting |
|  |  |  | II | NK/T Cell Lymphoma | Anti-PD-1 Ab, chemotherapy | NCT04414969 |  | Recruiting |
|  |  |  | II | Neuroendocrine Tumors | chemotherapy | NCT05076786 |  | Recruiting |
|  |  |  | II | Angioimmunoblastic T-cell Lymphoma | chemotherapy | NCT05179213 |  | Not yet recruiting |
|  |  |  | II | Neuroendocrine Tumors | anti-PD-1 (Sintilimab) | NCT05113355 |  | Recruiting |
|  |  |  | II | NSCLC | anti-PD-L1 (Envafolimab) | NCT05068427 |  | Recruiting |
|  |  |  | IV | NK/T-Cell Lymphoma | chemotherapy | NCT04490590 |  | Recruiting |
|  |  |  | IV | Breast Cancer | hormone therapy (Fulvestrant) | NCT05191914 |  | Not yet recruiting |
|  |  |  | Not Applicable | ENKTCL | chemotherapy, radiation | NCT04511351 |  | Recruiting |
|  |  |  | II | PTCL | IMiD (Lenalidomide) | NCT04329130 |  | Recruiting |
|  |  |  | II/III | Breast Cancer | hormone therapy (exemestane), chemotherapy | NCT05253066 |  | Not yet recruiting |
|  |  |  | II | AML | chemotherapy | NCT05305859 |  | Not yet recruiting |
|  |  |  | II | TNBC | chemotherapy | NCT04192903 |  | Recruiting |
|  |  |  | II | Sarcoma | anti-PD-1 (toripalimab) | NCT04025931 |  | Recruiting |
|  |  |  | II | PTCL | PD-1 antibody | NCT04512534 | Sincerely20 | Recruiting |
|  |  |  | II | Breast Cancer | multi-kinase inh(surufatinib), hormone therapy (fulvestrant) | NCT05186545 |  | Not yet recruiting |
|  |  |  | II | Breast Cancer | hormone therapy (Fulvestrant) | NCT04999540 |  | Not yet recruiting |
|  |  |  | I/II | ENKTCL | anti-PD-1 (Sintilimab) | NCT03820596 | SCENT | Completed |
|  |  |  | II | DLBCL | anti-CD20 (Rituximab), chemotherapy | NCT04022005 |  | Recruiting |
|  |  |  | Not Applicable | Breast Cancer | hormone therapy (Fulvestrant) | NCT05047848 |  | Recruiting |
|  |  |  | IV | NK/T Cell Lymphoma | PD-1 Antibody, IMiD (lenalidomide), chemotherapy | NCT04038411 |  | Unknown status |
|  |  |  | II | CTCL | anti-PD-1 (Sintilimab) | NCT04296786 |  | Recruiting |
|  |  |  | I | Neuroblastoma | chemotherapy | NCT05338541 | CSIIT-Q36 | Not yet recruiting |
|  |  |  | III | PTCL | chemotherapy | NCT04668690 |  | Not yet recruiting |
|  |  |  | II | DLBCL, FL | - | NCT03410004 |  | Unknown status |
|  |  |  | II | Head and Neck Adenoid Cystic Carcinomas | chemotherapy | NCT03639168 |  | Completed |
|  |  |  | II | DLBCL | anti-CD20 (Rituximab), chemotherapy | NCT03373019 |  | Unknown status |
|  |  |  | II | DLBCL | anti-CD20 (Rituximab), chemotherapy | NCT03201471 | DLBCL | Unknown status |
|  |  |  | Not Applicable | PTCL | chemotherapy | NCT03268889 | CHOP | Unknown status |
|  |  |  | II | Central Nervous System Lymphoma | anti-CD20 (rituximab), chemotherapy | NCT04516655 | C-R-HDMTX | Not yet recruiting |
|  |  |  | II | PTCL | - | NCT02944812 |  | Unknown status |
|  |  |  | II | B-cell NHL | chemotherapy | NCT03105596 |  | Unknown status |
|  |  |  | II | B-cell NHL | - | NCT03245905 |  | Recruiting |
|  |  |  | III | Melanoma | anti-PD-1 (nivolumab) | NCT04674683 |  | Recruiting |
|  |  |  | I/II | MM | chemotherapy | NCT04025450 |  | Recruiting |
|  |  |  | IV | PTCL | PD-1 blocking antibody, IMiD (lenalidomide), chemotherapy | NCT04040491 |  | Unknown status |
|  |  |  | II | lymphoma | stem cell transplantation | NCT03611231 |  | Not yet recruiting |
|  |  |  | I/II | Solid Tumors | chemotherapy, Immune checkpoint inhs | NCT05320640 |  | Recruiting |
|  |  |  | II | EBV-assoicated Solid Tumors | - | NCT03494634 |  | Unknown status |
|  |  |  | II | PTCL | chemotherapy | NCT02856997 |  | Unknown status |
|  |  |  | II | Breast Cancer | PARP1 inh (fluzoparib) | NCT05085626 |  | Recruiting |
|  |  |  | II | HL | anti-PD-1 (Camrelizumab), chemotherapy | NCT04233294 |  | Recruiting |
|  |  |  | II | ENKTCL | - | NCT02878278 |  | Unknown status |
|  |  |  | II | NSCLC | multi-kinase inh(EGFR-TKI) | NCT02815007 |  | Unknown status |
|  |  |  | I/II | Melanoma, RCC, NSCLC | anti-PD-1 (nivolumab) | NCT02718066 |  | Active, not recruiting |
|  |  |  | II | Adenocystic Carcinoma | - | NCT02883374 |  | Unknown status |
|  |  |  | I/II | NHL | anti-PD-1 (Camrelizumab), chemotherapy | NCT04337606 |  | Recruiting |
|  |  |  | I | NHL | - | NCT02697552 |  | Completed |
|  |  |  | I/II | NHL | multi-kinase inh(Chiauranib) | NCT03974243 |  | Completed |
|  |  |  | I/II | Cervical Cancer | anti-PD-1 (Toripalimab) | NCT04651127 |  | Recruiting |
|  |  |  | II | Angioimmunoblastic T-cell Lymphoma | - | NCT03273452 | PET | Unknown status |
|  |  |  | II | HL | anti-PD-1 (Camrelizumab), chemotherapy | NCT04514081 |  | Recruiting |
|  |  |  | I/II | AML | chemotherapy | NCT03031262 |  | Recruiting |
|  |  |  | II | ENKTCL | - | NCT03630731 |  | Unknown status |
|  |  |  | II | PTCL | - | NCT02753543 |  | Unknown status |
|  |  |  | III | Breast Cancer | hormone therapy (exemestane) | NCT02482753 |  | Completed |
|  |  |  | Not Applicable | Angioimmunoblastic T-cell Lymphoma | anti-CD20 (Rituximab), IMiD (lenalidomide) | NCT04319601 | AITL | Recruiting |
|  |  |  | I/II | T Cell NHL | chemotherapy | NCT02987244 |  | Recruiting |
|  |  |  | I/II | AML | chemotherapy | NCT02886559 |  | Unknown status |
|  |  |  | II | CRC | anti-PD-1 (Sintilimab), anti-VEGF (bevacizumab) | NCT04724239 |  | Not yet recruiting |
|  |  |  | II | ENKTCL | JAK1/3 inh (tofacitinib) | NCT03598959 |  | Unknown status |
|  |  |  | III | DLBCL | anti-CD20 (Rituximab), chemotherapy | NCT04231448 | DEB | Recruiting |
|  |  |  | II | DLBCL | chemotherapy, IMiD (Thalidomide) | NCT02733380 |  | Unknown status |
|  |  |  | II | PTCL | anti-PD-1 (Sintilimab), chemotherapy | NCT04052659 |  | Not yet recruiting |
|  |  |  | II | T-Cell Lymphoma | - | NCT02955589 |  | Completed |
|  |  |  | II | PTCL | - | NCT02953652 |  | Active, not recruiting |
|  |  |  | II/III | ALL | chemotherapy, radiation | NCT03553238 |  | Unknown status |
|  |  |  | II | DLBCL | anti-CD20 (Rituximab), chemotherapy | NCT02753647 |  | Unknown status |
|  |  |  | II | PTCL | chemotherapy | NCT04480125 |  | Recruiting |
|  |  |  | I | PTCL | chemotherapy | NCT02809573 |  | Completed |
|  |  |  | II | NSCLC | chemotherapy | NCT01836679 |  | Completed |
|  |  |  | III | PTCL | chemotherapy | NCT03023358 |  | Unknown status |
|  |  |  | II/III | T cell lymphoblastic lymphoma/leukemia | chemotherapy, radiation | NCT03564704 |  | Unknown status |
|  |  |  | II | Lymphoma | Chemotherapy | NCT03602131 |  | Unknown status |
|  |  |  | II/III | ALL | multi-kinase inh(dasatinib) | NCT03564470 |  | Unknown status |
|  |  |  | II | PTCL | Chemotherapy | NCT02879526 |  | Unknown status |
|  |  |  | II | DLBCL | Chemotherapy | NCT03151876 |  | Unknown status |
|  |  |  | II | PTCL | Chemotherapy | NCT03617432 |  | Recruiting |
|  |  |  | II | PTCL | Chemotherapy | NCT03321890 |  | Unknown status |
|  |  |  | II | MM | IMiD (lenalidomide), dexamethasone | NCT03605056 |  | Not yet recruiting |
|  |  |  | II | T-cell Lymphoma | Chemotherapy | NCT03853044 |  | Active, not recruiting |
|  |  |  | II | AML | Chemotherapy | NCT03985007 |  | Completed |
|  |  |  | II | AML | anti-PD-1 (Sintilimab), chemotherapy | NCT05008666 |  | Not yet recruiting |
|  |  |  | II | AML | Chemotherapy | NCT05029141 |  | Recruiting |
|  |  |  | I/II | AML | Chemotherapy | NCT03453255 |  | Unknown status |
|  |  |  | I/II | B-cell NHL | Cell Therapy (Decitabine-primed Tandem CAR19/20 engineered T cells), chemotherapy | NCT04553393 |  | Recruiting |
|  |  |  | II | Lymphoma | chemotherapy | NCT03629873 |  | Active, not recruiting |
|  |  |  | I/II | NHL | Bcl2/Bcl-xL inh (APG-1252) | NCT05186012 |  | Not yet recruiting |
|  |  |  | II | DLBCL | chemotherapy | NCT05348213 | R-ICE+X | Not yet recruiting |
|  |  |  | II | NSCLC | anti-CD20 (Rituximab),HER2/EGFR inh (Afatinib), chemotherapy | NCT03574402 | TRUMP | Recruiting |
|  |  |  | II | PTCL | chemotherapy | NCT04480099 |  | Recruiting |
|  |  |  | II | DLBCL | anti-CD20 (Rituximab), Prednisone, BTK inh (Ibrutinib), IMiD (Lenalidomide), chemotherapy | NCT04025593 |  | Recruiting |
| ***Targeting class I, II and IV HDACs (pan-HDAC)*** | | | | | | | | |
| Panobinostat (LBH 589, Farydak, MTX110) | Novartis/Secura Bio | pan-HDAC small molecule inhibitor | III | MM | proteasome inh (bortezomib), Dexamethasone | NCT01023308 | PANORAMA-1 | Completed |
| Abexinostat (PCI 24781, CRA-24781) | Xynomic Pharmaceuticals | pan-HDAC small molecule inhibitor | I/II | DLBCL, MCL | BTK inh (Ibrutinib) | NCT03939182 |  | Recruiting |
|  |  |  | II | FL | - | NCT03934567 |  | Recruiting |
|  |  |  | II | FL | - | NCT03600441 | FORERUNNER | Active, not recruiting |
|  |  |  | II | DLBCL | - | NCT03936153 |  | Recruiting |
|  |  |  | III | RCC | multi-kinase inh(Pazopanib) | NCT03592472 | RENAVIV | Recruiting |
|  |  |  | I | Solid Tumors | anti-PD-1 (Pembrolizumab) | NCT03590054 |  | Recruiting |
|  |  |  | I/II | NHL | - | NCT04024696 |  | Recruiting |
|  |  |  | I/II | Sarcoma | chemotherapy, G-CSF | NCT01027910 |  | Completed |
|  |  |  | I | Hematological Malignancies | - | NCT01149668 |  | Completed |
|  |  |  | I | Solid Tumors | - | NCT01543763 |  | Recruiting |
|  |  |  | I/II | Lymphoma | - | NCT00724984 | PCYC-0403 | Completed |
|  |  |  | I | Hematological Malignancies | - | NCT00562224 |  | Completed |
|  |  |  | I | Hematological Malignancies | - | NCT00473577 |  | Completed |
| Pracinostat (SB-939) | MEI Pharma | pan-HDAC small molecule inhibitor | I | AML | ADC (Gemtuzumab Ozogamicin) | NCT03848754 | PraGO | Active, not recruiting |
|  |  |  | I | Healthy Volunteers | anti-fungal (Itraconazole), antibiotic (Ciprofloxacin) | NCT02118909 |  | Completed |
|  |  |  | II | MF | JAK1/2 inh (Ruxolitinib) | NCT02267278 |  | Completed |
|  |  |  | Early I | Healthy Volunteers | - | NCT02058784 | HVFE | Completed |
|  |  |  | II | AML | chemotherapy | NCT01912274 |  | Completed |
|  |  |  | II | MDS | chemotherapy | NCT01993641 | MEI-005 | Completed |
|  |  |  | II | MDS | chemotherapy | NCT01873703 |  | Completed |
|  |  |  | I | Healthy Volunteers | - | NCT03495934 |  | Completed |
|  |  |  | II | Sarcoma | - | NCT01112384 | IND200 | Completed |
|  |  |  | II | MPN | - | NCT01200498 |  | Completed |
|  |  |  | II | PC | - | NCT01075308 |  | Completed |
|  |  |  | I | Solid Tumors, Leukemia | - | NCT01184274 |  | Completed |
|  |  |  | I | Solid Tumors, Hematological Neoplasms | chemotherapy | NCT00741234 |  | Completed |
|  |  |  | I | Solid Tumors | - | NCT00504296 |  | Completed |
| Resminostat (4SC-201, RAS2410) | 4SC AG | pan-HDAC small molecule inhibitor | I | Healthy volunteers | - | NCT04955340 |  | Completed |
|  |  |  | II | T-Cell lymphoma | - | NCT02953301 | RESMAIN | Recruiting |
|  |  |  | I/II | CRC | chemotherapy | NCT01277406 | SHORE | Completed |
|  |  |  | II | HL | - | NCT01037478 | SAPHIRE | Completed |
|  |  |  | I/II | HCC | Raf inh (Sorafenib) | NCT02400788 |  | Completed |
|  |  |  | II | HCC | Raf inh (Sorafenib) | NCT00943449 | Shelter | Completed |
| ***Co-targeting HDAC and other molecules*** | | | | | | | | |
| Fimepinostat (CUDC-907) | Curis | PI3K/HDAC small molecule dual inhibitor | Early I | Brain Tumors | - | NCT03893487 | PNOC016 | Recruiting |
|  |  |  | I | Solid Tumors | - | NCT02307240 |  | Completed |
|  |  |  | I | Lymphoma | anti-CD20 (Rituximab), BCL2 inh (venetoclax) | NCT01742988 |  | Completed |
|  |  |  | I | Solid TumorS | - | NCT02909777 |  | Recruiting |
|  |  |  | II | DLBCL | - | NCT02674750 |  | Completed |
| ***Targeting BET family proteins*** | | | | | | | | |
| ***Targeting BET family proteins (pan-BET)*** | | | | | | | | |
| INCB057643 | Incyte Corporation | small molecule inhibitor | I | Myeloid Neoplasms | stem cell transplantation, JAK1/2 Inh (Ruxolitinib) | NCT04279847 |  | Recruiting |
|  |  |  | I/II | Solid Tumors | chemotherapy, hormone therapy (Abiraterone), JAK1/2 inh (Ruxolitinib) | NCT02711137 |  | Terminated |
|  |  |  | I/II | Solid Tumors | anti-PD-1 (Pembrolizumab), IDO1 inh (Epacadostat) | NCT02959437 | ECHO-206 | Terminated |
| RO6870810 (RG 6146) | Hoffmann-La Roche | small molecule inhibitor | I | AML, MDS | - | NCT02308761 |  | Completed |
|  |  |  | I | MM | anti-CD38 (daratumumab) | NCT03068351 |  | Completed |
|  |  |  | I | Solid Tumors | - | NCT01987362 |  | Completed |
|  |  |  | I | Ovarian Cancer, TNBC | anti-PD-L1 (Atezolizumab) | NCT03292172 |  | Terminated |
|  |  |  | I | DLBCL, High-Grade B-cell Lymphoma | BCL2 inh (Venetoclax), anti-CD20 (Rituximab) | NCT03255096 |  | Completed |
| Molibresib (I-BET-762, GSK525762) | GlaxoSmithKline | small molecule inhibitor | I | lymphoma, pancreatic cancer | HDAC inh (Entinostat) | NCT03925428 |  | Withdrawn |
|  |  |  | II | Hematological malignancies | - | NCT01943851 |  | Completed |
|  |  |  | II | Solid Tumors | MEK1/2 inh (Trametinib) | NCT03266159 |  | Withdrawn |
|  |  |  | I | breast cancer | hormone therapy (Fulvestrant) | NCT02964507 |  | Completed |
|  |  |  | Compassionate use access study | Solid Tumors | - | NCT03702036 |  | No longer available |
|  |  |  | I | NUT Midline Carcinoma | - | NCT01587703 |  | Completed |
|  |  |  | I | Solid Tumors | hormone therapy (Abiraterone, Enzalutamide), Prednisone | NCT03150056 |  | Completed |
|  |  |  | I | Healthy volunteers | antibiotic (Rifampicin, Itraconazole) | NCT02706535 |  | Completed |
|  |  |  | I/II | NUT Carcinoma | chemotherapy | NCT04116359 |  | Withdrawn |
| Mivebresib (ABBV-075) | AbbVie | small molecule inhibitor | I | Malignancies | BCL2 inh (Venetoclax) | NCT02391480 |  | Completed |
|  |  |  | I | Myelofibrosis | chemotherapy, JAK1/2 inh (Ruxolitinib) | NCT04480086 |  | Active, not recruiting |
| Trotabresib (CC-90010) | Celgene Corporation | small molecule inhibitor | I | Astrocytoma, Glioblastoma | - | NCT04047303 |  | Active, not recruiting |
|  |  |  | I | Solid tumors | - | NCT03220347 |  | Recruiting |
|  |  |  | I | Glioblastoma | chemotherapy, Radiation | NCT04324840 |  | Recruiting |
|  |  |  | I | Pediatric Cancers | - | NCT03936465 |  | Recruiting |
|  |  |  | I | SCLC | anti-PD-1 (Nivolumab) | NCT03850067 |  | Recruiting |
| Pelabresib (CPI-0610) | MorphoSys | small molecule inhibitor | III | Myelofibrosis | JAK1/2 inh (Ruxolitinib) | NCT04603495 | MANIFEST-2 | Recruiting |
|  |  |  | II | Peripheral Nerve Tumors | - | NCT02986919 |  | Withdrawn |
|  |  |  | I | MM | - | NCT02157636 |  | Completed |
|  |  |  | I | lymphoma | - | NCT01949883 |  | Completed |
|  |  |  | I/II | Myelofibrosis | JAK1/2 inh (Ruxolitinib) | NCT02158858 | MANIFEST | Recruiting |
| BMS-986158 | Bristol Myers Squibb | small molecule inhibitor | I | Pediatric Cancers | - | NCT03936465 |  | Recruiting |
|  |  |  | I | Myelofibrosis | JAK1/2 inh (Ruxolitinib), JAK2 inh (Fedratinib) | NCT04817007 |  | Recruiting |
|  |  |  | I/II | Malignancies | anti-PD-1 (Nivolumab) | NCT02419417 |  | Completed |
|  |  |  | I/II | MM | EZH2 inh (Tazemetostat), MEK1/2 inh (Trametinib), Dexamethasone | NCT05372354 |  | Not yet recruiting |
| ODM-207 | OrionPharma | small molecule inhibitor | I/II | Solid Tumors | - | NCT03035591 | BETIDES | Completed |
| ***Targeting BRD4*** | | | | | | | | |
| PLX2853 | Daiichi Sankyo group | small molecule inhibitor | I/II | Ovarian Cancer | chemotherapy | NCT04493619 |  | Active, not recruiting |
|  |  |  | I/II | Solid tumors | - | NCT03297424 |  | Completed |
|  |  |  | I | AML, High-risk MDS | - | NCT03787498 |  | Completed |
|  |  |  | I/II | PC | PARP inh (Olaparib), hormone therapy (Abiraterone), Prednisone | NCT04556617 |  | Active, not recruiting |
| PLX51107 | Daiichi Sankyo group | small molecule inhibitor | I | AML, MDS | chemotherapy | NCT04022785 |  | Recruiting |
|  |  |  | I | Malignancies | - | NCT02683395 |  | Terminated |
| BI 894999 | Boehringer Ingelheim | small molecule inhibitor | I | Solid tumors | - | NCT02516553 |  | Completed |
| AZD5153 (SRA 515) | AstraZeneca | small molecule inhibitor | I/II | Sarcoma | MEK inh (Selumetinib), anti-PD-L1 (Durvalumab) | NCT05253131 |  | Not yet recruiting |
|  |  |  | I | Solid Tumors | PARP inh (Olaparib) | NCT03205176 |  | Completed |
|  |  |  | I | NHL | BTK inh (Acalabrutinib) | NCT03527147 | PRISM | Completed |
|  |  |  | I/II | AML | BCL2 inh (venetoclax) | NCT03013998 |  | Recruiting |
| ***Targeting EZH2 pathway*** | | | | | | | | |
| ***Targeting EZH2*** | | | | | | | | |
| Tazemetostat | Epizyme Inc | Enhancer of zeste homolog 2 (EZH2) inhibitor | III | FL | IMiD (Lenalidomide), anti-CD20 (Rituximab) | NCT04224493 |  | Recruiting |
| CPI-0209 | Constellation Pharmaceuticals Inc | Enhancer of zeste homolog 2 (EZH2) inhibitor | I/II | Solid Tumors and lymphoma | - | NCT04104776 |  | Recruiting |
| ***Targeting LSD1 pathway*** | | | | | | | | |
| ***Targeting LSD1*** | | | | | | | | |
| Ladademstat (ORY1001, RG-6016) | Oryzon Genomics | TCP-based irreversible LSD1 inhibitor | I | Acute Leukaemia | - | EudraCT2013-002447-29 |  | unknown |
|  |  |  | II | AML | chemotherapy | EudraCT2018-000482-36 | ALICE | unknown |
|  |  |  | II | SCLC, Neuroendocrine Cancer | chemotherapy | NCT05420636 |  | Not yet recruiting |
|  |  |  | I | AML | Flt3 inh (Gilteritinib) | NCT05546580 | FRIDA | Not yet recruiting |
| INCB059872 | Incyte Corporation | TCP-based irreversible LSD1 inhibitor | I | Ewing Sarcoma | - | NCT03514407 |  | Terminated |
|  |  |  | I/II | Solid Tumors, Hematological Neoplasms | chemotherapy, anti-PD-1 (nivolumab) | NCT02712905 |  | Active, not recruiting |
|  |  |  | I/II | Solid Tumors | anti-PD-1 (Pembrolizumab), IDO1 inh (Epacadostat) | NCT02959437 |  | Terminated (Sponsor decision) |
| Pulrodemstat (CC-90011) | Celgene Corporation | reversible LSD1 inhibitor | I | PC | hormone therapy (Abiraterone), Prednisone | NCT04628988 |  | Recruiting |
|  |  |  | II | Malignancies | anti-PD-1 (Nivolumab) | NCT04350463 |  | Recruiting |
|  |  |  | I | SCLC | chemotherapy, anti-PD-1 (Nivolumab) | NCT03850067 |  | Recruiting |
|  |  |  | I | AML | BCL2 inh (Venetoclax), chemotherapy | NCT04748848 |  | Completed |
|  |  |  | I | NHL | antibiotic (Rifampicin, Itraconazole) | NCT02875223 |  | Recruiting |
| Seclidemstat (SP-2577) | Salarius Pharmaceuticals | reversible LSD1 inhibitor | I/II | Solid Tumors | - | NCT05266196 |  | Enrolling by invitation |
|  |  |  | I | Solid Tumors | - | NCT03895684 |  | Completed |
|  |  |  | I | Ewing or Ewing-related Sarcoma | chemotherapy | NCT03600649 |  | Recruiting |
|  |  |  | I | Gynecological Cancer | anti-PD-1 (Pembrolizumab) | NCT04611139 |  | Withdrawn (Salarius discontinued support) |
|  |  |  | I/II | CML, MDS | chemotherapy | NCT04734990 |  | Recruiting |
| ***Targeting PRMT5 pathway*** | | | | | | | | |
| ***Targeting PRMT5*** | | | | | | | | |
| Pemrametostat (GSK-3326595) | GlaxoSmithKline, Epizyme | PRMT5 inhibitor | II | Breast Cancer | - | NCT04676516 | OTT-19-06 | Not yet recruiting |
|  |  |  | I | MDS, AML | chemotherapy | NCT03614728 |  | Terminated |
|  |  |  | I | Solid Tumors, NHL | anti-PD-1 (Pembrolizumab) | NCT02783300 | Meteor 1 | Recruiting |
| PF-06939999 | Pfizer | PRMT5 inhibitor | I | Solid Tumors | chemotherapy | NCT03854227 |  | Active, not recruiting |
| PRT543 | Prelude Therapeutics | PRMT5 inhibitor | I | Malignancies | - | NCT03886831 |  | Active, not recruiting |

Annotation:

1. Cells filled with blue color for drug names indicate that the corresponding drug has been approved by the FDA.
2. For drugs approved by FDA, owing to its proven efficacy and the abundance of the clinical trials, only one representative trial is listed.
3. As HMAs have been approved by the FDA for more than ten years, considering the abundance of their clinical trials and the scope of this review, only trials that testing the combination of HMAs and immunotherapies are listed.

NSCLC non-small cell lung cancer, CMML chronic myelomonocytic leukemia, MDS myelodysplastic syndrome, CRPC castration-resistant prostate cancer, SCLC small-cell lung cancer, UC urothelial carcinoma, CRC colorectal cancer, TNBC triple-negative breast cancer, AML acute myeloid leukemia, ESCC esophageal squamous-cell carcinoma, HL Hodgkin lymphoma, PTCL peripheral T cell lymphoma, CTCL cutaneous T cell lymphoma, NHL non-Hodgkin lymphoma, PMBCL primary mediastinal B-Cell lymphoma, HNSCC head and neck squamous-cell carcinoma, RCC renal cell carcinoma, ENKTCL extranodal NK/T-cell lymphoma, ALL acute lymphoblastic leukemia, DLBCL diffuse large B cell lymphoma, FL follicular lymphoma, CLL chronic lymphocytic leukemia, MCC Merkel cell carcinoma, MM multiple myeloma, CTCL cutaneous T-cell lymphoma, EC esophageal cancer, AEG adenocarcinoma of the esophagogastric junction, GC gastric cancer, MCL mantle cell lymphoma, MF myelofibrosis, MPN myeloproliferative neoplasms, PC prostate cancer, HCC hepatocellular carcinoma, CML chronic myeloid leukemia.

**Supplementary Table 6. Therapeutics targeting cytokines**

| Drug name | Manufacturer | Type of agent | Phase | Disease Area (Selected Indications) | Therapeutic combination | Identifier | | Status |
| --- | --- | --- | --- | --- | --- | --- | --- | --- |
|  |  |  |  |  |  | Trial number | Trial Name |  |
| ***Targeting Interleukins*** | | | | | | | | |
| ***Targeting IL-2*** | | | | | | | | |
| aldesleukin | Clinigen/Novartis | rhIL-2 | III | Melanoma | chemotherapy, G-CSF (filgrastim), IFNα | NCT00006237 | PROCLIVITY01 | Completed |
| ­­THOR-707 (SAR444245) | Synthorx Inc | non-α IL-2 variant | I/II | Solid Tumors | checkpoint inh, anti-EGFR Ab | NCT04009681 | THOR-­­­­­707-101 | Recruiting |
|  |  |  | II | Classic HL | anti-PD-1 (Pembrolizumab) | NCT05179603 | Pegasus Lymphom 205 | Recruiting |
|  |  |  | II | HNSCC | anti-PD-1 (Pembrolizumab), anti-EGFR (Cetuximab) | NCT05061420 |  | Recruiting |
|  |  |  | II | Gastrointestinal Cancer | anti-PD-1 (Pembrolizumab), anti-EGFR (Cetuximab) | NCT05104567 |  | Recruiting |
|  |  |  | I/II | Skin Cancer | anti-PD-1 (Cemiplimab) | NCT04913220 | Pegathor Skin 201 | Recruiting |
|  |  |  | II | Pleural Mesothelioma, NSCLC | anti-PD-1 (Pembrolizumab), chemotherapy | NCT04914897 | Pegathor Lung 202 | Recruiting |
|  |  |  | II | HPV Related Oropharynx Squamous Cell Carcinoma | anti-PD-1 (Cemiplimab) | NCT05535023 |  | Not yet recruiting |
| SHR-1916 | Jiangsu Hengrui Medicine | non-α IL-2 variant | I | Solid Tumors | - | NCT04842630 |  | Recruiting |
| Nemvaleukin alfa (ALKS 4230) | Alkermes plc | non-α IL-2 variant-IL-2Rα fusion protein (blocking the IL-2Rα binding) | II | Solid Tumors | anti-PD-1 (Pembrolizumab) | NCT04592653 | ARTISTRY-3 | Recruiting |
|  |  |  | II | Melanoma | - | NCT04830124 | ARTISTRY-6 | Recruiting |
|  |  |  | I/II | Solid Tumors | anti-PD-1 (Pembrolizumab) | NCT03861793 | ARTISTRY-2 | Recruiting |
|  |  |  | II | HNSCC | anti-PD-1 (Pembrolizumab) | NCT04144517 |  | Active, not recruiting |
|  |  |  | I/II | Solid Tumors | anti-PD-1 (pembrolizumab) | NCT02799095 | ARTISTRY-1 | Active, not recruiting |
|  |  |  | III | Ovarian Cancer, Fallopian Tube Cancer, Primary Peritoneal Cancer | anti-PD-1 (Pembrolizumab) | NCT05092360 | ARTISTRY-7 | Recruiting |
| Simlukafusp alfa (SIM, FAP-IL2v, RO6874281) | Hoffmann la Roche | IL-2 variant-anti-FAP antibody fusion protein | I | Melanoma | anti-PD-1 (Pembrolizumab) | NCT03875079 |  | Active, not recruiting |
|  |  |  | I | RCC | anti-PD-L1 (Atezolizumab), anti-VEGF (Bevacizumab) | NCT03063762 |  | Completed |
|  |  |  | I | Breast Cancer, Head and Neck Cancer | anti-HER2 (Trastuzumab), anti-EGFR (Cetuximab) | NCT02627274 |  | Active, not recruiting |
|  |  |  | II | Head and Neck, Oesophageal and Cervical Cancers | anti-PD-L1 (atezolizumab) | NCT03386721 |  | Completed |
|  |  |  | I/II | PDAC | anti-PD-L1 (atezolizumab) | NCT03193190 | MORPHEUS-PDAC | Active, not recruiting |
| Eciskafusp Alfa (PD-1–IL2v) | Hoffmann-La Roche | IL-2 variant-anti-PD-1 antibody fusion protein | I | Solid Tumors | anti-PD-1 (Atezolizumab) | NCT04303858 |  | Recruiting |
|  |  |  | I | Melanoma | anti-PD-1 (Pembrolizumab) | NCT03875079 |  | Completed |
| CUE-101 (E7-pHLA-IL2-Fc) | Hoffmann-La Roche | IL-2 variant-HLA complex+HPV E7 peptide fusion protein | I | Oropharyngeal Squamous Cell Carcinoma | - | NCT04852328 |  | Recruiting |
|  |  |  | I | HPV16+ HNSCC | anti-PD-1 (Pembrolizumab) | NCT03978689 |  | Recruiting |
| GI-101 (CD80-IgG4-IL-2-Fc) | Cue Biopharma | IL-2 variant-anti-CD80 fusion protein | I/II | Solid Tumors | anti-PD-1 (Pembrolizumab), multi-kinase inh (Lenvatinib), radiation | NCT04977453 |  | Recruiting |
| Cergutuzumab amunaleukin (CEA–IL2v) | Hoffmann-La Roche | IL-2 variant-anti-CEA fusion protein | I | Solid tumors | anti-PD-1 (Atezolizumab) | NCT02350673 |  | Completed |
| MDNA11 | Medicenna Therapeutics | IL-2 variant-rhalbumin fusion protein | I/II | Solid Tumors | checkpoint inh | NCT05086692 | ABILITY | Recruiting |
| Darleukin (L19-IL2, Daromun, Philogen) | Philogen SpA | IL-2-anti-ED-B fibronectin antibody fusion protein | II | Skin Cancer | ADC (L19-TNF) | NCT05329792 | IntriNSiC | Not yet recruiting |
|  |  |  | II | Non-melanoma skin cancer | ADC (L19-TNF) | NCT04362722 | DUNCAN | Recruiting |
|  |  |  | III | Melanoma | ADC (L19-TNF) | NCT02938299 | Pivotal | Recruiting |
|  |  |  | I/II | DLBCL | anti-CD20 (Rituximab) | NCT02957019 |  | Active, not recruiting |
|  |  |  | II | NSCLC | Radiation | NCT02735850 | ImmunoSABR | Withdrawn |
|  |  |  | I | Solid Tumors | - | NCT02086721 | L19-IL2 | Completed |
|  |  |  | II | Melanoma | ADC (L19-TNF) | NCT02076633 |  | Completed |
|  |  |  | I/II | Melanoma | chemotherapy | NCT02076646 |  | Active, not recruiting |
|  |  |  | II | NSCLC | Radiation | NCT03705403 | IMMUNOSABR2 | Recruiting |
|  |  |  | II | Melanoma | - | NCT01253096 |  | Completed |
|  |  |  | I | Pancreatic Cancer | chemotherapy | NCT01198522 |  | Terminated |
|  |  |  | I/II | Solid Tumors | - | NCT01058538 |  | Completed |
|  |  |  | II | Melanoma | chemotherapy | NCT01055522 |  | Terminated |
|  |  |  | III | Melanoma | - | NCT03567889 | NeoDREAM | Recruiting |
| STK-012 | Synthekine | α/β-IL-2 variant | I | Solid Tumors | anti-PD-1 (Pembrolizumab) | NCT05098132 |  | Recruiting |
| RG6292 (RO7296682) | Hoffmann la Roche | anti-CD25 mAb | I | Solid Tumors | - | NCT04158583 |  | Active, not recruiting |
|  |  |  | I | Solid Tumors | anti-PD-L1 (Atezolizumab) | NCT04642365 |  | Recruiting |
| XTX202 | Xilio Therapeutics | conditionally-activated IL-2 | I/II | Solid Tumors | - | NCT05052268 |  | Recruiting |
| ***Targeting IL-15*** | | | | | | | | |
| NIZ985 (hetIL-15) | Admune Therapeutics | IL-15-IL-15Rα fusion protein | I | Solid Tumors, Lymphoma | anti-PD-1 (Spartalizumab) | NCT04261439 |  | Recruiting |
|  |  |  | I | Solid Tumors | anti-PD-1 (Spartalizumab) | NCT02452268 |  | Completed |
| XmAb24306 (RO7310729, RG6323) | Xencor Inc | IL-15-IL-15Rα fusion protein | I | MM | anti-CD38 (Daratumumab) | NCT05243342 |  | Recruiting |
|  |  |  | I | Solid Tumors | anti-PD-L1 (Atezolizumab) | NCT04250155 |  | Recruiting |
| Inbakicept (N-803, ALT-803) | ImmunityBio | IL-15-IL-15Rα fusion protein | I | Healthy volunteers | - | NCT03381586 | QUILT-1.004 | Completed |
|  |  |  | II | AML | Cell Therapy (Haplo NK) | NCT03050216 | QUILT-3.033 | Completed |
|  |  |  | II/III | Bladder Cancer | cancer vaccine (BCG) | NCT03022825 | QUILT-3.032 | Recruiting |
|  |  |  | I | Solid Tumors | - | NCT01946789 |  | Completed |
|  |  |  | II/III | NSCLC | anti-PD-1 (Pembrolizumab) | NCT05096663 |  | Recruiting |
|  |  |  | II | AML, MDS | - | NCT03365661 | QUILT-3.034 | Withdrawn |
|  |  |  | PK Sub-study | Malignancies | cancer vaccine (BCG) | NCT04142359 |  | Terminated |
|  |  |  | II | Ovarian Cancer, Fallopian Tube Cancer, Primary Peritoneal Cancer | - | NCT03054909 |  | Active, not recruiting |
|  |  |  | I | MM | - | NCT02099539 | QUILT-3.005 | Unknown status |
|  |  |  | I/II | Solid Tumors | cancer vaccine (ETBX-011) | NCT03127098 | QUILT-3.040 | Completed |
|  |  |  | I/II | NMIBC | cancer vaccine (BCG) | NCT02138734 |  | Recruiting |
|  |  |  | I/II | Hematological Neoplasms | - | NCT01885897 |  | Completed |
|  |  |  | I/II | B Cell NHL | anti-CD20 (Rituximab) | NCT02384954 | QUILT-3.002 | Terminated |
|  |  |  | II | AML, MDS | - | NCT02989844 |  | Active, not recruiting |
|  |  |  | I | Pancreatic Cancer | chemotherapy | NCT02559674 | QUILT-2.001 | Completed |
|  |  |  | I/II | NSCLC | anti-PD-1 (Nivolumab) | NCT02523469 |  | Active, not recruiting |
|  |  |  | I | Malignancies | Cell Therapy (Natural Killer Cells) | NCT02890758 |  | Active, not recruiting |
|  |  |  | II | GEJC, HNSCC | anti-PD-1 (Pembrolizumab), Cell Therapy (PD-L1 t-haNK) | NCT04847466 |  | Recruiting |
|  |  |  | II | MCC | anti-PD-L1 (Avelumab), Cell Therapy (haNK™) | NCT03853317 | QUILT-3.063 | Terminated |
|  |  |  | II | NSCLC | anti-VEGFR2 (Ramucirumab), anti-PD-L1 (Atezolizumab) | NCT05007769 | RAN | Withdrawn |
|  |  |  | I/II | HNSCC | PD-L1 x TGFβ bsAb (M7824), cancer vaccine (TriAd vaccine) | NCT04247282 |  | Active, not recruiting |
|  |  |  | I/II | PC | PD-L1 x TGFβ bsAb (M7824), cancer vaccine (MVA-BN-Brachyury, FPV-Brachyury), IDO1 inhibitor (Epacadostat) | NCT03493945 | QuEST1 | Recruiting |
|  |  |  | III | NSCLC | anti-PD-1 (pembrolizumab), chemotherapy | NCT03520686 | QUILT 2.023 | Recruiting |
|  |  |  | II | MCC | Cell Therapy (aNK (NK-92)) | NCT02465957 | QUILT-3.009 | Active, not recruiting |
|  |  |  | II | Small Bowel Cancer, CRC | cancer vaccine (CV301), PD-L1 x TGFβ bsAb (MSB0011359C), ADC (NHS-IL12) | NCT04491955 |  | Recruiting |
|  |  |  | I/II | Squamous Cell Carcinoma | chemotherapy, radiation, cancer vaccine (ETBX-011, ETBX-021, ETBX-051, ETBX-061, GI-4000, GI-6207, GI-6301), anti-PD-L1 (Avelumab), anti-VEGF (bevacizumab), Cell Therapy (haNK) | NCT03387111 | QUILT-3.090 | Active, not recruiting |
|  |  |  | II | Solid Tumors | anti-PD-1 (pembrolizumab, nivolumab), anti-PD-L1 (atezolizumab, avelumab), anti-CD38 (durvalumab), PD-L1 t-haNK | NCT03228667 | QUILT-3.055 | Active, not recruiting |
|  |  |  | I/II | TNBC | chemotherapy, Cell Therapy (PD-L1 t-haNK), ADC (Sacituzumab Govitecan-Hziy) | NCT04927884 |  | Active, not recruiting |
|  |  |  | I | Solid Tumors | Cell Therapy (M-CENK) | NCT04898543 | QUILT-3.076 | Recruiting |
|  |  |  | I/II | AML | chemotherapy, Cell Therapy (Cytokine-induced killer cells), IL-2 | NCT01898793 |  | Active, not recruiting |
|  |  |  | I | HNSCC | Cell Therapy (CIML NK cell), anti-CTLA-4 (Ipilimumab) | NCT04290546 |  | Recruiting |
|  |  |  | II | MM | Cell Therapy (Expanded Natural Killer Cells) | NCT03003728 |  | Withdrawn |
|  |  |  | II | Pancreatic Cancer | chemotherapy, radiation, Cell Therapy (PD-L1 t-haNK) | NCT04390399 |  | Recruiting |
|  |  |  | I/II | Pancreatic Cancer | chemotherapy, radiation, cancer vaccine (ETBX-011, GI-4000), anti-PD-L1 (Avelumab), anti-VEGF (bevacizumab), Cell Therapy (haNK) | NCT03329248 | QUILT-3.060 | Active, not recruiting |
|  |  |  | I/II | Pancreatic Cancer | chemotherapy, radiation, cancer vaccine (ETBX-011, GI-4000), anti-PD-L1 (Avelumab), anti-VEGF (bevacizumab), Cell Therapy (haNK) | NCT03387098 | QUILT-3.070 | Unknown status |
|  |  |  | I/II | NHL | chemotherapy, radiation, cancer vaccine (ETBX-061), anti-PD-L1 (Avelumab), anti-VEGF (bevacizumab), Cell Therapy (haNK) | NCT03169790 | QUILT-3.052 | Withdrawn |
|  |  |  | I/II | TNBC | chemotherapy, radiation, cancer vaccine (ETBX-011, ETBX-051, ETBX-061, GI-4000, GI-6207, GI-6301), anti-PD-L1 (Avelumab), anti-VEGF (bevacizumab), Cell Therapy (haNK) | NCT03175666 | QUILT-3.049 | Withdrawn |
|  |  |  | I/II | Pancreatic Cancer | chemotherapy, radiation, cancer vaccine (ETBX-011, ETBX-021, ETBX-051, ETBX-061, GI-4000, GI-6207, GI-6301), anti-PD-L1 (Avelumab), anti-VEGF (bevacizumab), Cell Therapy (haNK) | NCT03586869 | QUILT-3.080 | Unknown status |
|  |  |  | I/II | Ovarian Cancer | chemotherapy, radiation, cancer vaccine (ETBX-011, ETBX-021, ETBX-051, ETBX-061, GI-4000, GI-6301), anti-PD-L1 (Avelumab), anti-VEGF (bevacizumab), Cell Therapy (haNK) | NCT03197584 | QUILT-3.051 | Withdrawn |
|  |  |  | I/II | Urothelial Carcinoma | chemotherapy, radiation, cancer vaccine (ETBX-011, ETBX-021, ETBX-051, ETBX-061, GI-4000, GI-6207, GI-6301), anti-PD-L1 (Avelumab), anti-VEGF (bevacizumab), Cell Therapy (haNK) | NCT03197571 | QUILT-3.048 | Withdrawn |
|  |  |  | I/II | HNSCC | chemotherapy, radiation, cancer vaccine (ETBX-011, ETBX-021, ETBX-051, ETBX-061, GI-4000, GI-6207, GI-6301), anti-PD-L1 (Avelumab), anti-PD-1 (nivolumab), anti-VEGF (bevacizumab), Cell Therapy (haNK) | NCT03169764 | QUILT-3.047 | Withdrawn |
|  |  |  | I/II | CRC | chemotherapy, radiation, cancer vaccine (ETBX-011, ETBX-021, ETBX-051, ETBX-061, GI-4000, GI-6207, GI-6301), anti-PD-L1 (Avelumab), anti-PD-1 (nivolumab), anti-VEGF (bevacizumab), Cell Therapy (haNK) | NCT03169777 | QUILT-3.050 | Withdrawn |
|  |  |  | I/II | NSCLC | chemotherapy, radiation, cancer vaccine (ETBX-011, ETBX-021, ETBX-051, ETBX-061, GI-4000, GI-6207, GI-6301), anti-PD-L1 (Avelumab), anti-PD-1 (nivolumab), anti-VEGF (bevacizumab), Cell Therapy (haNK) | NCT03169738 | QUILT-3.044 | Withdrawn |
|  |  |  | I/II | Pancreatic Cancer | chemotherapy, radiation, cancer vaccine (ETBX-011, GI-4000), anti-PD-L1 (Avelumab), anti-VEGF (bevacizumab), Cell Therapy (haNK) | NCT03136406 | QUILT-3.039 | Active, not recruiting |
|  |  |  | I/II | MCC | chemotherapy, radiation, cancer vaccine (ETBX-051, ETBX-061, GI-6301), anti-PD-L1 (Avelumab), anti-VEGF (bevacizumab), Cell Therapy (haNK) | NCT03167164 | QUILT-3.045 | Withdrawn |
|  |  |  | I/II | Melanoma | chemotherapy, radiation, cancer vaccine (ETBX-011, ETBX-051, ETBX-061, GI-6207, GI-6301), anti-PD-L1 (Avelumab), anti-PD-1 (nivolumab), anti-VEGF (bevacizumab), Cell Therapy (haNK) | NCT03167177 | QUILT-3.046 | Withdrawn |
|  |  |  | I/II | Chordoma | chemotherapy, radiation, cancer vaccine (ETBX-051, ETBX-061, GI-6301), anti-PD-L1 (Avelumab), anti-EGFR (Cetuximab), Cell Therapy (haNK) | NCT03647423 | QUILT-3.091 | Withdrawn |
|  |  |  | II | AML | Cell Therapy (CIML NK cell infusion) | NCT02782546 |  | Recruiting |
|  |  |  | I/II | TNBC | chemotherapy, cancer vaccine (ETBX-011, ETBX-051, ETBX-061, GI-4000, GI-6207, GI-6301), anti-PD-L1 (Avelumab), Cell Therapy (haNK) | NCT03387085 | QUILT-3.067 | Active, not recruiting |
|  |  |  | II | TNBC | chemotherapy, cancer vaccine (ETBX-011, ETBX-051, ETBX-061, GI-4000, GI-6207, GI-6301), anti-PD-L1 (Avelumab), Cell Therapy (haNK) | NCT03554109 | QUILT-3.057 | Withdrawn |
|  |  |  | I/II | CRC | chemotherapy, cancer vaccine (ETBX-011, ETBX-021, ETBX-051, ETBX-061, GI-4000, GI-6207, GI-6301), anti-PD-L1 (Avelumab), Cell Therapy (haNK) | NCT03563157 | QUILT 3.071 | Active, not recruiting |
|  |  |  | I/II | HCC | chemotherapy, cancer vaccine (ETBX-051, ETBX-061, GI-4000, GI-6207, GI-6301), anti-PD-L1 (Avelumab), Cell Therapy (haNK) | NCT03563170 | QUILT-3.072 | Withdrawn |
|  |  |  | II | Pancreatic Cancer | chemotherapy, cancer vaccine (ETBX-011, ETBX-021, ETBX-051, ETBX-061, GI-4000, GI-6207, GI-6301), anti-PD-L1 (Avelumab), Cell Therapy (haNK) | NCT03563144 | QUILT-3.088 | Withdrawn |
|  |  |  | II | NSCLC | chemotherapy, cancer vaccine (ETBX-011, ETBX-051, ETBX-061, GI-4000, GI-6301), anti-PD-L1 (Avelumab), Cell Therapy (haNK) | NCT03574649 | QUILT-2.024 | Withdrawn |
| SHR-1501 | Jiangsu Hengrui Medicine | IL-15-IL-15Rα fusion protein | I | Malignancies | - | NCT04025957 |  | Recruiting |
|  |  |  | I | Malignancies | anti-PD-L1 (SHR-1316) | NCT03995472 |  | Recruiting |
|  |  |  | I | Malignancies | - | NCT04025957 |  | Unknown status |
| BJ-001 | BJ Bioscience | IL-15-IL-15Rα-integrin-binding motif fusion protein | I | Solid Tumors | anti-PD-1 (pembrolizumab) | NCT04294576 | FIH | Recruiting |
| NKTR-255 | Nkarta Therapeutics | polymer conjugated IL-15 | I/II | Solid Tumors | anti-EGFR (Cetuximab) | NCT04616196 |  | Recruiting |
|  |  |  | I | Large B-Cell Lymphoma | chemotherapy, cancer vaccine (Lisocabtagene Maraleucel) | NCT05359211 |  | Not yet recruiting |
|  |  |  | I | MM, NHL | anti-CD20 (Rituximab), anti-CD38 (Daratumumab) | NCT04136756 |  | Recruiting |
|  |  |  | I | B-cell Acute Lymphoblastic Leukemia | Cell therapy (CD19/CD22 CAR T cells), chemotherapy | NCT03233854 |  | Recruiting |
|  |  |  | II | UC | anti-PD-L1 (Avelumab) | NCT05327530 | JAVELIN Bladder Medley | Not yet recruiting |
| ***Targeting IL-10*** | | | | | | | | |
| Ilodecakin (Pegilodecakin, AM0010) | ARMO BioSciences | PEGylated human IL-10 | Expanded Access Program | Solid Tumors | - | NCT03554434 |  | No longer available |
|  |  |  | I | Healthy Volunteers | - | NCT04194892 | Willow 3 | Completed |
|  |  |  | I | Healthy Volunteers | - | NCT03267732 | Willow 1 | Completed |
|  |  |  | I | Healthy Volunteers | - | NCT03381547 | Willow 2 | Completed |
|  |  |  | II | NSCLC | anti-PD-1 (Nivolumab) | NCT03382912 | Cypress 2 | Terminated (unfavorable benefit ratio) |
|  |  |  | II | NSCLC | anti-PD-1 (Pembrolizumab) | NCT03382899 | Cypress 1 | Terminated (unfavorable benefit ratio) |
|  |  |  | III | Pancreatic Cancer | chemotherapy | NCT02923921 | Sequoia | Completed |
|  |  |  | I | Solid Tumors | anti-PD-1 (Pembrolizumab, nivolumab), chemotherapy | NCT02009449 | IVY | Active, not recruiting |
| ***Targeting IL-12*** | | | | | | | | |
| NHS-IL12 (M9241) | National Cancer Institute (USA) | IL-12-anti-DNA-histone H1 complex mAb fusion protein | I/II | Solid Tumors | PD-L1 x TGFβ bsAb (Bintrafusp Alfa), HDAC inh (Entinostat) | NCT04708470 |  | Recruiting |
|  |  |  | I | Non-Prostate Genitourinary Malignancies | PD-L1 x TGFβ bsAb (M7824), Radiation | NCT04235777 |  | Recruiting |
|  |  |  | I/II | Kaposi Sarcoma | PD-L1 x TGFβ bsAb (M7824), radiation | NCT04303117 |  | Recruiting |
|  |  |  | I | Solid Tumors | anti-PD-1 (Avelumab) | NCT02994953 | COMBO | Terminated |
|  |  |  | I | Solid Tumors | - | NCT01417546 |  | Completed |
|  |  |  | II | PC | Radiation | NCT05361798 |  | Not yet recruiting |
|  |  |  | I | Breast Cancer | PD-L1 x TGFβ bsAb (Bintrafusp Alfa), Radiation | NCT04756505 | REINA | Recruiting |
|  |  |  | I/II | Pancreatic Cancer | PD-L1 x TGFβ bsAb (M7824), radiation | NCT04327986 |  | Completed |
|  |  |  | II | CRC, ICC | chemotherapy | NCT05286814 |  | Not yet recruiting |
|  |  |  | I/II | PC | hormone therapy, Prednisone, PD-L1 x TGFβ bsAb (M7824), chemotherapy | NCT04633252 |  | Recruiting |
|  |  |  | I/II | HPV-associated malignancies | cancer vaccine (PDS0101), PD-L1 x TGFβ bsAb (M7824) | NCT04287868 |  | Recruiting |
|  |  |  | II | Bowel Cancers, CRC | cancer vaccine (CV301), PD-L1 x TGFβ bsAb (MSB0011359C), IL-15 superagonist (N-803) | NCT04491955 |  | Recruiting |
| SON 1010 (IL12FHAB) | Sonnet Biotherapeutics | IL-12-FHAB fusion protein | I | Solid Tumors | - | NCT05352750 |  | Recruiting |
|  |  |  | Early phase I | Healthy Volunteers | - | NCT05408572 |  | Not yet recruiting |
| GEN-1 (EGEN-001) | Celsion Corporation | plasmid-encoded IL-12 | I/II | Ovarian Carcinoma, Fallopian Tube Carcinoma, Primary Peritoneal Carcinoma | chemotherapy | NCT03393884 | OVATION 2 | Recruiting |
|  |  |  | I | Ovarian Carcinoma, Fallopian Tube Carcinoma, Primary Peritoneal Carcinoma | chemotherapy | NCT02480374 |  | Completed |
|  |  |  | I | Ovarian Cancer | - | NCT00473954 |  | Completed |
|  |  |  | I/II | CRC | - | NCT01300858 |  | Terminated |
|  |  |  | I | Ovarian Carcinoma, Fallopian Tube Carcinoma, Primary Peritoneal Carcinoma | chemotherapy | NCT01489371 |  | Completed |
|  |  |  | II | Ovarian Carcinoma, Fallopian Tube Carcinoma, Primary Peritoneal Carcinoma | - | NCT01118052 |  | Completed |
|  |  |  | I | Ovarian Cancer | - | NCT00137865 |  | Terminated |
| MEDI 0457 (INO-9012) | AstraZeneca/Inovio Pharmaceuticals | plasmid-encoded IL-12 | I/II | HCC | cancer vaccine (GNOS-PV02), anti-PD-1 (Pembrolizumab) | NCT04251117 |  | Recruiting |
|  |  |  | I | PC | - | NCT02514213 |  | Completed |
|  |  |  | I/II | UC | cancer vaccine (INO-5401), anti-PD-L1 (Atezolizumab) | NCT03502785 |  | Active, not recruiting |
|  |  |  | I/II | Glioblastoma | cancer vaccine (INO-5401), anti-PD-1 (Cemiplimab), Radiation, chemotherapy | NCT03491683 |  | Active, not recruiting |
|  |  |  | I/II | HPV-associated Head and Neck Cancer | anti-PD-L1 (Durvalumab) | NCT03162224 |  | Completed |
|  |  |  | II | HPV+ OPSCC | anti-PD-L1 (Durvalumab) | NCT04001413 |  | Withdrawn |
|  |  |  | I | BRCA1/2 Mutation Carrier | cancer vaccine (INO-5401) | NCT04367675 |  | Recruiting |
|  |  |  | I | HPV-associated Cancers | cancer vaccine (INO-3106) | NCT02241369 |  | Completed |
|  |  |  | I | Glioblastoma | cancer vaccine (GNOS-PV01) | NCT04015700 |  | Recruiting |
|  |  |  | II | HPV-associated Cancers | anti-PD-L1 (Durvalumab) | NCT03439085 |  | Active, not recruiting |
|  |  |  | I | Solid Tumors | cancer vaccine (INO-1400, INO-1401) | NCT02960594 | TRT-001 | Completed |
|  |  |  | I/II | HNSCC | - | NCT02163057 |  | Completed |
|  |  |  | I/II | Cervical Cancer | - | NCT02172911 |  | Completed |
|  |  |  | II | Uterine Cervical Neoplasms | Radiation, chemotherapy | NCT02501278 |  | Withdrawn |
| Tavokinogene telseplasmid | OncoSec Medical | plasmid-encoded IL-12 | II | Melanoma | anti-PD-1 (Pembrolizumab) | NCT03132675 | Keynote695 | Recruiting |
|  |  |  | I | Healthy Volunteers | - | NCT05298046 |  | Not yet recruiting |
|  |  |  | II | TNBC | anti-PD-1 (Pembrolizumab), chemotherapy | NCT03567720 |  | Recruiting |
|  |  |  | I | Healthy Volunteers | - | NCT05313152 |  | Not yet recruiting |
|  |  |  | II | Melanoma | anti-PD-1 (nivolumab) | NCT04526730 |  | Recruiting |
|  |  |  | II | HNSCC | - | NCT02345330 |  | Terminated |
|  |  |  | II | MCC | - | NCT01440816 | MCC | Completed |
|  |  |  | II | Melanoma | - | NCT01502293 | IL-12MEL | Completed |
|  |  |  | II | CTCL | - | NCT01579318 | CTCL | Terminated |
|  |  |  | II | HNSCC | IDO1 inh (Epacadostat), anti-PD-1 (Pembrolizumab), cancer vaccine (CORVax) | NCT03823131 |  | Recruiting |
| INXN 2001 (ad-RTS-hIL-12) | ZIOPHARM Oncology Inc | adenovirus encoding activable IL-12 | I | Glioblastoma | - | NCT03679754 |  | Completed |
|  |  |  | II | Glioblastoma | anti-PD-1 (Cemiplimab) | NCT04006119 |  | Completed |
|  |  |  | I | Glioblastoma | anti-PD-1 (Nivolumab) | NCT03636477 |  | Completed |
|  |  |  | I | Glioblastoma, glioma | - | NCT02026271 |  | Completed |
|  |  |  | I/II | Breast Cancer | - | NCT02423902 |  | Completed |
|  |  |  | I/II | Brian Tumors | - | NCT03330197 |  | Terminated |
|  |  |  | I/II | Melanoma | - | NCT01397708 |  | Completed |
|  |  |  | II | Breast Cancer | chemotherapy | NCT01703754 |  | Completed |
| ***Targeting TGF-β*** | | | | | | | | |
| ***Targeting TGF-β*** | | | | | | | | |
| NIS793 | Novartis | antagonistic pan-TGF-β mAb | III | PDAC | Chemotherapy | NCT04935359 | daNIS-2 | Recruiting |
|  |  |  | II | CRC | anti-PD-1 (Tislelizumab), anti-VEGF (Bevacizumab), Chemotherapy | NCT04952753 | daNIS-3 | Recruiting |
|  |  |  | II | PDAC | anti-PD-1 (Spartalizumab), Chemotherapy | NCT04390763 | daNIS-1 | Recruiting |
|  |  |  | I/II | MF | JAK inh (Ruxolitinib) | NCT04097821 | ADORE | Recruiting |
|  |  |  | I | Solid Tumors | anti-PD-1 (Spartalizumab) | NCT02947165 |  | Completed |
|  |  |  | I | MDS | Anti-IL-1β (Canakinumab) | NCT04810611 |  | Recruiting |
| AVID-200 (BMS-986416) | Formation Biologics, Bristol-Myers Squibb | TGF-βR ECD-Fc fusion protein (TGF-β1/3 Trap) | I | Solid Tumors | anti-PD-1 (Nivolumab) | NCT04943900 |  | Recruiting |
|  |  |  | I | MF |  | NCT03895112 | MPN-RC 118 | Active, not recruiting |
|  |  |  | I | Solid Tumors |  | NCT03834662 |  | Active, not recruiting |
| Trabedersen | Oncotelic Therapeutics | antisense against TGF-β2 | I | Melanoma, Pancreatic Cancer, CRC |  | NCT00844064 |  | Completed |
|  |  |  | II | Glioblastoma |  | NCT00431561 |  | Completed |
| ***Targeting TGF-βR*** | | | | | | | | |
| Vactosertib (TEW-7197) | MedPacto | small molecule inhibitor | II | GC | anti-PD-L1 (Durvalumab) | NCT04893252 |  | Not yet recruiting |
|  |  |  | II | GC | anti-VEGFR2 (Ramucirumab), Chemotherapy | NCT04656002 |  | Not yet recruiting |
|  |  |  | II | NSCLC | anti-PD-1 (Pembrolizumab) | NCT04515979 |  | Recruiting |
|  |  |  | I | PDAC | Chemotherapy | NCT04258072 |  | Not yet recruiting |
|  |  |  | II | Ph-negative MPN |  | NCT04103645 |  | Recruiting |
|  |  |  | II | UC | anti-PD-L1 (Durvalumab) | NCT04064190 |  | Not yet recruiting |
|  |  |  | II | CRC | anti-PD-1 (Pembrolizumab) | NCT03844750 |  | Recruiting |
|  |  |  | I/II | NSCLC | anti-PD-L1 (Durvalumab) | NCT03732274 |  | Active, not recruiting |
|  |  |  | I/II | CRC |  | NCT03724851 |  | Active, not recruiting |
|  |  |  | I/II | GC | Chemotherapy | NCT03698825 |  | Active, not recruiting |
|  |  |  | I/II | Pancreatic Cancer | Chemotherapy | NCT03666832 | MP-PDAC-01 | Recruiting |
|  |  |  | I | MM | Pomalidomide | NCT03143985 |  | Recruiting |
|  |  |  | I | MDS |  | NCT03074006 |  | Completed |
|  |  |  | I | Solid Tumors |  | NCT02160106 |  | Completed |
| YL-13027 | Shanghai Yingli Pharmaceutical | small molecule inhibitor | I | Solid Tumors |  | NCT05228600 |  | Not yet recruiting |
|  |  |  | I | Solid Tumors |  | NCT03869632 |  | Recruiting |
| ***Targeting activation of L-TGF-β*** | | | | | | | | |
| SRK-181 | Scholar Rock | selective L-TGF-β1 antagonistic mAb | I | Solid Tumors | anti-PD-1/PD-L1 | NCT04291079 | DRAGON | Recruiting |
| ABBV-151 | AbbVie | selective L-TGF-β1 antagonistic mAb | I | Solid Tumors | anti-PD-1 (Budigalimab) | NCT03821935 |  | Recruiting |
| PF-06940434 | Pfizer | αvβ8 integrin mAb | I | Solid Tumors | anti-PD-1 (Sasanlimab) | NCT04152018 |  | Recruiting |
| ***Targeting chemokines*** | | | | | | | | |
| ***Targeting CXCR4*** | | | | | | | | |
| Balixafortide (POL6326) | Spexis | small molecule inhibitor | I/II | Breast Cancer | microtubule inh (Eribulin), chemotherapy | NCT04826016 | POLTER | Not yet recruiting |
|  |  |  | III | Breast Cancer | microtubule inh (Eribulin) | NCT03786094 | FORTRESS | Active, not recruiting |
|  |  |  | I | Breast Cancer | - | NCT01837095 |  | Completed |
|  |  |  | I | Healthy Volunteers | Stem cell transplantation | NCT01841476 |  | Completed |
|  |  |  | I/II | Hematological Neoplasms | Stem cell transplantation | NCT01413568 |  | Completed |
|  |  |  | II | MM | Stem cell transplantation | NCT01105403 |  | Completed |
| Motixafortide (BL-8040) | Biokine Therapeutics, BioLineRx | small molecule inhibitor | I | Healthy Volunteers | - | NCT05293171 |  | Active, not recruiting |
|  |  |  | I/II | CML | - | NCT02115672 |  | Withdrawn |
|  |  |  | II | PDAC | anti-PD-1 (Pembrolizumab), Chemotherapy | NCT02826486 | COMBAT | Active, not recruiting |
|  |  |  | I/II | AML | anti-PD-L1 (Atezolizumab) | NCT03154827 |  | Terminated |
|  |  |  | II | Pancreatic Cancer | anti-PD-1 (Pembrolizumab) | NCT02907099 |  | Active, not recruiting |
|  |  |  | II | T-cell Acute Lymphoblastic Leukemia/Lymphoma | chemotherapy | NCT02763384 |  | Recruiting |
|  |  |  | II | AML | chemotherapy | NCT01838395 |  | Completed |
|  |  |  | II | AML | chemotherapy | NCT02502968 | BLAST | Unknown status |
|  |  |  | II | Pancreatic Cancer | anti-CTLA-4 (Cemiplimab), chemotherapy | NCT04543071 |  | Recruiting |
|  |  |  | I/II | GC, EC, GEJC | anti-PD-L1 (atzolizumab) | NCT03281369 | MORPHEUS-pancreatic ductal adenocarcinoma (M-PDAC) | Recruiting |
|  |  |  | I/II | PDAC | anti-PD-L1 (atzolizumab) | NCT03193190 | MORPHEUS-gastric cancer (M-GC) | Active, not recruiting |
|  |  |  | III | MM | G-GSF, Stem cell transplantation | NCT03246529 | GENESIS | Active, not recruiting |
|  |  |  | II | Hematological Malignancies | Stem cell transplantation | NCT02639559 |  | Active, not recruiting |
|  |  |  | II | MDS | anti-thymocyte globulin, Methylprednisolone, immunosuppressant (Cyclosporine) | NCT02462252 |  | Completed |
|  |  |  | I | Healthy Volunteers | - | NCT02073019 |  | Completed |
| Mavorixafor (X4P-001) | Sanofi, X4 Pharmaceuticals | small molecule inhibitor | I | Melanoma | anti-PD-1 (Pembrolizumab) | NCT02823405 | X4P-001-MELA | Completed |
|  |  |  | I | Healthy Volunteers | - | NCT02680782 |  | Terminated |
|  |  |  | I/II | TNBC | - | NCT05103917 |  | Enrolling by invitation |
|  |  |  | I/II | ccRCC | anti-PD-1 (Nivolumab) | NCT02923531 |  | Completed |
|  |  |  | I/II | ccRCC | VEGFR1/2/3 inh (axitinib) | NCT02667886 |  | Active, not recruiting |
| ***Targeting CXCR2*** | | | | | | | | |
| AZD5069 | AstraZeneca | small molecule inhibitor | I/II | PC | hormone therpay (Enzalutamide) | NCT03177187 | ACE | Active, not recruiting |
|  |  |  | I | Healthy Volunteers | - | NCT01332903 |  | Completed |
|  |  |  | I | Healthy Volunteers | - | NCT00953888 |  | Completed |
|  |  |  | I | Healthy Volunteers | - | NCT01100047 | JSMAD | Completed |
|  |  |  | I | Healthy Volunteers | - | NCT01051505 |  | Completed |
|  |  |  | I/II | PDAC | anti-PD-L1 (MEDI4736) | NCT02583477 |  | Completed |
|  |  |  | I/II | Solid Tumors | anti-PD-L1 (MEDI4736), anti-CTLA-4 (tremelimumab) | NCT02499328 | SCORES | Active, not recruiting |
| SX-682 | Syntrix Biosystems Inc | CXCR1/2 small molecule inhibitor | I | MDS | - | NCT04245397 |  | Recruiting |
|  |  |  | I | Melanoma | anti-PD-1 (Pembrolizumab) | NCT03161431 |  | Recruiting |
|  |  |  | I/II | CRC | anti-PD-1 (Nivolumab) | NCT04599140 | STOPTRAFFIC-1 | Recruiting |
|  |  |  | I | PDAC | anti-PD-1 (Nivolumab) | NCT04477343 |  | Recruiting |
|  |  |  | I/II | Solid Tumors | PD-L1 x TGFβ bispecific Ab (M7824), cancer vaccine (MVA-BN-CV301, FPV-CV301) | NCT04574583 |  | Active, not recruiting |

Annotation:

1. Cells filled with blue color for drug names indicate that the corresponding drug has been approved by the FDA.
2. For drugs approved by FDA, owing to its proven efficacy and the abundance of the clinical trials, only one representative trial is listed.

HL Hodgkin lymphoma, HNSCC head and neck squamous cell carcinoma, NSCLC non-small cell lung cancer, UC urothelial carcinoma, RCC renal cell carcinoma, NHL non-Hodgkin lymphoma, PC prostate cancer, PDAC pancreatic ductal adenocarcinoma, DLBCL diffuse large B cell lymphoma, MM multiple myeloma, AML acute myeloid leukemia, MDS myelodysplastic syndrome, NMIBC non–muscle-invasive bladder cancer, GEJC gastroesophageal junction cancer, MCC Merkel cell carcinoma, CRC colorectal cancer, TNBC triple-negative breast cancer, HCC hepatocellular carcinoma, ICC intrahepatic cholangiocarcinoma, OPSCC oropharyngeal squamous cell carcinoma, CTCL cutaneous T-cell lymphoma, MF myelofibrosis, GC gastric cancer, MPN myeloproliferative neoplasms, CML chronic myeloid leukemia, EC esophageal cancer, ccRCC clear cell renal cell carcinoma.
